# Towards the Development of an In vivo Chemical Probe for Cyclin G Associated Kinase (GAK)

**DOI:** 10.3390/molecules24224016

**Published:** 2019-11-06

**Authors:** Christopher R. M. Asquith, James M. Bennett, Lianyong Su, Tuomo Laitinen, Jonathan M. Elkins, Julie E. Pickett, Carrow I. Wells, Zengbiao Li, Timothy M. Willson, William J. Zuercher

**Affiliations:** 1 Structural Genomics Consortium, UNC Eshelman School of Pharmacy, University of North Carolina at Chapel Hill, Chapel Hill, NC 27599, USA; julie.pickett@unc.edu (J.E.P.); carrow.wells@unc.edu (C.I.W.); tim.willson@unc.edu (T.M.W.); 2 Department of Pharmacology, School of Medicine, University of North Carolina at Chapel Hill, Chapel Hill, NC 27599, USA; 3 Structural Genomics Consortium and Target Discovery Institute, Nuffield Department of Clinical Medicine, University of Oxford, Oxford OX3 7DQ, UK; james.bennett@ndm.ox.ac.uk (J.M.B.); jon.elkins@sgc.ox.ac.uk (J.M.E.); 4 Drumetix Laboratories, Greensboro, NC 27409, USA; lsu@drumetix.com (L.S.); zli@drumetix.com (Z.L.); 5 School of Pharmacy, Faculty of Health Sciences, University of Eastern Finland, 70211 Kuopio, Finland; tuomo.laitinen@uef.fi; 6 Structural Genomics Consortium, Universidade Estadual de Campinas-UNICAMP, Campinas, São Paulo 13083-886, Brazil; 7 Lineberger Comprehensive Cancer Center, University of North Carolina at Chapel Hill, Chapel Hill, NC 27599, USA

**Keywords:** cyclin G associated kinase (GAK), kinase inhibitor design, in vivo kinase design, 1-aminobenzotriazole (ABT)

## Abstract

SGC-GAK-1 (**1**) is a potent, selective, cell-active chemical probe for cyclin G-associated kinase (GAK). However, **1** was rapidly metabolized in mouse liver microsomes by cytochrome P450-mediated oxidation, displaying rapid clearance in liver microsomes and in mice, which limited its utility in in vivo studies. Chemical modifications of **1** that improved metabolic stability, generally resulted in decreased GAK potency. The best analog in terms of GAK activity in cells was 6-bromo-*N*-(1*H*-indazol-6-yl)quinolin-4-amine (**35**) (IC_50_ = 1.4 μM), showing improved stability in liver microsomes while still maintaining a narrow spectrum activity across the kinome. As an alternative to scaffold modifications we also explored the use of the broad-spectrum cytochrome P450 inhibitor 1-aminobenzotriazole (ABT) to decrease intrinsic clearance of aminoquinoline GAK inhibitors. Taken together, these approaches point towards the development of an in vivo chemical probe for the dark kinase GAK.

## 1. Introduction

Cyclin G-associated kinase (GAK) is a 160 kDa member of the numb-associated kinase (NAK) family of serine/threonine kinases [1]. GAK was originally identified as a directly associated partner of cyclin G, and is ubiquitously expressed across tissues [2]. Within the cell, GAK localizes to the Golgi complex, cytoplasm, and nucleus [3]. GAK has been genetically associated with a diverse range of biological processes. Genome-wide association studies have identified single nucleotide polymorphisms in GAK that are associated with susceptibility to Parkinson’s disease [4]. Knock down of GAK has shown that it is required for the maintenance of centrosome maturation and progression through mitosis [5]. Conversely, GAK was found to be overexpressed in osteosarcoma cell lines and tissues, where it contributes to proliferation and survival [6].

**Figure 1:**
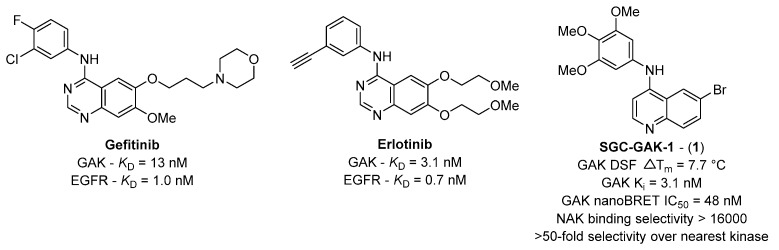
Examples of reported GAK inhibitors.

The biological implications of GAK depletion or over-expression prompt the question of whether there is therapeutic utility in targeting its kinase domain with small molecules. GAK is a collateral target of several clinical kinase inhibitors, including the epidermal growth factor receptor (EGFR) inhibitors erlotinib and gefitinib (Figure 1), but until recently potent selective cell-active inhibitors of GAK had not been identified [7] and thus the biological consequences of selective GAK inhibition remained untested. As part of an ongoing effort to generate chemical probes for dark kinases, we recently described SGC-GAK-1 (**1**) as a high-quality cell-active chemical probe for GAK (Figure 1) [8,9]. SGC-GAK-1 (**1**) showed IC_50_ = 48 nM in a GAK live cell target engagement assay and proved to be more than 50-fold selective for GAK across a panel of over 400 human kinases.

GAK is a potential therapeutic target for the treatment of androgen-independent prostate cancer. Expression of GAK is known to increase during prostate cancer progression to androgen independence and has been positively correlated with Gleason scores in resections from prostate cancer patients [10]. We have demonstrated that **1** potently inhibits the viability of prostate cancer cells that contain constitutively-active splice variants of the androgen receptor [9]. These results prompted us to identify a chemical probe of GAK that is suitable for use in rodent models of prostate cancer. We now describe our efforts in the optimization of 4-aminoquinolines as potential in vivo chemical probes for GAK.

## 2. Results

### 2.1. Initial Investigation of P450 Metabolism of **1**

The metabolic stability of **1** was evaluated in mouse liver microsomes (MLMs) to assess the potential for use as an in vivo chemical probe. SGC-GAK-1 (**1)** was incubated with MLMs in the presence or absence of added NADPH, and the compound was quantified at various time points over an hour. No degradation of **1** was observed in the absence of NADPH. However, **1** was rapidly consumed (T_1/2_ = 5.7 min; Cl_int_ = 990 mL/min/kg) in the presence of NADPH. This result suggests that **1** would be prone to rapid first pass metabolism when dosed in vivo.

**Figure 2:**
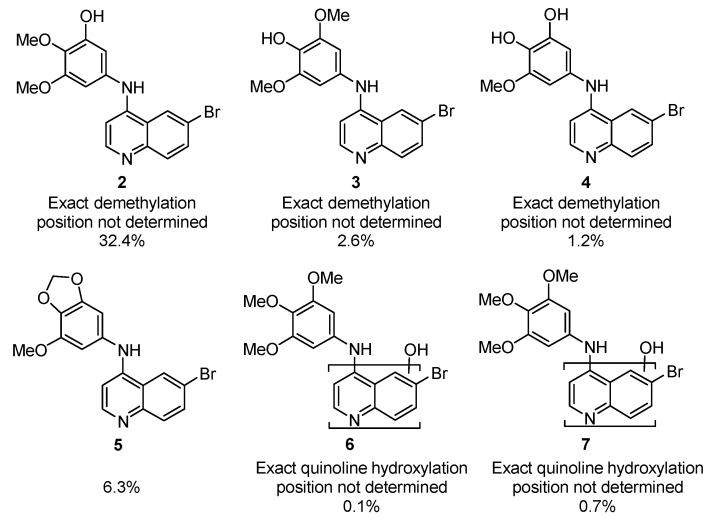
Metabolites of **1** identified (**2**–**7**) along with percentage abundance in respect to the parent compound. Compounds are ordered by HPLC retention time (see Supplementary Materials).

The necessity of NADPH for the metabolism of **1** is consistent with cytochrome P450-mediated oxidation. To further understand the metabolic fate of **1**, we conducted a metabolite identification experiment. 10 µM of **1** was incubated for 5 min (ratio of 0.5 with respect to propranolol, *n* = 4) in MLMs at 37 °C in the presence of an excess of NADPH before quenching with methanol. After centrifugation, the supernatant was subjected to analysis by LCMS, and structures of metabolites were assigned on the basis of their molecular weight and fragmentation pattern. The primary metabolite **2** was identified as a loss of CH_2_ from the 3,4,5-trimethoxyaniline (Figure 2 and Figure S1). Minor metabolites were identified as an alternate loss of CH_2_ from the 3,4,5-trimethoxyaniline (**3**), the loss of 2 × CH_2_ (**4**), and the formation of a cyclic acetal (**5**). Formation of **5** likely occurs via the product of a mono-demethylation intercepting an intermediate oxocarbenium ion formed during a second oxidative demethylation. Finally, two minor species with quinoline ring oxidation (**6** and **7**) were observed at low abundance. The liver microsomal stability and the metabolite identification experiments identified cytochrome P450-mediated oxidation as the primary route of metabolism of **1**, with the trimethoxyaniline as the major metabolic liability.

### 2.2. Synthesis of Analogs of **1**

**Scheme 1:**
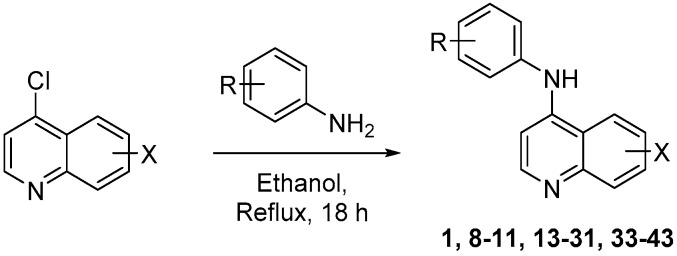
General displacement synthetic procedure.

**Scheme 2:**
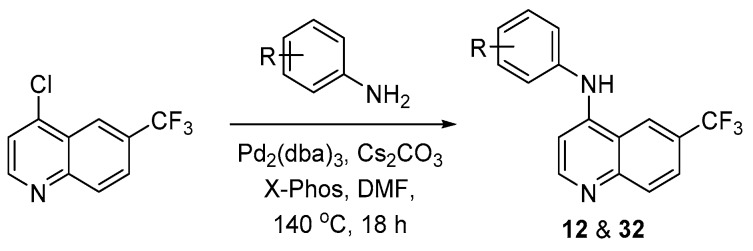
General Buchwald-Hartwig cross-coupling procedure.

In an attempt to address the poor metabolic stability of **1**, we prepared a focused array of analogs in which the trimethoxyaniline was replaced by various bioisosteres (**8**–**43**). Compounds **8**–**11**, **13**–**31**, and **33**–**43** were synthesized through nucleophilic aromatic displacement of the corresponding 4-chloroquinolines (Scheme 1) to furnish the products with good to excellent yields (54–89%) [8,9,11,12,13,14]. Additional analogs (**23**, **24**, and **27**) were synthesized by the same route with modest yields (12–38%) due to the reduced nucleophilicity of their respective anilines. An alternative route employing a Buchwald–Hartwig cross-coupling with 4-chloro-6-trifluoromethylquinoline enabled access to the 6-trifluoromethyl analogs (**12**) and (**32**) in 21% and 17% yields, respectively (Scheme 2) [8,9,12,13,14].

### 2.3. Metabolite Invesitigation Focused 4-Anilinoquinolines Analogs **8**–**13**

The metabolic stability of the 4-anilinoquinolines (**8**–**13**) were evaluated in MLMs (Table 1). Half-lives for metabolic clearance were normalized to propranolol in each experimental run to control for variations in the MLM preparations. Changing the substitution of the core quinoline heterocycle to 6,7-dimethoxy, as in **8**, marginally increased stability relative to the 6-bromo substitution and maintained an aniline *O*-demethylation species as the primary metabolite. Modification of the aniline portion had a wide range effects on metabolic stability. The 2,3-methylenedioxy analog **9** showed similar stability to **8**, with an aniline ring oxidation as the primary metabolite. Surprisingly, modification of the trimethoxy to the difluoro (**10** and **11**) yielded compounds with equal or less metabolic stability than **1**. Metabolite identification experiments with the fluorinated analogs continued to show aniline oxidation as the primary site of metabolism: direct ring oxidation was observed with **10** and hydroxyl replacement of a fluorine with **11**. However, replacement of the trimethoxyaniline with a perfluorinated aniline resulted in a significantly more stable analog **12**. Finally, the similar stability of 6-Br quinoline **1** and 6-CF_3_ quinoline **13** showed that switching of the 6-substituents had little effect on metabolism.

The analogs (**8**–**13**) were also assessed for GAK activity in a Time-resolved fluorescence energy transfer (TR-FRET) ligand-binding displacement assay and a live cell NanoBRET target engagement assay (Table 2) [8,9,12,13,14,15]. The 6,7-dimethoxyquinoline **8** displayed a high GAK potency biochemically and in cells (IC_50_ = 25 nM), as did the methylenedioxy compound **9** (IC_50_ = 22 nM). The two difluoroaniline isomers **10** and **11** had nanomolar *K*_i_ values in the biochemical assay but showed a larger drop-off in potency in the live cell NanoBRET assay, suggesting that they had poorer cell penetration. The metabolically stable pentafluoro aniline analog **12** had only weak GAK affinity in the biochemical assay (*K*_i_ = 880 nM), and did not progress to cellular evaluation. Finally, **13** showed a 4-fold loss in cellular GAK potency relative to **1**. Considering both the MLM stability and cellular GAK activity of these initial analogs, we selected the methylenedioxy compound **9** for further rounds of optimization.

### 2.4. Design and Tesing of 4-Anilinoquinolines Analogs **14**–**43**

**Figure 3:**
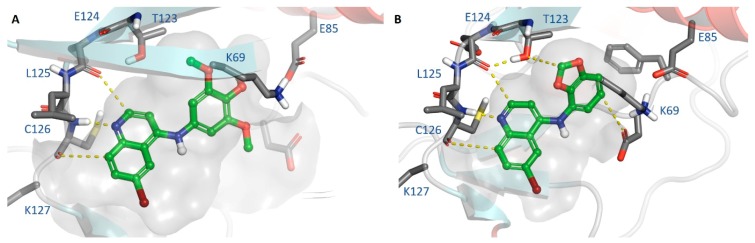
Docking of **1** (**A**) and **9** (**B**) into the ATP binding site of GAK (PDB: 5Y7Z). The protein is shown as a ribbon view with key amino acids as sticks with carbon atoms in grey, nitrogen in blue, oxygen in red, and sulphur in yellow. The GAK inhibitor is shown as sticks with carbon atoms in green, nitrogen in blue, and oxygen in red. The ATP-binding pocket is shown in light grey. Hydrogen bonds from the hinge residues to the inhibitor are highlighted as yellow dashed lines.

**Figure 4:**
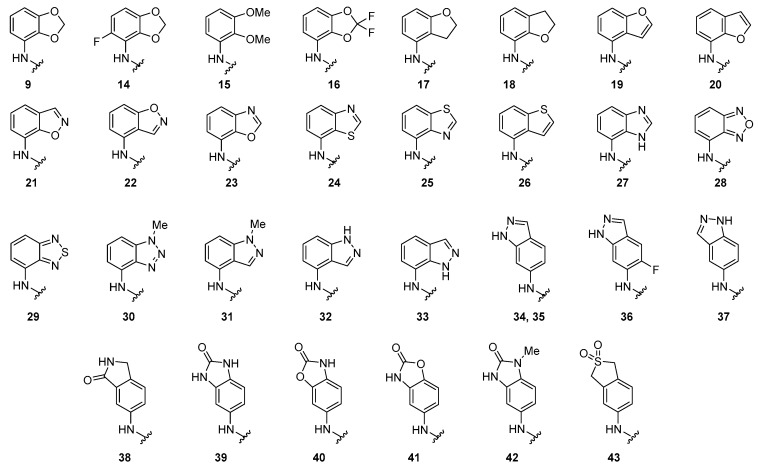
Aniline fragments incorporated into compounds **9** and **14**–**43** in Table 3.

Docking of **1** and **9** into a model of GAK derived from the co-crystal structure with gefitinib (Figure 3) was used to define key structural features for favorable binding using Schrödinger Maestro [16]. The compounds were minimized using LigPrep before docking into the ATP-binding site prepared from PDB structure 5Y7Z [17]. As expected, the most favorable docking pose placed the quinoline nitrogen in a position to accept a hydrogen bond from the backbone NH of the hinge region residue C126. The 5-membered ring of the methylenedioxy in **9** adopted a conformation where it could form a weak hydrophobic interaction with T123 [18]. Based on these results, we designed and prepared a series of isosteric replacements of the substituted aniline (**14**–**43**) that would maintain a favorable interaction with GAK and be less prone to oxidative metabolism. The analogs were assessed for GAK activity in the biochemical and live cell assays and for metabolic stability in MLM (Figure 4 and Table 3).

Results of the initial compound evaluation underscored the difficulty in the concurrent optimization of GAK potency and metabolic stability (Table 4). Consistent with the docking model supporting a favorable fit for bicyclic aniline fragments, a majority of the analogs (**14–43**) demonstrated potent GAK activity in a biochemical assay (*K*_i_ < 100 nM). Importantly, all GAK active analogs (**14**–**43**) maintained good NAK family selectivity in biochemical assays (Table S11). Unfortunately, potent biochemical GAK activity did not necessarily translate to potent GAK activity in cells. Most of the heterocycles showed lower GAK activity in the live cell NanoBRET assay compared to the methylenedioxy analog **9**, although six compounds demonstrated submicromolar IC_50_ values in cells: benzofurans **19** and **20**, benzoxazole **23**, benzothiazole **24**, benzothiadiazole **29**, and indazole **32**. Interestingly, we observed that a switch of the 2,3-substitution in the aniline heterocycle to the corresponding 3,4-substitution often led to greatly increased metabolic stability, with six compounds demonstrating T_1/2_ ratios ≥ 2-fold higher than **9**: indazoles **34** and **35**, benzimidazolone **39** and **42**, and benzoxazolidinones **40** and **41**. The benzimidazolone **39** was the most stable heterocycle in MLM with a T_1/2_ of >200 min and T_1/2_ ratio > 25. Unfortunately, the matched pair of indazoles **34** and **35** were the only compounds to maintain any measurable activity in the live cell GAK NanoBRET assay, with IC_50_ = 1.4 and 2.6 μM, respectively. However, this level of potency was 30- to 60-fold lower than SGC-GAK-1 (**1**) and did not meet our minimum criteria for use as an in vivo chemical probe for GAK.

### 2.5. Metabolite ID Profiling and Kinome Scan of **35**

**Figure 5:**
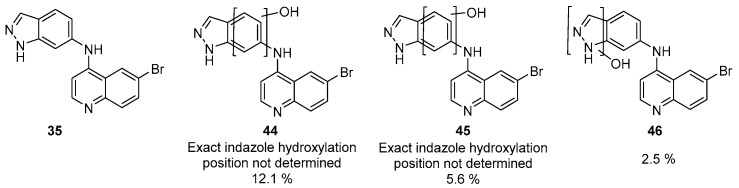
Compound **35** and corresponding metabolites (**44–46**) identified along with percentage abundance ordered by retention time (see Supplementary Materials).

Despite the limited utility of indazole **35** as an in vivo chemical probe for GAK we decided to perform a metabolite identification experiment to elaborate the origin of its improved stability. Following incubation in MLM, metabolites were identified that were nucleated around the aniline ring. Oxidation of the phenyl ring of the indazole yielded the major metabolites (**44**–**45**). Minor metabolites were identified as hydroxylation on an indazole nitrogen (**46**) (Figure 5). Notably, the quinoline core remained less metabolically labile than the indazole heterocycle.

**Figure 6:**
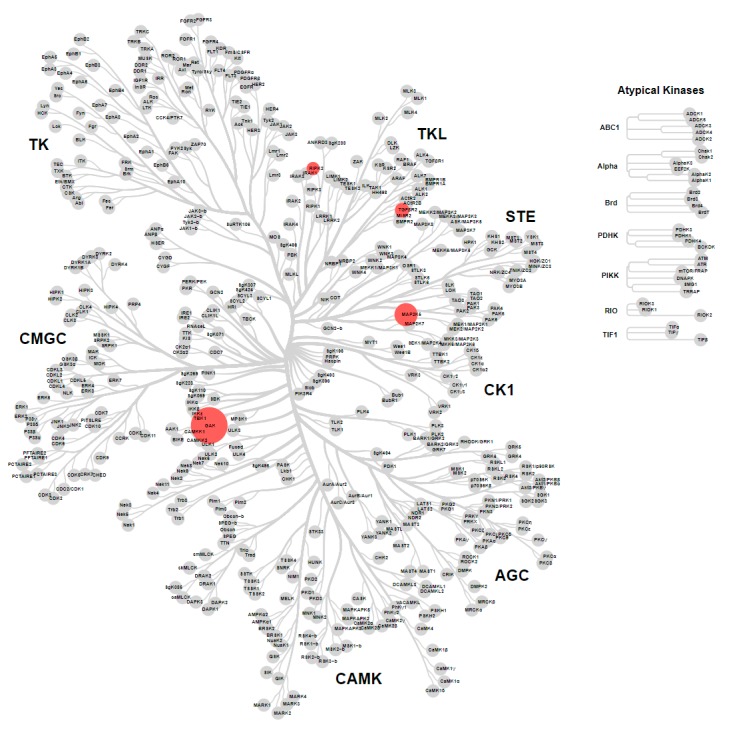
Representation of the human kinome based on sequence identity-displaying KinomeScan results for **35** screened at 1 μM. Red dots represent hits differentiated by size; kinases inhibited at 1 μM GAK, 90%; MEK5, 89%; TGFBR2, 82%; RIPK2, 71% (see Supplementary Materials).

Compound **35** was also submitted to a KinomeScan assay to determine whether the indazole moiety had changed the selectivity profile across > 400 human kinases. The results (Figure 6) showed activity on only four kinases (GAK, MEK5, TGFBR2 & RIPK2) with a 1 µM concentration of **35**. So, although activity on GAK was reduced compared to **1**, the indazole **35** still maintained a narrow spectrum kinase inhibition profile in vitro.

### 2.6. Electronic Fuikui Modelling Aniline of 4-Anilinoquinolines

**Figure 7:**
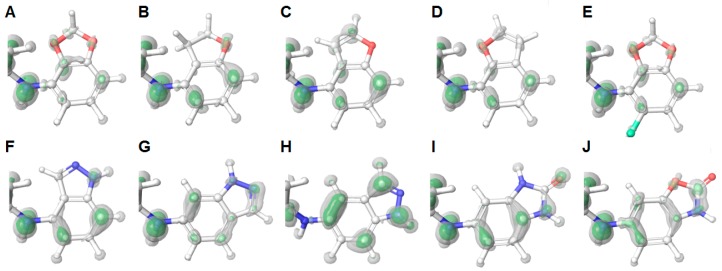
Electronic mapping of the scaffolds showing Fuikui minus visualized with two isovalues, 0.002 (white) and 0.004 (green): (**A**) **9**, (**B**) **17**, (**C**) **19**, (**D**) **20**, (**E**) **14**, (**F**) **32**, (**G**) **35**, (**H**) **37**, (**I**) **39**, (**J**) **40**.

In order to rationalize the observed structure activity relationships within the MLM results and gage propensity for reactive oxidation events to occur, we attempted to model the compound electronics using frontier molecular orbital theory and Fukui indices [19] (Figure 7). The negative values for f− correspond to regions that lose electron density when the molecule undergoes an electrophilic attack or when the molecule itself acts as a nucleophile [19]. Unfortunately, the results did not align with the structure activity relationships observed in the MLM.

### 2.7. Modelling of Lead Compounds in XenoSite

**Figure 8:**
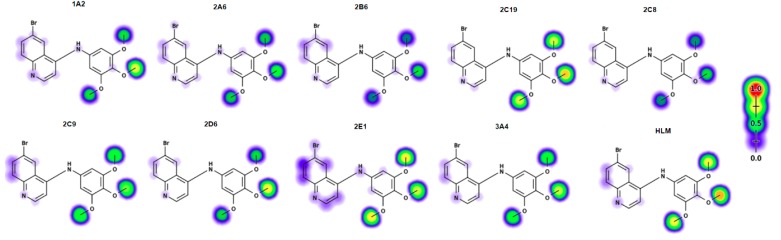
Compound **1** in XenoSite’s P450 Metabolism P450 1.0 algorithm.

**Figure 9:**
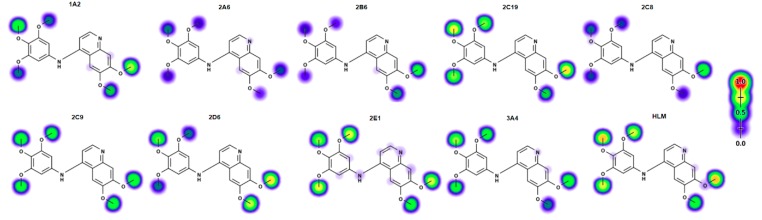
Compound **8** in XenoSite’s P450 Metabolism P450 1.0 algorithm.

**Figure 10:**
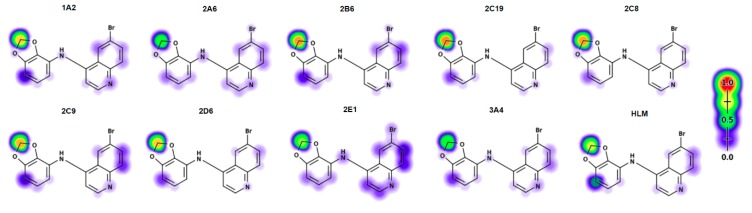
Compound **9** in XenoSite’s P450 Metabolism P450 1.0 algorithm.

**Figure 11:**
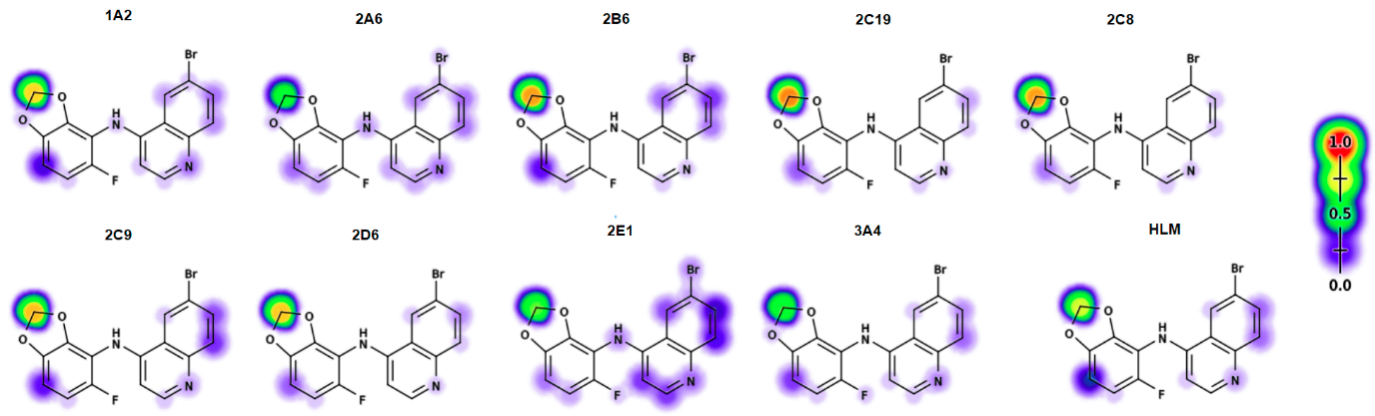
Compound **14** in XenoSite’s P450 Metabolism P450 1.0 algorithm.

**Figure 12:**
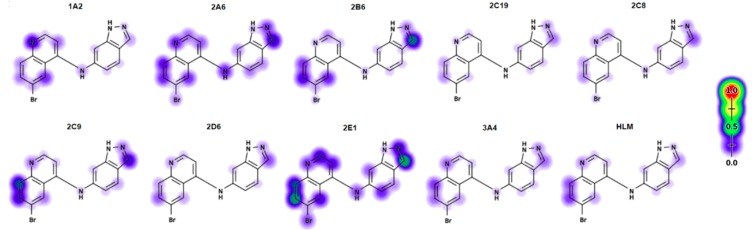
Compound **35** in XenoSite’s P450 Metabolism P450 1.0 algorithm.

**Figure 13:**
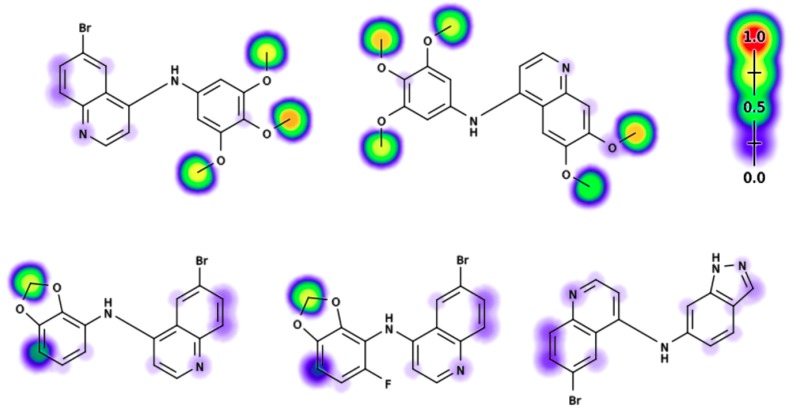
Summary image highlighting the predicted metabolic profiles of **1**, **8**, **9**, **14**, and **35** in XenoSite’s P450 Metabolism P450 1.0 algorithm.

We also analyzed the five lead compounds that retained GAK activity (**1**, **8**, **9**, **14**, and **35**) using the P450 modelling software XenoSite (https://swami.wustl.edu/xenosite/) [20]. The XenoSite models were trained on a large set of publicly available P450 metabolism data [20,21,22] to predict the sites of metabolism by CYP1A2, -2A6, -2B6, -2C19, -2C8, -2C9, -2D6, -2E1 and -3A4 (Figure 8, Figure 9, Figure 10, Figure 11, Figure 12 and Figure 13) [20].

Several functional groups were predicted by XenoSite to be metabolically labile, including the trimethoxy functionality of **1** and **8** and the benzo[*d*][1,3]dioxol-4-yl of **9** and **14**. The bromine substitution of **1** appeared to deactivate the core quinoline structure compared with **8**, but this did not translate to a large increase in stability (Figure 8; Figure 9). This analysis highlights that the main metabolism is likely to occur on the aniline ring system. The methylenes of the benzo[*d*][1,3]dioxol-4-yl of **9** and **14** were also predicted to be a hot spot for metabolism (Figure 10; Figure 11). In contrast, the indazole of **35** showed less propensity for metabolism, a prediction that was consistent with the observed MLM experimental data (Figure 12).

### 2.8. 4-Anilinoquinolines Stablisation with ABT

Having modelled the P450 metabolism, we measured the stability of analogs **8**, **9**, **14**, and **35** in MLM in comparison to **1.** The experiments were run with and without the broad spectrum P450 inhibitor, 1-aminobenzotriazole (ABT) [23] (Table 4). In the absence of ABT, we observed increased stability of **8** and **35**, with a 5- to 20-fold increase in the half-life in MLM compared to **1** (Table 5). However, the benzo[*d*][1,3]dioxol-4-yl analogs **9** and **14** showed higher intrinsic clearance and shorter half-lives. In the presence of ABT, **1**, **8**, and **35** showed greatly increased stability. Surprisingly, ABT did not, however, increase the stability of analogs **9** and **14**. ABT is reported to inhibit 100% of CYP2A6 and -3A4 activity but residual activity (up to 20%) can be measured with CYP1A2, -2B6, -2C8, -2C9, -2C19, and -2D6 [24]. CYP2C9 was particularly resistant to inhibition by ABT, with 60% of the activity remaining. A separate study indicated that ABT caused a concentration-dependent inhibition of CYP1A2, -2B6, -2C9, -2C19, -2D6, and -3A4 [25]. Taken together, our results suggest that CYP2C9 or -2C8 may be responsible for the metabolism of the benzo[*d*][1,3]dioxol-4-yl group of **9** and **14** in MLM. XenoSite predicts a metabolism hotspot for these analogs with CYP2C9 or -2C8 that ABT may be unable to block in MLM.

### 2.9. In Vivo Stabilisation of **1** and **8** with ABT

**Figure 14:**
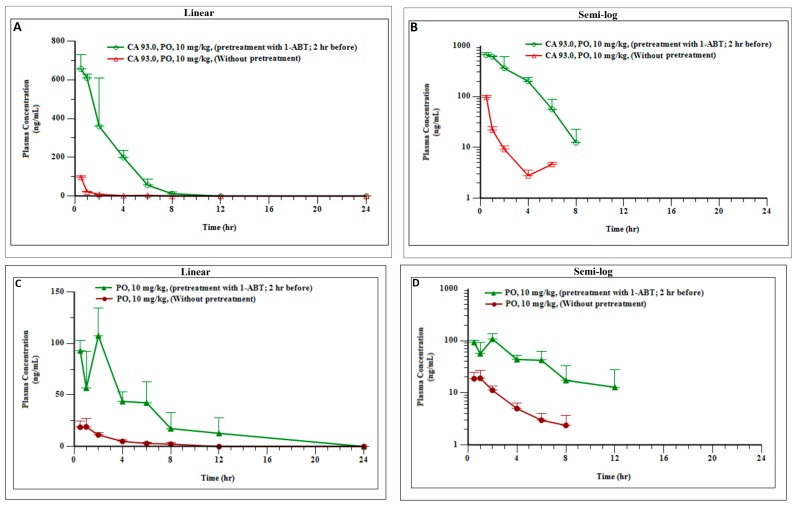
Plasma concentration profiles of **1** (**A**,**B**) and **8** (**C**,**D**) in mice (n = 18) following a 10 mg/kg po dose with (green) or without (red) 2 h pretreatment with 50 mg/kg ABT.

Having been unable to identify a potent (sub-µM) cell-active GAK inhibitor with improved metabolic stability in MLM, we decided to test an alternative strategy to enable dosing of a GAK chemical probe in vivo. Since in vitro inhibition of P450 enzymes by cotreatment with ABT in MLM had greatly increased the metabolic stability of **1** and **8**, we explored the effect of co-dosing in vivo. A pharmacokinetic experiment was performed in which C57BL/6 mice were dosed orally with 10 mg/kg of **1** or **8** with or without 2 h pre-treatment with 50 mg/kg ABT (Table 5). As predicted by the microsomal stability experiment, orally dosed **1** or **8** were rapidly cleared in mice with a T_1/2_ < 1 h and a low C_max_ (<100 ng/mL). In contrast, pretreatment with ABT resulted in a dramatically extended T_1/2_ with a >20-fold higher exposure for **1** and 8-fold higher exposure for **8**. In the absence of ABT, the plasma concentration**s** fell below the cellular IC_50_ after approximately 1 h for **1** and after 2 h for **8**. In contrast, pretreatment with ABT resulted in a sustained plasma concentration of **1** at 2-fold above the cellular IC_50_ for 6 h with a 10 mg/kg dose (Figure 14A,B). This shift was even more pronounced for **8** with 2-fold above the cellular IC_50_ maintained for >8 h with a 10 mg/kg dose (Figure 14C,D). These results demonstrate that the decreased metabolism observed upon co-dosing of ABT in MLM can translate to increased exposure of 4-anilinoquinoline GAK inhibitors in vivo.

## 3. Discussion

Chemical probes are potent and selective modulators of protein function that are useful as reagents to elucidate the biology of their targets [26,27]. The use of chemical probes in phenotypic models of disease can also provide powerful evidence to support target validation for drug discovery. Positive results in cellular experiments naturally lead to interest in using the chemical probe for target validation in animal models. However, many chemical probes are not amenable to use in vivo because they are unable to attain sufficient exposure to their target protein to support valid experiments. The pharmacokinetic profiles of probes can be addressed through careful chemical optimization of the series but at the cost of significant investments of time and resources.

A common pharmacokinetic liability of compounds is that they are rapidly metabolized by cytochrome P450 enzymes in the liver and cleared from the animal, a process known as first pass metabolism. As a result, compounds are routinely evaluated for stability in MLM prior to progression to in vivo pharmacokinetic studies [28]. It has been well established that molecules with low MLM stability are prone to rapid first pass metabolism in vivo. The challenge of optimizing compounds to improve in vivo pharmacokinetic properties while maintaining their target potency and selectivity has vexed many medicinal chemistry programs, including our own exploration of the dark kinase GAK.

The application of the machine learning algorithms such as XenoSite to develop predictive models of P450 metabolism has greatly improved the design of molecules with fewer metabolic liabilities. The XenoSite model accurately predicted the reduced metabolic liability of indazole **35**, which showed improved stability in liver microsomes compared to **1**. However, the modest cellular GAK activity of **35** (IC_50_ = 1.4 μM) meant that it was unsuitable for use as a chemical probe. Unfortunately, all of the other 4-aminoquinolines analogs with improved metabolic stability had even poorer GAK activity. We found that replacement of the 3,4,5-trimethoxy substitution with 5-membered heterocycles annulated at the aniline 2,3-positions yielded several compounds that maintained high GAK activity in biochemical assays, but without improvement in microsomal stability. On the other hand, compounds with 3,4-annulation of heterocycles in general proved to be significantly more metabolically stable, but at the cost of weakened GAK activity. The identification of 3,4,5-trimethoxyphenyl heterocyclic replacement groups with increased microsomal stability is a valuable learning tool. The 3,4,5-trimethoxyphenyl group is a commonly encountered substructure in ATP-competitive inhibitors of protein kinases and in a variety of other molecules with biological activity, such as the antibiotic trimethoprim, the antihypertensive reserpine, the anti-inflammatory colchicine, and the DHFR-inhibitor trimetrexate for the treatment of leiomyosarcoma [29]. Thus, other medicinal chemistry campaigns requiring isosteres of the 3,4,5-trimethoxyphenyl group may consider incorporation of select heterocyclic replacements identified here.

Although our original goal of the chemical optimization of **1** to decrease intrinsic metabolism while retaining potent GAK activity remained unrealized, we were able to use co-dosing with ABT to reduce P450 metabolism and increase in vivo exposure. ABT is a potent inhibitor of CYP3A4, CYP2D6, and several other P450 enzymes, and has been successfully employed to enhance the pharmacokinetics of metabolically labile compounds [24,30]. Although not an approved drug for administration to patients, co-dosing with ABT to block oxidative metabolism in animal models is significantly less demanding of time and resources than chemical optimization, and may be broadly applicable for the development of in vivo chemical probes [31]. For example, co-dosing of ABT with the antifungal APX001 increased exposure of the compound and enhanced its efficacy in a murine model of invasive candidiasis [32]. Long-term dosing of ABT is also possible. ABT has displayed a well-behaved pharmacokinetic profile over five days when dosing with an osmotic pump [25]. Minimal side effects were observed for chronic ABT dosing, the most prominent of which was delayed gastric emptying [33]. ABT can also be utilized in species other than mice for in vivo studies. ABT is a competent cytochrome P450 inhibitor in rats, guinea pigs, and other species, and has been used to enhance pharmacokinetic exposure in these animals [30,34]. Our results further extend the utility of ABT by demonstrating an increase of in vivo exposure for certain 4-anilinoquinoline GAK inhibitors in mice.

Not all 4-anilinoquinolines showed reduced MLM metabolism in the presence of ABT. The 4-anilinoquinolines **9** and **14** containing the benzo[*d*][1,3]dioxol-4-yl group had shorter half-lives than **1** in MLM and were still rapidly metabolized in the presence of ABT. Notably, the XenoSite model predicted that the benzo[*d*][1,3]dioxol-4-yl group was a hotspot for metabolism by CYP2C9 and CYP2C8, two P450s that are not strongly inhibited by ABT. For compounds such as these, it may be possible to use the co-dosing of other P450 inhibitors such as atipamezole to block CYP2C8/9 activity [35]. The development of a range of broad spectrum P450 inhibitors with overlapping profiles that can be used safely in animal models to block P450 metabolism would be a powerful addition to the medicinal chemist’s toolbox.

## 4. Materials and Methods

### 4.1. Computational Methods

Small molecules were prepared using the ligprep module of Schrödinger (Schrödinger Release 2018-4: LigPrep, Schrödinger, LLC, New York, NY, USA, 2018.) Structures were superimposed using flexible ligand alignment functionality (Schrödinger Release 2018-4: Maestro, Schrödinger, LLC, New York, NY, USA, 2018) using compound **8** as a template and employing the Bemis–Murcko method for common substructure recognition [14].

### 4.2. Biology Methods

#### 4.2.1. NAK Family TR-FRET Assays

Assays performed as previously described [12].

#### 4.2.2. GAK NanoBRET Assay

Assays performed as previously described [9].

#### 4.2.3. Metabolite Identification

Compounds were incubated at a concentration of 10 µM with mouse liver microsomes in the presence of an excess of NADPH. The duplicate incubations were performed in 0.5 mL 96-well plates in a shaking water bath maintained at 37 °C for 30 min and quenched by the addition of an equal volume of methanol (see Supplementary Materials for full details and reports).

#### 4.2.4. Pharmacokinetic Profiling

Pharmacokinetic profiling was performed by SAI Life Sciences (Hyderabad, India). All procedures of the present study were in accordance with the guidelines provided by the Committee for the Purpose of Control and Supervision of Experiments on Animals (CPCSEA) as published in the Gazette of India, 15 December 1998. Prior approval of the Institutional Animal Ethics Committee (IAEC) was obtained before initiation of the study. Healthy male C57BL/6 mice (8–12 weeks old) weighing between 20 and 35 g were procured from (Envigo Research Private Ltd., City, Hyderabad, India). Three mice were housed in each cage. Temperature and humidity were maintained at 22 ± 3 °C and 30–70%, respectively, and illumination was controlled to give a sequence of a 12 h light and 12 h dark cycle. Temperature and humidity were recorded by an automatically controlled data logger system. All animals were provided a laboratory rodent diet (Envigo Research Private Ltd., Hyderabad, India). Reverse osmosis water treated with ultraviolet light was provided ad libitum. Eighteen male mice were weighed and divided in to two groups (Group 1 and Group 2), with nine mice in each group. Animals in Group 1 were administered orally with a solution formulation of **1** or **8** at 10 mg/kg dose. Animals in Group 2 were administered orally with a solution formulation of ABT (2 h before dosing of **1** or **8**) at 50 mg/kg dose. After 2 h of ABT administration, animals were administered orally with solution formulation of **1** or **8** at 10 mg/kg dose. Solution formulation of **1** or **8** was prepared in 5% NMP, 5% solutol HS-15 in normal saline. The dosing volume administered was 10 mL/kg for the oral route. Blood (~60 µL) samples were collected from a set of three mice at each time point in a labeled micro centrifuge tube containing K2EDTA solution as anticoagulant at pre-dose, 0.5, 1, 2, 4, 6, 8, 12, and 24 h. Plasma samples were separated by centrifugation at 4000 rpm for 10 min and stored below −70 °C until bioanalysis. Concentrations of **1** or **8** in mice plasma samples were determined by a LC-MS/MS method. The plasma concentration-time data for **1** or **8** was provided for data analysis by the bioanalytical group (Sai Life Sciences Ltd., Pune, India). The plasma concentration-time data were then used for the pharmacokinetic analysis. A non-compartmental analysis module in Phoenix WinNonlin (Version 6.3, Certara USA Inc., Raleigh, NC, USA) was used to assess the pharmacokinetic parameters. Maximum concentration (C_max_) and time to reach the maximum concentration (T_max_) were the observed values. The areas under the concentration time curve (AUC_last_ and AUC_inf_) were calculated by linear trapezoidal rule.

#### 4.2.5. KinomeScan Assay

KinomeScan Assay on **35** was performed as previously described at EuroFins DiscoverX (Fremont, CA, USA) [7].

### 4.3. Chemistry Methods and Compound Characterization

#### 4.3.1. General Information

All reactions were performed using flame-dried round-bottomed flasks or reaction vessels. Where appropriate, reactions were carried out under an inert atmosphere of nitrogen with dry solvents, unless otherwise stated. Yields refer to chromatographically and spectroscopically pure isolated yields. Reagents were purchased at the highest commercial quality and used without further purification. Reactions were monitored by thin-layer chromatography carried out on 0.25-mm E. Merck silica gel plates (60_F-254_) using ultraviolet light as a visualizing agent. NMR spectra were recorded on a Varian Inova 400 (Varian, Palo Alto, CA, USA) or Inova 500 spectrometer (Varian, Palo Alto, CA, USA) and calibrated using residual undeuterated solvent as an internal reference (CDCl_3_: ^1^H NMR = 7.26, ^13^C NMR = 77.16). The following abbreviations or combinations thereof were used to explain the multiplicities observed: s = singlet, d = doublet, t = triplet, q = quartet, m = multiplet, br = broad. Liquid chromatography (LC) and high resolution mass spectra (HRMS) were recorded on a ThermoFisher hybrid LTQ FT (ICR 7T) (ThermoFisher, Waltham, MA USA). The University of Southampton (Southampton, UK) small molecule x-ray facility collected and analyzed all X-ray diffraction data.

Compounds **1**, **8**, and **13** were synthesized as previously reported [8,9,12]. Compound spectra of **9**–**12**, **14**–**43** are included in the Supplementary Materials. All compounds were >98% pure by ^1^H/^13^C NMR and LCMS.

#### 4.3.2. General Procedure for the Synthesis of 4-anilinoquinolines (Scheme 1)

6-Bromo-4-chloroquinoline (1.0 equiv.) and 3,4,5-trimethoxyaniline (1.1 equiv.) were suspended in ethanol (10 mL) and refluxed for 18 h. The crude mixture was purified by flash chromatography using EtOAc:hexane followed by 1–5% methanol in EtOAc, solvent was removed under reduced pressure to afford the desired product (**1**, **8**–**11**, **13**–**31**, and **33**–**43**).

#### 4.3.3. General Procedure for the Synthesis of 4-anilinoquinolines (Scheme 2)

4-Chloro-6-(trifluoromethyl)quinoline (1.0 equiv.), aniline (1.1 equiv.), *bis*(dibenzylideneacetone)palladium(0) (Pd(dba)_2_) (0.1 equiv.), 2-dicyclohexylphosphino-2′,4′,6′-triisopropylbiphenyl (XPhos) (0.15 equiv.), and cesium carbonate (2.0 equiv.) were all suspended in dimethylformamide 15 mL and degassed for 5 min. The mixture was heated to 140 °C for 18 h. The crude mixture was passed through a plug of Celite 545 before been purified by flash chromatography 20–100% ethyl acetate/hexane followed by 1–5% methanol/ethyl acetate and solvent removed under reduced pressure to afford the desired product (**12** and **32**).

#### 4.3.4. Compound Characterization

*N-(Benzo[d]*[1,3]*dioxol-4-yl)-6-bromoquinolin-4-amine* (**9**) was obtained as a yellow solid (157 mg, 0.458 mmol, 74%). m.p. > 270 °C decomp.; ^1^H NMR (400 MHz, DMSO-*d*_6_) δ 11.21 (s, 1H), 9.24 (d, *J* = 1.9 Hz, 1H), 8.57 (d, *J* = 6.9 Hz, 1H), 8.25–8.02 (m, 2H), 7.16–6.81 (m, 3H), 6.65 (d, *J* = 6.9 Hz, 1H), 6.11 (s, 2H). ^13^C NMR (101 MHz, DMSO-*d*_6_) δ 153.1, 148.8, 142.9, 141.5, 137.2, 136.5, 126.3, 122.6, 122.5, 120.0, 119.5, 119.1, 118.4, 107.9, 101.84, 101.80. HRMS *m*/*z* [M + H]^+^ calculated for C_16_H_12_N_2_O_2_Br: 343.0082, found 343.0072, LC t_R_ = 3.43 min, >98% purity.

*6-Bromo-N-(2,5-difluorophenyl)quinolin-4-amine* (**10**) was obtained as a colorless solid (125 mg, 0.373 mmol, 60%). m.p. 187–189 °C; ^1^H NMR (400 MHz, DMSO-*d*_6_) δ 11.11 (s, 1H), 9.21 (d, *J* = 2.0 Hz, 1H), 8.59 (d, *J* = 6.9 Hz, 1H), 8.20 (dd, *J* = 9.1, 1.9 Hz, 1H), 8.12 (d, *J* = 9.0 Hz, 1H), 7.78–7.46 (m, 2H), 7.38–7.25 (m, 1H), 6.56 (dd, *J* = 6.9, 2.3 Hz, 1H). 13C NMR (101 MHz, DMSO-*d*_6_) δ 161.5 (dd, *J* = 247.9, 11.7 Hz), 157.1 (dd, *J* = 251.6, 13.2 Hz), 154.5, 143.4, 137.3, 136.7, 130.2 (dd, *J* = 10.2, 2.0 Hz), 126.2, 122.7, 121.0 (dd, *J* = 12.6, 3.9 Hz), 120.2, 118.4, 113.0 (dd, *J* = 22.6, 3.7 Hz), 105.8 (dd, *J* = 27.1, 24.0 Hz), 100.9 (d, *J* = 1.8 Hz). HRMS *m*/*z* [M + H]^+^ calculated for C_15_H_10_N_2_F_2_Br: 334.9995, found 334.9985, LC t_R_ = 3.65 min, >98% purity.

*6-Bromo-N-(2,4-difluorophenyl)quinolin-4-amine* (**11**) was obtained as a colorless solid (94 mg, 0.281 mmol, 45%). m.p. 301–303 °C; 1H NMR (400 MHz, DMSO-*d*_6_) δ 11.18 (s, 1H), 9.20 (d, *J* = 2.0 Hz, 1H), 8.62 (d, *J* = 6.8 Hz, 1H), 8.20 (dd, *J* = 9.0, 2.0 Hz, 1H), 8.12 (d, *J* = 9.0 Hz, 1H), 7.65–7.48 (m, 2H), 7.40 (ddt, *J* = 9.2, 8.0, 3.5 Hz, 1H), 6.68 (dd, *J* = 6.8, 2.7 Hz, 1H). 13C NMR (101 MHz, DMSO-*d*_6_) δ 158.2 (dd, *J* = 242.3, 2.3 Hz), 153.2 (dd, *J* = 245.2, 2.9 Hz), 153.9, 143.6, 137.4, 136.7, 126.2, 125.6 (dd, *J* = 14.7, 11.0 Hz), 122.8, 120.3, 118.5, 118.4 (dd, *J* = 22.5, 9.7 Hz), 116.3 (dd, *J* = 23.9, 8.2 Hz), 115.5, 115.2, 101.5 (d, *J* = 2.3 Hz). HRMS *m*/*z* [M + H]^+^ calculated for C_15_H_10_N_2_F_2_Br: 334.9995, found 334.9985, LC t_R_ = 3.63 min, >98% purity.

*6-Bromo-N-(perfluorophenyl)quinolin-4-amine* (**12**) was obtained as a brown solid (51.4 mg, 0.136 mmol, 21%) m.p. > 300 °C; ^1^H NMR (400 MHz, Methanol-*d*_4_) δ 8.91 (d, *J* = 4.8 Hz, 1H), 8.56 (dp, *J* = 1.9, 0.9 Hz, 1H), 8.25 (dp, *J* = 8.8, 0.8 Hz, 1H), 8.05 (dd, *J* = 8.8, 2.1 Hz, 1H), 7.79 (d, *J* = 4.8 Hz, 1H). 13C NMR (100 MHz, Methanol-*d*_4_) δ 153.7, 150.9 (d, *J* = 1.3 Hz), 145.0, 140.7–140.3 (m, 1C), 139.1–138.8, 138.2–137.2, 136.7–136.5, 134.6–134.3, 131.9, 130.7(q, *J* = 32.9 Hz), 127.4 (q, *J* = 3.1 Hz), 127.0, 126.57, 124.1, 123.9, 123.2 (q, *J* = 4.6 Hz). HRMS *m*/*z* [M + H]^+^ calculated for C_16_H_7_N_2_F_8_: 379.0481, found 379.0473, LC t_R_ = 4.38 min, >98% purity.

*6-Bromo-N-(5-fluorobenzo[d][1,3]dioxol-4-yl)quinolin-4-amine* (**14**) was obtained as a beige solid (174 mg, 0.483 mmol, 78%). m.p. 224–227 °C; ^1^H NMR (400 MHz, DMSO-*d*_6_) δ 11.08 (s, 1H), 9.26 (d, *J* = 1.7 Hz, 1H), 8.63 (d, *J* = 6.8 Hz, 1H), 8.28–8.06 (m, 2H), 7.04 (dd, *J* = 8.7, 4.1 Hz, 1H), 6.94 (dd, *J* = 10.8, 8.6 Hz, 1H), 6.67 (dd, *J* = 6.8, 1.2 Hz, 1H), 6.17 (s, 2H). ^13^C NMR (100 MHz, DMSO-*d*_6_) δ 153.8, 152.4 (d, *J* = 240.7 Hz), 144.8 (d, *J* = 1.8 Hz), 143.9 (d, *J* = 6.3 Hz), 143.4, 137.3, 136.8, 126.1, 122.7, 120.4, 118.4, 108.7 (d, *J* = 20.5 Hz), 107.9 (d, *J* = 22.4 Hz), 107.5 (d, *J* = 9.2 Hz), 103.2, 101.7. HRMS *m*/*z* [M + H]^+^ calculated for C_16_H_10_N_2_O_2_FBr: 359.9910, found 360.9987, LC t_R_ = 3.53 min, >98% purity.

*6-Bromo-N-(2,3-dimethoxyphenyl)quinolin-4-amine* (**15**) was obtained as a colorless solid (187 mg, 0.520 mmol, 84%). m.p. 150–153 °C; ^1^H NMR (400 MHz, DMSO-*d*_6_) δ 11.03–10.85 (br s, 1H), 9.19 (d, *J* = 2.0 Hz, 1H), 8.50 (d, *J* = 6.9 Hz, 1H), 8.18 (dd, *J* = 9.0, 2.0 Hz, 1H), 8.08 (d, *J* = 9.0 Hz, 1H), 7.26 (dd, *J* = 8.4, 7.7 Hz, 1H), 7.20 (dd, *J* = 8.4, 1.7 Hz, 1H), 7.00 (dd, *J* = 7.7, 1.7 Hz, 1H), 6.39 (d, *J* = 6.9 Hz, 1H), 3.89 (s, 3H), 3.67 (s, 3H). ^13^C NMR (101 MHz, DMSO- *d*_6_) δ 155.1, 154.0, 144.8, 143.2, 137.7, 137.0, 130.5, 126.6, 125.2, 123.0, 120.3, 119.9, 118.6, 113.5, 101.6, 61.2, 56.5. HRMS *m*/*z* [M + H]^+^ calculated for C_17_H_16_N_2_O_2_Br: 359.0395, found 359.0284, LC t_R_ = 3.62 min, >98% purity.

*6-Bromo-N-(2,2-difluorobenzo[d][1,3]dioxol-4-yl)quinolin-4-amine* (**16**) was obtained as a colorless solid (190 mg, 0.501 mmol, 81%). m.p. 272–274 °C; ^1^H NMR (400 MHz, DMSO-*d*_6_) δ 11.36 (s, 1H), 9.22 (d, *J* = 2.0 Hz, 1H), 8.68 (d, *J* = 6.8 Hz, 1H), 8.21 (dd, *J* = 9.0, 2.0 Hz, 1H), 8.13 (d, *J* = 9.0 Hz, 1H), 7.50 (dd, *J* = 7.5, 1.7 Hz, 1H), 7.44–7.21 (m, 2H), 6.82 (d, *J* = 6.8 Hz, 1H). ^13^C NMR (100 MHz, DMSO-*d*_6_) δ 153.2, 144.1, 143.6, 137.4, 137.1, 136.8, 131.0 (t, *J* = 254.8 Hz), 126.3, 125.5, 122.7, 122.2, 120.4 (d, *J* = 3.0 Hz, 2C), 118.7, 109.2, 101.9. HRMS *m*/*z* [M + H]^+^ calculated for C_16_H_10_N_2_O_2_F_2_Br: 378.9894, found 378.9880, LC t_R_ = 4.13 min, >98% purity.

*6-Bromo-N-(2,3-dihydrobenzofuran-4-yl)quinolin-4-amine* (**17**) was obtained as a yellow solid (133 mg, 0.390 mmol, 64%). m.p. 291–293 °C; ^1^H NMR (400 MHz, DMSO-*d*_6_) δ 11.09 (s, 1H), 9.19 (d, *J* = 2.0 Hz, 1H), 8.52 (d, *J* = 6.9 Hz, 1H), 8.17 (dd, *J* = 9.0, 2.0 Hz, 1H), 8.08 (d, *J* = 9.0 Hz, 1H), 7.30 (t, *J* = 8.0 Hz, 1H), 6.89 (ddd, *J* = 10.4, 8.0, 0.8 Hz, 2H), 6.56 (d, *J* = 6.9 Hz, 1H), 4.59 (t, *J* = 8.7 Hz, 2H), 3.10 (t, *J* = 8.7 Hz, 2H). ^13^C NMR (100 MHz, DMSO-*d*_6_) δ 161.4, 153.6, 143.1, 137.3, 136.6, 133.7, 129.5, 126.3, 124.2, 122.5, 119.9, 118.5, 117.8, 108.6, 100.9, 71.2, 27.9. HRMS *m*/*z* [M + H]^+^ calculated for C_17_H_14_N_2_OBr: 341.0289, found 341.0283, LC t_R_ = 3.59 min, >98% purity.

*6-Bromo-N-(2,3-dihydrobenzofuran-7-yl)quinolin-4-amine* (**18**) was obtained as a yellow solid (173 mg, 0.507 mmol, 82%). m.p. 301–304 °C; ^1^H NMR (400 MHz, DMSO-*d*_6_) δ 11.08 (s, 1H), 9.25–9.17 (m, 1H), 8.52 (d, *J* = 6.9 Hz, 1H), 8.22–8.04 (m, 2H), 7.32 (dq, *J* = 7.3, 1.1 Hz, 1H), 7.23–7.15 (m, 1H), 6.99 (dd, *J* = 7.9, 7.3 Hz, 1H), 6.44 (d, *J* = 6.9 Hz, 1H), 4.59 (t, *J* = 8.7 Hz, 2H), 3.30 (t, *J* = 8.7 Hz, 2H). ^13^C NMR (101 MHz, DMSO-*d*_6_) δ 153.9, 153.5, 142.5, 137.1, 136.4, 129.8, 126.2, 125.5, 124.6, 122.4, 121.3, 119.8, 119.1, 118.2, 101.5, 71.9, 29.3. HRMS *m*/*z* [M + H]^+^ calculated for C_17_H_14_N_2_OBr: 341.0289, found 341.0285 LC t_R_ = 3.70 min, >98% purity.

*N-(Benzofuran-4-yl)-6-bromoquinolin-4-amine* (**19**) was obtained as a yellow solid (162 mg, 0.476 mmol, 77%). m.p. > 300 °C; ^1^H NMR (400 MHz, DMSO-*d*_6_) δ 11.36 (s, 1H), 9.27 (d, *J* = 2.0 Hz, 1H), 8.50 (d, *J* = 6.9 Hz, 1H), 8.20 (dd, *J* = 9.0, 2.0 Hz, 1H), 8.16–7.90 (m, 2H), 7.73 (dt, *J* = 8.3, 0.9 Hz, 1H), 7.51 (t, *J* = 8.0 Hz, 1H), 7.38 (dd, *J* = 7.7, 0.8 Hz, 1H), 6.91 (dd, *J* = 2.2, 1.0 Hz, 1H), 6.52 (d, *J* = 6.9 Hz, 1H). ^13^C NMR (100 MHz, DMSO- *d*_6_) δ 155.4, 154.0, 146.7, 143.0, 137.4, 136.5, 130.0, 129.7, 126.5, 125.3, 123.7, 122.5, 120.2, 119.9, 118.7, 111.1, 105.2, 101.0. HRMS *m*/*z* [M + H]^+^ calculated for C_17_H_12_N_2_OBr: 339.0133, found 339.0129, LC t_R_ = 3.82 min, >98% purity.

*N-(Benzofuran-7-yl)-6-bromoquinolin-4-amine* (**20**) was obtained as a yellow solid (157 mg, 0.464 mmol, 75%). m.p. 256–259 °C; ^1^H NMR (400 MHz, DMSO-*d*_6_) δ 11.37 (s, 1H), 9.27 (d, *J* = 2.0 Hz, 1H), 8.53 (d, *J* = 6.9 Hz, 1H), 8.21 (dd, *J* = 9.1, 2.0 Hz, 1H), 8.13 (d, *J* = 9.0 Hz, 1H), 8.07 (d, *J* = 2.2 Hz, 1H), 7.79 (dd, *J* = 6.0, 2.9 Hz, 1H), 7.55–7.26 (m, 2H), 7.13 (d, *J* = 2.1 Hz, 1H), 6.46 (d, *J* = 6.9 Hz, 1H). ^13^C NMR (100 MHz, DMSO-*d*_6_) δ 154.0, 148.3, 146.7, 143.1, 137.3, 136.7, 129.5, 126.2, 124.0, 122.6, 122.0, 121.3, 121.2, 120.4, 118.5, 107.5, 101.4. HRMS *m*/*z* [M + H]^+^ calculated for C_17_H_12_N_2_OBr: 339.0133, found 339.0129, LC t_R_ = 4.36 min, >98% purity.

*N-(6-Bromoquinolin-4-yl)benzo[d]isoxazol-7-amine* (**21**) was obtained as a colorless solid (141 mg, 0.414 mmol, 67%). m.p. > 300 °C; ^1^H NMR (400 MHz, DMSO-*d*_6_) δ 11.49 (s, 1H), 9.45 (s, 1H), 9.29 (d, *J* = 2.0 Hz, 1H), 8.45 (d, *J* = 6.9 Hz, 1H), 8.31 (dd, *J* = 6.8, 2.4 Hz, 1H), 8.21 (dd, *J* = 9.0, 2.0 Hz, 1H), 8.11 (d, *J* = 9.0 Hz, 1H), 7.75–7.62 (m, 2H), 6.41 (d, *J* = 6.9 Hz, 1H). ^13^C NMR (100 MHz, DMSO-*d*_6_) δ 157.6, 154.5, 148.3, 142.7, 137.3, 136.6, 135.8, 130.7, 126.4, 126.2, 123.9, 122.6, 122.5, 120.0, 118.4, 101.9. HRMS *m*/*z* [M + H]^+^ calculated for C_16_H_11_N_3_OBr: 340.0085, found 340.0074, LC t_R_ = 3.18 min, >98% purity.

*N-(6-Bromoquinolin-4-yl)benzo[d]isoxazol-4-amine* (**22**) was obtained as a dark green solid (122 mg, 0.359 mmol, 58%). m.p. > 300 °C; ^1^H NMR (400 MHz, DMSO-*d*_6_) δ 11.37 (s, 1H), 9.31 (s, 1H), 9.24 (d, *J* = 2.0 Hz, 1H), 8.58 (d, *J* = 6.8 Hz, 1H), 8.22 (dd, *J* = 9.0, 1.9 Hz, 1H), 8.12 (d, *J* = 9.0 Hz, 1H), 7.93–7.70 (m, 2H), 7.53 (dd, *J* = 4.6, 3.7 Hz, 1H), 6.85 (d, *J* = 6.8 Hz, 1H). ^13^C NMR (101 MHz, DMSO- *d*_6_) δ 162.7, 153.9, 145.7, 143.7, 137.6, 136.7, 131.8, 131.4, 126.5, 122.8, 120.2, 120.1, 119.2, 117.4, 108.6, 101.6. HRMS *m*/*z* [M + H]^+^ calculated for C_16_H_11_N_3_OBr: 340.0085, found 340.0084, LC t_R_ = 3.38 min, >98% purity.

*N-(6-Bromoquinolin-4-yl)benzo[d]oxazol-7-amine* (**23**) was obtained as a dark yellow solid (65 mg, 0.192 mmol, 31%). m.p. 305–308 °C; ^1^H NMR (400 MHz, DMSO-*d*_6_) δ 10.80 (s, 1H), 10.16 (s, 1H), 9.10 (d, *J* = 2.0 Hz, 1H), 8.58 (d, *J* = 6.9 Hz, 1H), 8.18 (dd, *J* = 9.0, 2.0 Hz, 1H), 8.03 (d, *J* = 9.0 Hz, 1H), 7.49 (d, *J* = 7.9 Hz, 1H), 7.21 (t, *J* = 7.9 Hz, 1H), 6.50 (d, *J* = 6.9 Hz, 1H). ^13^C NMR (176 MHz, DMSO-*d*_6_) δ 160.9, 154.9, 149.2, 143.9, 143.3, 138.2, 136.8, 128.5, 127.0, 126.9, 126.3, 123.5, 121.1, 120.0, 119.1, 101.5. HRMS *m*/*z* [M + H]^+^ calculated for C_16_H_11_N_3_OBr: 340.0085, found 340.0084, LC t_R_ = 3.67 min, >98% purity.

*N-(6-Bromoquinolin-4-yl)benzo[d]thiazol-7-amine* (**24**) was obtained as a dark yellow solid (84 mg, 0.235 mmol, 38%). m.p. 240–243 °C; ^1^H NMR (400 MHz, DMSO-*d*_6_) δ 9.54 (s, 1H), 9.39 (d, *J* = 0.4 Hz, 1H), 8.73 (t, *J* = 1.4 Hz, 1H), 8.44 (d, *J* = 5.2 Hz, 1H), 8.05 (dd, *J* = 8.2, 1.0 Hz, 1H), 7.96–7.75 (m, 2H), 7.66 (t, *J* = 7.9 Hz, 1H), 7.55–7.46 (m, 1H), 6.29 (d, *J* = 5.2 Hz, 1H). ^13^C NMR (100 MHz, DMSO-*d*_6_) δ 156.1, 154.9, 151.1, 147.5, 145.9, 134.7, 132.4, 131.5, 129.2, 127.3, 124.7, 121.8, 120.9, 120.4, 117.9, 103.6. HRMS *m*/*z* [M + H]^+^ calculated for C_16_H_11_N_3_F: 355.9857, found 355.9846, LC t_R_ = 3.20 min, >98% purity.

*N-(6-Bromoquinolin-4-yl)benzo[d]thiazol-4-amine* (**25**) was obtained as a colorless solid (150 mg, 0.421 mmol, 68%). m.p. 281–283 °C; ^1^H NMR (400 MHz, DMSO- *d*_6_) δ 11.46 (s, 1H), 9.45 (s, 1H), 9.27 (d, *J* = 2.0 Hz, 1H), 8.45 (d, *J* = 6.9 Hz, 1H), 8.37–8.25 (m, 1H), 8.21 (dd, *J* = 9.0, 2.0 Hz, 1H), 8.10 (d, *J* = 9.0 Hz, 1H), 7.75–7.59 (m, 2H), 6.42 (d, *J* = 6.9 Hz, 1H). ^13^C NMR (100 MHz, DMSO-*d*_6_) δ 157.6, 154.4, 148.3, 142.7, 137.4, 136.6, 135.8 130.7, 126.4, 126.2, 123.9, 122.7, 122.5, 120.0, 118.4, 101.9. HRMS *m*/*z* [M + H]^+^ calculated for C_16_H_11_N_3_SBr: 355.9857, found 355.9846, LC t_R_ = 3.30 min, >98% purity.

*N-(Benzo[b]thiophen-4-yl)-6-bromoquinolin-4-amine* (**26**) was obtained as a dark yellow solid (147 mg, 0.414 mmol, 67%). m.p. 308–311 °C; ^1^H NMR (400 MHz, DMSO-*d*_6_) δ 11.40 (s, 1H), 9.28 (d, *J* = 2.0 Hz, 1H), 8.46 (d, *J* = 6.9 Hz, 1H), 8.20 (dd, *J* = 9.0, 2.0 Hz, 1H), 8.16 (dt, *J* = 8.1, 0.9 Hz, 1H), 8.11 (d, *J* = 9.0 Hz, 1H), 7.88 (dd, *J* = 5.5, 0.4 Hz, 1H), 7.60–7.52 (m, 1H), 7.49 (dd, *J* = 7.5, 1.0 Hz, 1H), 7.37 (dd, *J* = 5.6, 0.8 Hz, 1H), 6.37 (d, *J* = 6.9 Hz, 1H). ^13^C NMR (100 MHz, DMSO-*d*_6_) δ 154.6, 143.0, 141.1, 137.4, 136.6, 135.8, 131.4, 129.1, 126.5, 125.3, 122.8, 122.5, 122.4, 121.3, 119.8, 118.6, 100.9. HRMS *m*/*z* [M + H]^+^ calculated for C_17_H_12_N_2_SBr: 354.9905, found 354.9900, LC t_R_ = 4.64 min, >98% purity.

*N-(1H-Benzo[d]imidazol-7-yl)-6-bromoquinolin-4-amine* (**27**) was obtained as a dark yellow solid (25 mg, 0.074 mmol, 12%). m.p. 270–273 °C; ^1^H NMR (400 MHz, DMSO-*d*_6_) δ 8.85 (d, *J* = 1.4 Hz, 1H), 8.37 (d, *J* = 5.3 Hz, 1H), 8.15 (s, 1H), 7.80 (d, *J* = 1.2 Hz, 2H), 7.49 (d, *J* = 7.9 Hz, 1H), 7.24 (t, *J* = 7.7 Hz, 1H), 7.18 (d, *J* = 7.5 Hz, 1H), 6.49 (dd, *J* = 5.3, 1.5 Hz, 1H). ^13^C NMR (100 MHz, DMSO-*d*_6_) δ 175.3, 151.0, 151.0, 147.9, 147.5, 141.9, 141.6, 139.8, 132.0, 131.3, 129.2, 125.1, 122.0, 121.0, 117.3, 117.0. HRMS *m*/*z* [M + H]^+^ calculated for C_16_H_12_N_4_Br: 339.0245, found 339.0232, LC t_R_ = 2.46 min, >98% purity.

*N-(6-Bromoquinolin-4-yl)benzo[c][1,2,5]oxadiazol-4-amine* (**28**) was obtained as a black solid (152 mg, 0.445 mmol, 72%). m.p. > 300 °C; ^1^H NMR (400 MHz, DMSO-*d*_6_) δ 11.65 (s, 1H), 9.29 (d, *J* = 2.0 Hz, 1H), 8.63 (d, *J* = 6.8 Hz, 1H), 8.23 (dd, *J* = 9.0, 1.9 Hz, 1H), 8.18–8.06 (m, 2H), 7.83–7.70 (m, 2H), 6.94 (d, *J* = 6.8 Hz, 1H). ^13^C NMR (101 MHz, DMSO-*d*_6_) δ 153.6, 150.3, 145.9, 143.3, 137.4, 136.8, 133.2, 128.0, 126.4, 125.7, 122.8, 120.5, 118.9, 115.3, 103.5. HRMS *m*/*z* [M + H]^+^ calculated for C_15_H_10_N_4_OBr: 341.0038, found 341.0027, LC t_R_ = 3.39 min, >98% purity.

*N-(6-Bromoquinolin-4-yl)benzo[c][1,2,5]thiadiazol-4-amine* (**29**) was obtained as a yellow solid (164 mg, 0.458 mmol, 74%). m.p. > 300 °C; ^1^H NMR (400 MHz, DMSO-*d*_6_) δ 11.54 (s, 1H), 9.28 (d, *J* = 2.0 Hz, 1H), 8.48 (d, *J* = 6.8 Hz, 1H), 8.19 (ddd, *J* = 8.5, 6.4, 1.6 Hz, 2H), 8.12 (d, *J* = 9.0 Hz, 1H), 7.95–7.78 (m, 2H), 6.61 (d, *J* = 6.8 Hz, 1H). ^13^C NMR (100 MHz, DMSO-*d*_6_) δ 155.6, 153.8, 149.9, 143.3, 137.9, 136.5, 130.3, 129.3, 126.2, 126.1, 123.2, 120.8, 120.1, 118.8, 103.0. HRMS *m*/*z* [M + H]^+^ calculated for C_15_H_10_N_4_SBr: 356.9810, found 356.9798, LC t_R_ = 3.34 min, >98% purity.

*6-Bromo-N-(1-methyl-1H-benzo[d][1,2,3]triazol-4-yl)quinolin-4-amine* (**30**) was obtained as a colorless solid (118 mg, 0.334 mmol, 54%). m.p. 202–205 °C; ^1^H NMR (400 MHz, DMSO-*d*_6_) δ 9.54 (s, 1H), 8.82 (dd, *J* = 1.9, 0.8 Hz, 1H), 8.47 (d, *J* = 5.2 Hz, 1H), 7.98–7.75 (m, 2H), 7.64 (dd, *J* = 8.3, 0.9 Hz, 1H), 7.57 (dd, *J* = 8.3, 7.3 Hz, 1H), 7.31 (dd, *J* = 7.3, 1.0 Hz, 1H), 6.73 (d, *J* = 5.3 Hz, 1H), 4.33 (s, 3H). ^13^C NMR (100 MHz, DMSO-*d*_6_) δ 151.0, 147.5, 146.9, 139.6, 135.0, 132.2, 131.7, 131.4, 128.0, 124.9, 121.4, 117.9, 116.2, 106.2, 104.9, 34.3. HRMS *m*/*z* [M + H]^+^ calculated for C_16_H_13_N_5_Br: 354.0354, found 354.0343, LC t_R_ = 3.04 min, >98% purity.

*6-Bromo-N-(1-methyl-1H-indazol-4-yl)quinolin-4-amine* (**31**) was obtained as a dark gray solid (131 mg, 0.371 mmol, 60%). m.p. 206–209 °C; ^1^H NMR (400 MHz, DMSO-*d*_6_) δ 11.38 (s, 1H), 9.29 (d, *J* = 2.0 Hz, 1H), 8.49 (d, *J* = 6.9 Hz, 1H), 8.19 (dd, *J* = 9.0, 2.0 Hz, 1H), 8.12 (d, *J* = 9.0 Hz, 1H), 8.00 (d, *J* = 0.9 Hz, 1H), 7.75 (dt, *J* = 8.5, 0.8 Hz, 1H), 7.56 (dd, *J* = 8.5, 7.3 Hz, 1H), 7.26 (dd, *J* = 7.3, 0.7 Hz, 1H), 6.59 (d, *J* = 6.9 Hz, 1H), 4.12 (s, 3H). ^13^C NMR (100 MHz, DMSO-*d*_6_) δ 154.1, 142.9, 141.0, 137.4, 136.5, 130.9, 129.3, 126.8, 126.5, 122.5, 119.9, 119.2, 118.8, 117.2, 109.6, 101.3, 35.7. HRMS *m*/*z* [M + H]^+^ calculated for C_17_H_14_N_4_Br: 353.0402, found 353.0340, LC t_R_ = 3.48 min, >98% purity.

*6-Bromo-N-(1H-indazol-4-yl)quinolin-4-amine* (**32**) was obtained as a bright yellow solid (174 mg, 0.513 mmol, 83%). m.p. > 300 °C; ^1^H NMR (400 MHz, DMSO-*d*_6_) δ 13.51 (s, 1H), 11.34 (s, 1H), 9.27 (d, *J* = 2.0 Hz, 1H), 8.50 (d, *J* = 6.9 Hz, 1H), 8.20 (dd, *J* = 9.0, 2.0 Hz, 1H), 8.11 (d, *J* = 9.0 Hz, 1H), 8.02 (d, *J* = 1.1 Hz, 1H), 7.65 (dt, *J* = 8.4, 0.9 Hz, 1H), 7.52 (dd, *J* = 8.4, 7.2 Hz, 1H), 7.23 (dd, *J* = 7.3, 0.7 Hz, 1H), 6.60 (d, *J* = 6.9 Hz, 1H). ^13^C NMR (100 MHz, DMSO-*d*_6_) δ 154.1, 143.0, 141.4, 137.4, 136.5, 131.9, 129.2, 126.8, 126.4, 122.5, 119.9, 118.8, 118.6, 117.1, 110.1, 101.2. HRMS *m*/*z* [M + H]^+^ calculated for C_16_H_12_N_4_Br: 339.0245, found 339.0234, LC t_R_ = 3.08 min, >98% purity.

*N-(1H-Indazol-4-yl)-6-(trifluoromethyl)quinolin-4-amine* (**33**) was obtained as a dark yellow solid (36 mg, 0.110 mmol, 17%). m.p. 106–109 °C; ^1^H NMR (700 MHz, DMSO-*d*_6_) δ 9.27 (d, *J* = 4.6 Hz, 1H), 8.89 (s, 1H), 8.82 (d, *J* = 2.0 Hz, 1H), 8.41 (d, *J* = 8.9 Hz, 1H), 8.15 (dd, *J* = 8.9, 2.1 Hz, 1H), 8.02 (d, *J* = 4.6 Hz, 1H), 6.99 (d, *J* = 8.3 Hz, 1H), 6.93 (dd, *J* = 8.3, 7.1 Hz, 1H), 6.40 (d, *J* = 7.0 Hz, 1H), 5.51 (s, 1H), 3.50 (s, 1H). ^13^C NMR (176 MHz, DMSO-*d*_6_) δ 154.0, 150.6, 144.4, 138.8, 131.7, 128.1 (q, *J* = 32.1 Hz), 126.7, 126.6, 126.1, 125.2, 123.6, 123.4, 121.7, 118.4, 107.4, 104.8, 40.3. HRMS *m*/*z* [M + H]^+^ calculated for C_17_H_12_N_4_F_3_: 329.1014, found 353.0340, LC t_R_ = 5.66 min, >98% purity.

*N-(1H-Indazol-7-yl)-6-(trifluoromethyl)quinolin-4-amine* (**34**) was obtained as a bright yellow solid (189 mg, 0.576 mmol, 89%). m.p. 251–254 °C; ^1^H NMR (400 MHz, DMSO-*d*_6_) δ 13.43 (s, 1H), 11.89–11.60 (m, 1H), 9.55–9.41 (m, 1H), 8.56 (d, *J* = 7.0 Hz, 1H), 8.47–8.21 (m, 2H), 8.15 (d, *J* = 1.0 Hz, 1H), 7.93 (dd, *J* = 8.5, 0.7 Hz, 1H), 7.70 (dt, *J* = 1.9, 0.9 Hz, 1H), 7.21 (dd, *J* = 8.5, 1.8 Hz, 1H), 6.89 (d, *J* = 7.0 Hz, 1H). ^13^C NMR (100 MHz, DMSO-*d*_6_) δ 156.2, 144.4, 140.6, 135.2, 134.1 (1C, br s), 129.7 (1C, d, *J* = 3.1 Hz), 128.3, 127.2 (q, *J* = 32.9 Hz), 125.6, 123.3 (d, *J* = 4.2 Hz), 122.8, 122.5, 122.3 (d, *J* = 5.9 Hz), 118.6, 117.2, 107.4 (1C, br s), 101.6. HRMS *m*/*z* [M + H]^+^ calculated for C_17_H_12_N_4_F_3_: 329.1014, found 329.1006, LC t_R_ = 3.42 min, >98% purity.

*6-Bromo-N-(1H-indazol-6-yl)quinolin-4-amine* (**35**) was obtained as an orange solid (180 mg, 0.532 mmol, 86%). m.p. > 300 °C; ^1^H NMR (400 MHz, DMSO-*d*_6_) δ 13.34 (s, 1H), 11.19 (s, 1H), 9.18 (d, *J* = 2.0 Hz, 1H), 8.51 (d, *J* = 6.9 Hz, 1H), 8.47–8.09 (m, 2H), 8.07 (d, *J* = 9.0 Hz, 1H), 7.95 (dd, *J* = 8.5, 0.7 Hz, 1H), 7.66 (dt, *J* = 1.9, 0.9 Hz, 1H), 7.20 (dd, *J* = 8.5, 1.8 Hz, 1H), 6.88 (d, *J* = 6.9 Hz, 1H). ^13^C NMR (100 MHz, DMSO-*d*_6_) δ 154.3, 143.1, 140.2, 137.4, 136.5, 135.0, 133.8, 126.2, 122.6, 122.1, 121.9, 119.8, 118.7, 118.2, 106.7, 100.6. HRMS *m*/*z* [M + H]^+^ calculated for C_16_H_12_N_4_Br: 339.0245, found 339.0243, LC t_R_ = 3.33 min, >98% purity.

*6-Bromo-N-(5-fluoro-1H-indazol-6-yl)quinolin-4-amine* (**36**) was obtained as a beige solid (188 mg, 0.526 mmol, 85%). m.p. > 300 °C; ^1^H NMR (400 MHz, DMSO-*d*_6_) δ 13.51 (s, 1H), 11.24 (s, 1H), 9.23 (d, *J* = 2.0 Hz, 1H), 8.56 (d, *J* = 6.9 Hz, 1H), 8.49–8.13 (m, 2H), 8.11 (d, *J* = 9.0 Hz, 1H), 7.88 (d, *J* = 10.3 Hz, 1H), 7.80 (dd, *J* = 6.3, 1.1 Hz, 1H), 6.61 (dd, *J* = 6.9, 2.5 Hz, 1H). ^13^C NMR (100 MHz, DMSO-*d*_6_) δ 154.8, 151.9 (d, *J* = 240.3 Hz), 143.3, 137.3, 136.7, 136.5, 134.4–133.0 (m, 1C), 126.2, 123.9 (d, *J* = 17.7 Hz), 122.6, 121.6 (d, *J* = 9.5 Hz), 120.1, 118.4, 109.8, 106.5 (d, *J* = 22.1 Hz), 101.2. HRMS *m*/*z* [M + H]^+^ calculated for C_16_H_11_N_4_FBr: 357.0151, found 357.0150, LC t_R_ = 3.43 min, >98% purity.

*6-Bromo-N-(1H-indazol-5-yl)quinolin-4-amine* (**37**) was obtained as a mustard solid (172 mg, 0.507 mmol, 82%). m.p. 260–263 °C; ^1^H NMR (400 MHz, Methanol-*d*_4_) δ 8.89 (d, *J* = 2.0 Hz, 1H), 8.35 (d, *J* = 7.0 Hz, 1H), 8.28–8.00 (m, 2H), 8.00–7.81 (m, 2H), 7.76 (dt, *J* = 8.8, 0.9 Hz, 1H), 7.46 (dd, *J* = 8.8, 1.9 Hz, 1H), 6.82 (d, *J* = 7.0 Hz, 1H). ^13^C NMR (100 MHz, Methanol-*d*_4_) δ 157.2, 143.8, 140.8, 138.8, 138.3, 135.4, 131.1, 126.9, 126.1, 124.9, 123.3, 122.0, 120.0, 119.3, 113.1, 101.6. HRMS *m*/*z* [M + H]^+^ calculated for C_16_H_12_N_4_Br: 339.0245, found 339.0243, LC t_R_ = 3.18 min, >98% purity.

*6-[(6-Bromoquinolin-4-yl)amino]-2,3-dihydro-1H-isoindol-1-one* (**38**) was obtained as a yellow solid (160 mg, 0.452 mmol, 73%). m.p. > 300 °C; ^1^H NMR (400 MHz, DMSO-*d*_6_) δ 11.19 (s, 1H), 9.18 (d, *J* = 2.0 Hz, 1H), 8.77 (s, 1H), 8.54 (d, *J* = 6.9 Hz, 1H), 8.16 (dd, *J* = 9.0, 2.0 Hz, 1H), 8.07 (d, *J* = 9.0 Hz, 1H), 7.95–7.72 (m, 2H), 7.70 (dd, *J* = 8.0, 2.0 Hz, 1H), 6.85 (d, *J* = 6.9 Hz, 1H), 4.45 (s, 2H). ^13^C NMR (100 MHz, DMSO-*d*_6_) δ 169.0, 154.0, 143.3, 142.9, 137.5, 136.9, 136.5, 134.3, 128.4, 126.2, 125.4, 122.7, 119.9, 119.4, 118.8, 100.5, 45.0. HRMS *m*/*z* [M + H]^+^ calculated for C_17_H_13_N_3_OBr: 354.0242, found 354.0240, LC t_R_ = 3.46 min, >98% purity.

*5-[(6-Bromoquinolin-4-yl)amino]-2,3-dihydro-1H-1,3-benzodiazol-2-one* (**39**) was obtained as a yellow solid (180 mg, 0.507 mmol, 82%). m.p. > 300 °C; ^1^H NMR (400 MHz, DMSO-*d*_6_) δ 10.88 (p, *J* = 2.4 Hz, 3H), 9.07 (d, *J* = 2.1 Hz, 1H), 8.46 (d, *J* = 7.0 Hz, 1H), 8.15 (dd, *J* = 9.0, 2.0 Hz, 1H), 8.00 (d, *J* = 9.0 Hz, 1H), 7.08 (d, *J* = 8.7 Hz, 1H), 7.04–6.96 (m, 2H), 6.73 (d, *J* = 7.0 Hz, 1H). ^13^C NMR (100 MHz, DMSO-*d*_6_) δ 155.4, 154.5, 143.0, 137.5, 136.4, 130. 7, 129.7, 129.1, 126.0, 122.7, 119.6, 118.4, 117.9, 109.2, 106.1, 100.3. HRMS *m*/*z* [M + H]^+^ calculated for C_16_H_12_N_4_OBr: 355.0194, found 355.0195, LC t_R_ = 3.43 min, >98% purity.

*6-[(6-Bromoquinolin-4-yl)amino]-2,3-dihydro-1,3-benzoxazol-2-one* (**40**) was obtained as a yellow solid (165 mg, 0.464 mmol, 75%). m.p. > 300 °C; ^1^H NMR (400 MHz, Methanol-*d*_4_) δ 8.84 (d, *J* = 1.9 Hz, 1H), 8.40 (d, *J* = 6.9 Hz, 1H), 8.14 (dd, *J* = 9.0, 2.0 Hz, 1H), 7.87 (d, *J* = 9.0 Hz, 1H), 7.40 (dd, *J* = 1.7, 0.8 Hz, 1H), 7.35–7.18 (m, 2H), 6.89 (d, *J* = 6.9 Hz, 1H). ^13^C NMR (100 MHz, Methanol-*d*_4_) δ 156.9, 156.5, 146.1, 144.6, 138.1, 132.8, 131.6, 126.8, 123.9, 123.8, 122.9, 122.0, 120.2, 111.7, 109.0, 101.9. HRMS *m*/*z* [M + H]^+^ calculated for C_16_H_11_N_3_O_2_Br: 356.0035, found 356.0032, LC t_R_ = 3.66 min, >98% purity.

*5-[(6-Bromoquinolin-4-yl)amino]-2,3-dihydro-1,3-benzoxazol-2-one* (**41**) was obtained as a yellow solid (163 mg, 0.458 mmol, 74%). m.p. > 300 °C; ^1^H NMR (400 MHz, DMSO-*d*_6_) δ 12.01 (s, 1H), 11.07 (s, 1H), 9.14 (d, *J* = 2.0 Hz, 1H), 8.51 (d, *J* = 6.9 Hz, 1H), 8.16 (dd, *J* = 9.0, 2.0 Hz, 1H), 8.05 (d, *J* = 9.0 Hz, 1H), 7.46 (d, *J* = 8.4 Hz, 1H), 7.30–6.99 (m, 2H), 6.79 (d, *J* = 6.9 Hz, 1H). ^13^C NMR (100 MHz, DMSO-*d*_6_) δ 154.5, 154.4, 143.1, 142.3, 137.4, 136.5, 132.8, 131.6, 126.2, 122.5, 119.8, 119.2, 118.5, 110.5, 107.5, 100.5. HRMS *m*/*z* [M + H]^+^ calculated for C_16_H_11_N_3_O_2_Br: 356.0035, found 356.0032, LC t_R_ = 3.71 min, >98% purity.

*5-[(6-Bromoquinolin-4-yl)amino]-1-methyl-2,3-dihydro-1H-1,3-benzodiazol-2-one* (**42**) was obtained as a yellow solid (162 mg, 0.439 mmol, 71%). m.p. > 300 °C; ^1^H NMR (400 MHz, DMSO-*d*_6_) δ 11.16 (s, 1H), 11.10 (s, 1H), 9.17 (d, *J* = 2.0 Hz, 1H), 8.46 (d, *J* = 7.0 Hz, 1H), 8.13 (dd, *J* = 9.0, 1.9 Hz, 1H), 8.05 (d, *J* = 9.0 Hz, 1H), 7.23 (d, *J* = 8.2 Hz, 1H), 7.17–6.98 (m, 2H), 6.71 (d, *J* = 7.0 Hz, 1H), 3.33 (s, 3H). ^13^C NMR (100 MHz, DMSO-*d*_6_) δ 154.6, 154.5, 142.7, 137.3, 136.4, 130.3, 130.1, 129.1, 126.2, 122.4, 119.6, 118.4, 118.0, 108.3, 106.3, 100.2, 26.6. HRMS *m*/*z* [M + H]^+^ calculated for C_17_H_14_N_4_OBr: 369.0351, found 369.0350 LC t_R_ = 3.70 min, >98% purity.

*5-((6-Bromoquinolin-4-yl)amino)-1,3-dihydrobenzo[c]thiophene 2,2-dioxide* (**43**) was obtained as an orange solid (205 mg, 0.526 mmol, 85%). m.p. > 300 °C; ^1^H NMR (400 MHz, DMSO-*d*_6_) δ 11.19 (s, 1H), 9.19 (d, *J* = 2.0 Hz, 1H), 8.55 (d, *J* = 6.9 Hz, 1H), 8.17 (dd, *J* = 9.0, 1.9 Hz, 1H), 8.09 (d, *J* = 9.0 Hz, 1H), 7.58 (d, *J* = 8.2 Hz, 1H), 7.54–7.36 (m, 2H), 6.88 (d, *J* = 6.9 Hz, 1H), 4.59 (d, *J* = 7.1 Hz, 4H). ^13^C NMR (100 MHz, DMSO-*d*_6_) δ 153.9, 143.2, 137.4, 136.9 136.6, 134.1, 131.5, 127.5, 126.3, 125.0, 122.5, 120.0, 118.7, 100.5, 56.1, 55.8. HRMS *m*/*z* [M + H]^+^ calculated for C_17_H_14_N_2_O_2_SBr: 388.9959, found 388.9955, LC t_R_ = 3.65 min, >98% purity.

#### 4.3.5. Mass Spectrometry Method

Samples were analyzed with a ThermoFisher Q Exactive HF-X (ThermoFisher, Bremen, Germany) mass spectrometer coupled with a Waters Acquity H-class liquid chromatograph system (Waters Corporation, Milford, MA, USA). Samples were introduced via a heated electrospray source (HESI) at a flow rate of 0.6 mL/min. Electrospray source conditions were set as: spray voltage 3.0 kV, sheath gas (nitrogen) 60 arb, auxiliary gas (nitrogen) 20 arb, sweep gas (nitrogen) 0 arb, nebulizer temperature 375 °C, capillary temperature 380 °C, RF funnel 45 V. The mass range was set to 150–2000 *m*/*z*. All measurements were recorded at a resolution setting of 120,000.

Separations were conducted on a Waters Acquity UPLC BEH C18 column (2.1 × 50 mM, 1.7 μM particle size). LC conditions were set at 100% water with 0.1% formic acid (A) ramped linearly over 9.8 min to 95% acetonitrile with 0.1% formic acid (B) and held until 10.2 min. At 10.21 min the gradient was switched back to 100% A and allowed to re-equilibrate until 11.25 min. Injection volume for all samples was 3 μL.

Xcalibur (ThermoFisher) was used to analyze the data. Solutions were analyzed at 0.1 mg/mL or less based on responsiveness to the ESI mechanism. Molecular formula assignments were determined with Molecular Formula Calculator. All observed species were singly charged, as verified by unit *m*/*z* separation between mass spectral peaks corresponding to the ^12^C and ^13^C^12^C_c-1_ isotope for each elemental composition.

### 4.4. Abbreviations Used

cyclin G-associated kinase (GAK)
mouse liver microsomes (MLM)
nicotinamide adenine dinucleotide phosphate (NADPH)
liquid chromatography mass spectra (LCMS)
mitogen-activated protein kinase kinase 5 (MEK5/MAP2K5)
transforming growth factor beta receptor 2 (TGFBR2)
receptor-interacting serine/threonine-protein kinase 2 (RIPK2)
1-aminobenzotriazole(ABT)
milligrams per kilogram (MPK)
*N*-methyl-2-pyrrolidoneolidone (NMP)
*Bis*(dibenzylideneacetone)palladium(0) (Pd(dba)_2_)
2-Dicyclohexylphosphino-2′,4′,6′-triisopropylbiphenyl (XPhos)

## 5. Conclusions

We found that optimization of a series of 4-anilinoquinolines GAK inhibitors to decrease liver metabolism was not compatible with maintaining potent enzyme activity. Nonetheless, several heterocyclic replacements of the trimethoxyaniline moiety were identified that had greatly improved metabolic stability and may be useful as bioisosteres in other lead optimization projects. Co-dosing with the P450 inhibitor ABT proved to be a more general approach to blocking the metabolism of several 4-anilinoquinolines GAK inhibitors in MLM and in vivo. The availability of additional non-toxic broad spectrum P450 inhibitors could further broaden the implementation of this approach to other chemical probes.

## Figures and Tables

**Table 1:** Initial microsomal stability assessment (see Supplementary Materials for details).

Cmpd	R	X	T_1/2_ (min) ^a^	T_1/2_ Ratio ^b^	Primary Metabolism Site	Primary Metabolite Mass
**8**	3,4,5-(OMe)_3_	6,7-(OMe)_2_	12.2	1.6	Aniline	Parent − CH2
**9**	2,3-O-CH_2_-O-	6-Br	8.9	1.9	Aniline	Parent + O
**10**	2,5-F	6-Br	1.7	0.17	Aniline	Parent + O
**11**	2,4-F	6-Br	3.2	0.47	Aniline	Parent − F + OH
**12**	2,3,4,5,6-F_5_	6-CF_3_	45.6	7.0	nt	nt
**13**	3,4,5-(OMe)_3_	6-CF_3_	6.4	0.78	Aniline	Parent + O

nt: not tested; ^a^ mean average, n = 2; ^b^ T_1/2_ ratio is the normalization with respect to internal control (propranolol).

**Table 2:** GAK affinity measurements and microsomal clearance of initial quinoline series.

Cmpd	R	X	Biochemical ^a^ GAK *K*_i_ (nM)	Live Cell ^b^ GAK IC_50_ (nM) ^c^
**8**	3,4,5-(OMe)_3_	6,7-(OMe)_2_	0.54 ^d^	25 ^d^
**9**	2,3-O-CH_2_-O-	6-Br	1.9	22
**10**	2,5-F	6-Br	15	870
**11**	2,4-F	6-Br	35	2700
**12**	2,3,4,5,6-F_5_	6-CF_3_	880	nt
**13**	3,4,5-(OMe)_3_	6-CF_3_	3.9 ^d^	180 ^d^

nt: not tested; ^a^ TR-FRET assay; ^b^ nanoBRET; ^c^ mean average, n = 2; ^d^ reference [12].

**Table 3:**
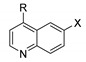
GAK affinity measurements and microsomal clearance of isosteric replacements of **9**.

Cmpd ^a^	X	Biochemical ^b^ GAK *K*_i_ (nM)	Live Cell ^c^ GAK IC_50_ (nM) ^d^	T_1/2_ (Min) ^d^	T_1/2_ Ratio ^e^
**9**	Br	1.9	22	8.9	1.9
**14**	Br	1.4	120	1.8	0.27
**15**	Br	740	>5000	1.0	0.12
**16**	Br	460	>5000	2.0	0.24
**17**	Br	5.2	>5000	1.7	0.16
**18**	Br	29	2500	0.82	0.10
**19**	Br	1.9	180	1.7	0.16
**20**	Br	4.3	570	0.9	0.08
**21**	Br	37	1400	5.2	0.64
**22**	Br	19	>5000	1.4	0.17
**23**	Br	19	340	1.7	0.21
**24**	Br	11	220	5.3	0.68
**25**	Br	26	>5000	2.6	0.32
**26**	Br	23	3300	4.1	0.50
**27**	Br	4.1	>5000	8.1	1.0
**28**	Br	41	2500	1.2	0.15
**29**	Br	5.3	400	1.6	0.20
**30**	Br	2.8	>5000	0.73	0.09
**31**	Br	3.6	>5000	0.80	0.10
**32**	Br	14	760	2.5	0.32
**33**	CF_3_	720	>5000	5.9	0.90
**34**	CF_3_	34	2600	46.4	9.8
**35**	Br	20	1400	45.6	9.8
**36**	Br	nt	>5000	14.8	2.2
**37**	Br	47	>5000	7.5	0.92
**38**	Br	62	>5000	8.1	1.0
**39**	Br	360	>5000	>200	>25
**40**	Br	680	>5000	55.9	6.9
**41**	Br	110	>5000	27.8	3.4
**42**	Br	1600	>5000	33.6	4.2
**43**	Br	1700	>5000	15.6	1.9

nt: not tested; ^a^ R groups are shown in Figure 4; ^b^ TR-FRET assay; ^c^ nanoBRET; ^d^ mean average, n = 2; ^e^ T_1/2_ ratio is the normalization with respect to internal control (propranolol).

**Table 4:** Pharmacokinetic profile of **1** and **8** with and without pre-administration of ABT.

Cmpd	T_1/2_ (min) ^a^	T_1/2_ Ratio	Cl_int_ (mL/min/kg)	T_1/2_ (min) ^a^	T_1/2_ Ratio ^b^	Cl_int_ (mL/min/kg)
	(−) ABT			(+) ABT		
**1**	5.7	0.58	990	130	13.2	40
**8**	18	1.3	320	>200	14.7	<28
**9**	0.74	0.05	7610	1.8	0.13	3140
**14**	0.74	0.05	7550	2.5	0.18	2290
**35**	46	9.8	120	170	17.5	35

^a^ mean average, n = 2; ^b^ T_1/2_ ratio following normalization with respect to an internal control (propranolol).

**Table 5:** Pharmacokinetic profile of **1** and **8** with and without pre-administration of ABT.

Experiment	T_max_ (h)	C_max_ (ng/mL)	AUC_inf_ (h·ng/mL)
10 mpk 1	0.5	97	98
2 h pretreat 50 mpk ABT 10 mpk 1	0.5	650	1900
10 mpk 8	1.0	19	71
2 h pretreat 50 mpk ABT 10 mpk 8	2.0	107	569

## Supplementary Materials

The following are available online at ‘https://www.mdpi.com/1420-3049/24/22/4016/s1, HRMS, ^1^H, and ^13^C NMR spectra of all new compounds. Metabolite identification profiling and Kinomescan data on compound **35**.
