# Supplementary material for: Towards the Development of an In vivo Chemical Probe for Cyclin G Associated Kinase (GAK)
Source: Molecules. 2019 Nov 6;24(22):4016. doi: 10.3390/molecules24224016 (PMC6891286; doi:10.3390/molecules24224016)
Supplement: Supplementary file 1 [file molecules-24-04016-s001.pdf]

Supplementary Materials

Towards the development of an *in vivo* chemical probe for cyclin G associated kinase (GAK)

Christopher R. M. Asquith [1,2,*], James M. Bennett [3], Lianyong Su [4], Tuomo Laitinen [5], Jonathan M. Elkins [3,6], Julie E. Pickett [1], Carrow I. Wells [1], Zengbiao Li [4], Timothy M. Willson [1], William J. Zuercher [1,7,*]

[1] Structural Genomics Consortium, UNC Eshelman School of Pharmacy, University of North Carolina at Chapel Hill, Chapel Hill, NC 27599, USA. (C.A), (J. E. P.), (C. I. W.), (T.M.W.), (W.J.Z.)
[2] Department of Pharmacology, University of North Carolina at Chapel Hill, Chapel Hill, NC 27599, USA (C.A.)
[3] Structural Genomics Consortium and Target Discovery Institute, Nuffield Department of Clinical Medicine, University of Oxford, Oxford, OX3 7DQ, UK. (J. M. B.), (J. M. E.)
[4] Drumetix Laboratories, Greensboro, NC 27409, USA. (L. S.), (Z. L.)
[5] School of Pharmacy, Faculty of Health Sciences, University of Eastern Finland, 70211, Kuopio, Finland. (T.L.)
[6] Structural Genomics Consortium, Universidade Estadual de Campinas - UNICAMP, Campinas, São Paulo, 13083-886, Brazil. (J.M.E.)
[7] Lineberger Comprehensive Cancer Center, University of North Carolina at Chapel Hill, Chapel Hill, NC 27599, USA. (W.J.Z.)

1. Metabolite Profiling
1.1. General Numbering of Metabolites
1.2. General Strategy for Metabolite Identification
2. Metabolite Profiling of 1
2.1. Product Ion Spectrum of 1 and Assignment of Fragments
2.2. Identification of Metabolites in Mouse Liver Microsomal Incubation
2.2.1. Identification of M1 at 4.14 min.
2.2.2. Identification of M2 at 4.46 min and M3 at 4.67 min.
2.2.3. Identification of M4 and M5 at 4.76 min and 6.78 min, respectively.
2.2.4. Identification of M6 at 7.66 min.
2.2.5. Peak Areas of Metabolites and 1
2.3. Metabolite Profiling Graphs and Tables for 1
3. Metabolite Profiling of 8
3.1. Product Ion Spectrum of 8 and Assignment of Fragments
3.2. Identification of Metabolites in Mouse Liver Microsomal Incubation
3.2.1. Identification of M1 at 3.38 min.
3.2.2. Identification of M2 at 3.68 min and M3 at 3.93 min.
3.2.3. Identification of M4, M5, M6, and M7 at 4.21, 4.31, 4.40, and 4.50 min, Respectively
3.3. Peak Areas of Metabolites and 8
3.4. Metabolite Profiling Graphs and Tables for 8
4. Metabolite Profiling of 9
4.1. Metabolite ID of 9 in Mouse Liver Microsomal Incubation
4.2. Metabolite Profiling Graphs and Tables for 9
5. Metabolite Profiling of 10
5.1. Product Ion Spectrum of 10 and Assignment of 10 Fragments
5.2. Identification of Metabolites in Mouse Liver Microsomal Incubation
5.2.1. Identification of M1 at 4.72 min, M2 at 8.47 min, and M3 at 8.70 min.
5.2.2. Peak Areas of Metabolites and 10
6. Metabolite Profiling of 11
6.1. Product Ion Spectrum of 11 and Assignment of 11 Fragments
6.2. Identification of Metabolites in Mouse Liver Microsomal Incubation
6.2.1. Identification of M1 at 4.68 min.
6.2.2. Identification of M2 at 4.77 min, M3 at 5.06 min, M4 at 7.49 min, and M5 at 7.90 min.
6.3. Peak Areas of Metabolites and 11
7. Metabolite Profiling of 35
7.1. Product Ion Spectrum of 35 and Assignment of 11 Fragments
7.2. Identification of Metabolites in Mouse Liver Microsomal Incubation
7.2.1. Identification of M1 at 3.69 min, M2 at 4.50 min, and M3 at 5.76 min.
7.3. Peak Areas of Metabolites and 35
7.4. Metabolite Profiling Graphs and Tables for 35
8. NAK family FRET screening and Kinetic Solubility for 1, 8-43
9. LabBook Codes and SMILES for 1, 8-43
10. Characterisation of compounds 9-43
11. KINOMEscan® results of 35

1. Metabolite Profiling

1.1. General Numbering of Metabolites

The metabolites are numbered according to their retention times in the gradient LC-MS analysis in positive ion mode. The metabolite with the lowest retention time is labeled as M1. The M number increases consecutively for metabolites with increasing retention time. Retention of a metabolite shifted slightly over time, especially for injections on different days. To avoid confusion, labelled retention times of a metabolite peak in different injections are the same, which may lead to mismatch between labelled retention time and actual retention time.

1.2. General Strategy for Metabolite Identification

The vehicle control and study samples were injected into LC-MS with gradient elution using a Q1 scan method. Searching for potential metabolites was then performed using LightSight software (Applied Biosystems/MDS Sciex) by comparing a total ion current chromatogram of Q1 scan from a study sample with a chromatogram from the corresponding vehicle control sample. The study sample was then injected again to obtain the product ion spectra of potential metabolites using product ion scan methods. The fragmentation patterns of the potential metabolites were then compared to the fragmentation of the parent compound to elucidate the structures of the metabolites.

2. Metabolite Profiling of 1

2.1. Product Ion Spectrum of 1 and Assignment of Fragments

The total ion current chromatogram from product ion scan of ion 389.2 (m/z) from 5 µL injection of 10 µM of **1** incubated in mouse liver microsomes for 5 minutes is shown in **Figure S1**. The product ion spectrum derived from the peak in **Figure S1** is shown in **Figure S2**. The assignment of fragments is shown in **Figure S3**.

2.2. Identification of Metabolites in Mouse Liver Microsomal Incubation

2.2.1. Identification of M1 at 4.14 min.

The component showed a quasi-molecular ion (MH) at m/z 361, a -28 dalton change relative to **1**. Extract ion (m/z 361) chromatograms, derived from the total ion chromatograms with Q1 scan from vehicle control sample Vehicle-MS-1 and study sample **1**-10 µM-MS-1, are shown in **Figure S4**. There was a peak at 4.14 min. in the chromatogram for the study sample. This peak was not observed in the chromatogram for the vehicle control sample. Please note that peaks at other times in the chromatogram for the study sample in **Figure S4** were from fragments of other metabolites, instead of metabolites with a quasi-molecular ion of 361 (m/z).

The -28 dalton corresponds to the mass change for the loss of two methylene units from **1**. The product ion spectrum for M1 is shown in **Figure S5**. Product ion 207 (m/z) was abundant in the product ion spectrum for M1. A structure consistent with the product ion spectrum was proposed for M1 as shown in **Table S1**.

2.2.2. Identification of M2 at 4.46 min and M3 at 4.67 min.

The components showed a quasi-molecular ion (MH) at m/z 375, a -14 dalton change relative to **1**. Extract ion (m/z 375) chromatograms, derived from the total ion chromatograms with Q1 scan from vehicle control sample Vehicle-MS-1 and study sample **1**-10µM-MS-1, are shown in **Figure S6**. There were peaks at 4.46 min. and 4.67 min., respectively, in the chromatogram for the study sample. These peaks were not observed in the chromatogram for the vehicle control sample. The -14 dalton corresponds to the mass change for the loss of one methylene unit from **1**.

The product ion spectrum for M2 is shown in **Figure S7**. Product ion 207 (m/z) was abundant in the product ion spectrum for M2. Product ion 373 (m/z) was not observed in the product ion spectrum for M2. Instead, product ion 359 (m/z) was observed. A structure consistent with the product ion spectrum was proposed for M2 as shown in **Table S1**.

The product ion spectrum for M3 is shown in **Figure S8**. Product ion 207 (m/z) was abundant in the product ion spectrum for M3. Product ion 373 (m/z) was not observed in the product ion spectrum for M3. Instead, product ion 359 (m/z) was observed. A structure consistent with the product ion spectrum was proposed for M3 as shown in Table 1.

2.2.3. Identification of M4 and M5 at 4.76 min and 6.78 min, respectively.

The components showed a quasi-molecular ion (MH) at m/z 405, a +16 dalton change relative to **1**. Extract ion (m/z 405) chromatograms, derived from the total ion chromatograms with Q1 scan from vehicle control sample Vehicle-MS-1 and study sample **1**-10µM-MS-1, are shown in **Figure S9**. There were peaks at 4.76 min and 6.78 min., respectively, in the chromatogram for the study sample. These peaks were not observed in the chromatogram for the vehicle control sample. The +16 dalton corresponds to the mass change for the oxidation of **1**.

The product ion spectrum for M4 is shown in **Figure S10**. No product ion 207 (m/z) signal was observed in the product ion spectrum for M4, indicating that oxidation most likely occurred in the quinoline ring structure. A structure consistent with the product ion spectrum was proposed for M4 as shown in **Table S1**.

The product ion spectrum for M5 is shown in **Figure S11**. Ion 207 (m/z) signal was much weaker in the product ion spectrum for M5 than that for **1**, indicating that oxidation most likely occurred in the quinoline ring structure. A structure consistent with the product ion spectrum was proposed for M5 as shown in **Table S1**.

2.2.4. Identification of M6 at 7.66 min.

The components showed a quasi-molecular ion (MH) at m/z 373, a -16 dalton change relative to **1**. Extract ion (m/z 373) chromatograms, derived from the total ion chromatograms with Q1 scan from vehicle control sample Vehicle-MS-1 and study sample **1**-10µM-MS-1, are shown in **Figure S12**. There was a peak at 7.66 min in the chromatogram for the study sample. This peak was not observed in the chromatogram for the vehicle control sample. The -16 dalton corresponds to the mass change for the loss of one methane molecule from **1**.

The product ion spectrum for M6 is shown in **Figure S13**. Product ion 207 (m/z) was abundant in the product ion spectrum for M6, indicating the neutral loss of methane occurred in the parts of CA93.0 other than the quinoline ring structure. A structure consistent with the product ion spectrum was proposed for M6 as shown in **Table S1**.

2.2.5. Peak Areas of Metabolites and 1

The peak areas of metabolites and **1** are listed in **Table S2**. The metabolite with the most intensive signal is M2, from the loss of one methylene unit from **1**. The major metabolism sites for **1** are methoxy groups. The metabolite with the most intensive mass spectrometric signal was formed through O-demethylation.

2.3. Metabolite Profiling Graphs and Tables for 1

Table S1. Metabolites of **1** (CA93.0) Observed in Mouse Liver Microsomal Incubation

Metabolite	Retention (min)	Mass to Charge Ratio (m/z) in Postive Ion Mode	Presence of Product Ion (Yes/No)		Proposed Structure and Fragment of Metabolite
			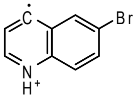 Exact Mass: 206.97	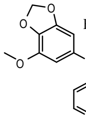 Exact Mass: 373.02	
**M1**	4.14	361.2	Yes	No (345 = 373 - 28 (C2H4))	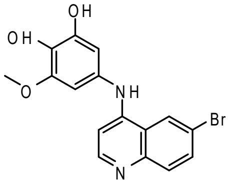 (Two demethylations. Exact demethylation positions not sure.)
**M2**	4.46	375.2	Yes	No (359 = 373 - 14 (CH2))	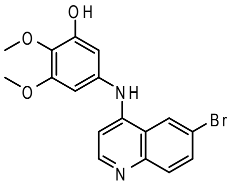 (Demethylation. Exact demethylation position not sure.)
**M3**	4.67	375.2	Yes	No (359 = 373 - 14 (CH2))	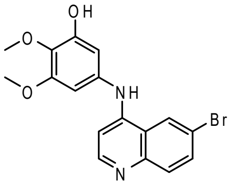 (Demethylation. Exact demethylation position not sure.)
**M4**	4.76	405.2	No		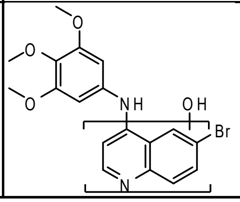 (Quinoline ring oxidation. Exact oxidation position not sure.)

Table S1. Metabolites of **1** (CA93.0) Observed in Mouse Liver Microsomal Incubation Continued

Metabolite	Retention (min)	Mass to Charge Ratio (m/z) in Postive Ion Mode	Presence of Product Ion (Yes/No)		Proposed Structure and Fragment of Metabolite
			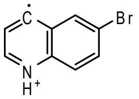 Exact Mass: 206.97	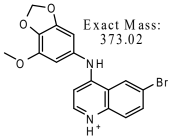	
**M5**	6.78	405.2	Weak (223 = 207 + 16 (O); 205 = 207 + 16 (O) - 18 (H_2O))		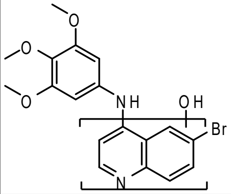 (Quinoline ring oxidation. Exact oxidation position not sure.)
**M6**	7.66	373.2	Yes	No	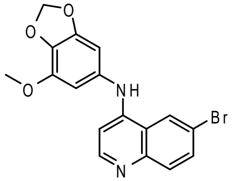
**CA93.0**	5.06	389.2	Yes		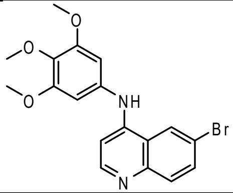

Table S2. Peak Area and Percent Peak Area of Metabolites of **1** (CA93.0) Observed in Mouse Liver Microsomal Incubation

Metabolite	Retention (min)	Mass to Charge Ratio (m/z)	Peak Area in Liver Microsomes	% Peak Area (over CT10258)	Rank in Peak Area Among Metabolites
			Mouse	Mouse	Mouse
**M1**	4.14	361.2	3.76E+05	1.2	4
**M2**	4.46	375.2	1.04E+07	32.4	1
**M3**	4.67	375.2	8.40E+05	2.6	3
**M4**	4.76	405.2	4.48E+04	0.1	6
**M5**	6.78	405.2	2.30E+05	0.7	5
**M6**	7.66	373.2	2.02E+06	6.3	2
**CA93.0**	5.06	389.2	3.21E+07	100.0	N/A

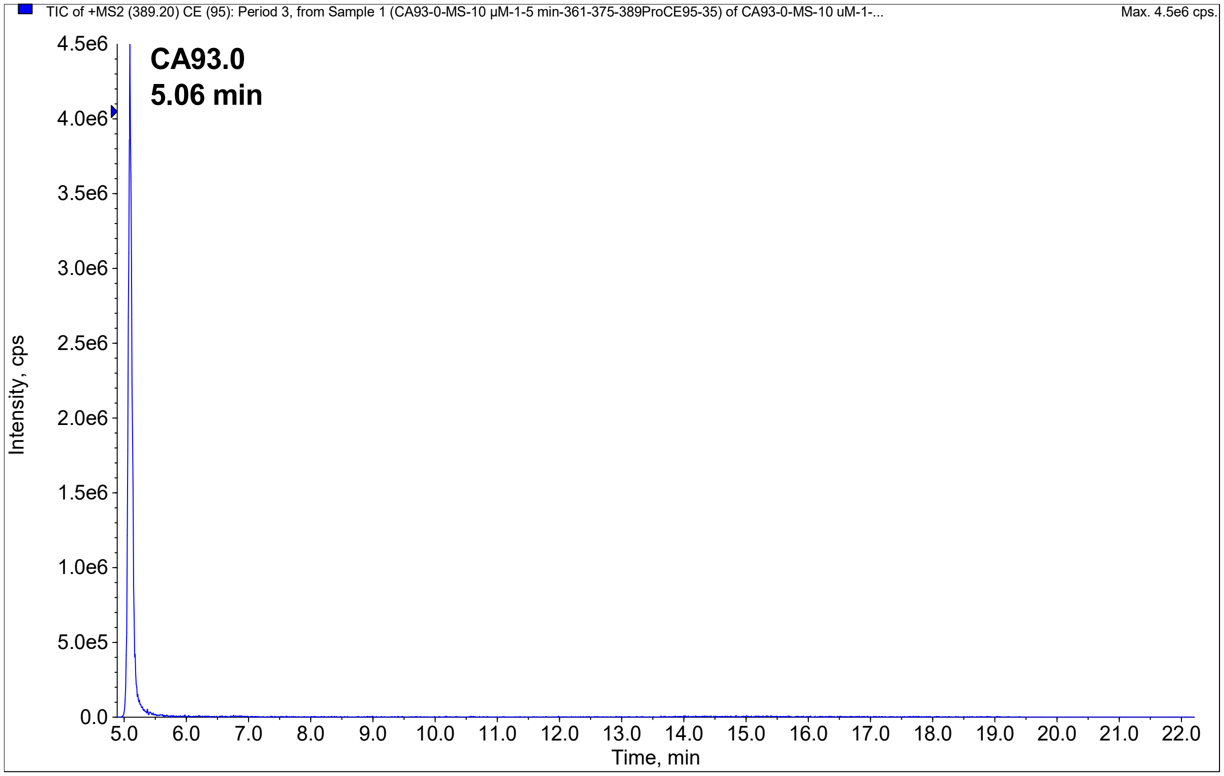

Figure S1. Total ion current chromatogram from product ion scan of ion 389.2 (m/z) from 5 µL injection of 10 µM of **1** (CA93.0), incubated in mouse liver microsomes for 5 minutes.

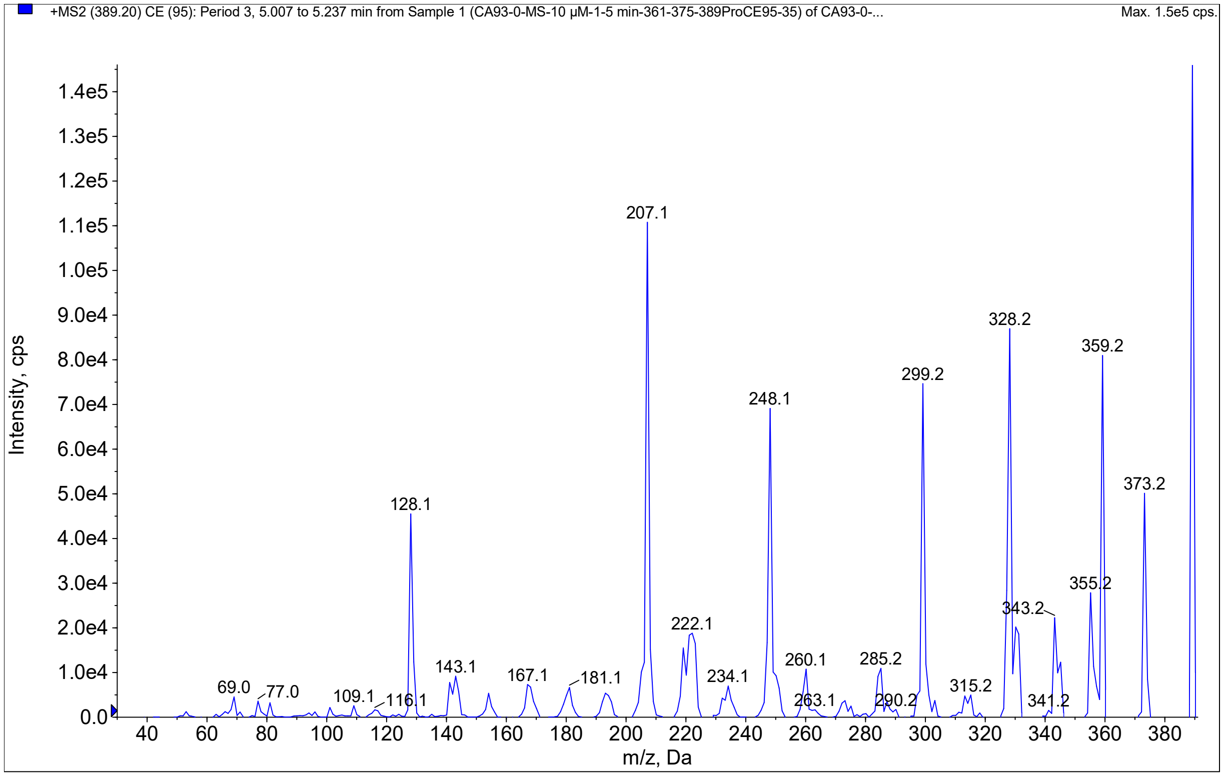

Figure S2. Product ion spectrum for ion 389.2 (m/z) derived from the peak at 5.06 min in Figure S1.

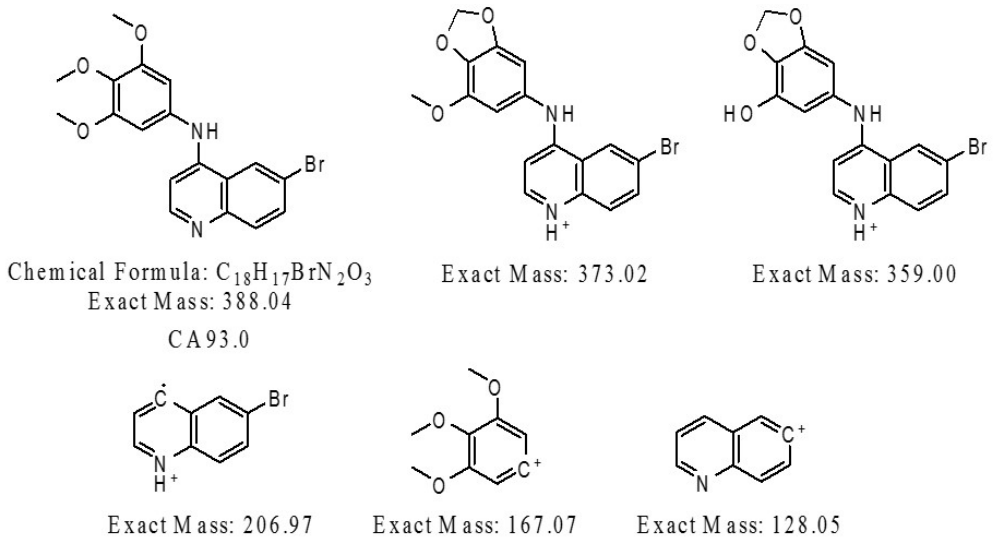

Figure S3. Assignment of fragments in **1** (CA93.0) product ion spectrum.

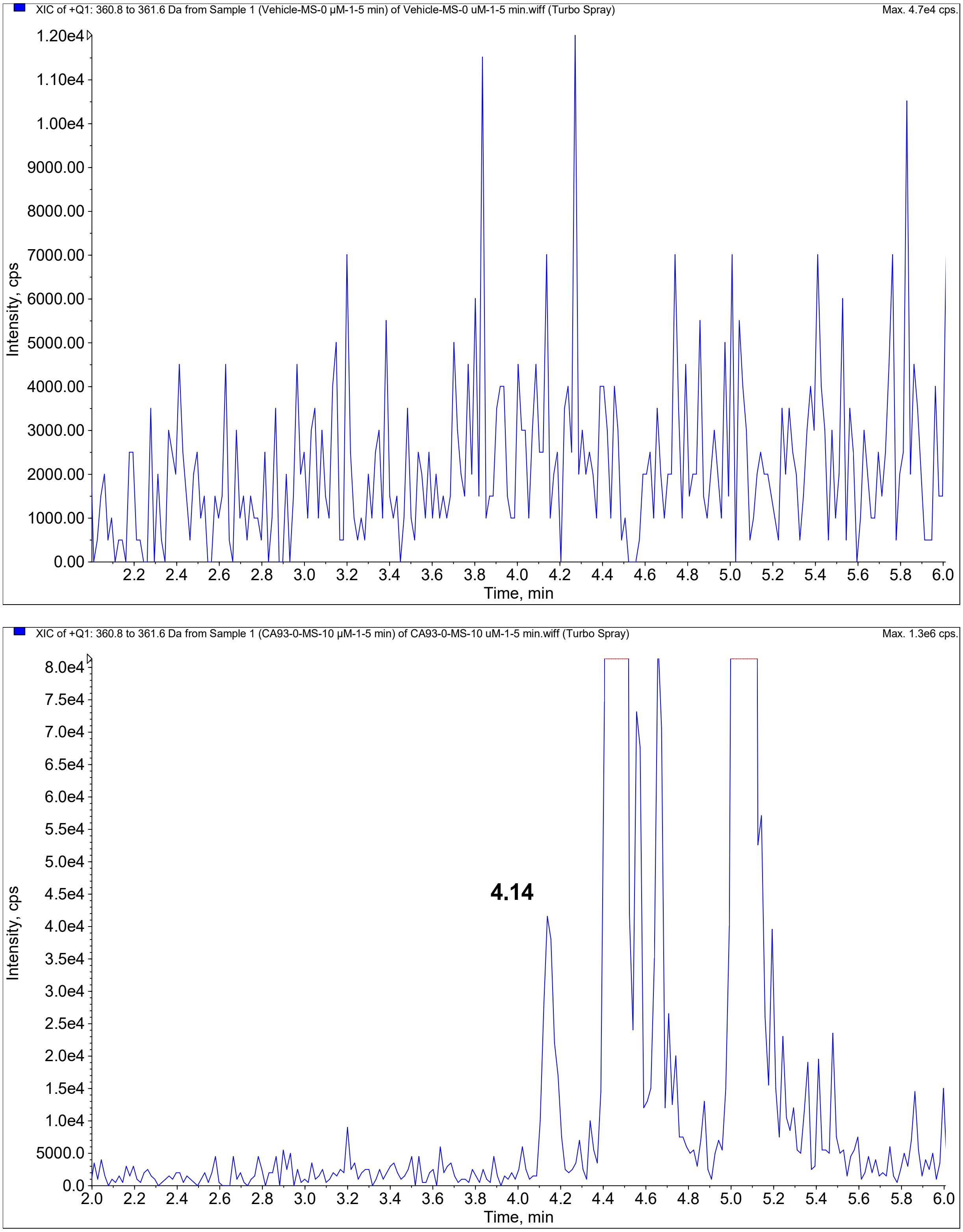

Figure S4. Extract ion (m/z 361) chromatograms from vehicle control sample Vehicle-MS-1 (top) and study sample **1**-10µM-MS-1 (bottom).

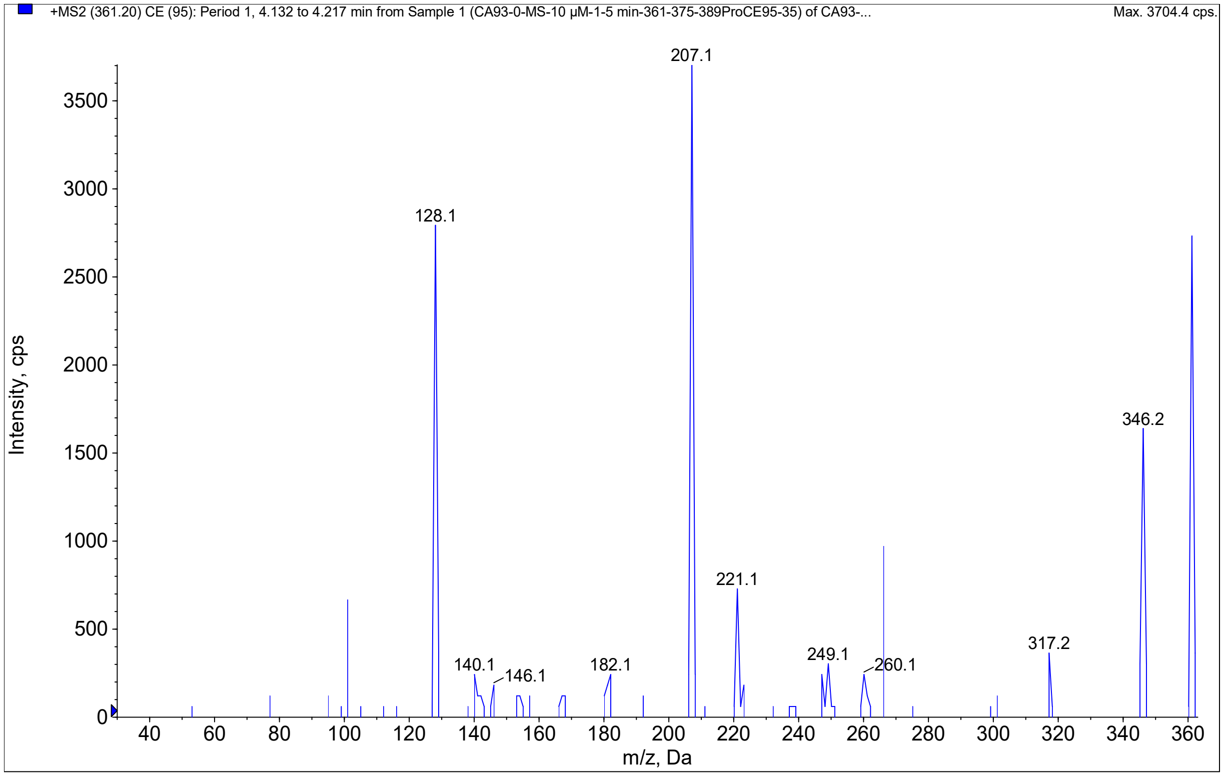

Figure S5. Product ion spectrum for ion 361 (m/z) derived from a peak at 4.14 min in the total ion current chromatogram from product ion scan of ion 361 (m/z) from study sample **1**-10µM-MS-1.

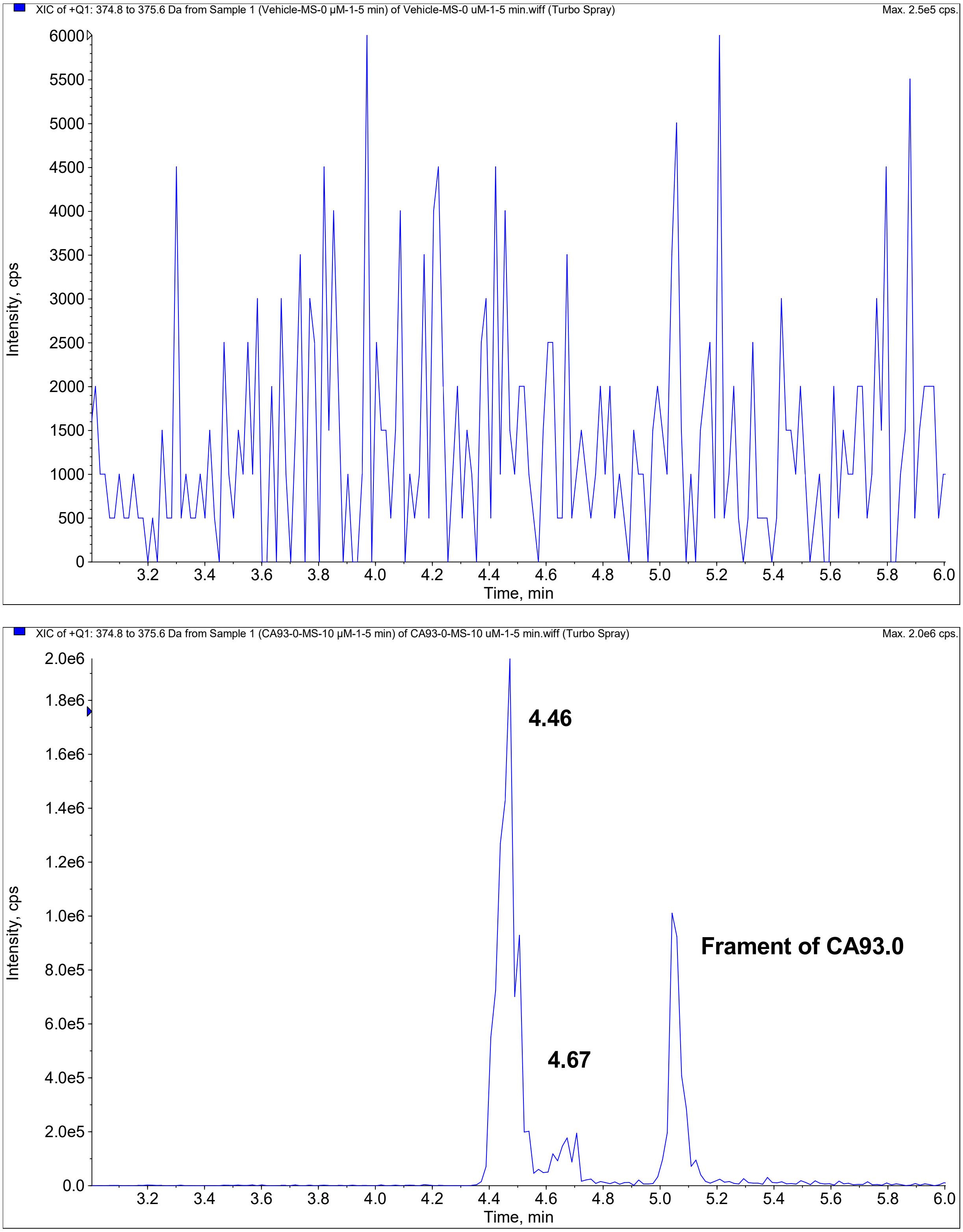

Figure S6. Extract ion (m/z 375) chromatograms from vehicle control sample Vehicle-MS-1 (top) and study sample **1**-10µM-MS-1 (bottom).

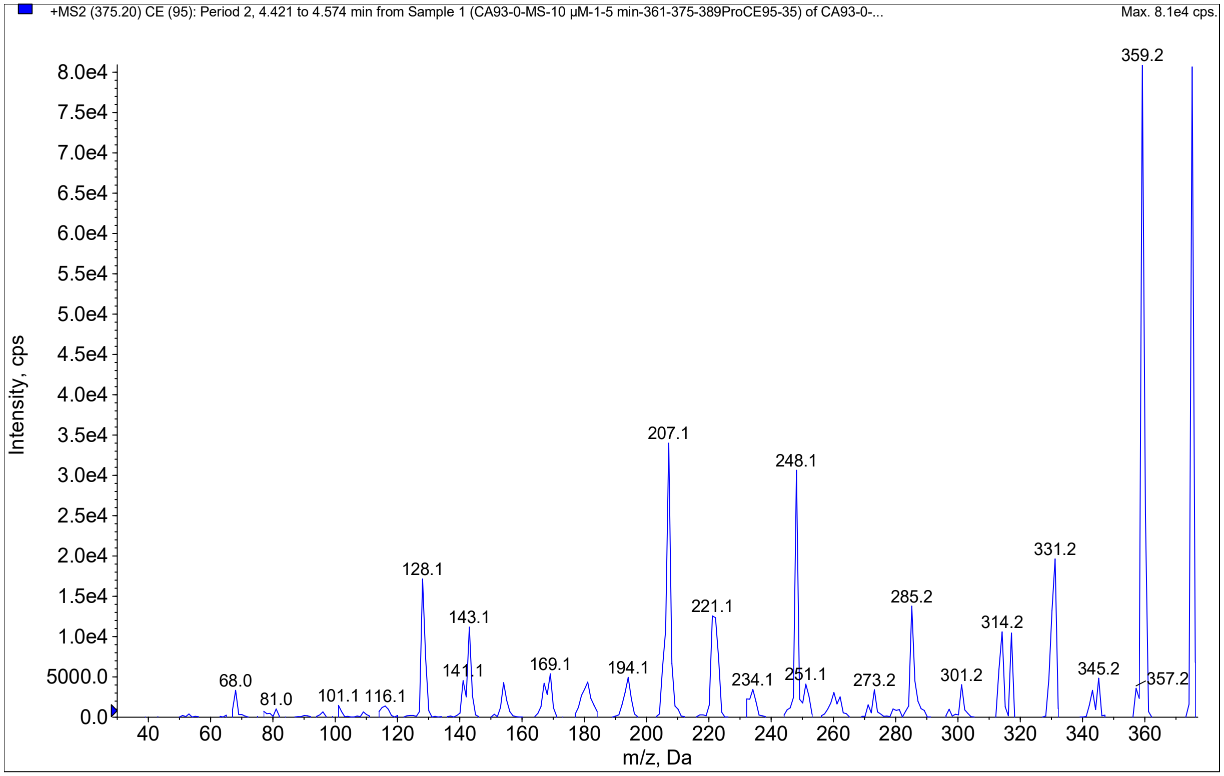

Figure S7. Product ion spectrum for ion 375 (m/z) derived from a peak at 4.46 min in the total ion current chromatogram from product ion scan of ion 375 (m/z) from study sample **1**-10μM-MS-1.

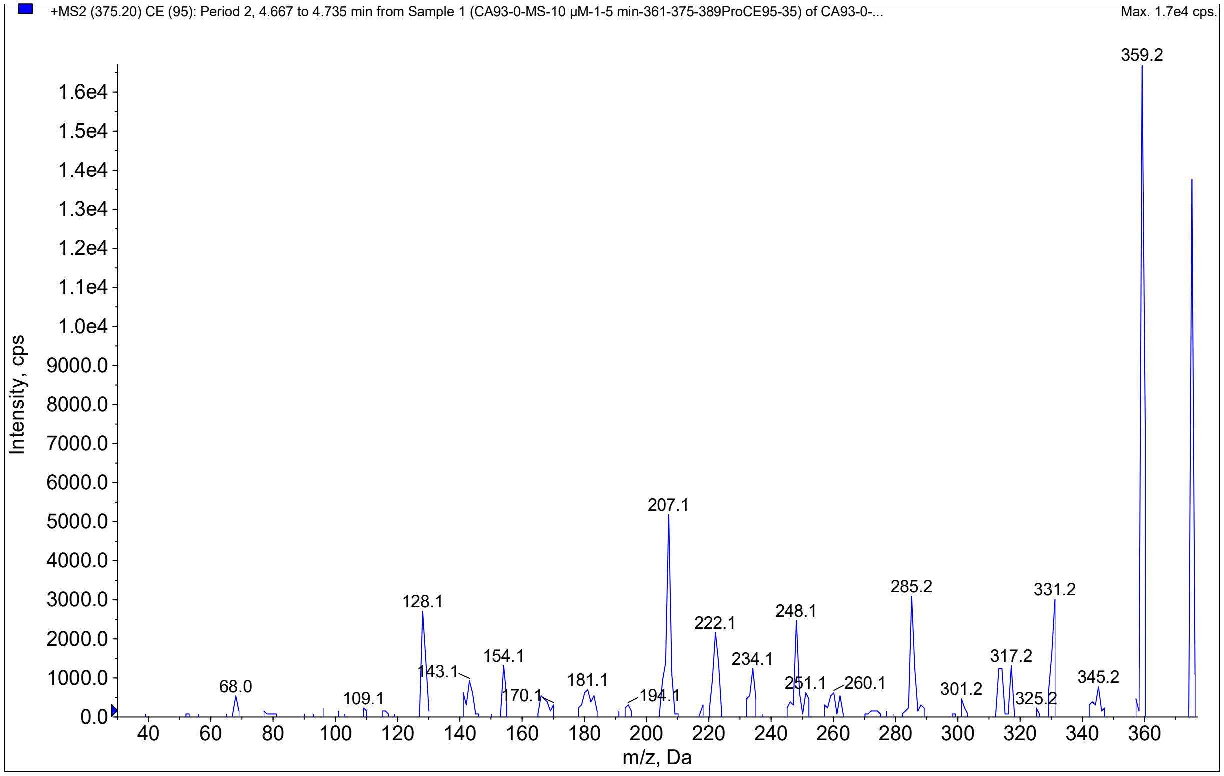

Figure S8. Product ion spectrum for ion 375 (m/z) derived from a peak at 4.67 min in the total ion current chromatogram from product ion scan of ion 375 (m/z) from study sample **1**-10µM-MS-1.

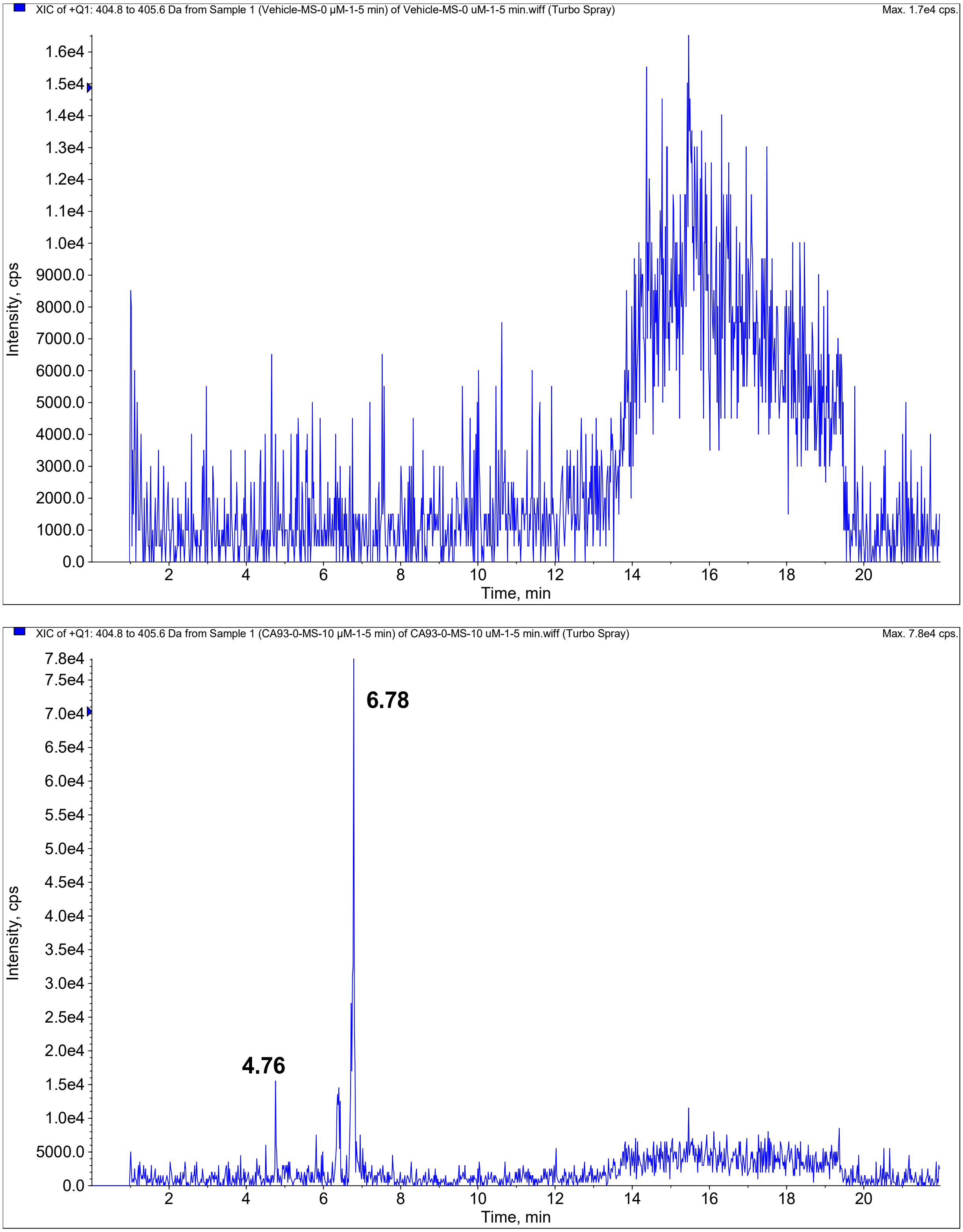

Figure S9. Extract ion (m/z 405) chromatograms from vehicle control sample Vehicle-MS-1 (top) and study sample **1**-10μM-MS-1 (bottom).

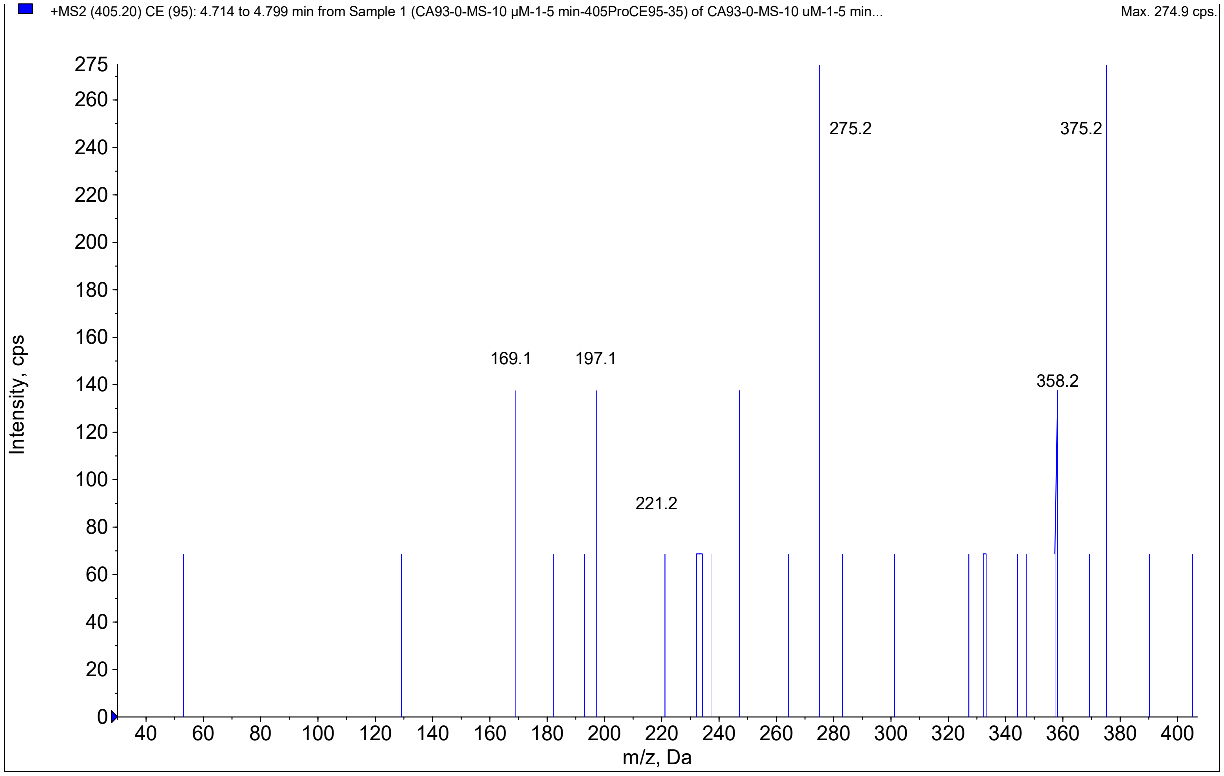

Figure S10. Product ion spectrum for ion 405 (m/z) derived from a peak at 4.76 min in the total ion current chromatogram from product ion scan of ion 405 (m/z) from study sample **1**-10µM-MS-1.

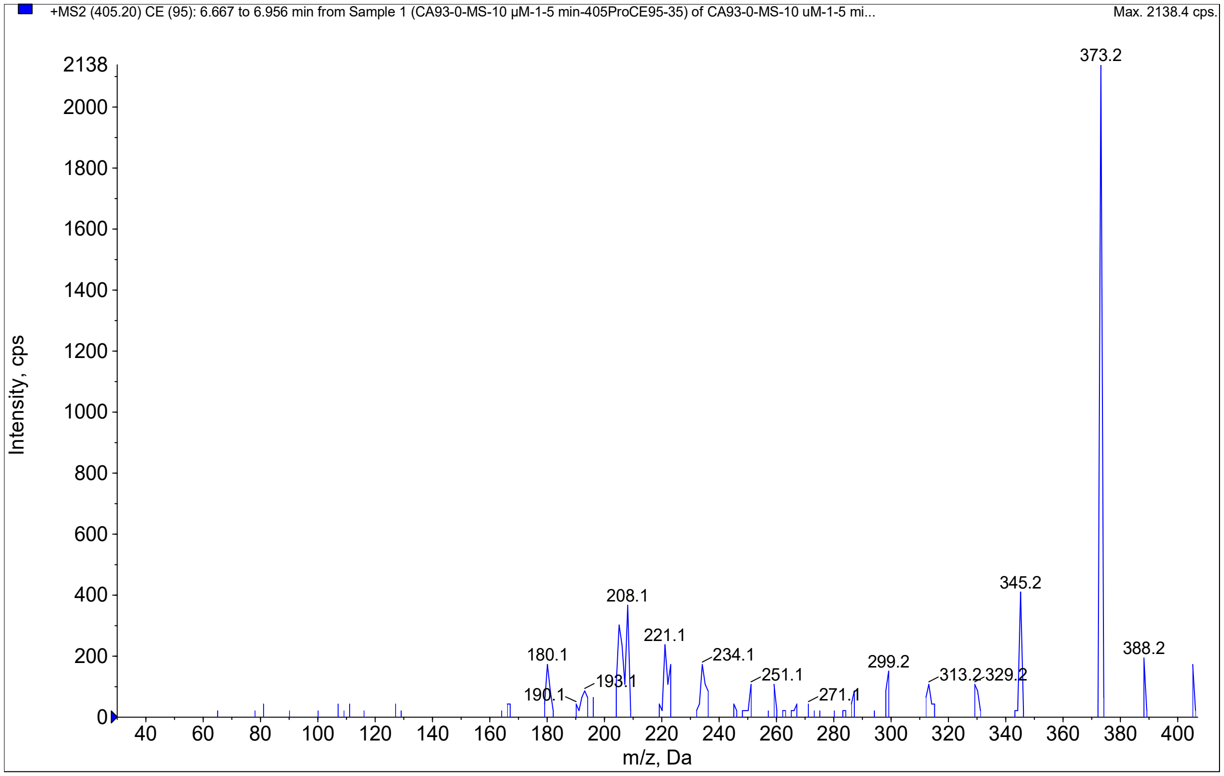

Figure S11. Product ion spectrum for ion 405 (m/z) derived from a peak at 6.78 min in the total ion current chromatogram from product ion scan of ion 405 (m/z) from study sample **1**-10µM-MS-1.

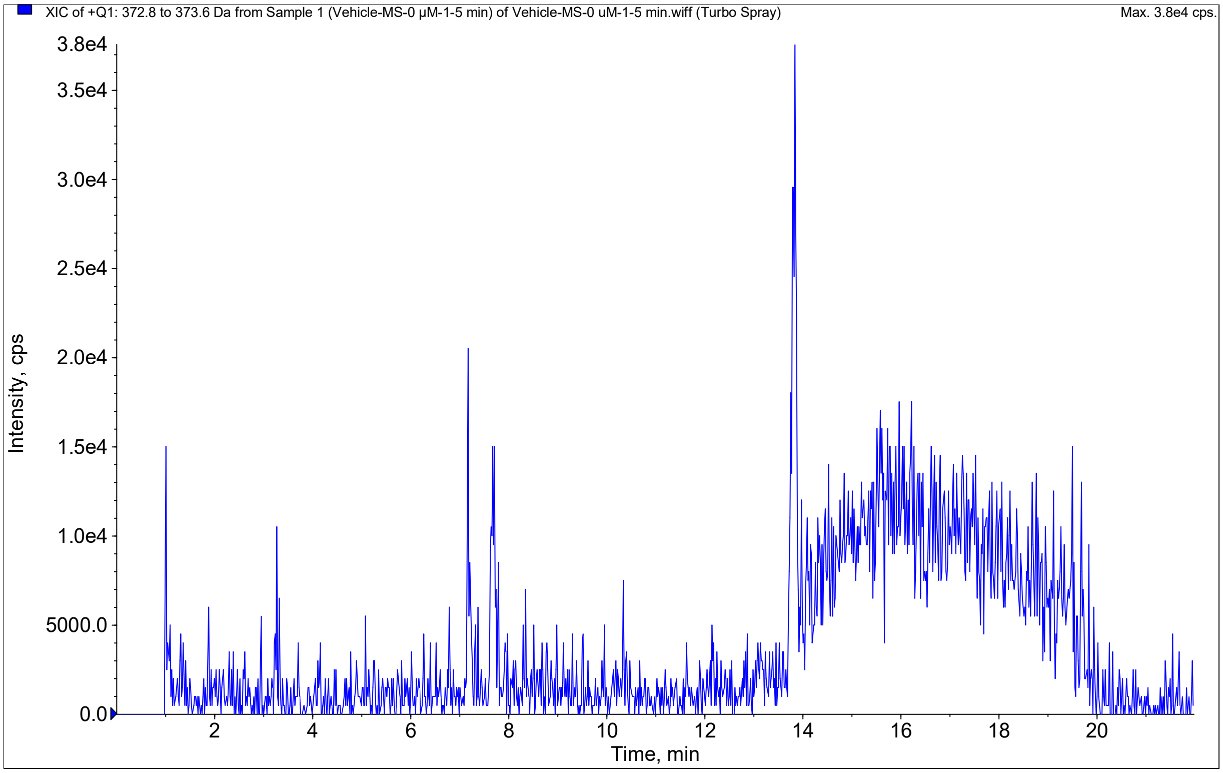

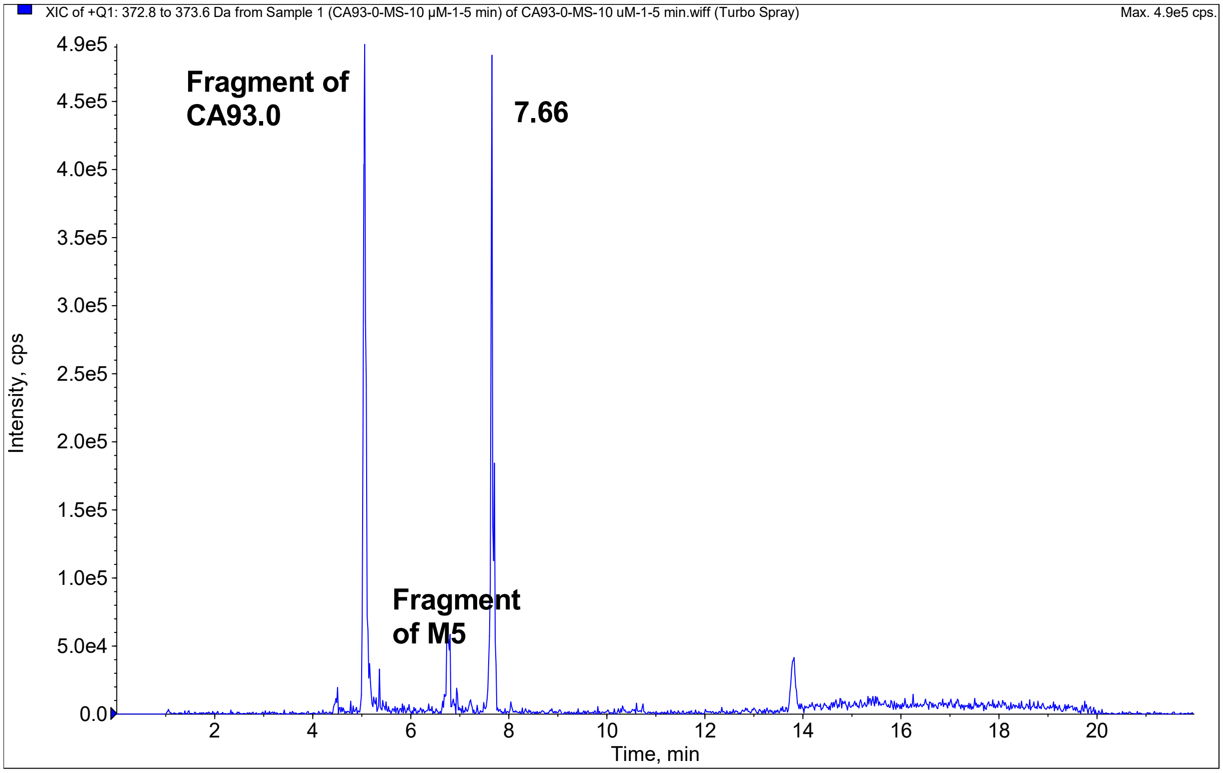

Figure S12. Extract ion (m/z 373) chromatograms from vehicle control sample Vehicle-MS-1 (top) and study sample **1**-10μM-MS-1 (bottom).

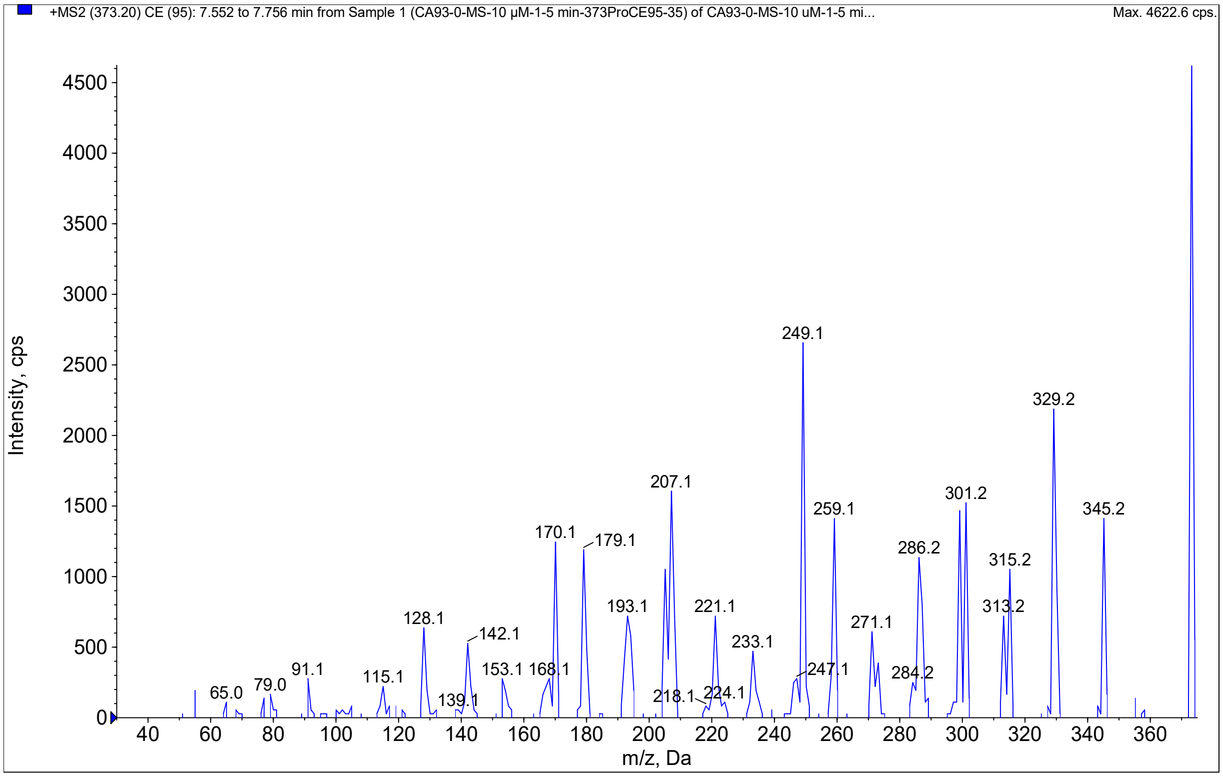

Figure S13. Product ion spectrum for ion 373 (m/z) derived from a peak at 7.66 min in the total ion current chromatogram from product ion scan of ion 373 (m/z) from study sample **1**-10µM-MS-1.

3. Metabolite Profiling of 8

3.1. Product Ion Spectrum of 8 and Assignment of Fragments

The total ion current chromatogram from product ion scan of ion 371.2 (m/z) from 5 µL injection of 10 µM of **8** incubated in mouse liver microsomes for 30 minutes is shown in **Figure S14**. The product ion spectrum derived from the peak in Figure 1 is shown in **Figure S15**. The assignment of fragments is shown in **Figure S16**.

3.2. Identification of Metabolites in Mouse Liver Microsomal Incubation

3.2.1. Identification of M1 at 3.38 min.

The component showed a quasi-molecular ion (MH) at m/z 329, a -42 dalton change relative to **8**. Extract ion (m/z 329) chromatograms, derived from the total ion chromatograms with Q1 scan from vehicle control sample Vehicle-MS-1 and study sample **8**-10µM-MS-1, are shown in **Figure S17**. There was a peak at 3.38 min in the chromatogram for the study sample. This peak was not observed in the chromatogram for the vehicle control sample.

The -42 dalton corresponds to the mass change for the loss of three methylene units from **8**. The product ion spectrum for M1 is shown in **Figure S18**. Product ion 189 (m/z) was not observed in the product ion spectrum for M1. Instead, product ion 175 (m/z) was observed, indicating the loss of one methylene unit in the group corresponding to fragment 189 (m/z). The loss of the other two methylene units must occur in other parts of **8**. A structure consistent with the product ion spectrum was proposed for M1 as shown in **Table S3**.

3.2.2. Identification of M2 at 3.68 min and M3 at 3.93 min.

The components showed a quasi-molecular ion (MH) at m/z 343, a -28 dalton change relative to **8**. Extract ion (m/z 343) chromatograms, derived from the total ion chromatograms with Q1 scan from vehicle control sample Vehicle-MS-1 and study sample **8**-10µM-MS-1, are shown in **Figure S19**. There were peaks at 3.68 min and 3.93 min., respectively, in the chromatogram for the study sample. These peaks were not observed in the chromatogram for the vehicle control sample. The -28 dalton corresponds to the mass change for the loss of two methylene units from **8**.

The product ion spectrum for M2 is shown in **Figure S20**. Little or no product ion 189 (m/z) signal was observed in the product ion spectrum for M2. Instead, product ion 175 (m/z) was observed, indicating the loss of one methylene unit in the group corresponding to fragment 189 (m/z). The loss of the other methylene unit must occur in other parts of **8**. A structure consistent with the product ion spectrum was proposed for M2 as shown in **Table S3**.

The product ion spectrum for M3 is shown in **Figure S21**. Product ion 189 (m/z) was abundant in the product ion spectrum for M3, indicating no loss of methylene units in the group corresponding to fragment 189 (m/z). The loss of two methylene units must occur in other parts of **8**. A structure consistent with the product ion spectrum was proposed for M3 as shown in **Table S3**.

3.2.3. Identification of M4, M5, M6, and M7 at 4.21, 4.31, 4.40, and 4.50 min, Respectively

The components showed a quasi-molecular ion (MH) at m/z 357, a -14 dalton change relative to **8**. Extract ion (m/z 357) chromatograms, derived from the total ion chromatograms with Q1 scan from vehicle control sample Vehicle-MS-1 and study sample **8**-10μM-MS-1, are shown in **Figure S22**. There were peaks at 4.21, 4.31, 4.40, and 4.50 min, respectively, in the chromatogram for the study sample. These peaks were not observed in the chromatogram for the vehicle control sample. The -14 dalton corresponds to the mass change for the loss of one methylene unit from **8**.

The product ion spectrum for M4 is shown in Figure 10. Product ion 189 (m/z) was abundant in the product ion spectrum for M4, indicating no loss of methylene units in the group corresponding to fragment 189 (m/z). The loss of one methylene unit must occur in other parts of **8**. A structure consistent with the product ion spectrum was proposed for M4 as shown in **Table S3**.

The product ion spectrum for M5 is shown in **Figure S23**. Little or no product ion 189 (m/z) signal was observed in the product ion spectrum for M5. Instead, product ion 175 (m/z) was observed, indicating the loss of one methylene unit in the group corresponding to fragment 189 (m/z). A structure consistent with the product ion spectrum was proposed for M5 as shown in **Table S3**.

The product ion spectrum for M6 is shown in **Figure S24**. Product ion 189 (m/z) was abundant in the product ion spectrum for M6, indicating no loss of methylene units in the group corresponding to fragment 189 (m/z). The loss of one methylene unit must occur in other parts of **8**. A structure consistent with the product ion spectrum was proposed for M6 as shown in **Table S3**.

The product ion spectrum for M7 is shown in **Figure S25**. Little or no product ion 189 (m/z) signal was observed in the product ion spectrum for M7. Instead, product ion 175 (m/z) was observed, indicating the loss of one methylene unit in the group corresponding to fragment 189 (m/z). A structure consistent with the product ion spectrum was proposed for M7 as shown in **Table S3**

3.3. Peak Areas of Metabolites and 8

The peak areas of metabolites and **8** are listed in **Table S4**. The metabolites with most intense signals are M4 and M5, from the loss of one methylene unit from **8**. The major metabolism sites for **8** are methoxy groups. Major metabolites are formed through O-demethylation.

3.4. Metabolite Profiling Graphs and Tables for 8

Table S3. Metabolites of **8** (CA75) Observed in Mouse Liver Microsomal Incubation

Metabolite	Retention (min)	Mass to Charge Ratio (m/z) in Postive Ion Mode	Presence of Product Ion (Yes/No)		Proposed Structure and Fragment of Metabolite
			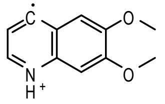 Exact Mass: 189.08	Extra Ions (m/z)	
**M1**	3.38	329.2	No (189 - 14 (CH2) = 175)	175	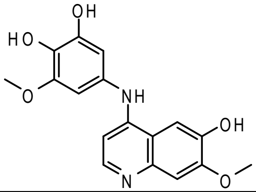 (Two demethylations in the top left group and another demethylaton in the bottom right group; Exact demethylation positions not sure.)
**M2**	3.68	343.2	Litte (189 - 14 (CH2) = 175)	175	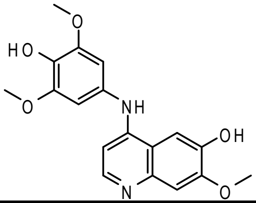 (One demethylation in the top left group and another demethylaton in the bottom right group; Exact demethylation positions not sure.)
**M3**	3.93	343.2	Yes		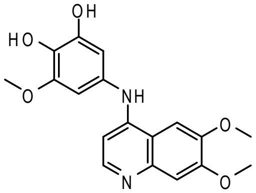 (Exact demethylation positions among the three methoxy groups not sure)
**M4**	4.21	357.2	Yes		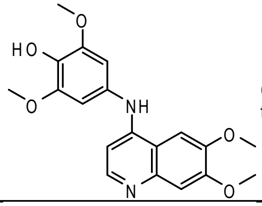 (Exact demethylation position among the three methoxy groups not sure)

Table S3. Metabolites of **8** (CA75) Observed in Mouse Liver Microsomal Incubation Continued

Metabolite	Retention (min)	Mass to Charge Ratio (m/z) in Postive Ion Mode	Presence of Product Ion (Yes/No) 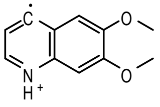 Exact Mass: 189.08	Extra Ions (m/z)	Proposed Structure and Fragment of Metabolite
**M5**	4.31	357.2	Litte (189 - 14 (CH2) = 175)	175	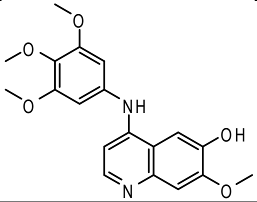 or 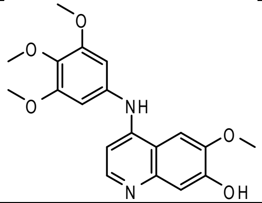
**M6**	4.40	357.2	Yes		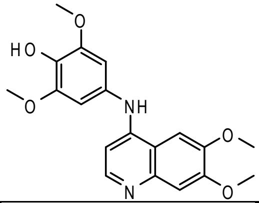 (Exact demethylation position among the three methoxy groups not sure)
**M7**	4.50	357.2	Litte (189 - 14 (CH2) = 175)	175	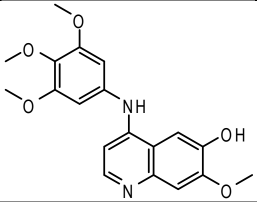 or 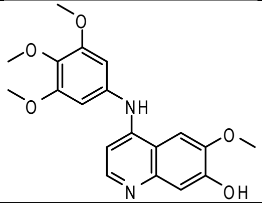
**CA75**	4.80	371.2	Yes	No	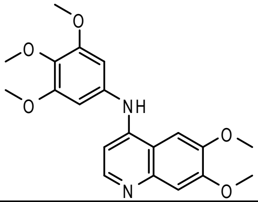

Table S4. Peak Area and Percent Peak Area of Metabolites of **8** (CA75) Observed in Mouse Liver Microsomal Incubation

Metabolite	Retention (min)	Mass to Charge Ratio (m/z)	Peak Area in Liver Microsomes	% Peak Area (over CA75)	Rank in Peak Area Among Metabolites
			Mouse	Mouse	Mouse
**M1**	3.38	329.2	9.51E+05	4.4	6
**M2**	3.68	343.2	2.40E+06	11.1	4
**M3**	3.93	343.2	1.77E+06	8.2	5
**M4**	4.21	357.2	1.08E+07	49.8	1
**M5**	4.31	357.2	7.22E+06	33.3	2
**M6**	4.40	357.2	7.96E+05	3.7	7
**M7**	4.50	357.2	2.60E+06	12.0	3
**CA75**	4.80	371.2	2.17E+07	100.0	N/A

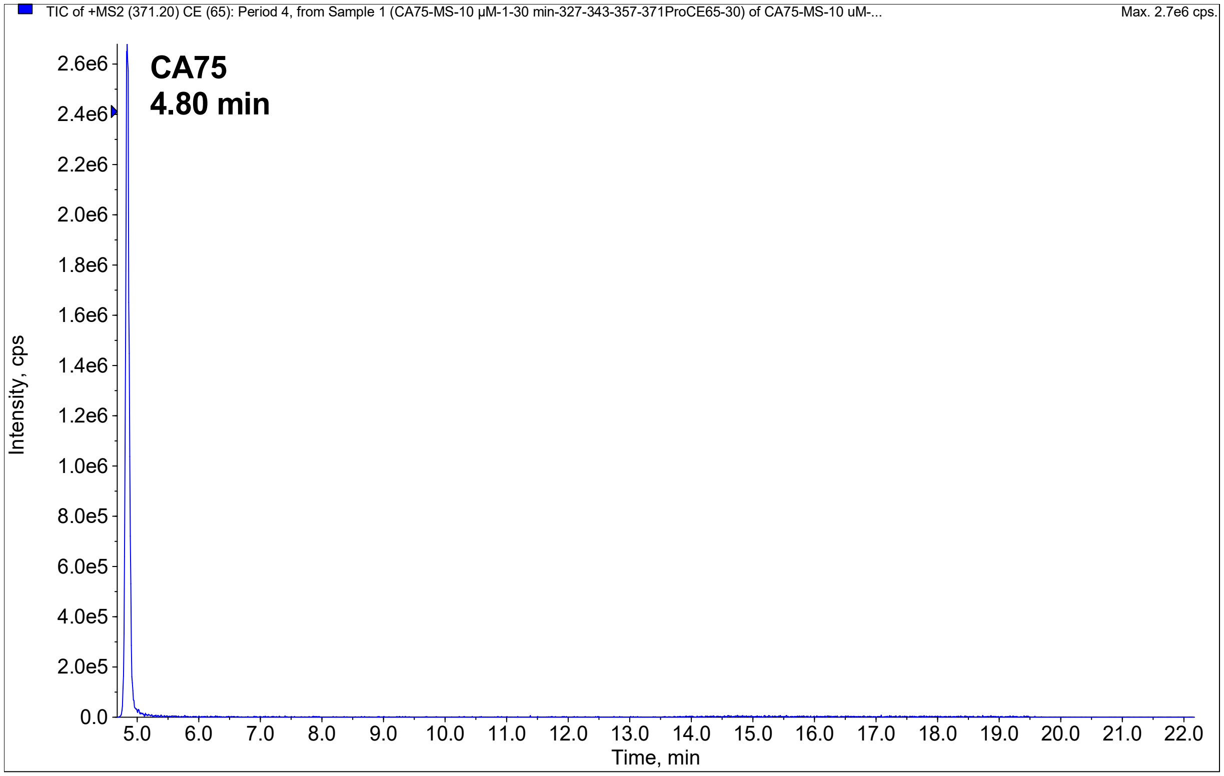

Figure S14. Total ion current chromatogram from product ion scan of ion 371.2 (m/z) from 5 µL injection of 10 µM of **8** incubated in mouse liver microsomes for 30 minutes.

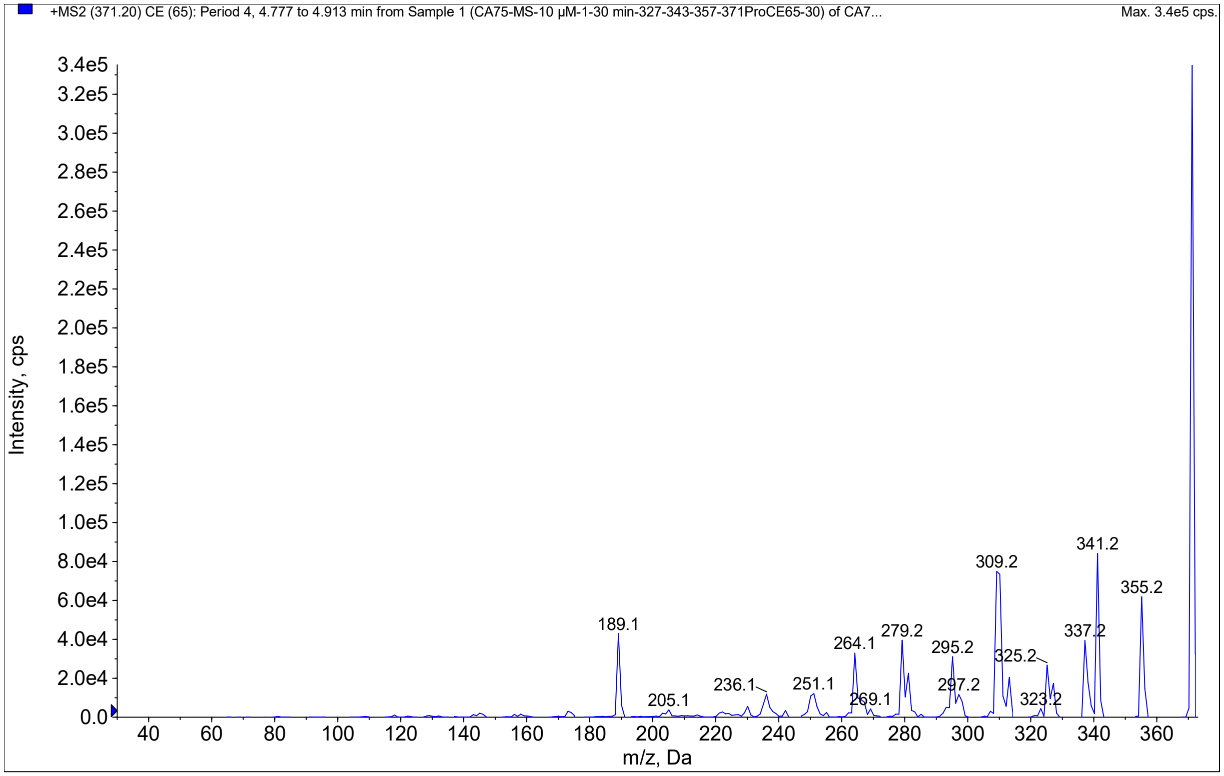

Figure S15. Product ion spectrum for ion 371.2 (m/z) derived from the peak at 4.88 min in **Figure S14**.

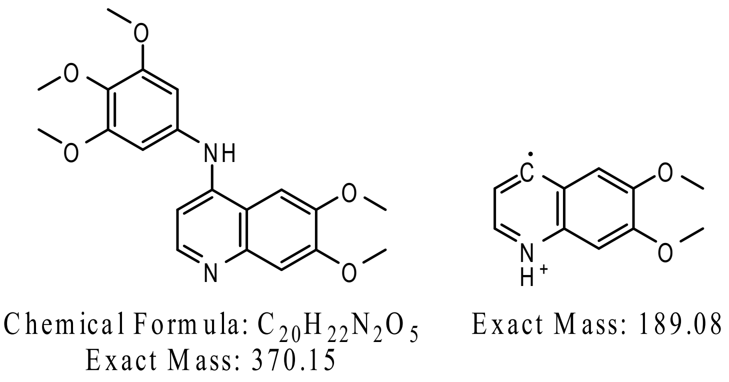

Figure S16. Assignment of fragment 189 (m/z) in **8** product ion spectrum.

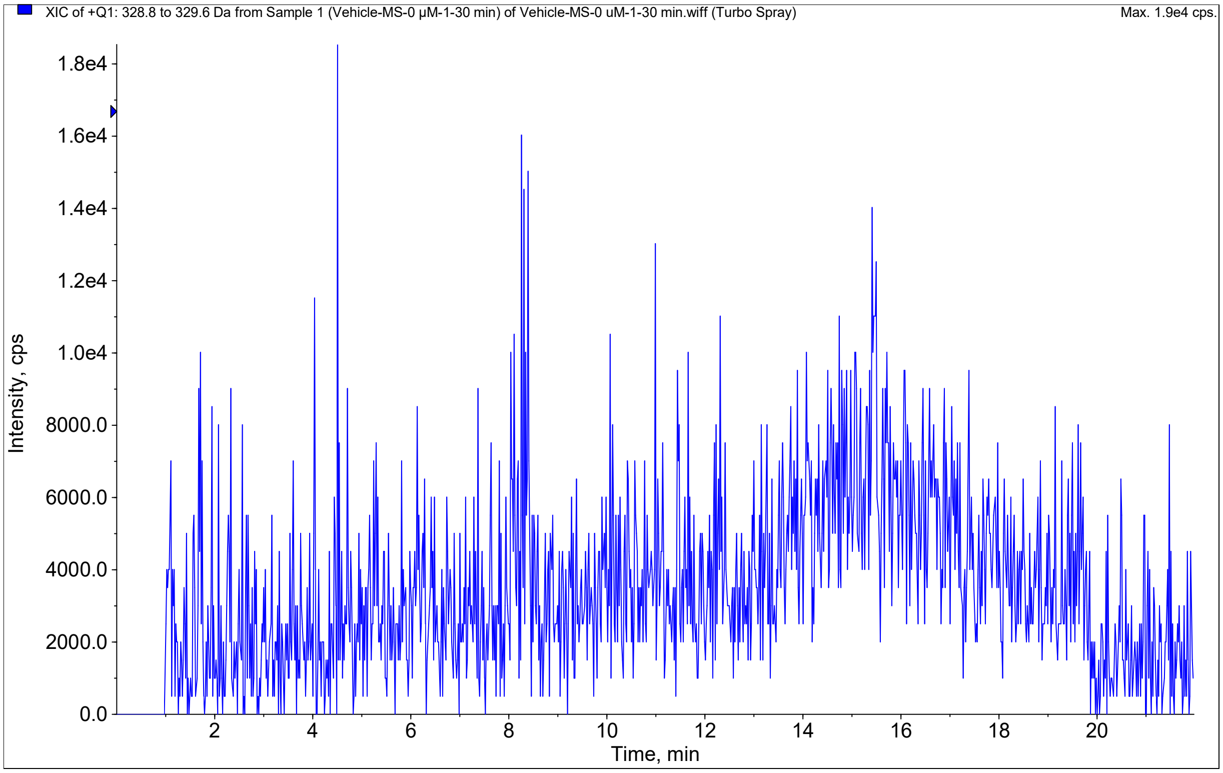

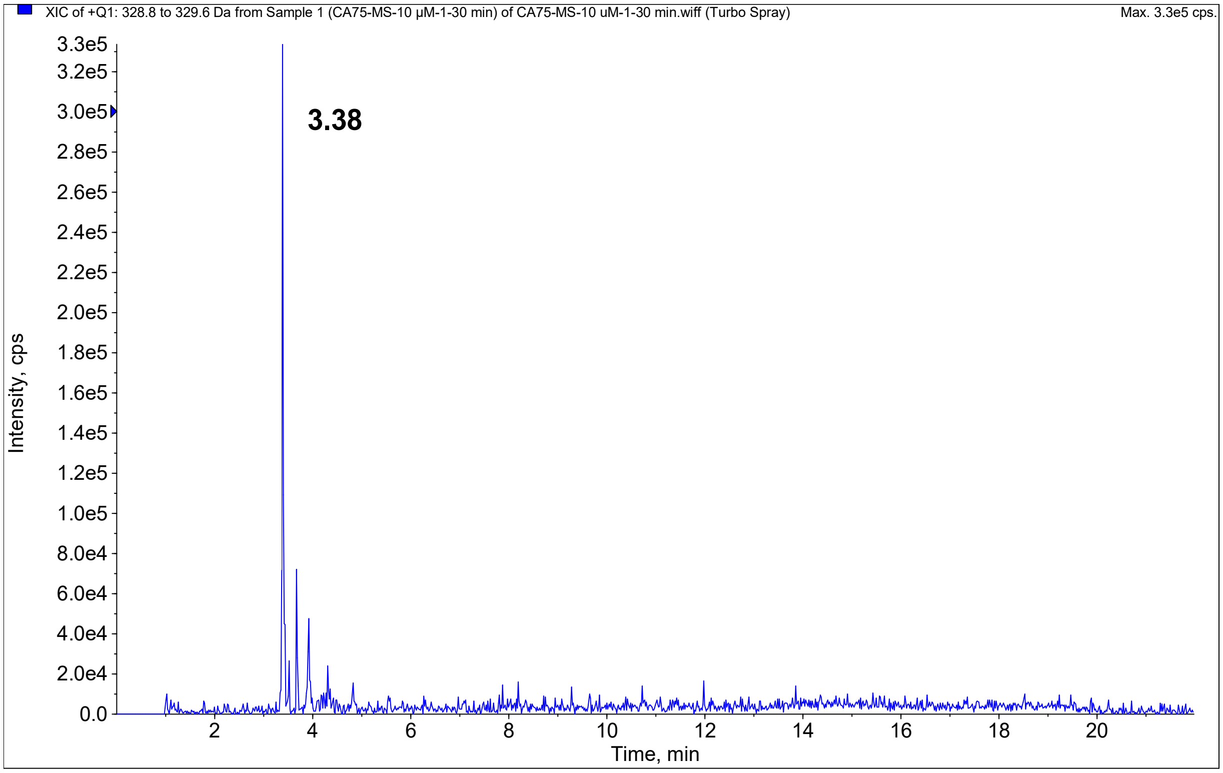

Figure S17. Extract ion (m/z 329) chromatograms from vehicle control sample Vehicle-MS-1 (top) and study sample **8**-10µM-MS-1 (bottom).

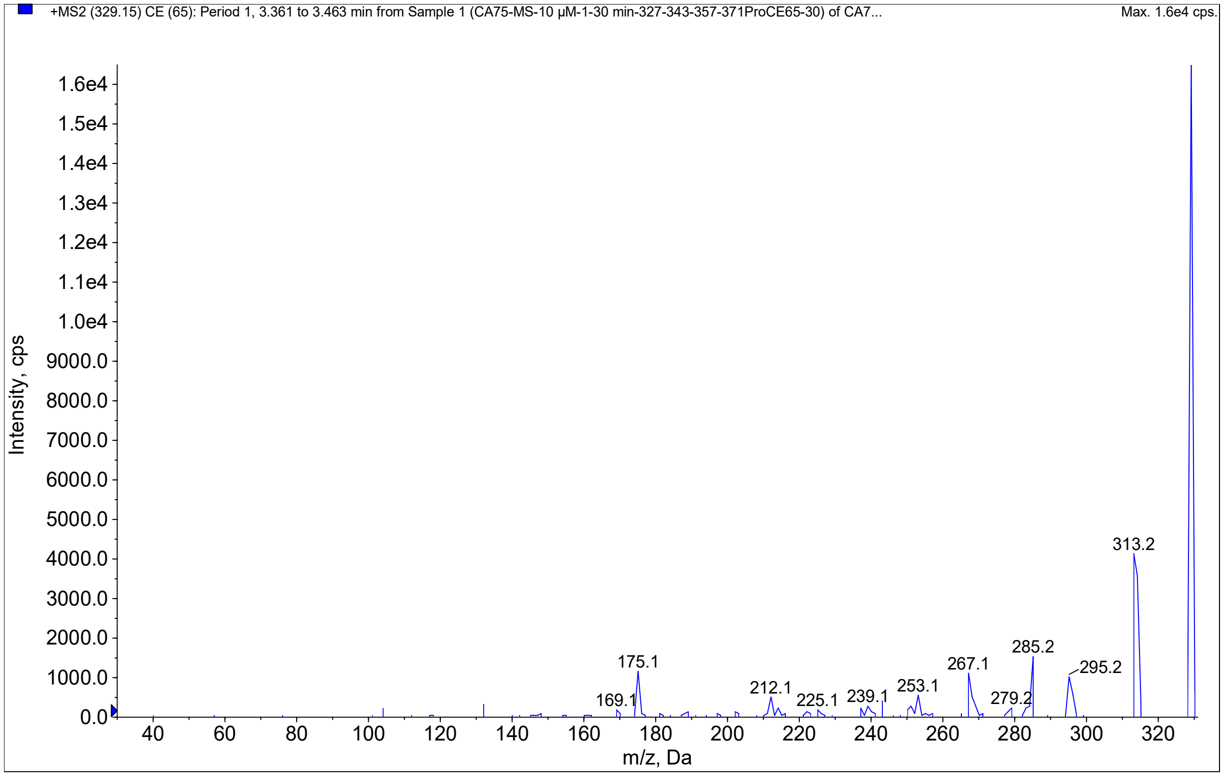

Figure S18. Product ion spectrum for ion 329 (m/z) derived from a peak at 3.38 min in the total ion current chromatogram from product ion scan of ion 329 (m/z) from study sample **8**-10µM-MS-1.

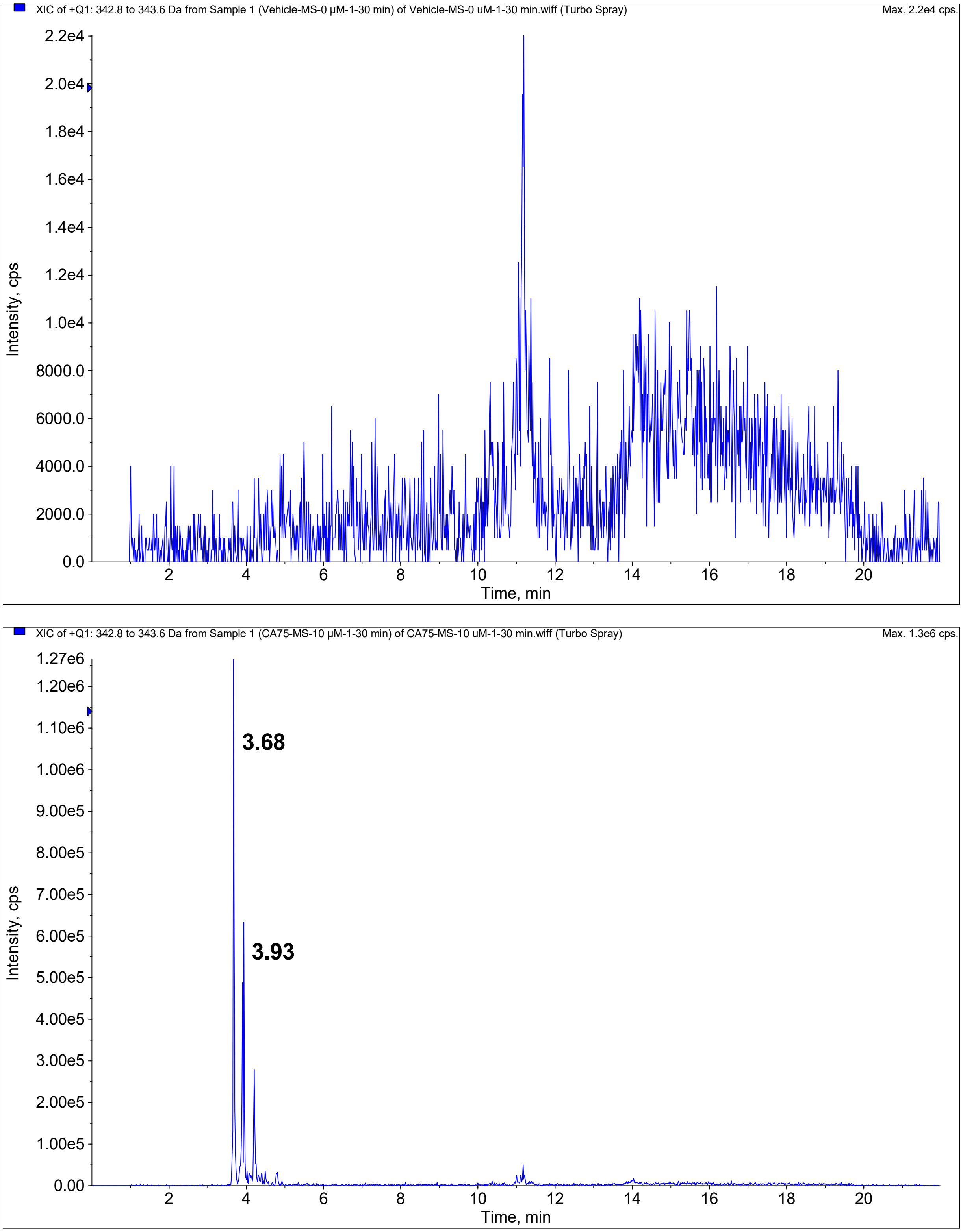

Figure S19. Extract ion (m/z 343) chromatograms from vehicle control sample Vehicle-MS-1 (top) and study sample **8**-10µM-MS-1 (bottom).

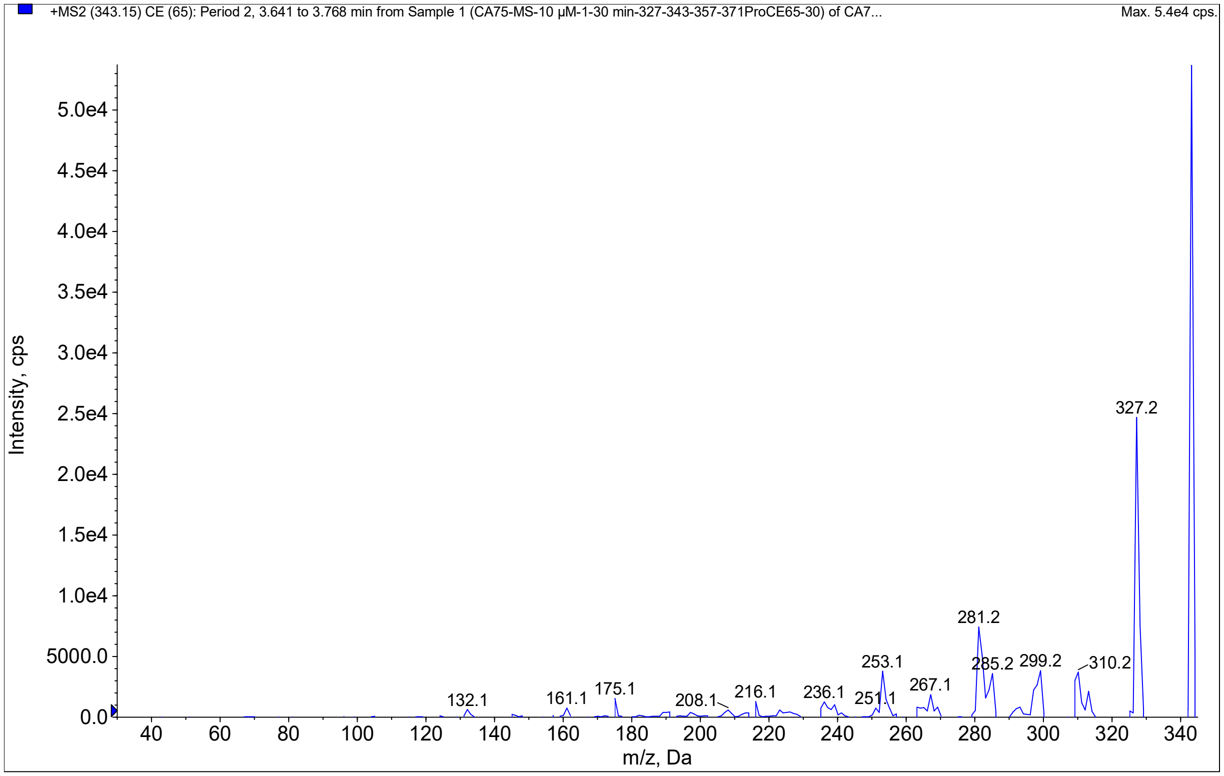

Figure S20. Product ion spectrum for ion 343 (m/z) derived from a peak at 3.68 min in the total ion current chromatogram from product ion scan of ion 343 (m/z) from study sample **8**-10µM-MS-1.

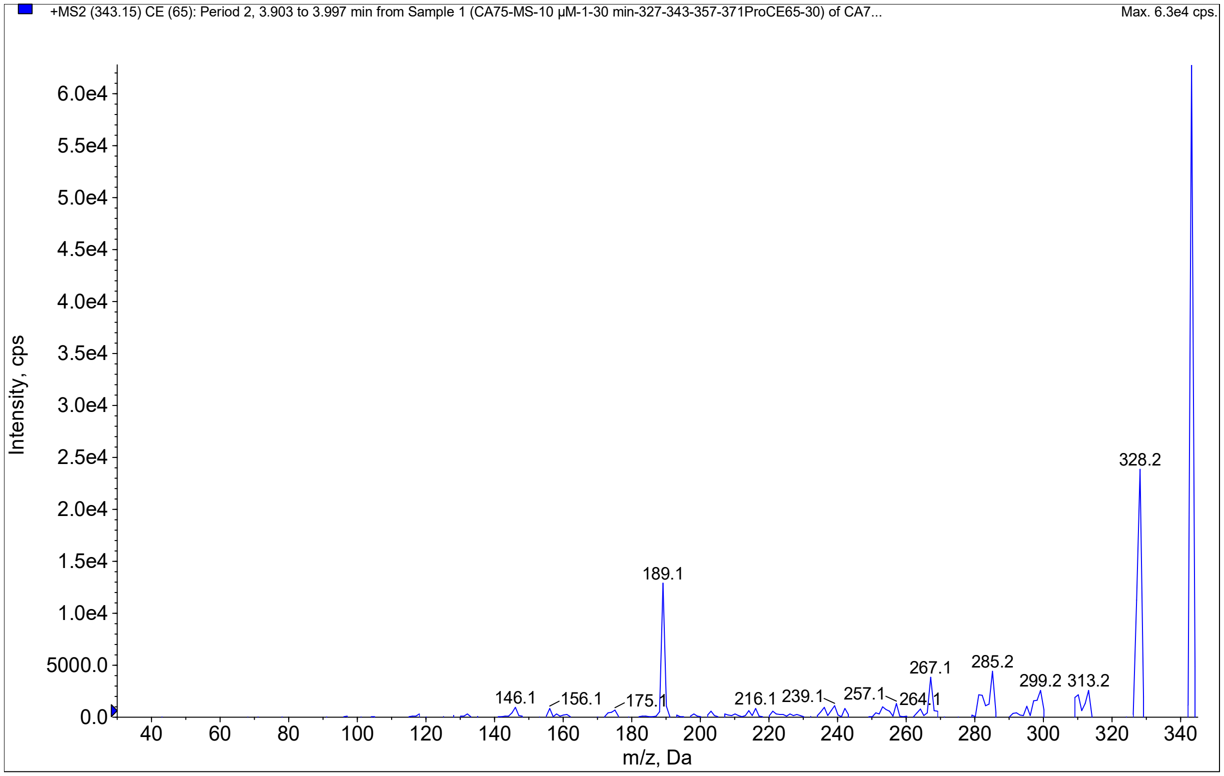

Figure S20. Product ion spectrum for ion 343 (m/z) derived from a peak at 3.93 min in the total ion current chromatogram from product ion scan of ion 343 (m/z) from study sample **8**-10µM-MS-1.

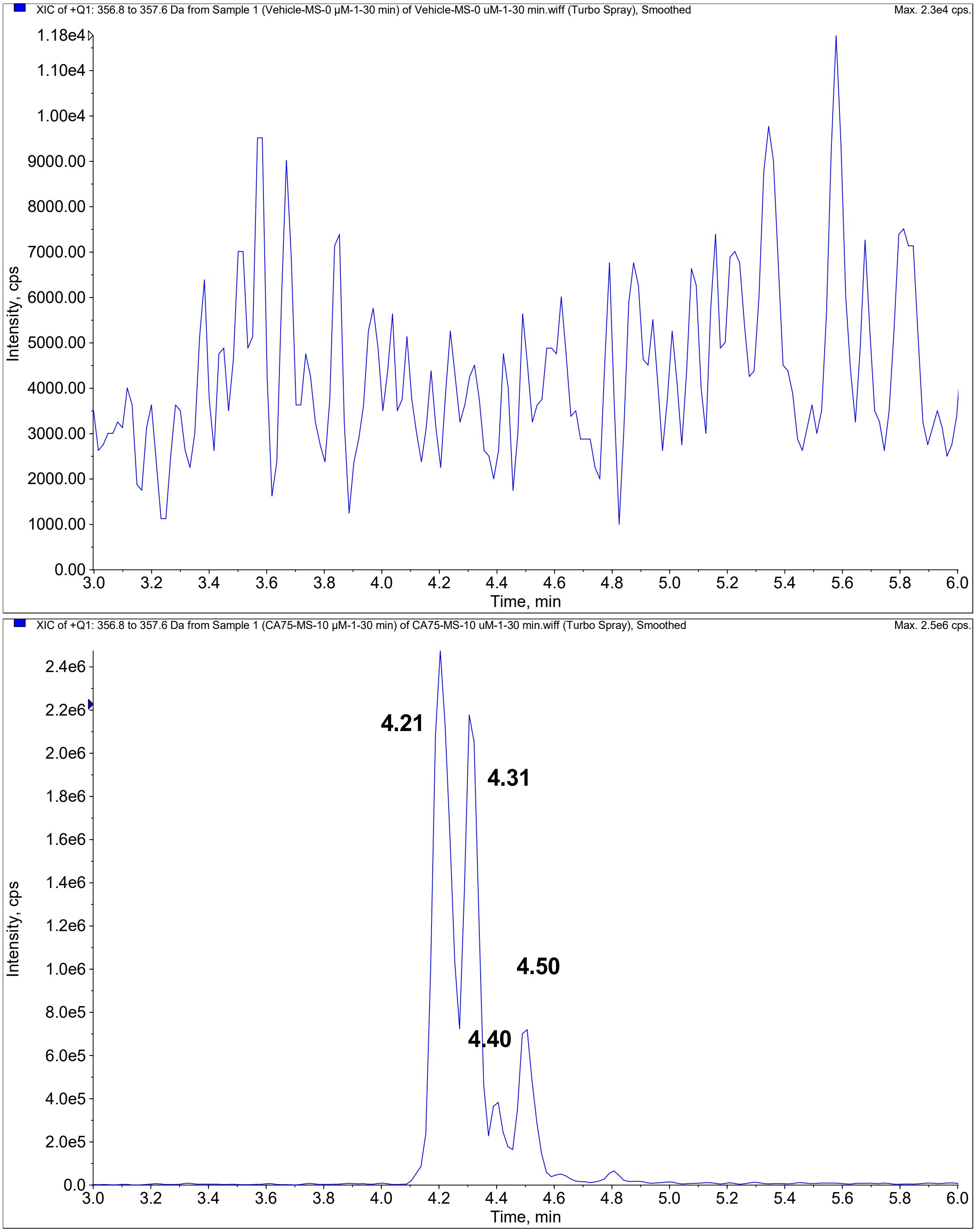

Figure S21. Extract ion (m/z 357) chromatograms from vehicle control sample Vehicle-MS-1 (top) and study sample **8**-10μM-MS-1 (bottom).

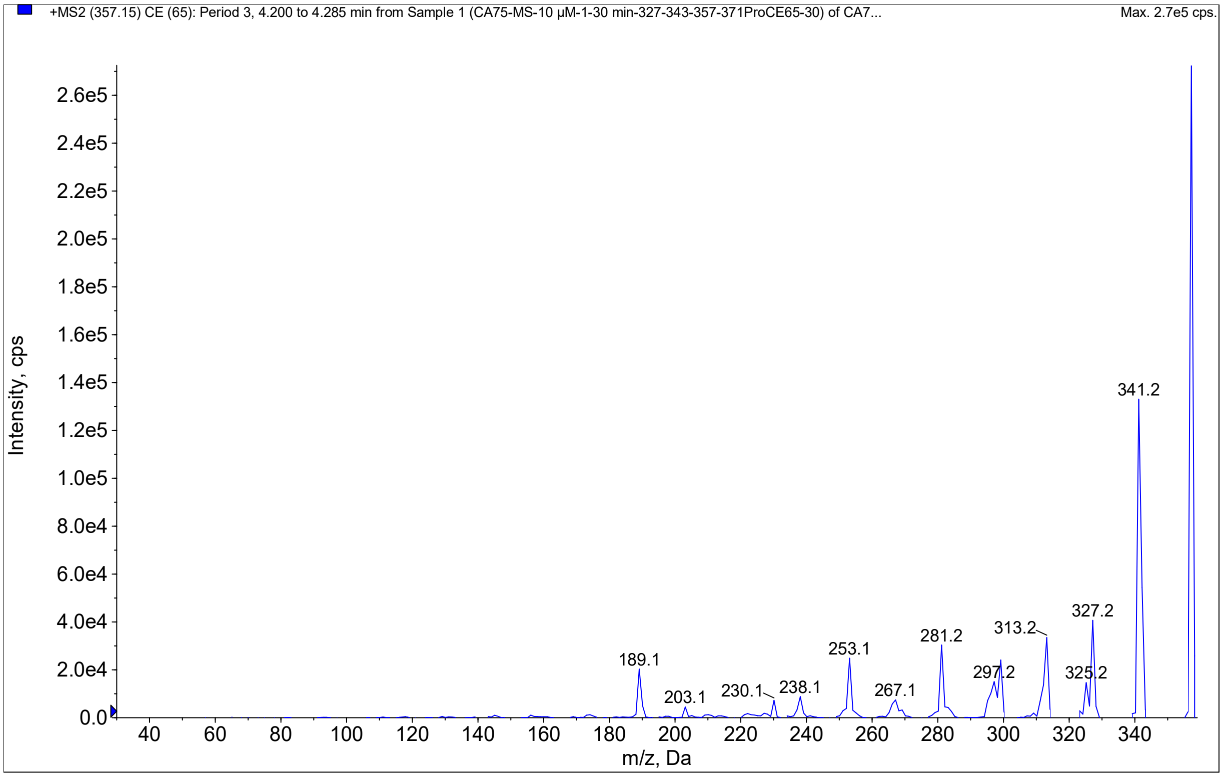

Figure S22. Product ion spectrum for ion 357 (m/z) derived from a peak at 4.21 min in the total ion current chromatogram from product ion scan of ion 357 (m/z) from study sample **8**-10µM-MS-1.

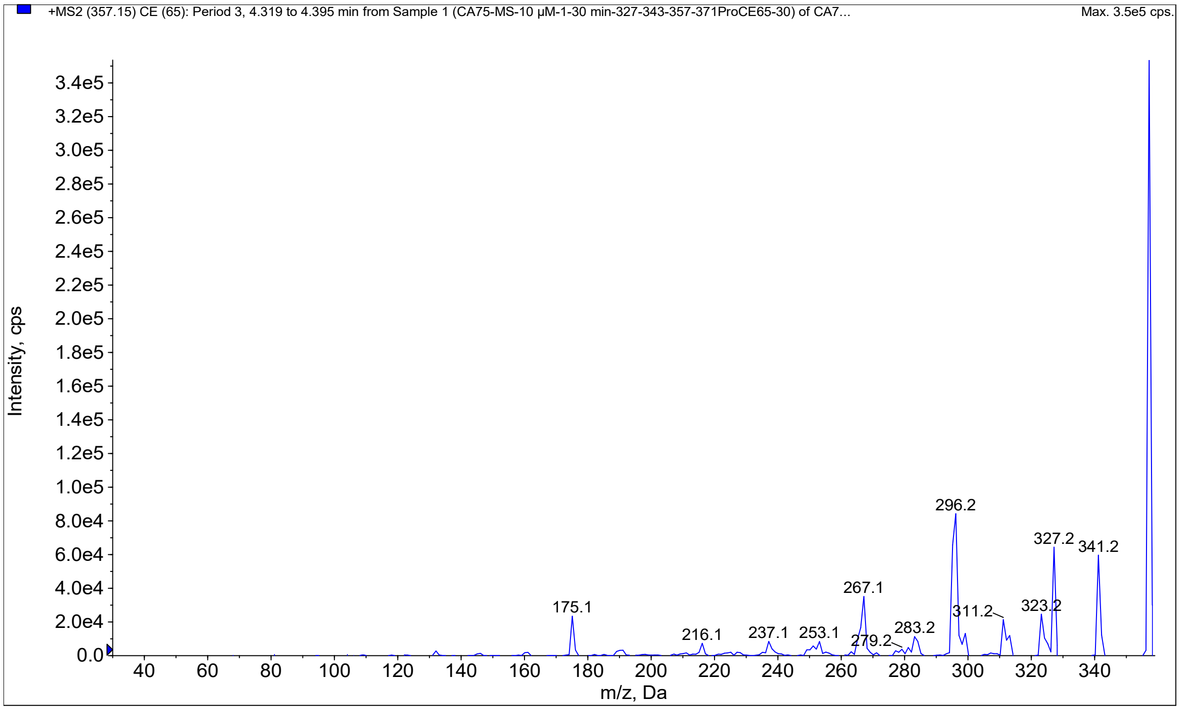

Figure S23. Product ion spectrum for ion 357 (m/z) derived from a peak at 4.31 min in the total ion current chromatogram from product ion scan of ion 357 (m/z) from study sample **8**-10µM-MS-1.

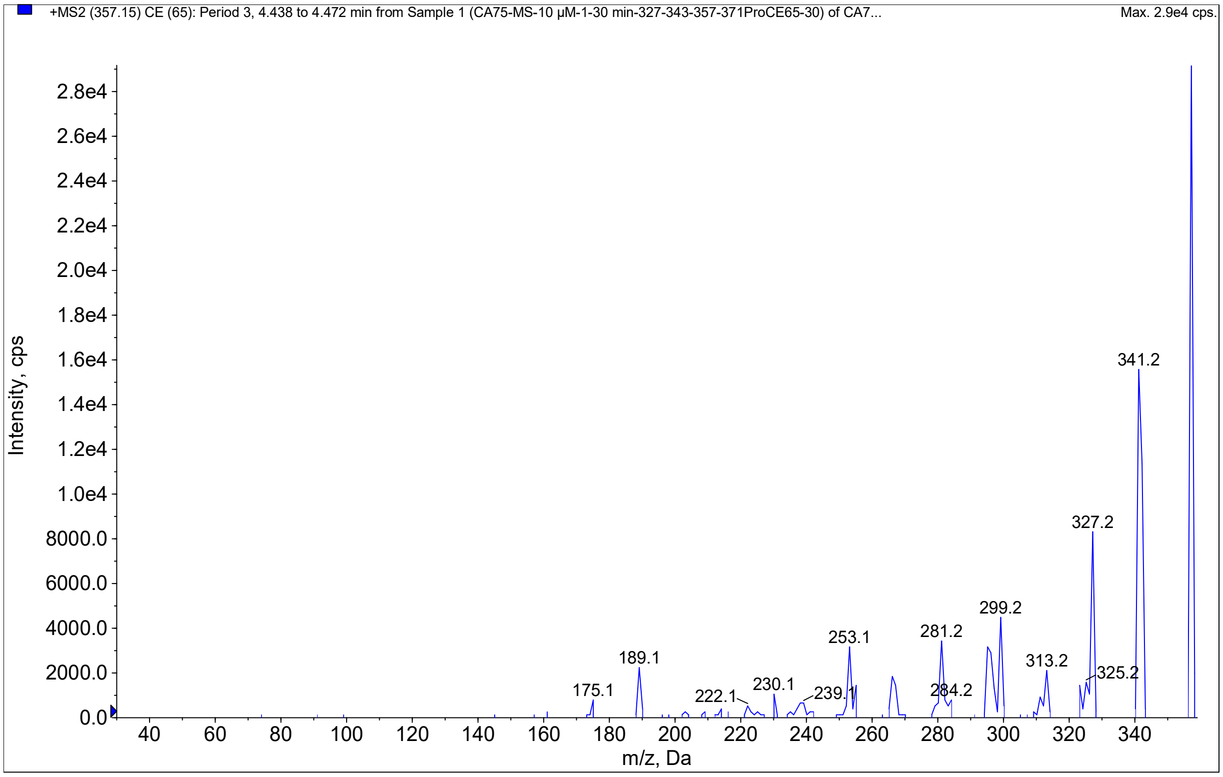

Figure S24. Product ion spectrum for ion 357 (m/z) derived from a peak at 4.40 min in the total ion current chromatogram from product ion scan of ion 357 (m/z) from study sample **8**-10µM-MS-1.

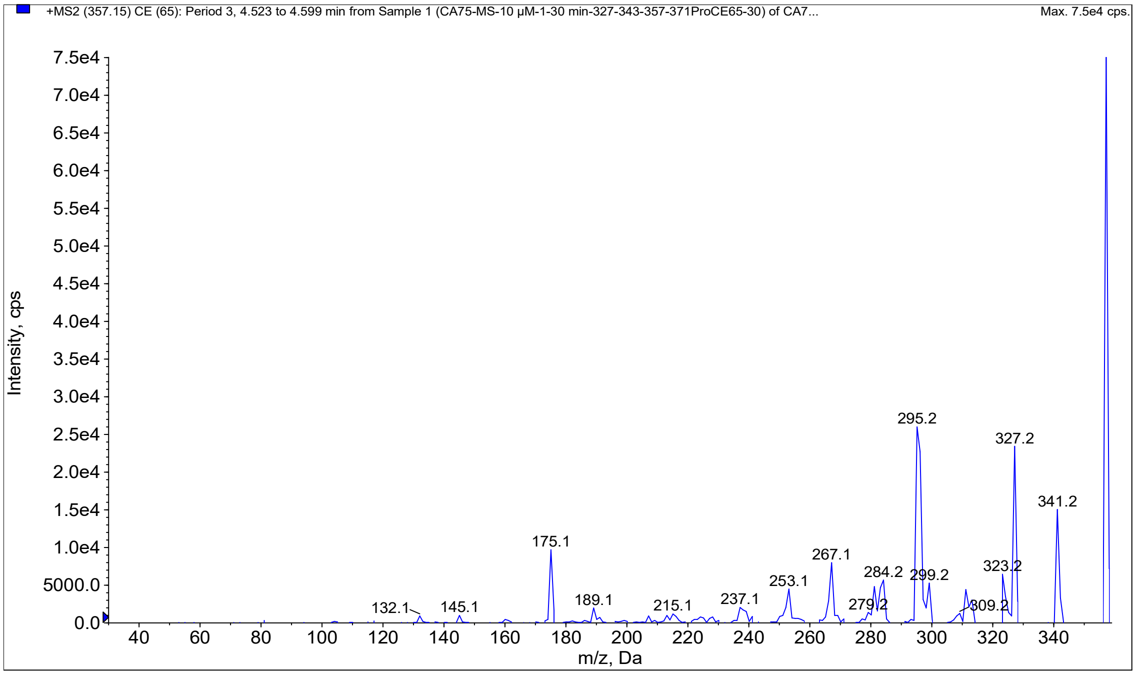

Figure S25. Product ion spectrum for ion 357 (m/z) derived from a peak at 4.50 min in the total ion current chromatogram from product ion scan of ion 357 (m/z) from study sample **8**-10µM-MS-1.

4. Metabolite Profiling of 9

4.1. Metabolite ID of 9 in Mouse Liver Microsomal Incubation

As shown in **Figure S26** and **S27**, **9** appeared at 2.70 min with mass to charge ratios of 343 and 345 (m/z), while the metabolite was eluted at 2.51 min with mass to charge ratios of 359 and 361 (m/z). This indicates that the metabolite is an oxidation product of **9**. The product ion spectra of both isotopes of **9** are shown in **Figure S28** and **S29**, respectively. The product ion spectra of both isotopes of the **9** oxidation metabolites are shown in **Figure S30** and **S31**, respectively.

4.2. Metabolite Profiling Graphs and Tables for 9

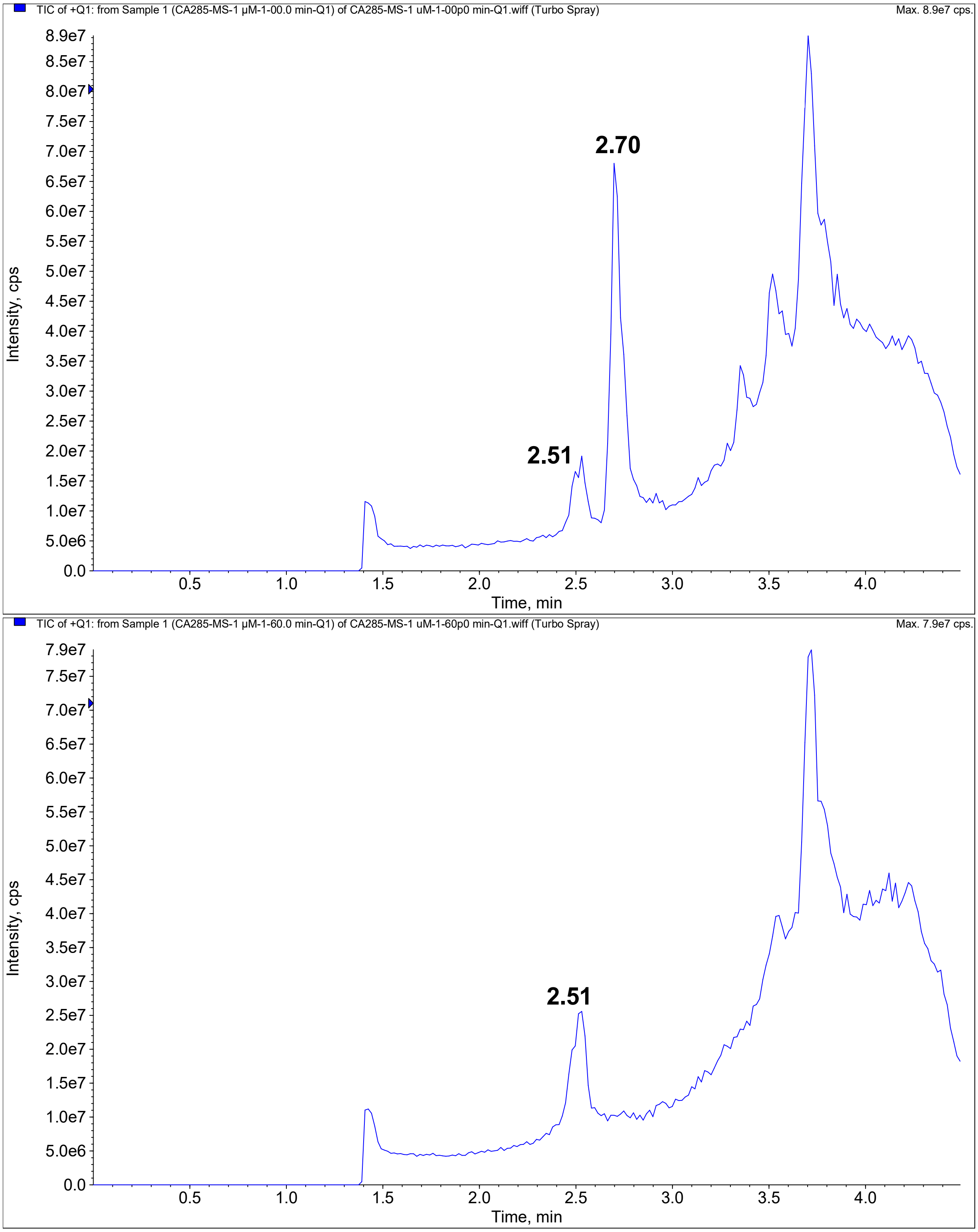

Figure S26. Total ion chromatograms of mouse liver microsomal incubation of **9** collected at 0 min (top) and 60 min (bottom) (ESI positive, Q1 scan).

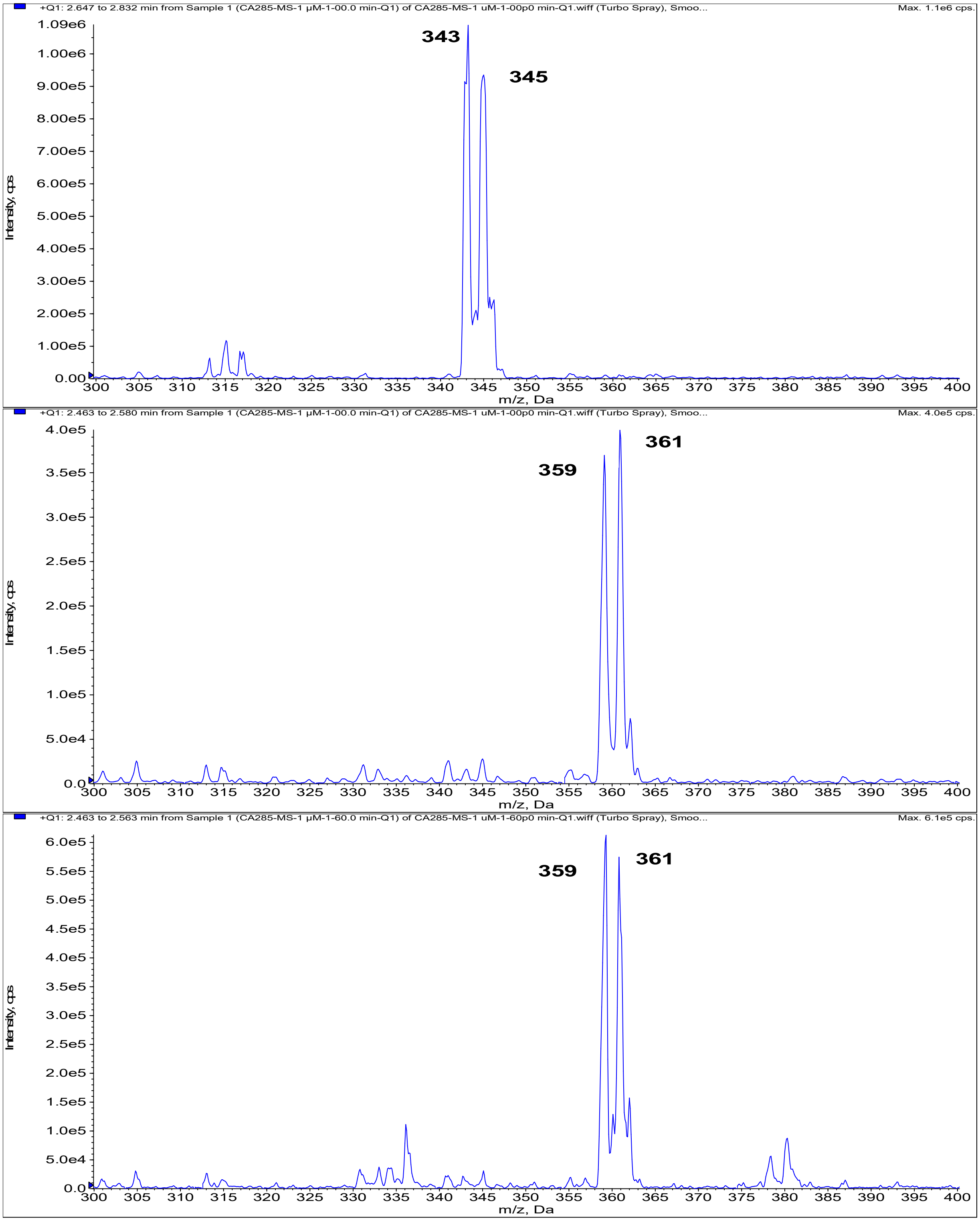

Figure S27. Mass spectra of the peak at 2.70 min (top) and 2.51 min (middle) in the chromatogram of **9**-0 min incubation and the peak at 2.51 min (bottom) in the chromatogram of **9**-60 min incubation.

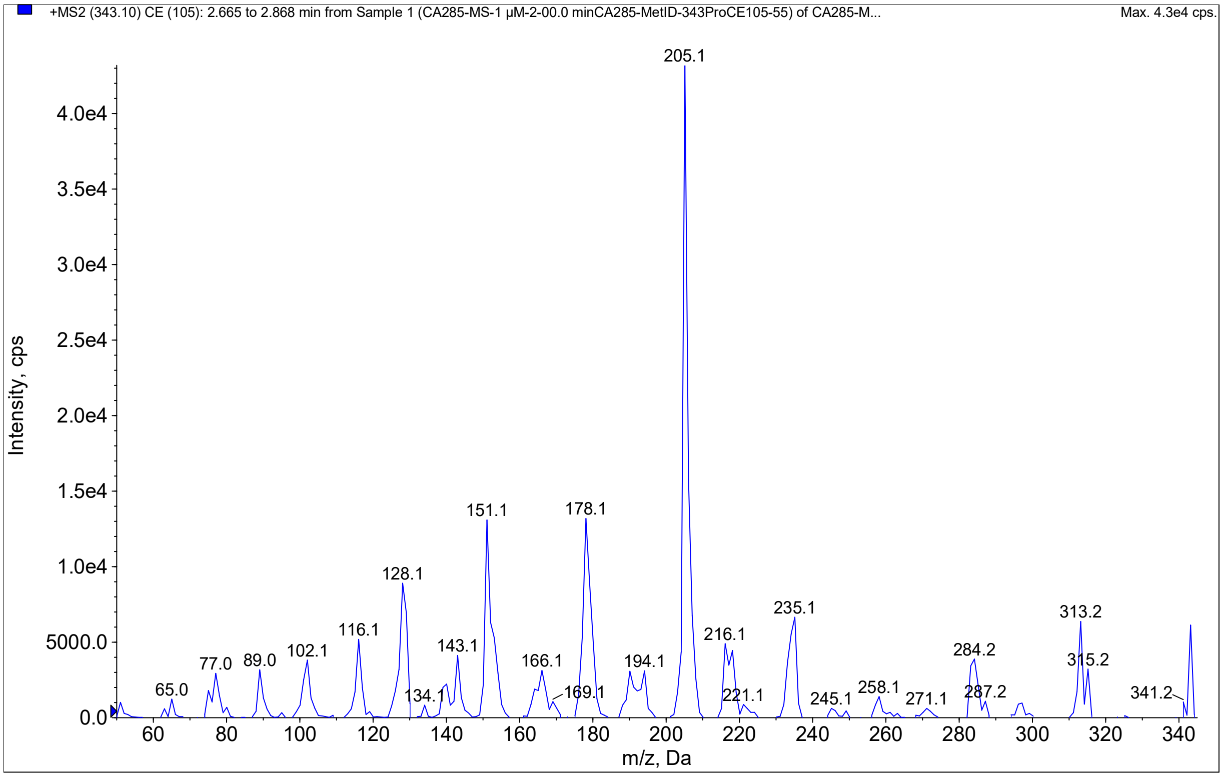

Figure S28. Product ion spectrum of ion 343 (m/z) at 2.70 min from injection **9**-0 min incubation.

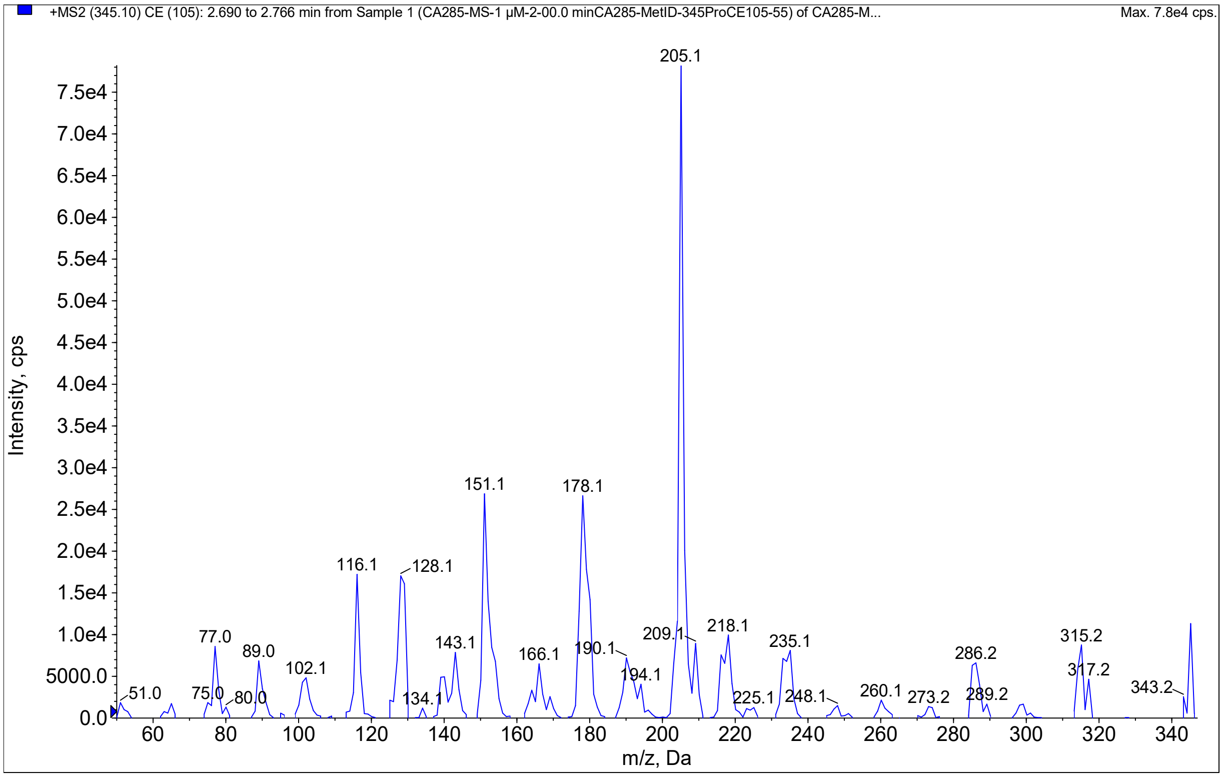

Figure S29. Product ion spectrum of ion 345 (m/z) at 2.70 min from injection **9**-0 min incubation.

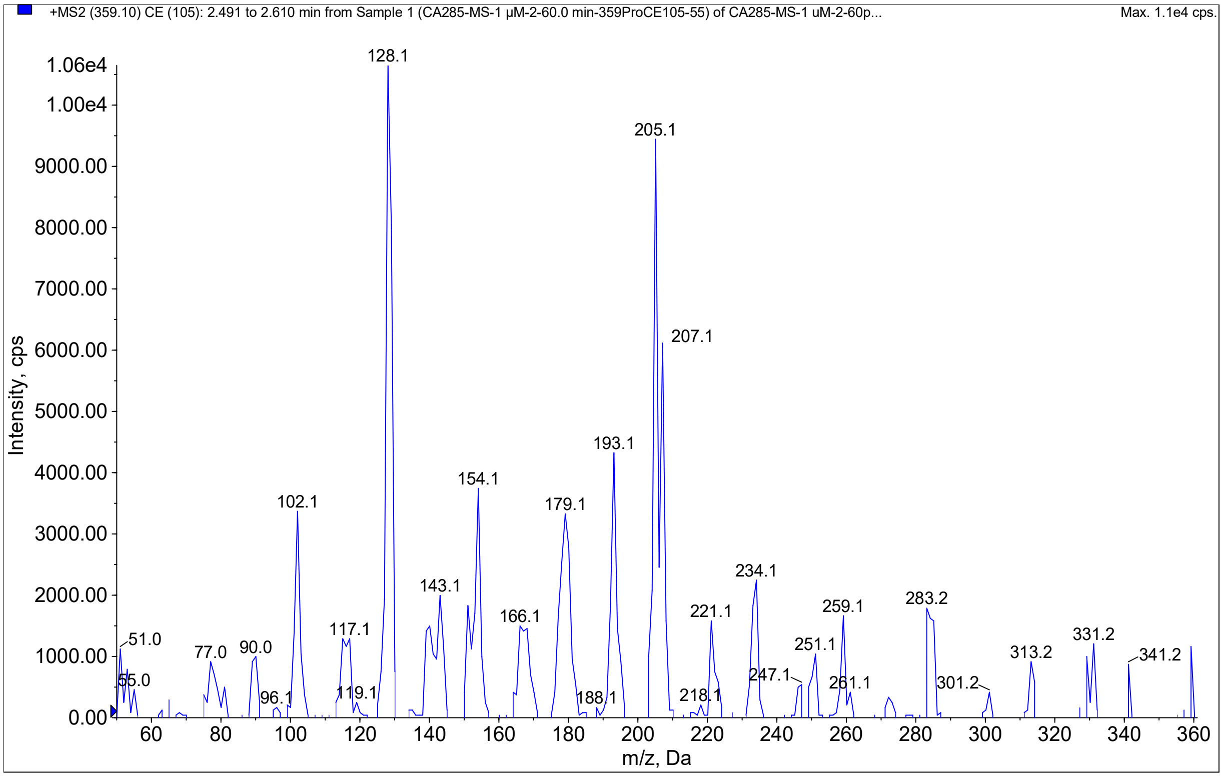

Figure S30. Product ion spectrum of ion 359 (m/z) at 2.51 min from injection **9**-60 min incubation.

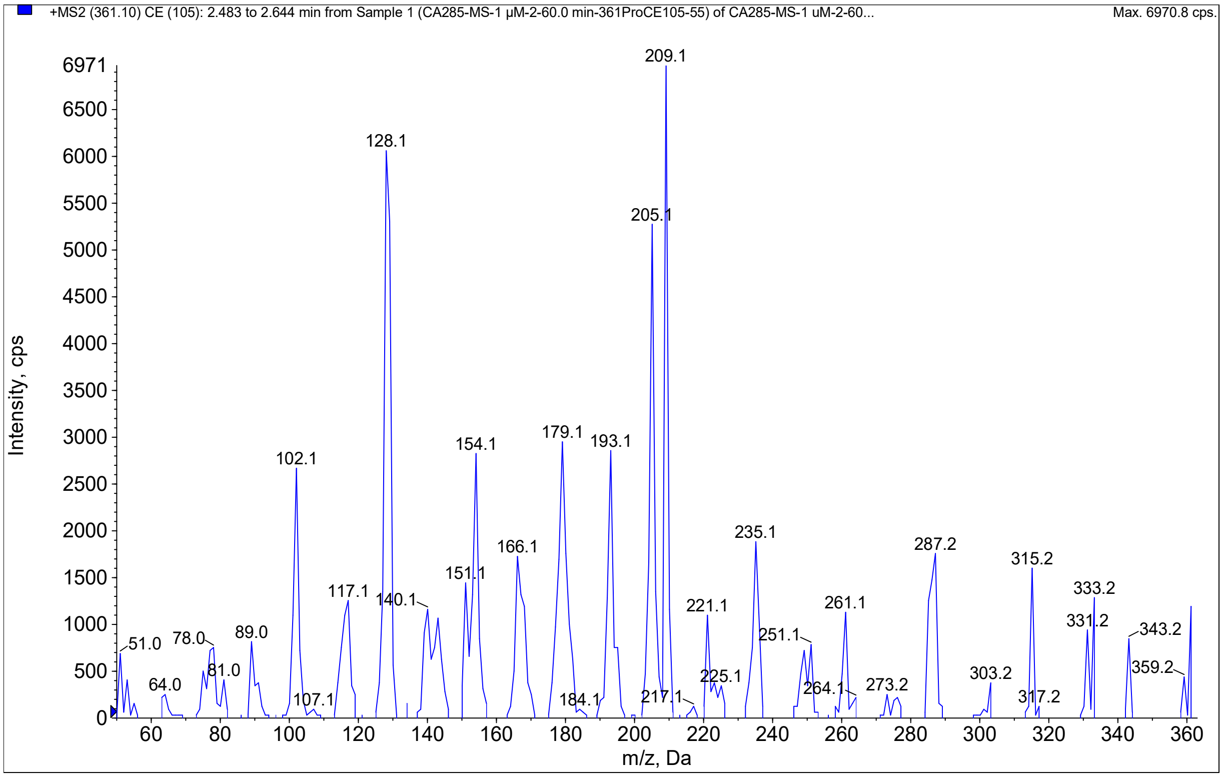

Figure S31. Product ion spectrum of ion 361 (m/z) at 2.51 min from injection **9**-60 min incubation.

5. Metabolite Profiling of 10

5.1. Product Ion Spectrum of 10 and Assignment of 10 Fragments

The total ion current chromatogram from product ion scan of ion 335.2 (m/z) from 5 µL injection of 10 µM of 10 incubated in mouse liver microsomes for 5 minutes is shown in **Figure S32**. The product ion spectrum derived from the peak in **Figure S32** is shown in **Figure S33**. The assignment of fragments is shown in **Figure S34**.

5.2. Identification of Metabolites in Mouse Liver Microsomal Incubation

5.2.1. Identification of M1 at 4.72 min, M2 at 8.47 min, and M3 at 8.70 min.

The components showed a quasi-molecular ion (MH) at m/z 351, a +16 dalton change relative to **10**. Extract ion (m/z 351) chromatograms, derived from the total ion chromatograms with Q1 scan from vehicle control sample Vehicle-MS-1 and study sample **10**-10µM-MS-1, are shown in **Figure S35**. There were peaks at 4.72, 8.47, and 8.70 min, respectively, in the chromatogram for the study sample. These peaks were not observed in the chromatogram for the vehicle control sample. The +16 dalton corresponds to the mass change for the oxidation of **10**.

The product ion spectrum for M1 is shown in **Figure S36**. Ions 128, 143, and 207 (m/z) were observed in the product ion spectrum for M1, indicating that oxidation most likely occurred in the part other than the quinoline ring structure. A structure was proposed for M1 as shown in **Table S5**. The presence of product ions 204 and 223 (m/z), which were not observed in the product ion spectrum of **10**, is still to be explained.

The product ion spectrum for M2 is shown in **Figure S37**. Ions 128 and 207 (m/z) were not observed in the product ion spectrum for M2. The most abundant product ion is ion 223 (m/z), indicating that oxidation most likely occurred in the quinoline ring structure. A structure consistent with the product ion spectrum was proposed for M2 as shown in **Table S5**.

The product ion spectrum for M3 is shown in **Figure S38**. Ions 128 and 207 (m/z) were much less abundant in the product ion spectrum for M3 than in that for **10**, indicating that oxidation most likely occurred in the quinoline ring structure. A structure consistent with the product ion spectrum was proposed for M3 as shown in **Table S5**.

5.2.2. Peak Areas of Metabolites and 10

The peak areas of metabolites and **10** are listed in **Table S6**. The metabolite with the most intensive signal is M1, from the oxidation of **10**. The metabolite with the most intensive mass spectrometric signal was formed the oxidation of **10**.

5.3. Metabolite Profiling Graphs and Tables for 10

Table S5. Metabolites of **10** (CA378) Observed in Mouse Liver Microsomal Incubation

Metabolite	Retention (min)	Mass to Charge Ratio (m/z) in Postive Ion Mode	Presence of Product Ion (Yes/No)				Tentatively Proposed Structure
			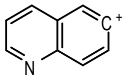 Exact Mass: 128.05	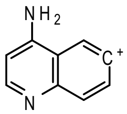 Exact Mass: 143.06	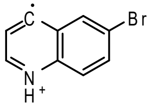 Exact Mass: 206.97	Extra Ions (m/z)	
**M1**	4.72	351.2	Yes	Yes	Yes	204 and 223	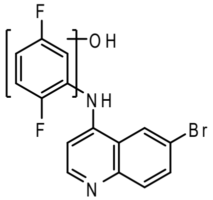
**M2**	8.47	351.2	No	Yes	No (223 = 207 + 16 (O); very strong signal)	223, 242, and 243	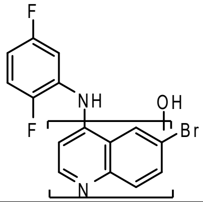
**M3**	8.70	351.2	Weak	Yes	Weak (223 = 207 + 16 (O); very strong signal)	223	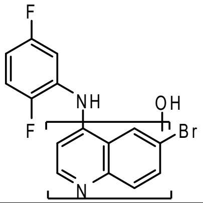
CA378	5.33	335.2	Yes	Yes	Yes		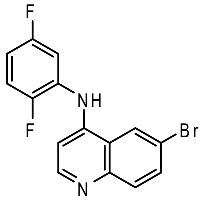

Table S6. Peak Area and Percent Peak Area of Metabolites of **10** (CA378) Observed in Mouse Liver Microsomal Incubation

Metabolite	Retention (min)	Mass to Charge Ratio (m/z)	Peak Area in Liver Microsomes	% Peak Area (over CT10258)	Rank in Peak Area Among Metabolites
			Mouse	Mouse	Mouse
**M1**	4.72	351.2	1.65E+07	113.8	1
**M2**	8.47	351.2	7.56E+05	5.2	2
**M3**	8.70	351.2	3.68E+05	2.5	3
**CA378**	5.33	335.2	1.45E+07	100.0	N/A

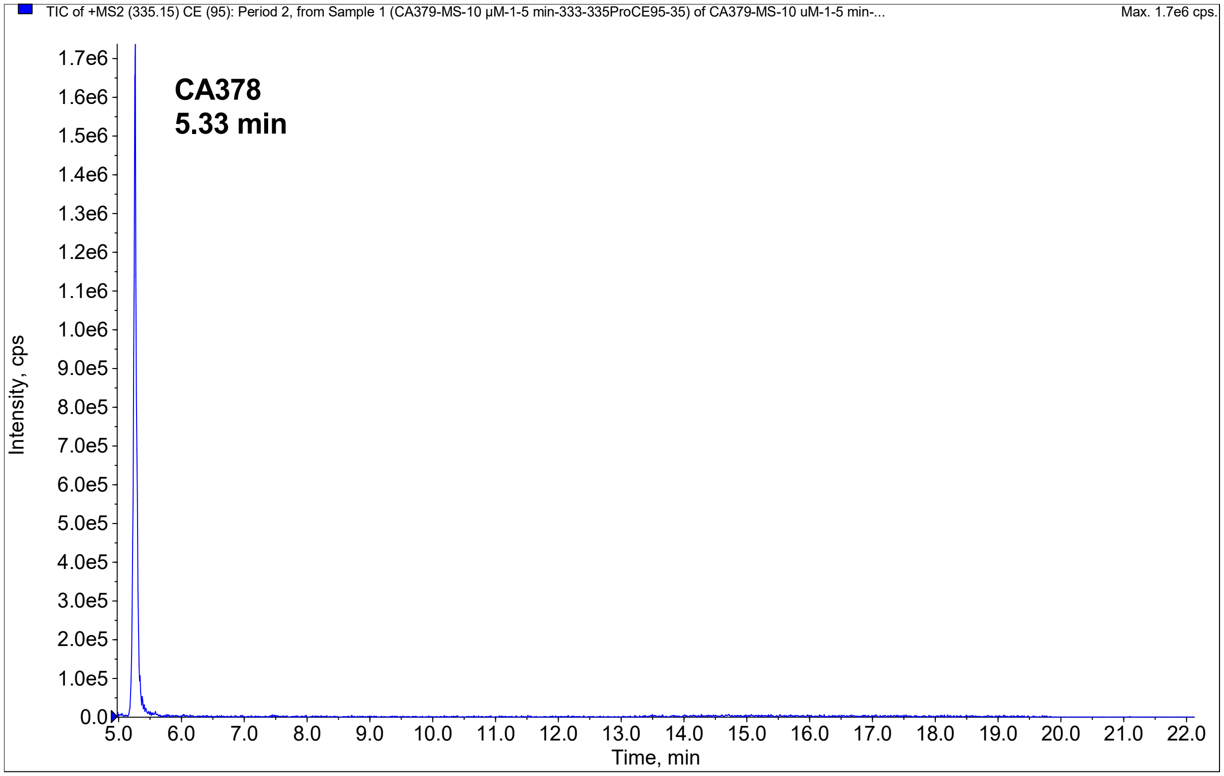

Figure S32. Total ion current chromatogram from product ion scan of ion 335.2 (m/z) from 5 µL injection of 10 µM of **10** (CA378) incubated in mouse liver microsomes for 5 minutes.

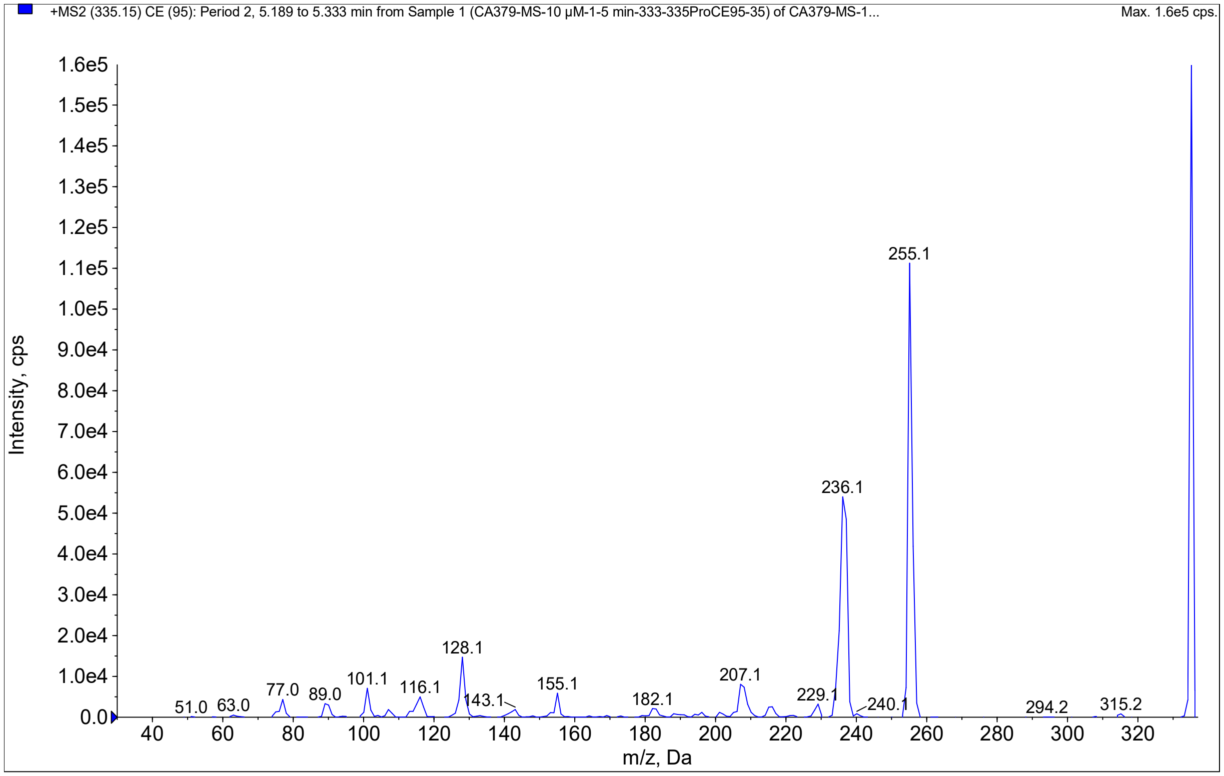

Figure S33. Product ion spectrum for ion 335.2 (m/z) derived from the peak at 5.33 min in **Figure S32**.

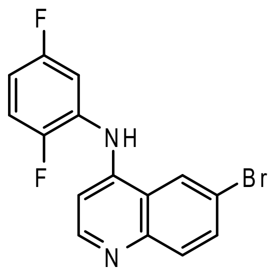

Chemical Formula: $C_{15}H_9BrF_2N_2$
Exact Mass: 333.99
CA378

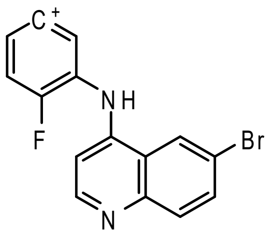

Exact Mass: 314.99

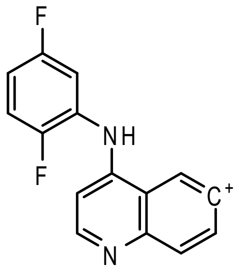

Exact Mass: 255.07

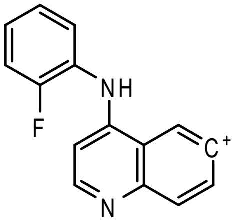

Exact Mass: 237.08

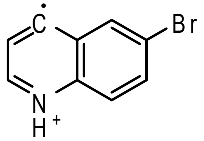

Exact Mass: 206.97

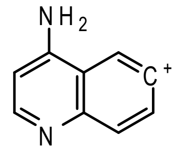

Exact Mass: 143.06

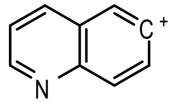

Exact Mass: 128.05

Figure S34. Assignment of fragments in **10** (CA378) product ion spectrum.

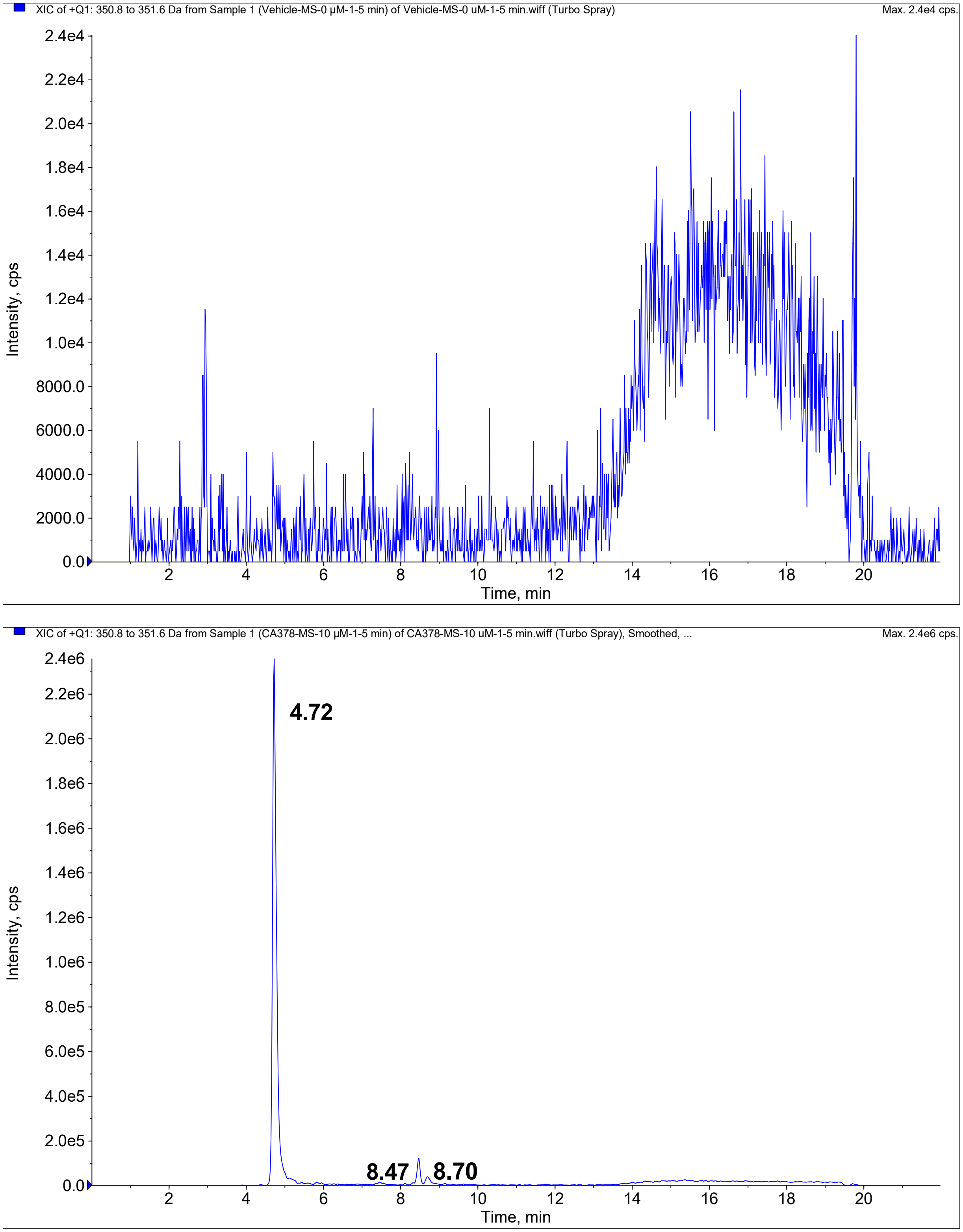

Figure S35. Extract ion (m/z 351) chromatograms from vehicle control sample Vehicle-MS-1 (top) and study sample **10**-10µM-MS-1 (bottom).

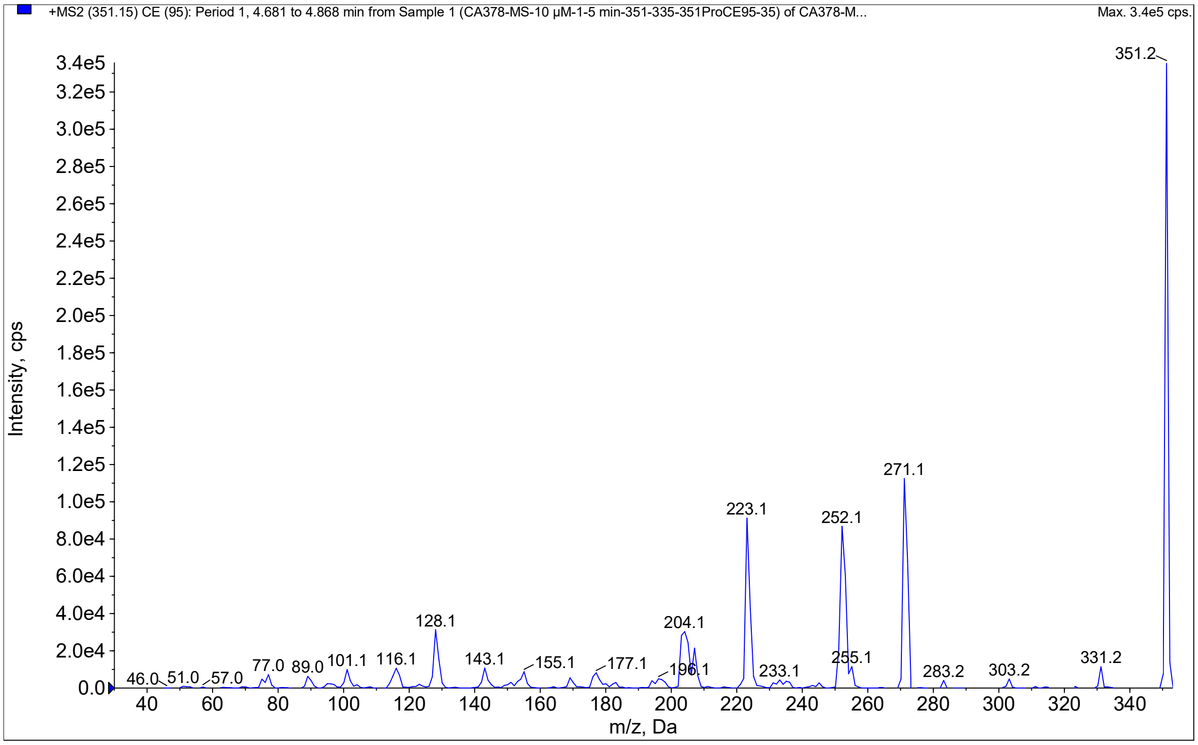

Figure S36. Product ion spectrum for ion 351 (m/z) derived from a peak at 4.72 min in the total ion current chromatogram from product ion scan of ion 351 (m/z) from study sample **10**-10µM-MS-1.

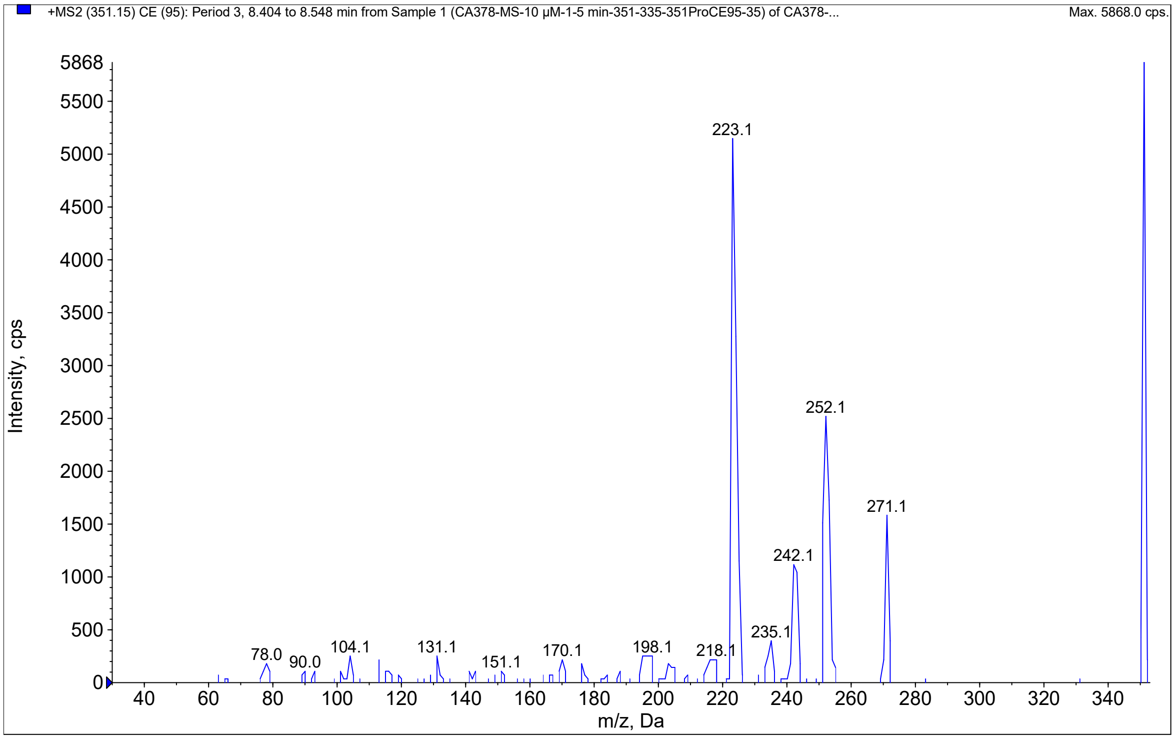

Figure S37. Product ion spectrum for ion 351 (m/z) derived from a peak at 8.47 min in the total ion current chromatogram from product ion scan of ion 351 (m/z) from study sample **10**-10µM-MS-1.

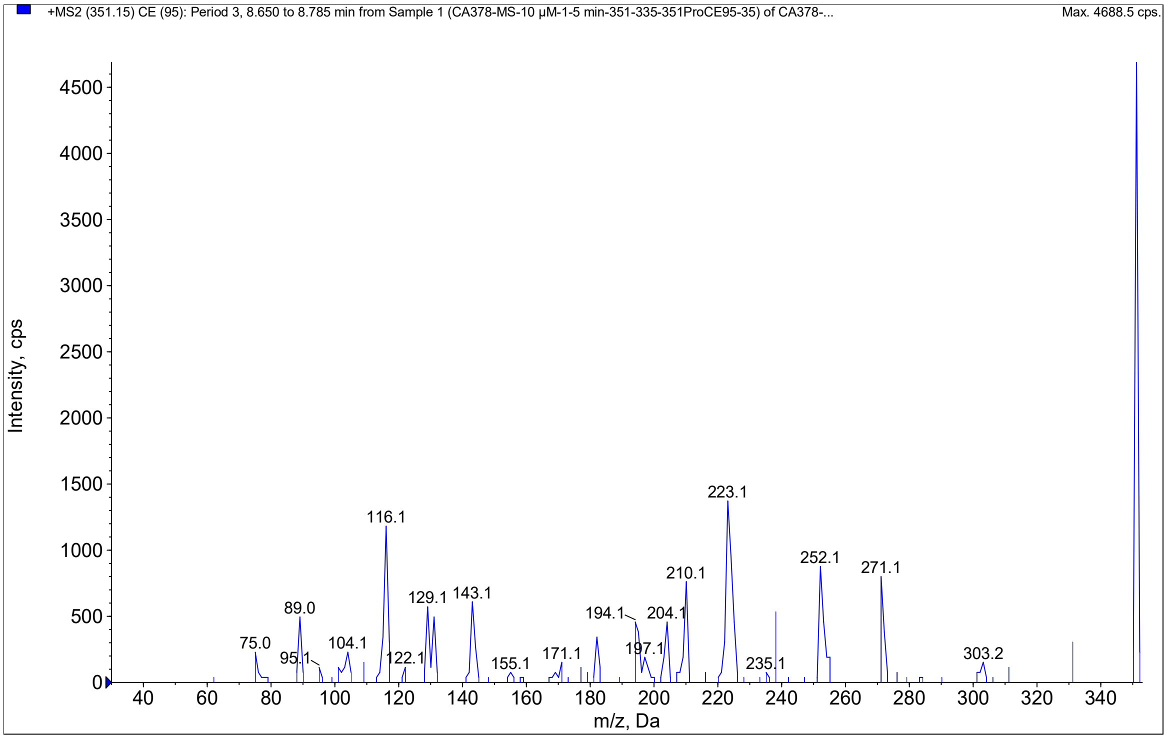

Figure S38. Product ion spectrum for ion 351 (m/z) derived from a peak at 8.70 min in the total ion current chromatogram from product ion scan of ion 351 (m/z) from study sample **10**-10μM-MS-1.

6. Metabolite Profiling of 11

6.1. Product Ion Spectrum of 11 and Assignment of 11 Fragments

The total ion current chromatogram from product ion scan of ion 335.2 (m/z) from 5 µL injection of **11** µM of 11 incubated in mouse liver microsomes for 5 minutes is shown in **Figure S39**. The product ion spectrum derived from the peak in **Figure S39** is shown in **Figure S40**. The assignment of fragments is shown in **Figure S41**.

6.2. Identification of Metabolites in Mouse Liver Microsomal Incubation

6.2.1. Identification of M1 at 4.68 min.

The component showed a quasi-molecular ion (MH^+) at m/z 333, a -2 dalton change relative to **11**. Extract ion (m/z 333) chromatograms, derived from the total ion chromatograms with Q1 scan from vehicle control sample Vehicle-MS-1 and study sample **11**-10µM-MS-1, are shown in **Figure S42**. There was a peak at 4.68 min in the chromatogram for the study sample. This peak was not observed in the chromatogram for the vehicle control sample.

The -2 dalton corresponds to the mass change of substituting one fluorine atom with a hydroxyl group. The product ion spectrum for M1 is shown in **Figure S43**. Product ion 207 (m/z) was abundant in the product ion spectrum for M1. A structure consistent with the product ion spectrum was proposed for M1 as shown in **Table S7**.

6.2.2. Identification of M2 at 4.77 min, M3 at 5.06 min, M4 at 7.49 min, and M5 at 7.90 min.

The components showed a quasi-molecular ion (MH) at m/z 351, a +16 dalton change relative to **11**. Extract ion (m/z 351) chromatograms, derived from the total ion chromatograms with Q1 scan from vehicle control sample Vehicle-MS-1 and study sample **11**-10µM-MS-1, are shown in **Figure S44**. There were peaks at 4.77, 5.06, 7.49, and 7.90 min, respectively, in the chromatogram for the study sample. These peaks were not observed in the chromatogram for the vehicle control sample. The +16 dalton corresponds to the mass change for the oxidation of **11**. The product ion spectra for M2, M3, M4, and M5 are shown in **Figure S45-48**, respectively. Structures consistent with the product ion spectra are to be proposed.

6.3. Peak Areas of Metabolites and 11

The peak areas of metabolites and **11** are listed in **Table S8**. The metabolite with the most intensive signal is M1, from the substitution of one fluorine atom with a hydroxyl group in **11**. The metabolite with the most intensive mass spectrometric signal was formed through the substitution of one fluorine atom with a hydroxyl group in **11**.

6.4. Metabolite Profiling Graphs and Tables for 11

Table S7. Metabolites of **11** (CA379) Observed in Mouse Liver Microsomal Incubation Continued

Metabolite	Retention (min)	Mass to Charge Ratio (m/z) in Postive Ion Mode	Presence of Product Ion (Yes/No)					Proposed Structure
			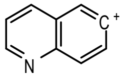 Exact Mass: 128.05	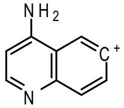 Exact Mass: 143.06	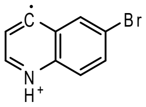 Exact Mass: 206.97	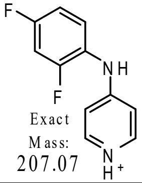 Exact Mass: 207.07	Extra Ions (m/z)	
**M1**	4.68	333.2	Yes	Yes	Yes	205 = 207 - 19 (F) + 17 (OH)		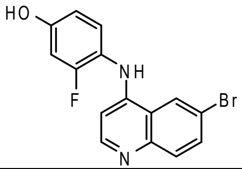
**M2**	4.77	351.2	Yes	No	No	No (223 = 207 + 16 (O))		Oxidation metabolite; to propose structure
**M3**	5.06	351.2	Yes	Yes	Yes	223 = 207 + 16 (O)		Oxidation metabolite; to propose structure
**M4**	7.49	3512	No	Weak	No	No ions 207 and 223 (m/z)	334 = 351 - 17 (NH3)	Oxidation metabolite; to propose structure
**M5**	7.90	351.2	Weak	Yes	Weak	223 = 207 + 16 (O)		Oxidation metabolite; to propose structure
CA379	5.23	335.2	Yes	Yes	Yes	Yes		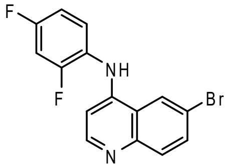

Table S8. Peak Area and Percent Peak Area of Metabolites of **11** (CA379) Observed in Mouse Liver Microsomal Incubation

Metabolite	Retention (min)	Mass to Charge Ratio (m/z)	Peak Area in Liver Microsomes	% Peak Area (over CT10258)	Rank in Peak Area Among Metabolites
			Mouse	Mouse	Mouse
**M1**	4.68	333.2	5.82E+06	86.4	1
**M2**	4.77	351.2	1.50E+05	2.2	5
**M3**	5.06	351.2	7.27E+05	10.8	3
**M4**	7.49	3512	2.17E+05	3.2	4
**M5**	7.90	351.2	1.12E+06	16.6	2
**CA379**	5.23	335.2	6.74E+06	100.0	N/A

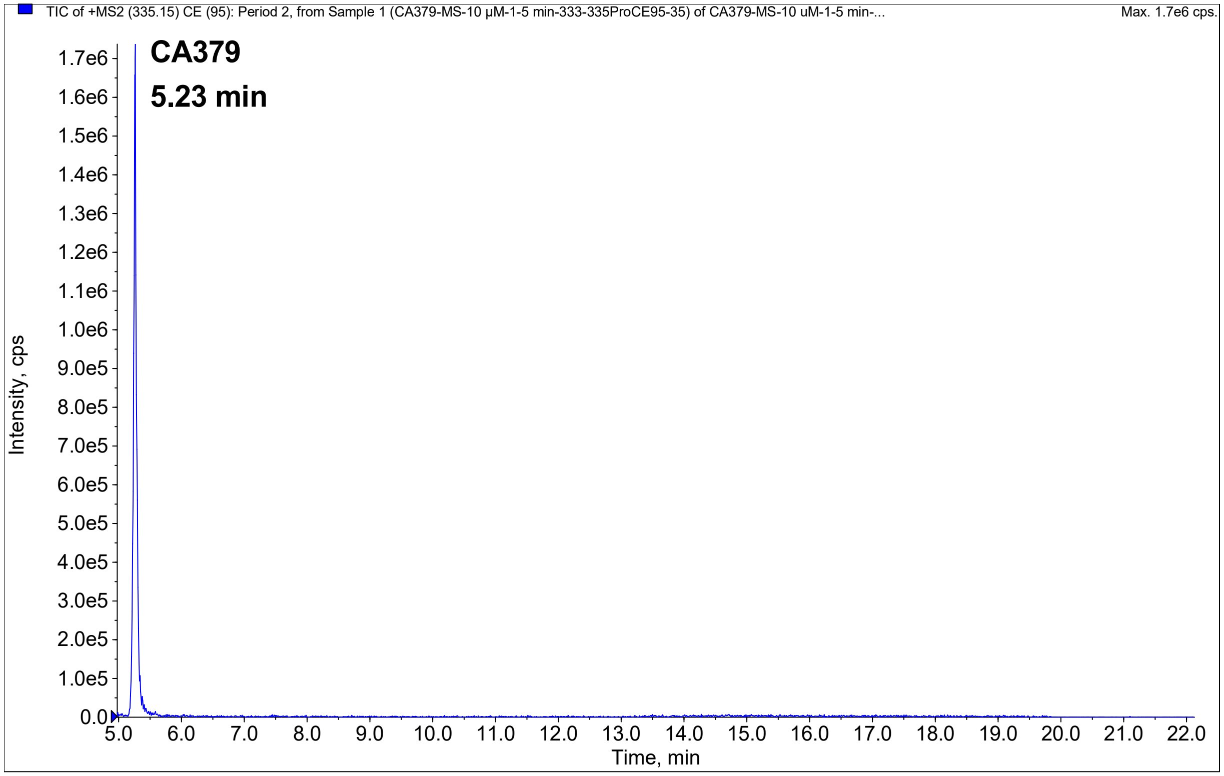

Figure S39. Total ion current chromatogram from product ion scan of ion 335.2 (m/z) from 5 µL injection of 10 µM **11** incubated in mouse liver microsomes for 5 minutes.

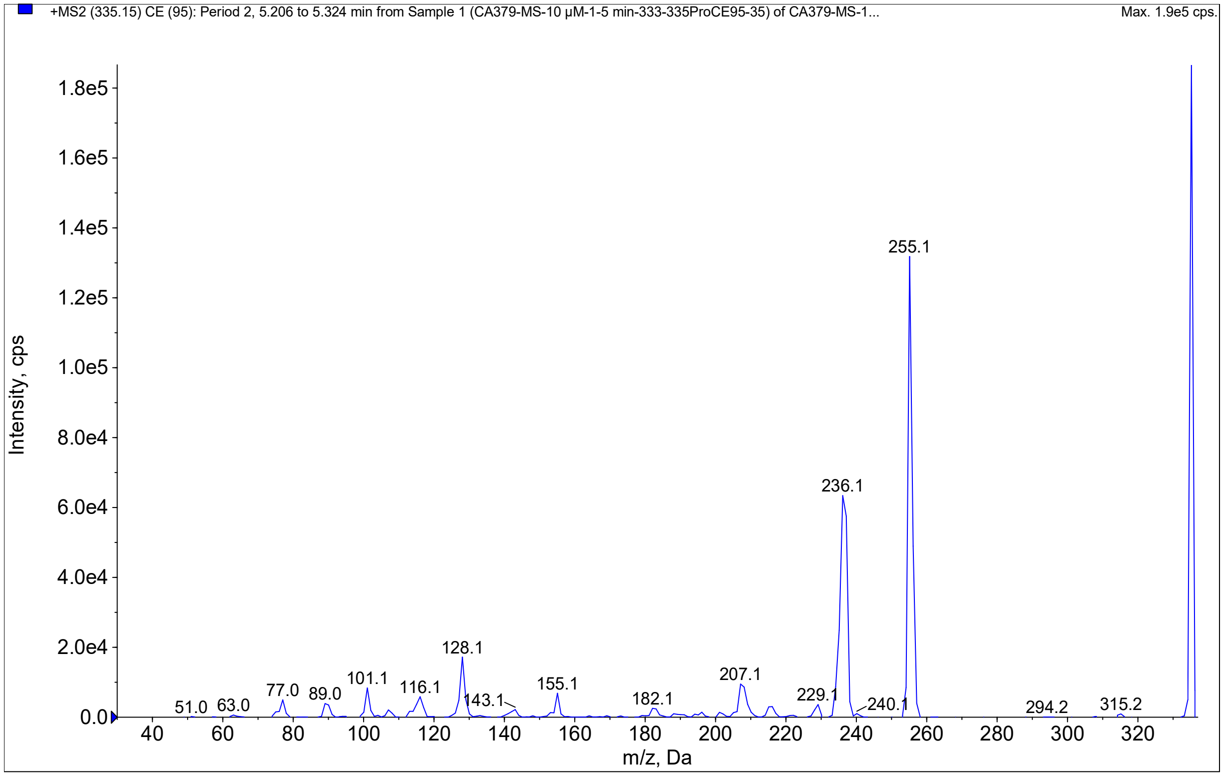

Figure S40. Product ion spectrum for ion 335.2 (m/z) derived from the peak at 5.23 min in **Figure S39**.

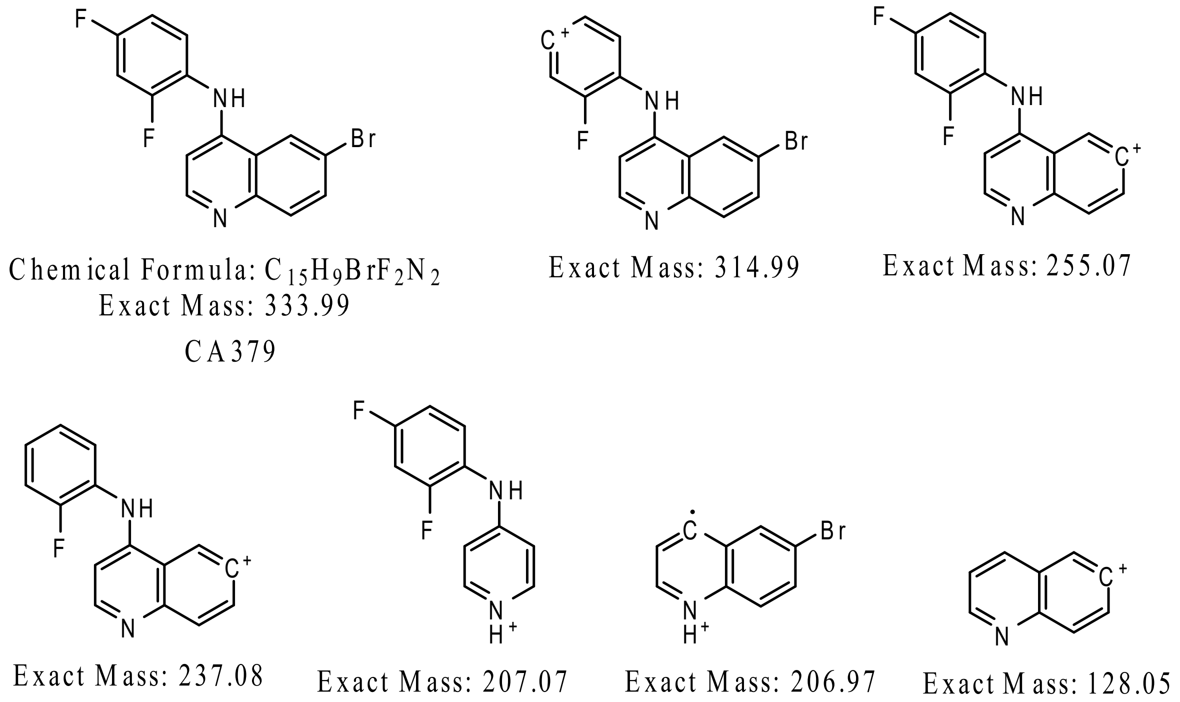

Figure S41. Assignment of fragments in **11** (CA379) product ion spectrum.

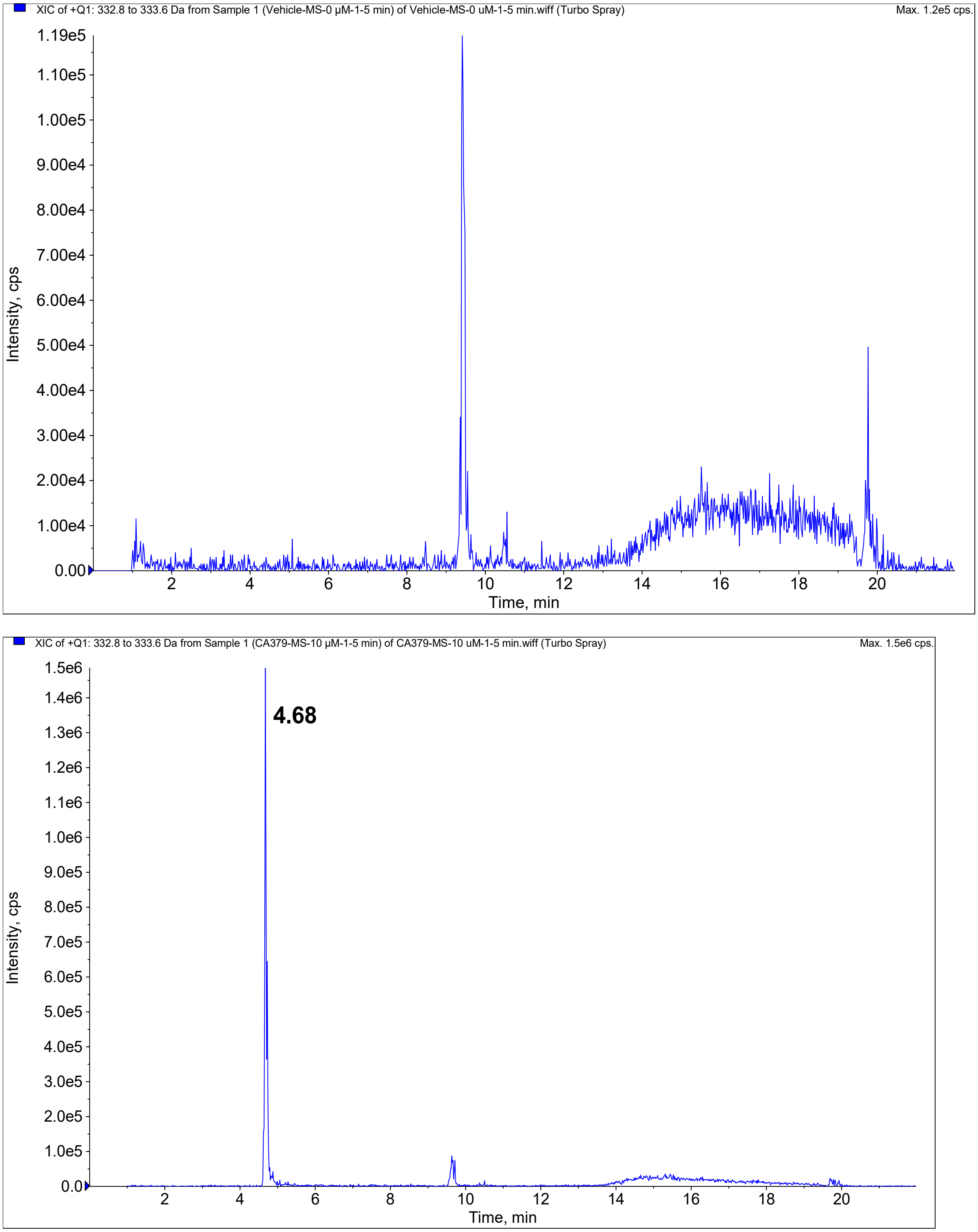

Figure S42. Extract ion (m/z 333) chromatograms from vehicle control sample Vehicle-MS-1 (top) and study sample **11**-10µM-MS-1 (bottom).

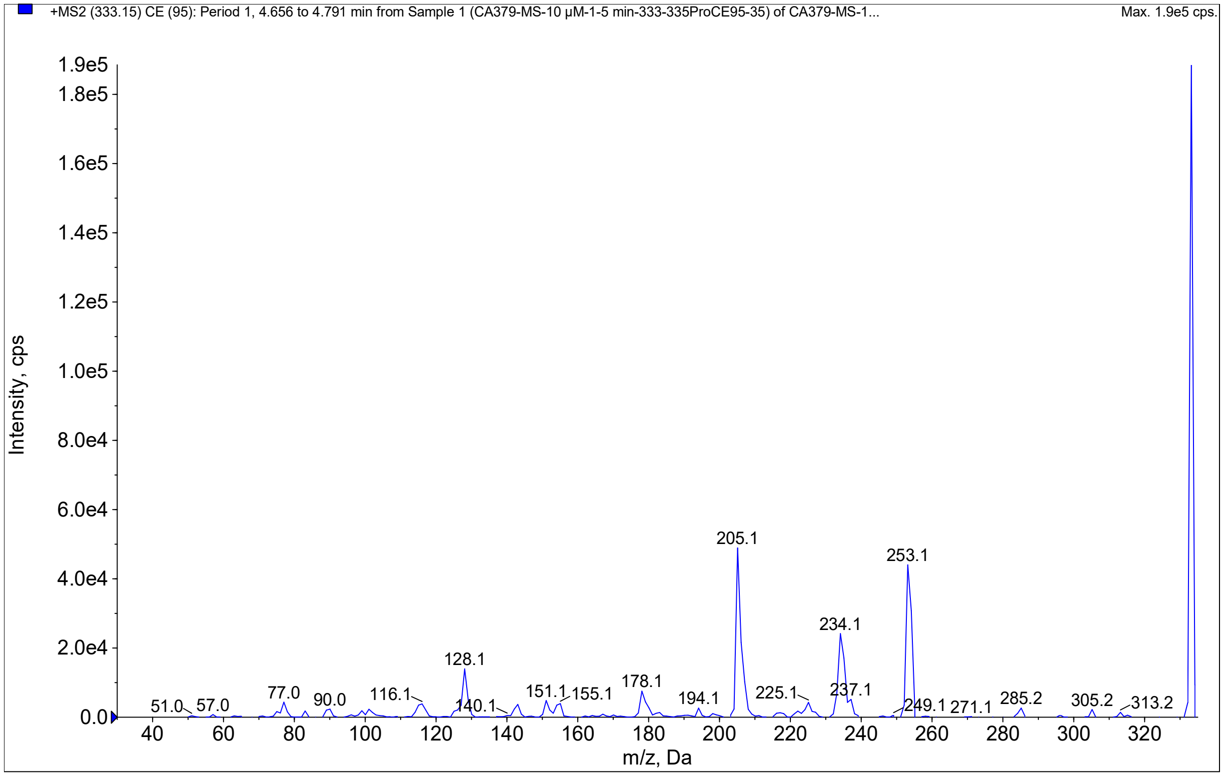

Figure S43. Product ion spectrum for ion 333 (m/z) derived from a peak at 4.68 min in the total ion current chromatogram from product ion scan of ion 333 (m/z) from study sample **11**-10µM-MS-1.

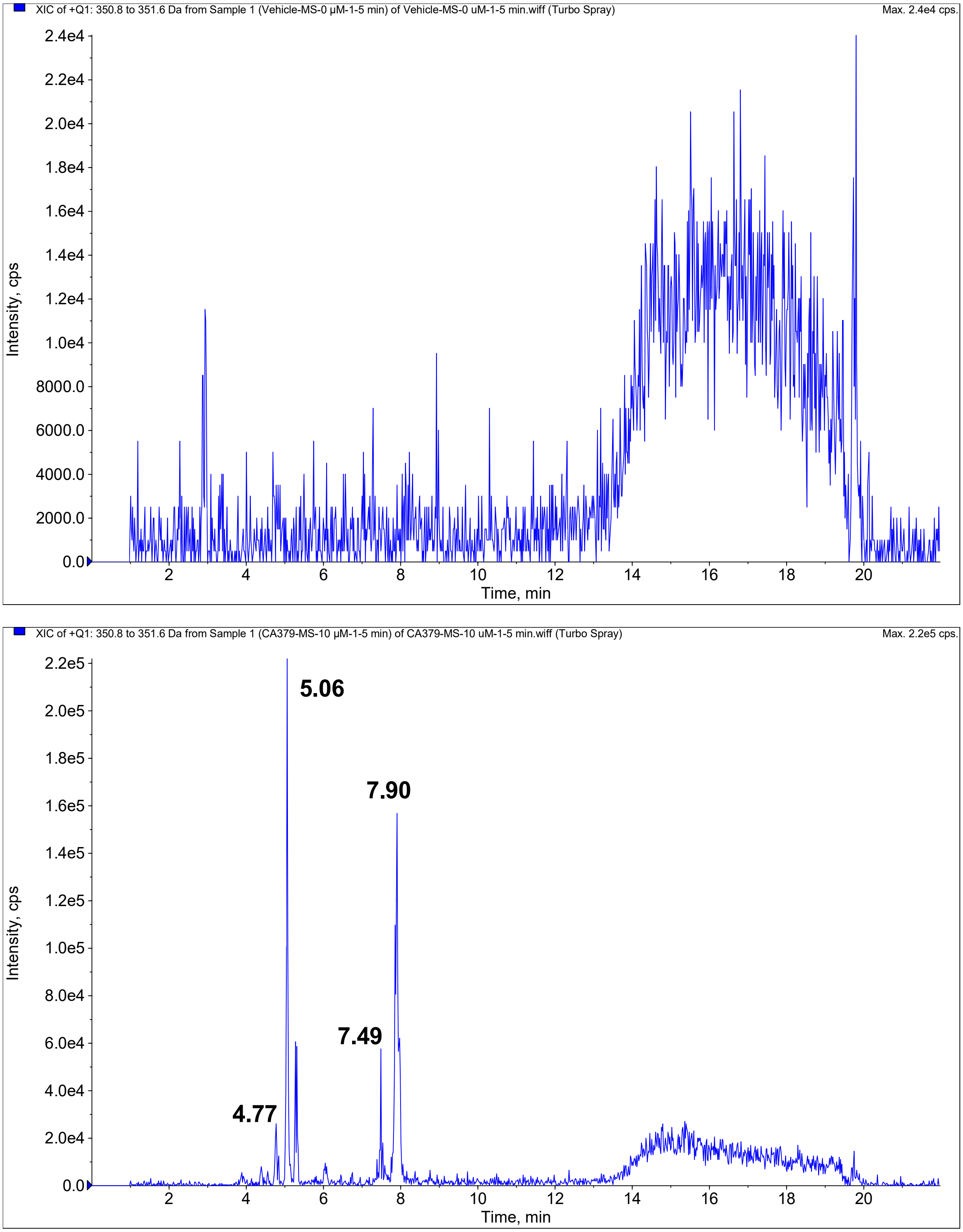

Figure S44. Extract ion (m/z 351) chromatograms from vehicle control sample Vehicle-MS-1 (top) and study sample **11**-10µM-MS-1 (bottom).

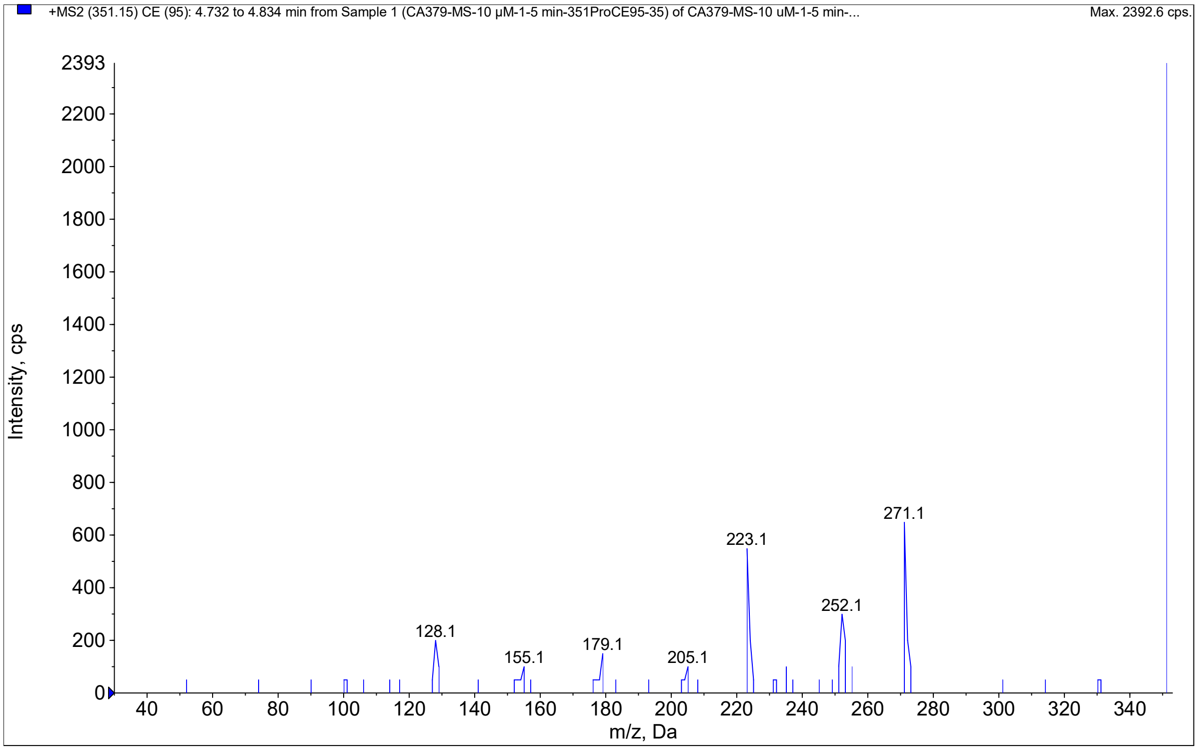

Figure S45. Product ion spectrum for ion 351 (m/z) derived from a peak at 4.77 min in the total ion current chromatogram from product ion scan of ion 351 (m/z) from study sample **11**-10µM-MS-1.

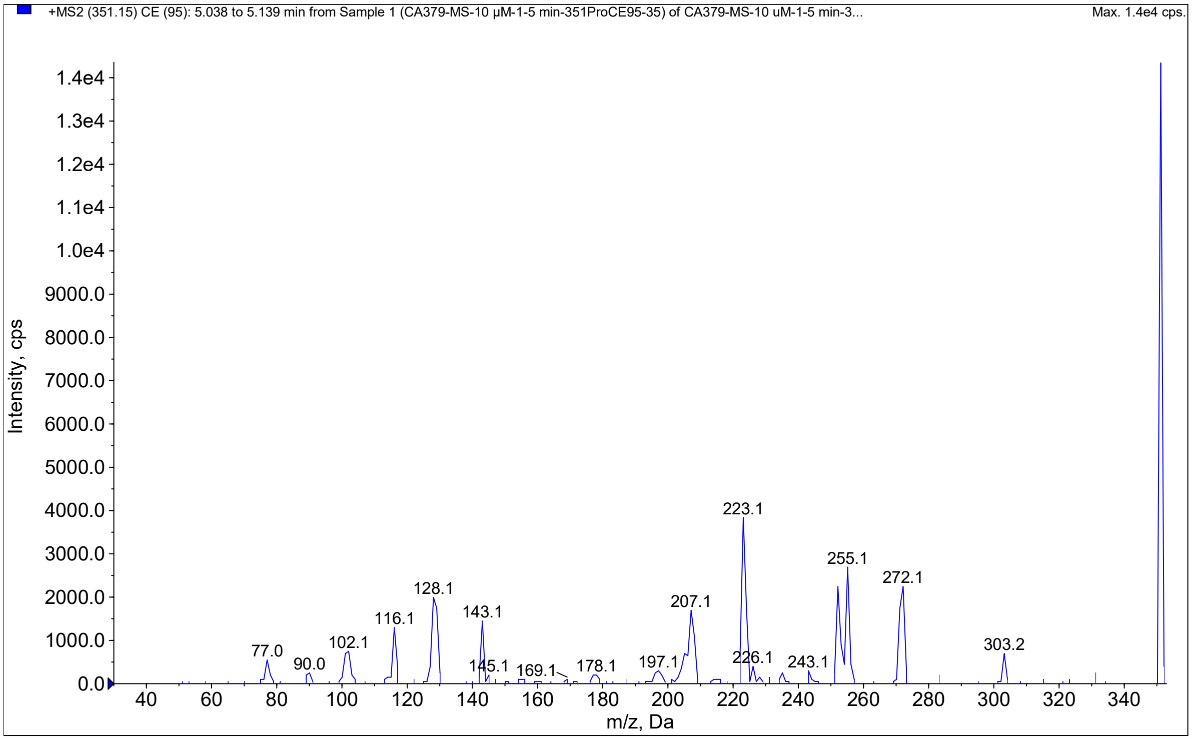

Figure S46. Product ion spectrum for ion 351 (m/z) derived from a peak at 5.06 min in the total ion current chromatogram from product ion scan of ion 351 (m/z) from study sample **11**-10µM-MS-1.

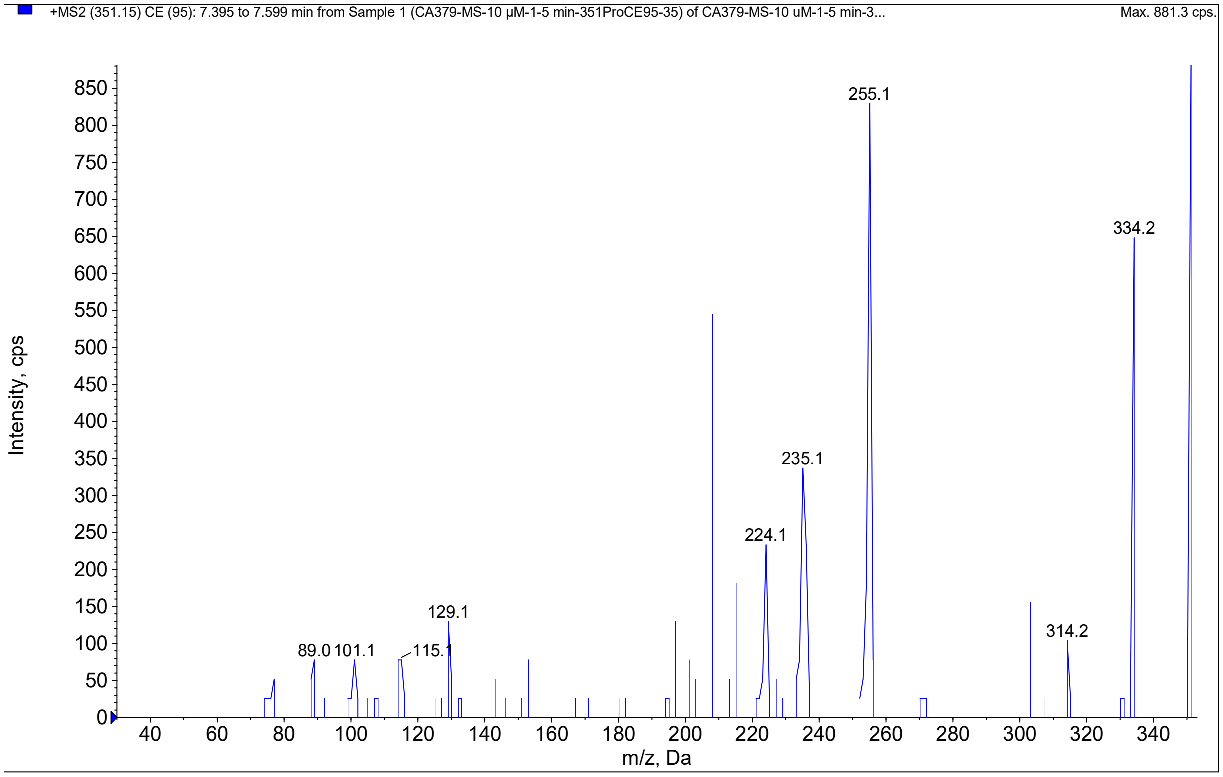

Figure S47. Product ion spectrum for ion 351 (m/z) derived from a peak at 7.49 min in the total ion current chromatogram from product ion scan of ion 351 (m/z) from study sample **11**-10µM-MS-1.

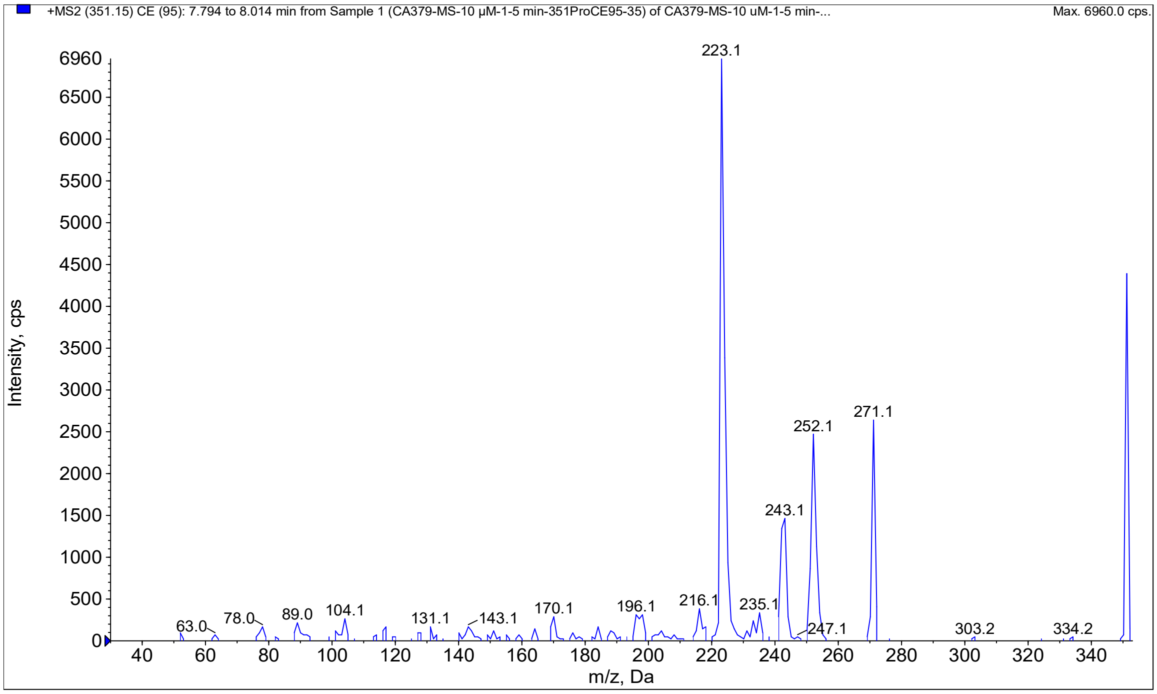

Figure S48. Product ion spectrum for ion 351 (m/z) derived from a peak at 7.90 min in the total ion current chromatogram from product ion scan of ion 351 (m/z) from study sample **11**-10µM-MS-1.

7. Metabolite Profiling of 35

7.1. Product Ion Spectrum of 35 and Assignment of 11 Fragments

The total ion current chromatogram from product ion scan of ion 339.2 (m/z) from 5 µL injection of 10 µM of **35** incubated in mouse liver microsomes for 30 minutes is shown in **Figure S49**. The product ion spectrum derived from the peak in **Figure S49** is shown in **Figure S50**. The assignment of fragments is shown in **Figure S51**.

7.2. Identification of Metabolites in Mouse Liver Microsomal Incubation

7.2.1. Identification of M1 at 3.69 min, M2 at 4.50 min, and M3 at 5.76 min.

The components showed a quasi-molecular ion (MH) at m/z 355, a +16 dalton change relative to **35**. Extract ion (m/z 355) chromatograms, derived from the total ion chromatograms with Q1 scan from vehicle control sample Vehicle-MS-1 and study sample **35**-10µM-MS-1, are shown in **Figure S51**. There were peaks at 3.69 min, 4.50 min, and 5.76 min, respectively, in the chromatogram for the study sample. These peaks were not observed in the chromatogram for the vehicle control sample. The +16 dalton corresponds to the mass change for the oxidation of **35**.

The product ion spectrum for M1 is shown in **Figure S52**. No product ion 311 (m/z) signal was observed in the product ion spectrum for M1. Instead, product ion 327 (m/z) was observed, indicating that oxidation didn't occur in the pyrazole ring structure. Product ion 143 (m/z) was still abundant in the product ion spectrum for M1, indicating the oxidation didn't occur in the quinoline ring structure. Product ion 89 (m/z) signal was weak in the product ion spectrum for M1. Instead, product ion 105 (m/z) was observed, indicating that the oxidation occurred in the group corresponding to fragment 89 (m/z). A structure consistent with the product ion spectrum was proposed for M1 as shown in **Table S9**.

The product ion spectrum for M2 is shown in **Figure S53**. No product ion 311 (m/z) signal was observed in the product ion spectrum for M2. Instead, product ion 327 (m/z) was observed, indicating that oxidation didn't occur in the pyrazole ring structure. Product ion 143 (m/z) was still abundant in the product ion spectrum for M2, indicating the oxidation didn't occur in the quinoline ring structure. Product ion 89 (m/z) signal was weak in the product ion spectrum for M2. Instead, product ion 105 (m/z) was observed, indicating that the oxidation occurred in the group corresponding to fragment 89 (m/z). A structure consistent with the product ion spectrum was proposed for M2 as shown in **Table S9**.

The product ion spectrum for M3 is shown in **Figure S54**. The most intensive product ion is 338 (m/z), which is from a neural loss of NH_3 from M3. For **35**, however, little product ion signal from a neutral loss of NH_3 was observed. The enabling of a neutral loss of NH_3 is most likely from the oxidation in the pyrazole ring structure in **35**. A structure consistent with the product ion spectrum was proposed for M3 as shown in **Table S9**.

7.3. Peak Areas of Metabolites and 35.

The peak areas of metabolites and **35** are listed in **Table S10**. The metabolite with the most intensive signal is M1, from the oxidation of benzene ring in the indazole ring structure. The major metabolism sites for 35 are in the indazole ring structure. Major metabolites are formed through oxidation.

7.4. Metabolite Profiling Graphs and Tables for 35

Table S9. Metabolites of **35** (CA237) Observed in Mouse Liver Microsomal Incubation

Metabolite	Retention (min)	Mass to Charge Ratio (m/z) in Postive Ion Mode	Presence of Product Ion (Yes/No)			Extra Ion (m/z)	Proposed Structure
			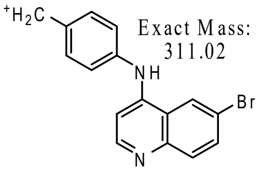 Exact Mass: 311.02	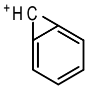 Exact Mass: 89.04	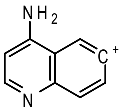 Exact Mass: 143.06		
**M1**	3.69	355.2	No (327 = 311 + 16 (O))	Weak (105 = 89 + 16 (O))	Yes		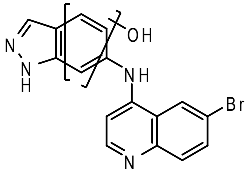
**M2**	4.50	355.2	No (327 = 311 + 16 (O))	Weak (105 = 89 + 16 (O))	Yes		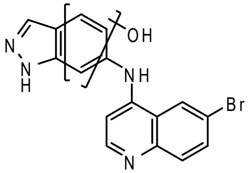
**M3**	5.76	355.2	Weak	No	No	338 (355 - 17 (NH3))	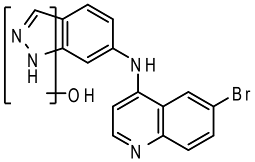
CA237	4.56	339.2	Yes	Yes	Yes		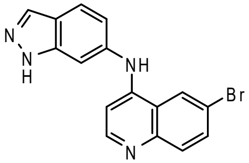

Table S10. Peak Area and Percent Peak Area of Metabolites of **35** (CA237) Observed in Mouse Liver Microsomal Incubation

Metabolite	Retention (min)	Mass to Charge Ratio (m/z)	Peak Area in Liver Microsomes	% Peak Area (over CA237)	Rank in Peak Area Among Metabolites
			Mouse	Mouse	Mouse
**M1**	3.69	355.2	1.67E+06	12.1	1
**M2**	4.50	355.2	7.77E+05	5.6	2
**M3**	5.76	355.2	3.46E+05	2.5	3
**CA237**	4.56	339.2	1.38E+07	100.0	N/A

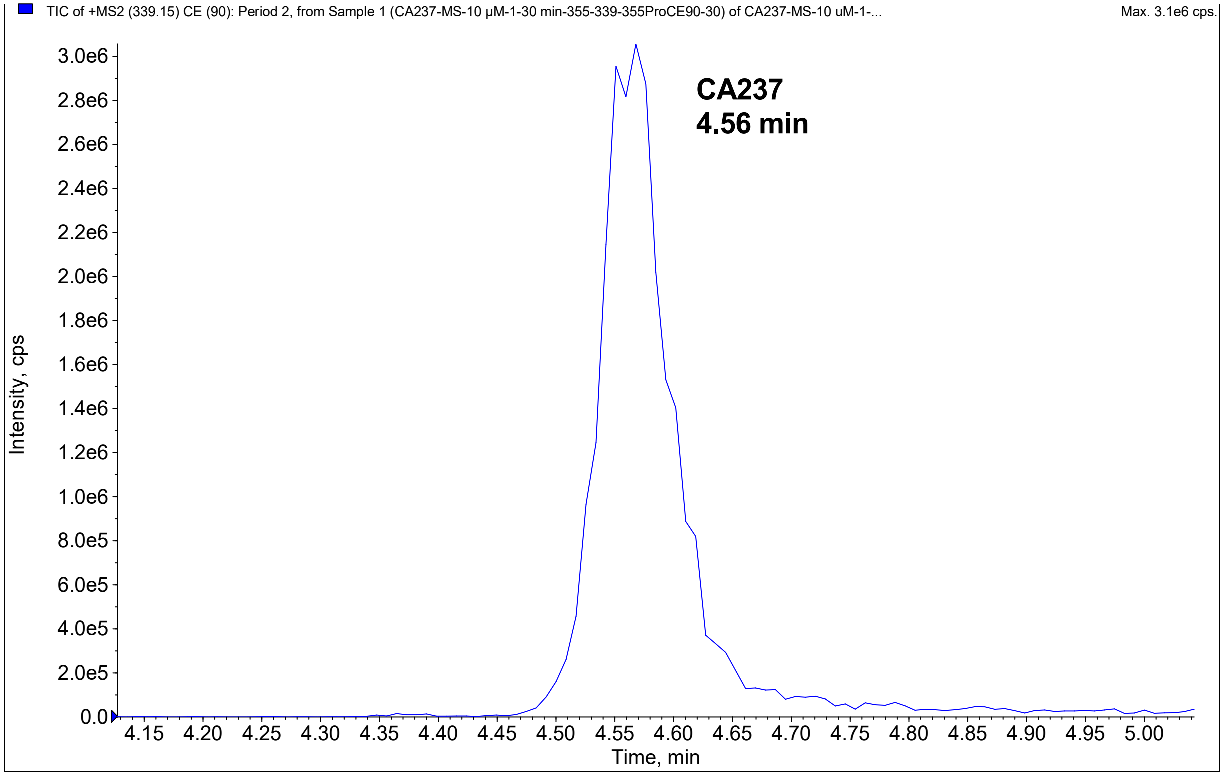

Figure S49. Total ion current chromatogram from product ion scan of ion 339.2 (m/z) from 5 µL injection of 10 µM of **35** incubated in mouse liver microsomes for 30 minutes.

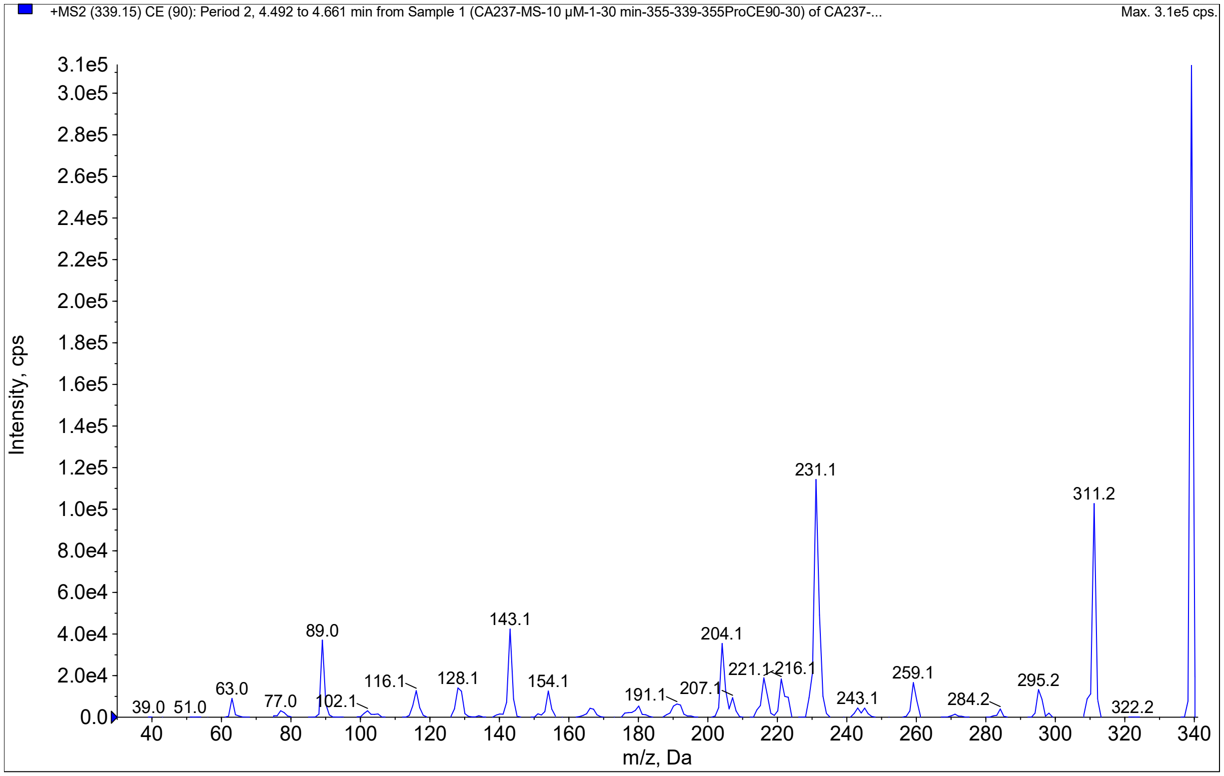

Figure S50. Product ion spectrum for ion 339.2 (m/z) derived from the peak at 4.56 min in **Figure S49**.

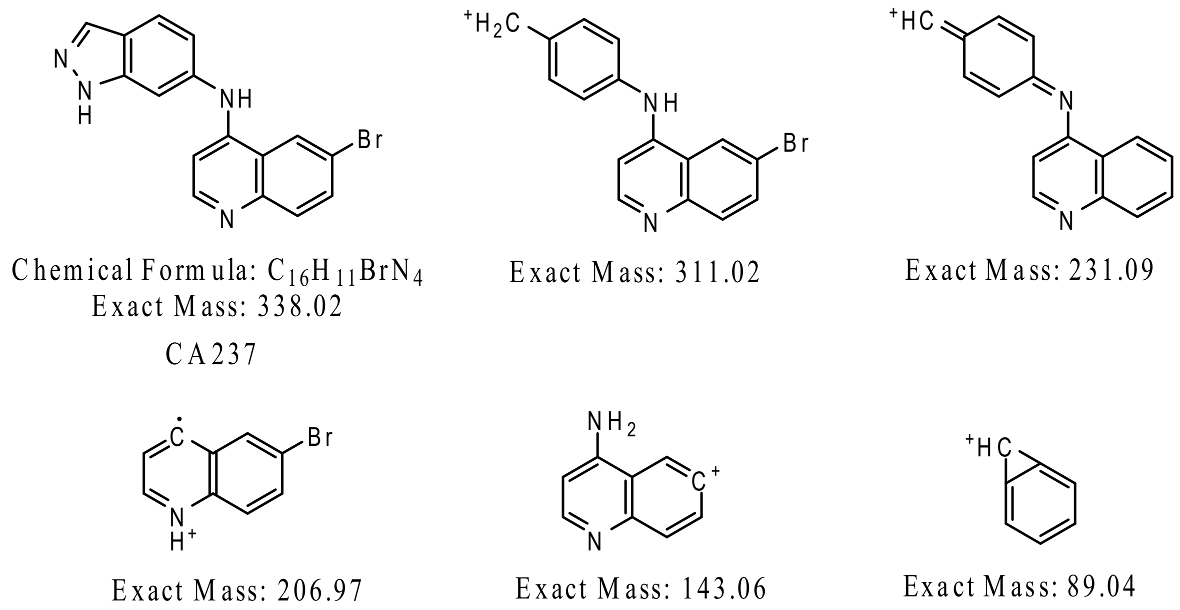

Figure S51. Assignment of fragments in **35** product ion spectrum.

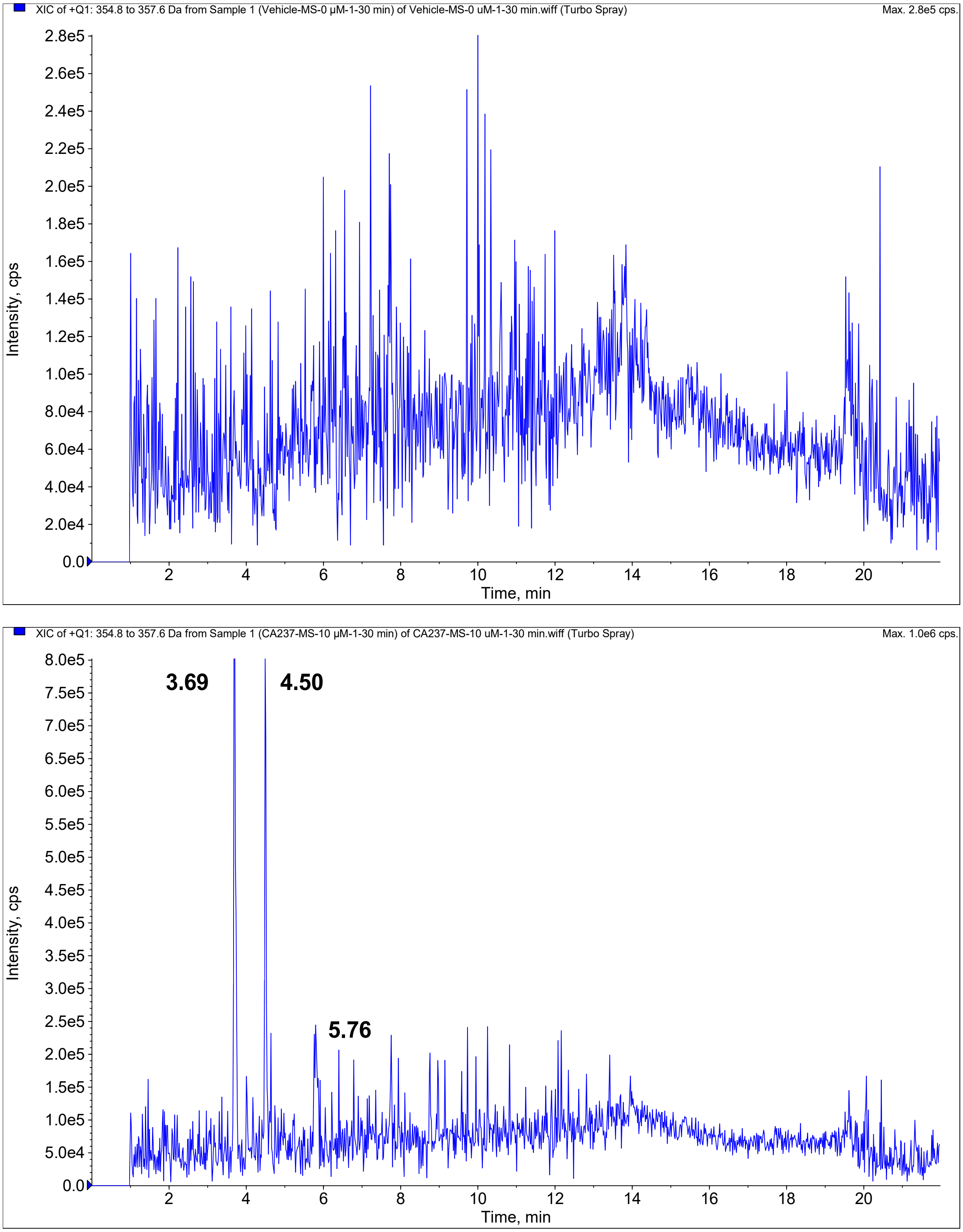

Figure S52. Extract ion (m/z 355) chromatograms from vehicle control sample Vehicle-MS-1 (top) and study sample **35**-10µM-MS-1 (bottom).

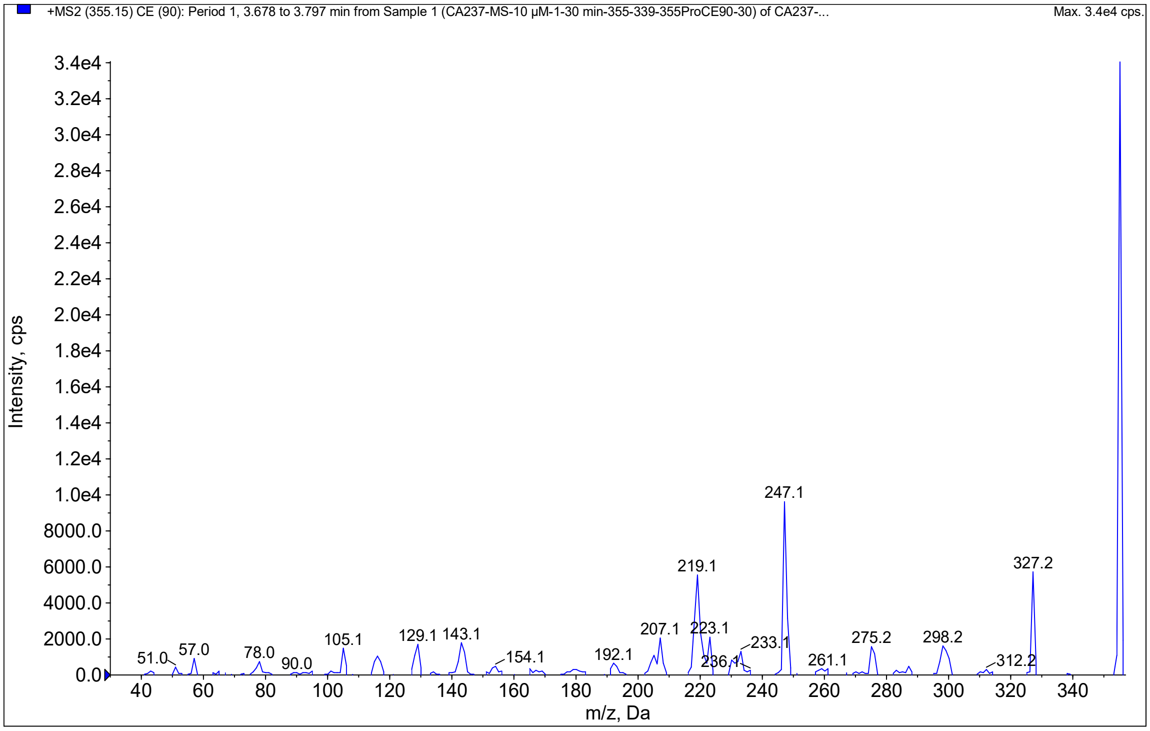

Figure S53. Product ion spectrum for ion 355 (m/z) derived from a peak at 3.69 min in the total ion current chromatogram from product ion scan of ion 355 (m/z) from study sample **35**-10µM-MS-1.

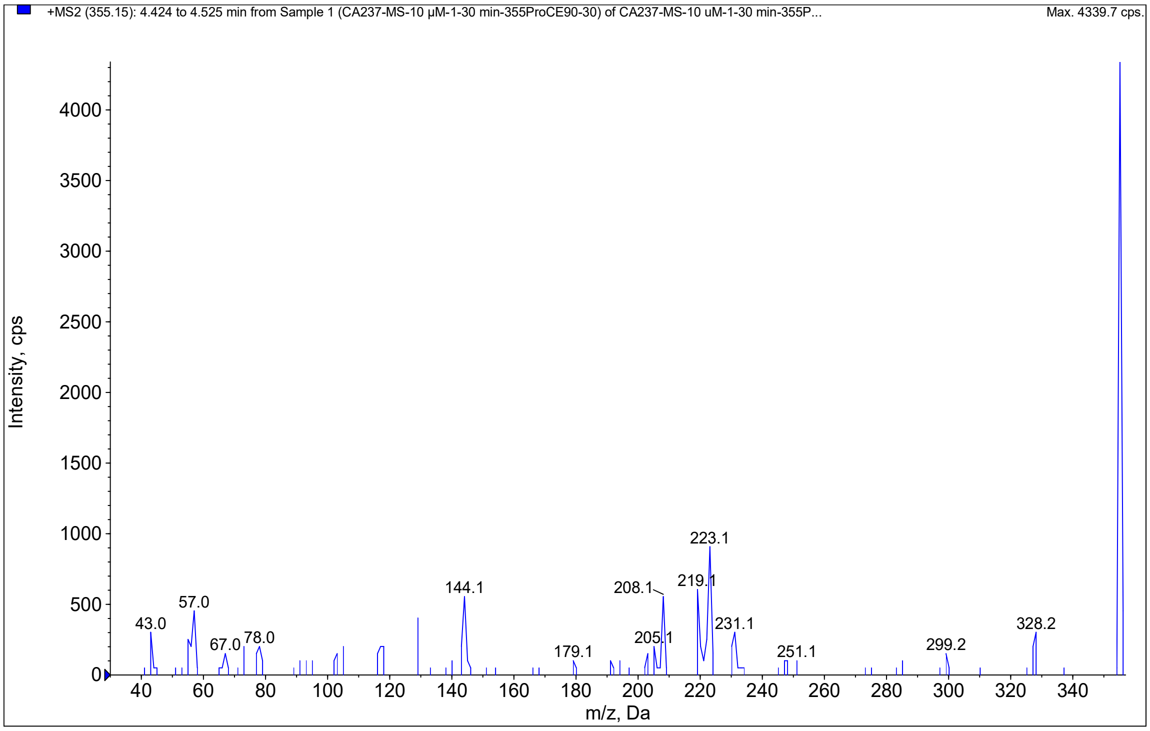

Figure S54. Product ion spectrum for ion 355 (m/z) derived from a peak at 4.50 min in the total ion current chromatogram from product ion scan of ion 355 (m/z) from study sample **35**-10µM-MS-1.

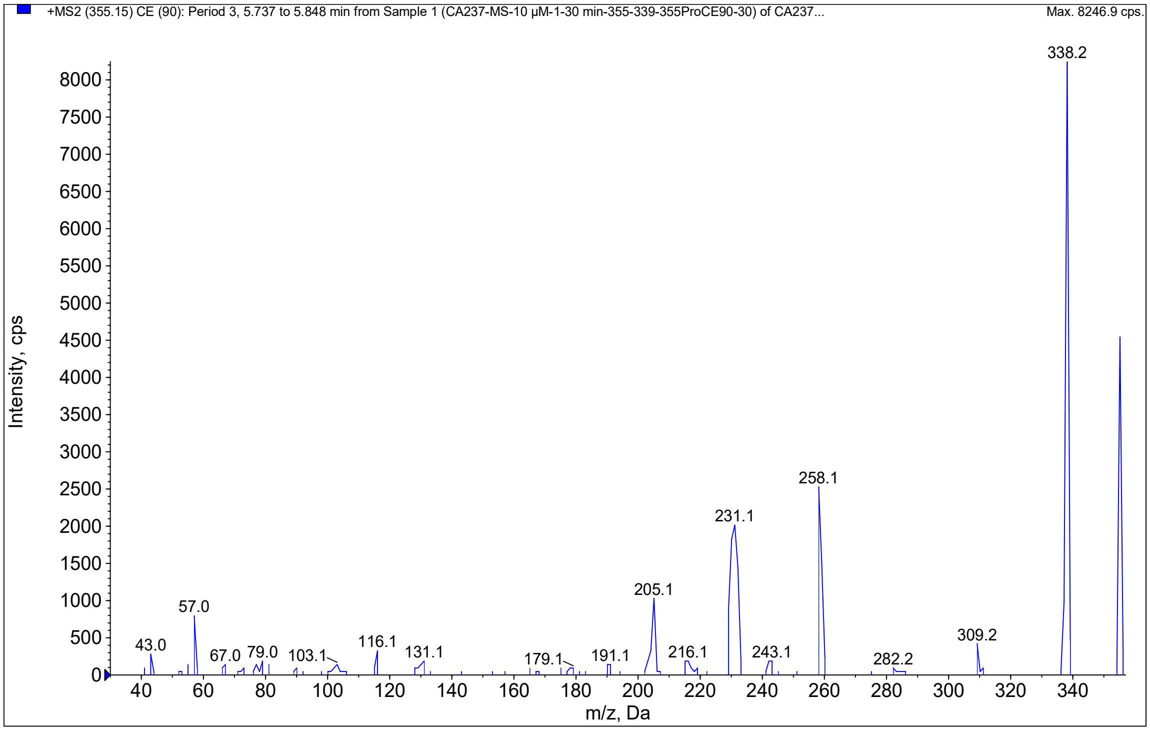

Figure S55. Product ion spectrum for ion 355 (m/z) derived from a peak at 5.76 min in the total ion current chromatogram from product ion scan of ion 355 (m/z) from study sample **35**-10µM-MS-1.

8. NAK family FRET screening and Kinetic Solubility for 1, 8-43

Table S11. NAK family FRET screening and Kinetic Solubility for **1**, **8**-**43**

Number	Binding displacement assay				Kinetic Solubility	
	GAK	AAK1	BMP2K	STK16	PBS pH7.4 (100 μM)	
	K_i (μM)				μM	μg/mL
**1**	0.0031	53	>100	51	57.8	22.5
**8**	0.00054	28	63	>100	128.4	47.6
**9**	0.0019	>100	>100	>100	7.3	2.5
**10**	0.015	3.8	9.8	6.2	2.2	0.7
**11**	0.035	5.7	18	11	39.5	13.2
**12**	0.88	>100	>100	19	-	-
**13**	0.0039	54	>100	17	-	-
**14**	0.0014	9.7	16	20	8.8	3.2
**15**	0.74	3.3	8.7	>100	-	-
**16**	0.46	>100	>100	>100	-	-
**17**	0.0052	>100	>100	>100	-	-
**18**	0.029	3.5	11	>100	-	-
**19**	0.0019	1.9	11	>100	-	-
**20**	0.0043	4.6	16	>100	-	-
**21**	0.037	4.6	12	7.6	24.9	8.5
**22**	0.019	4.6	15	15	3.6	1.2
**23**	0.011	>100	>100	>100	25.9	8.8
**24**	0.0041	2.6	7.9	7.8	8.6	3.1
**25**	0.026	1.2	>100	>100	1.1	0.4
**26**	0.023	>100	>100	>100	-	-
**27**	0.066	1.3	2.3	26	-	-
**28**	0.041	4.6	13	11	-	-
**29**	0.0053	2.0	4.3	22	1.7	0.6
**30**	2.8	8.7	20	>100	-	-
**31**	3.6	13	16	28	-	-
**32**	0.014	4.6	7.4	19	5.1	1.7
**33**	0.72	4.6	16	11	24.2	8.2
**34**	0.034	0.58	2.1	3.4	19.4	6.4
**35**	0.020	2.1	6.5	7.4	12.3	4.2
**36**	-	-	-	-	2.0	0.7
**37**	0.047	0.52	0.66	5.7	12.3	4.2
**38**	0.062	1.4	0.98	2.7	-	-
**39**	0.36	1.9	22	21	-	-
**40**	0.68	2.4	4.9	8.8	-	-
**41**	0.11	37	79	>100	-	-
**42**	1.6	4.1	6.6	9.3	-	-
**43**	1.7	3.3	6.4	8.0	-	-

9. LabBook Codes and SMILES for 1, 8-43.

Table S12. LabBook Codes and SMILES for **1**, **8**-**43**.

Name	Lab Book Code	SMILES
1	CA93.0	BrC1=CC2=C(NC3=CC(OC)=C(OC)C(OC)=C3)C=CN=C2C=C1
8	CA75	COC1=CC2=C(NC3=CC(OC)=C(OC)C(OC)=C3)C=CN=C2C=C1OC
9	CA285	BrC(C=C1)=CC(C1=NC=C2)=C2NC3=C(OCO4)C4=CC=C3
10	CA378	BrC(C=C1)=CC2=C1N=CC=C2NC3=CC(F)=CC=C3F
11	CA379	FC1=CC(F)=CC=C1NC2=CC=NC3=C2C=C(Br)C=C3
12	CA477	FC1=C(NC2=CC=NC3=C2C=C(Br)C=C3)C(F)=C(F)C(F)=C1F
13	CA62	FC(C1=CC2=C(NC3=CC(OC)=C(OC)C(OC)=C3)C=CN=C2C=C1)(F)F
14	CA485	FC1=CC=C2C(OCO2)=C1NC3=CC=NC4=C3C=C(C=C4)Br
15	CA404	COC1=CC=CC(NC2=C(C=C(Br)C=C3)C3=NC=C2)=C1OC
16	CA421	FC1(F)OC2=C(NC3=C(C=C(Br)C=C4)C4=NC=C3)C=CC=C2O1
17	CA449	BrC(C=C1)=CC(C1=NC=C2)=C2NC3=C(CCO4)C4=CC=C3
18	CA457	BrC(C=C1)=CC(C1=NC=C2)=C2NC3=C(OCC4)C4=CC=C3
19	CA448	BrC(C=C1)=CC(C1=NC=C2)=C2NC3=C(C=CO4)C4=CC=C3
20	CA450	BrC(C=C1)=CC(C1=NC=C2)=C2NC3=CC=CC4=C3OC=C4
21	CA406	BrC(C=C1)=CC(C1=NC=C2)=C2NC3=C(ON=C4)C4=CC=C3
22	CA487	BrC(C=C1)=CC(C1=NC=C2)=C2NC3=C(C=NO4)C4=CC=C3
23	CA422	BrC(C=C1)=CC(C1=NC=C2)=C2NC3=C(OC=N4)C4=CC=C3
24	CA415	BrC(C=C1)=CC(C1=NC=C2)=C2NC3=C(SC=N4)C4=CC=C3
25	CA405	BrC(C=C1)=CC(C1=NC=C2)=C2NC3=C(N=CS4)C4=CC=C3
26	CA451	BrC(C=C1)=CC(C1=NC=C2)=C2NC3=C(C=CS4)C4=CC=C3
27	CA423	BrC(C=C1)=CC(C1=NC=C2)=C2NC3=C(NC=N4)C4=CC=C3
28	CA424	BrC(C=C1)=CC(C1=NC=C2)=C2NC3=CC=CC4=NON=C34
29	CA425	BrC(C=C1)=CC(C1=NC=C2)=C2NC3=CC=CC4=NSN=C34
30	CA427	BrC(C=C1)=CC(C1=NC=C2)=C2NC3=C(N=NN4C)C4=CC=C3
31	CA429	BrC(C=C1)=CC(C1=NC=C2)=C2NC3=C(C=NN4C)C4=CC=C3
32	CA416	BrC(C=C1)=CC(C1=NC=C2)=C2NC3=C(C=NN4)C4=CC=C3
33	CA478	FC(C(C=C1)=CC(C1=NC=C2)=C2NC3=C(C=NN4)C4=CC=C3)(F)F
34	CA476	FC(C(C=C1)=CC(C1=NC=C2)=C2NC3=C(NN=C4)C4=CC=C3)(F)F
35	CA237	BrC1=CC2=C(C=C1)N=CC=C2NC3=CC(NN=C4)=C4C=C3
36	CA488	FC1=CC2=C(NN=C2)C=C1NC3=CC=NC4=C3C=C(Br)C=C4
37	CA236	BrC1=CC2=C(C=C1)N=CC=C2NC3=CC(C=NN4)=C4C=C3
38	CA238	BrC(C=C1)=CC(C1=NC=C2)=C2NC3=CC=C(CNC4=O)C4=C3
39	CA244	BrC(C=C1)=CC(C1=NC=C2)=C2NC3=CC=C(NC(N4)=O)C4=C3
40	CA246	BrC(C=C1)=CC(C1=NC=C2)=C2NC3=CC=C(NC(O4)=O)C4=C3
41	CA261	BrC(C=C1)=CC(C1=NC=C2)=C2NC3=CC=C(OC(N4)=O)C4=C3
42	CA255	BrC(C=C1)=CC(C1=NC=C2)=C2NC3=CC=C(N(C)C(N4)=O)C4=C3
43	CA243	BrC(C=C1)=CC(C1=NC=C2)=C2NC3=CC=C(CS(C4)(=O)=O)C4=C3

10. Characterisation of compounds 9-43

N-(benzo[*d*][1,3]dioxol-4-yl)-6-bromoquinolin-4-amine (**9**)

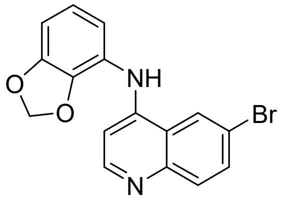

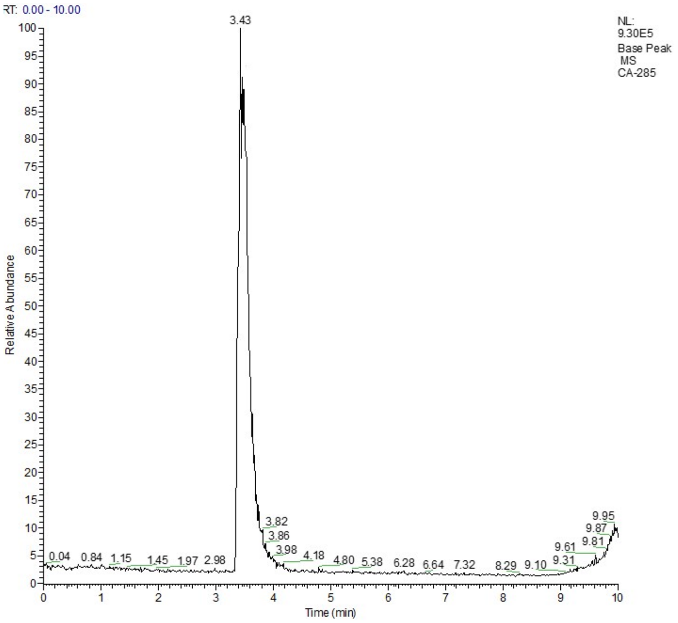

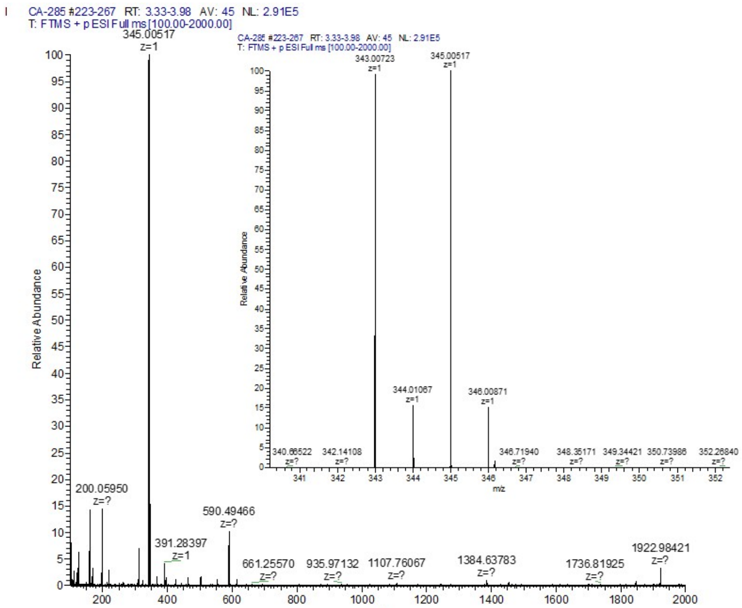

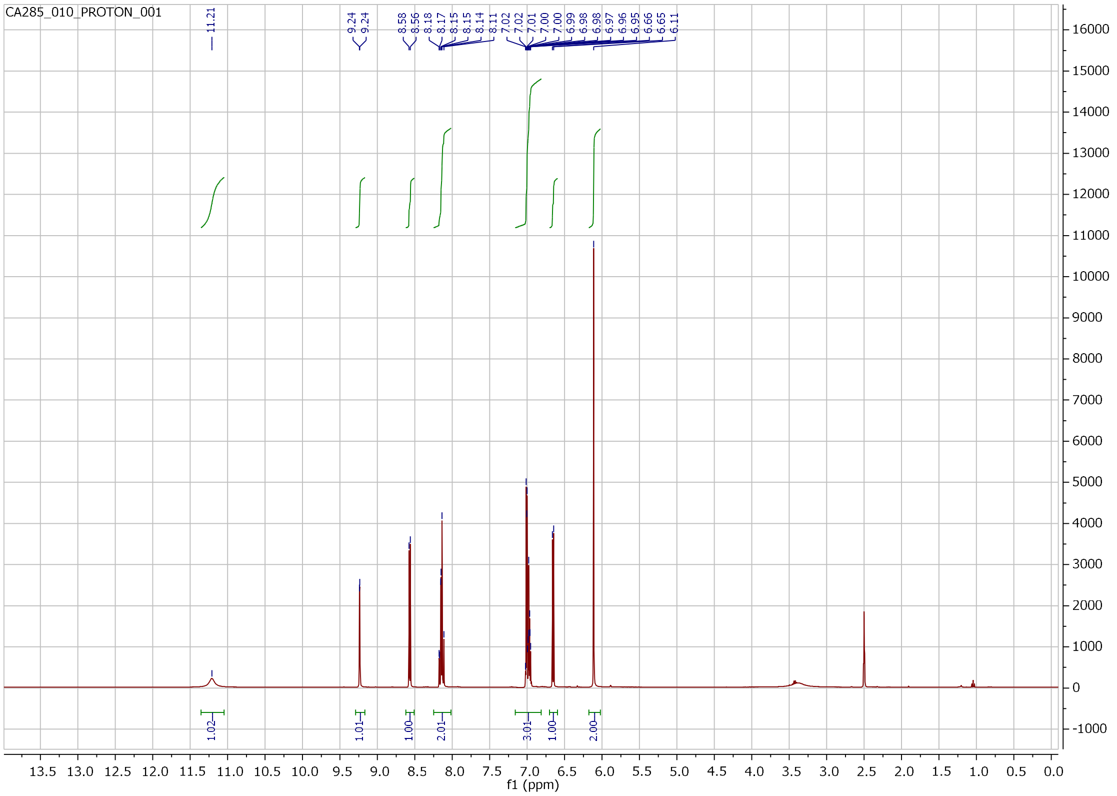
CA285_010_PROTON_001
f1 (ppm)

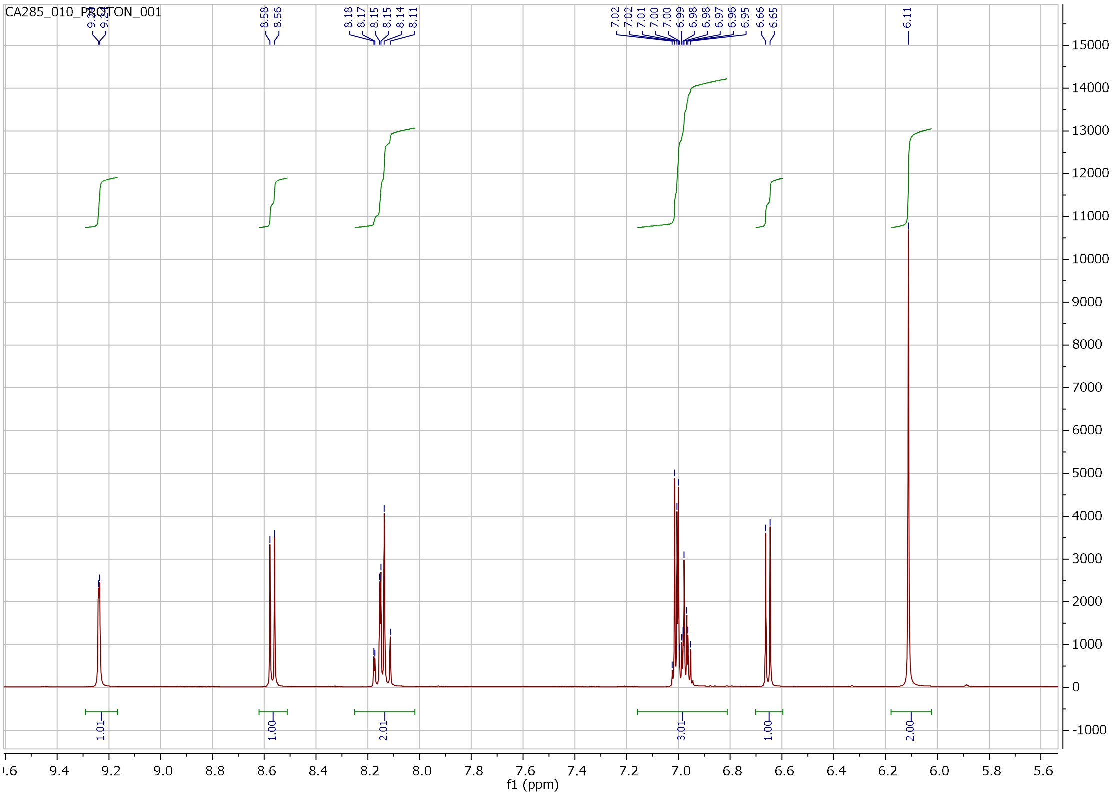
CA285_010_PROTON_001
f1 (ppm)

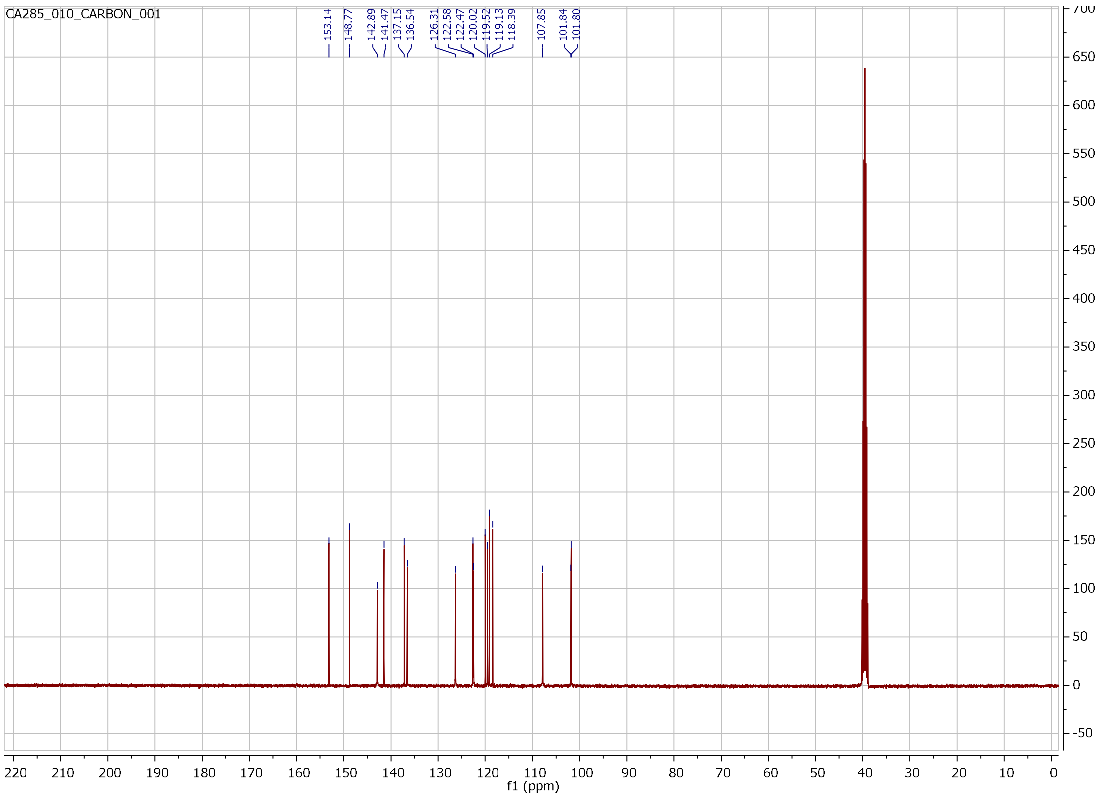
CA285_010_CARBON_001
153.14
148.77
142.89
141.47
137.15
136.54
126.31
122.58
122.47
120.02
119.52
119.13
118.39
107.85
101.84
101.80
f1 (ppm)

6-bromo-*N*-(2,5-difluorophenyl)quinolin-4-amine (**10**)

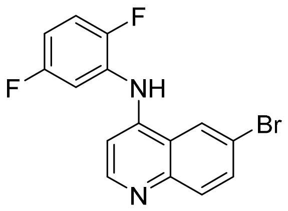

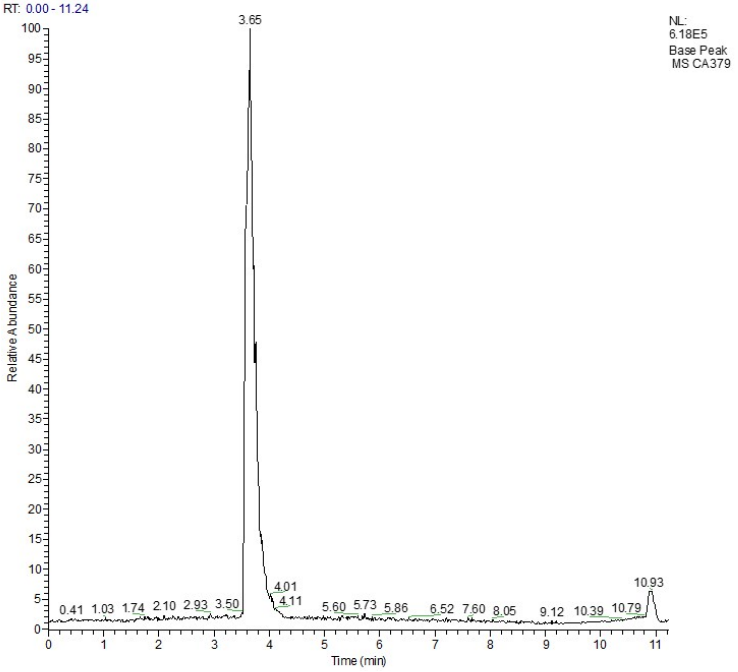

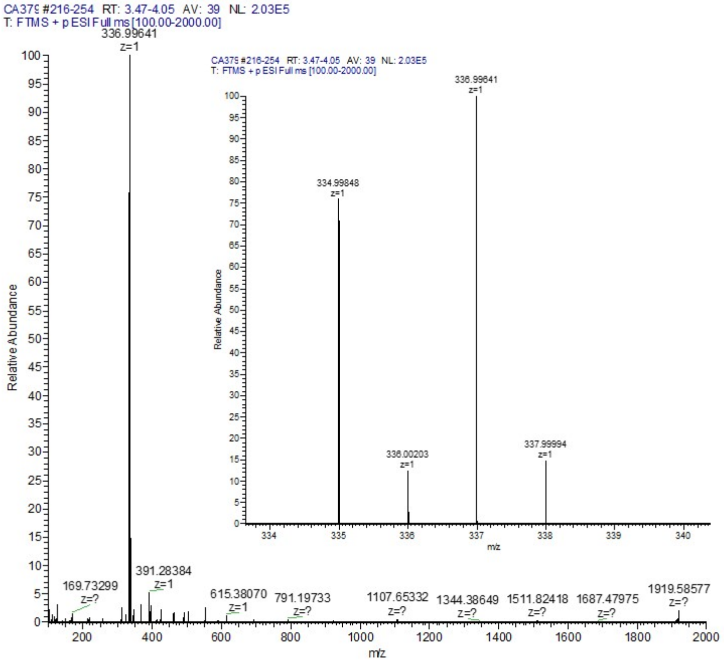

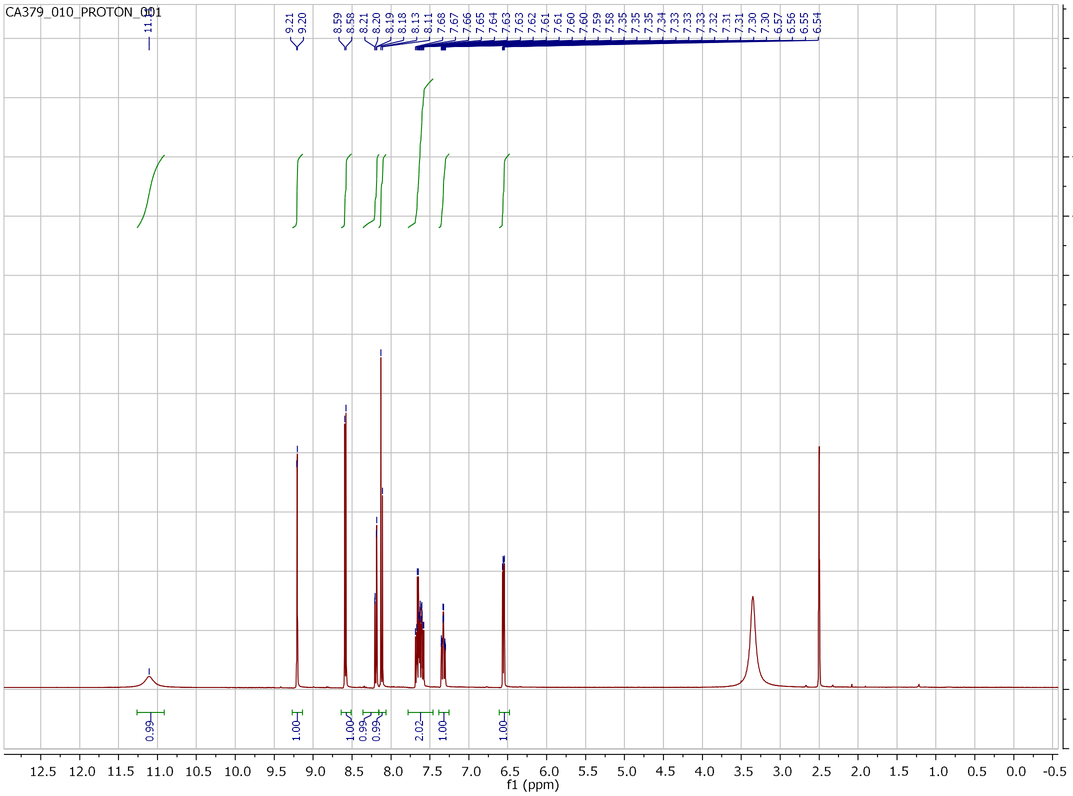
CA379_010_PROTON_001
f1 (ppm)

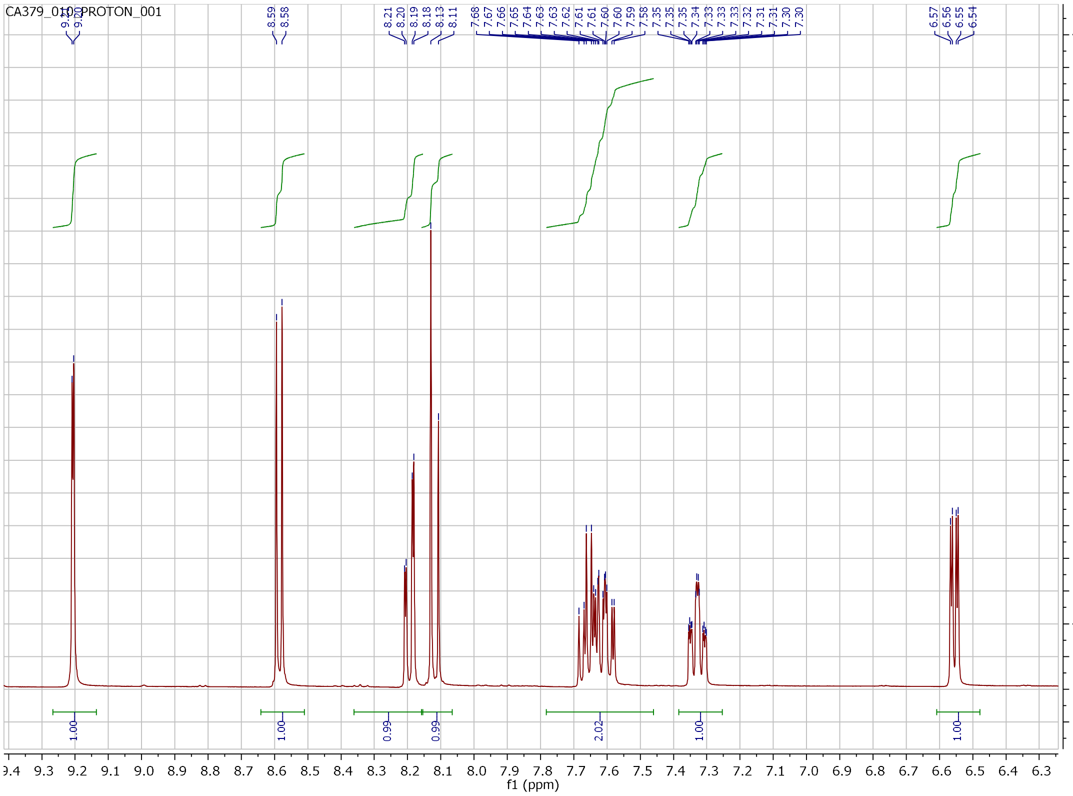
CA379_010_PROTON_001
f1 (ppm)

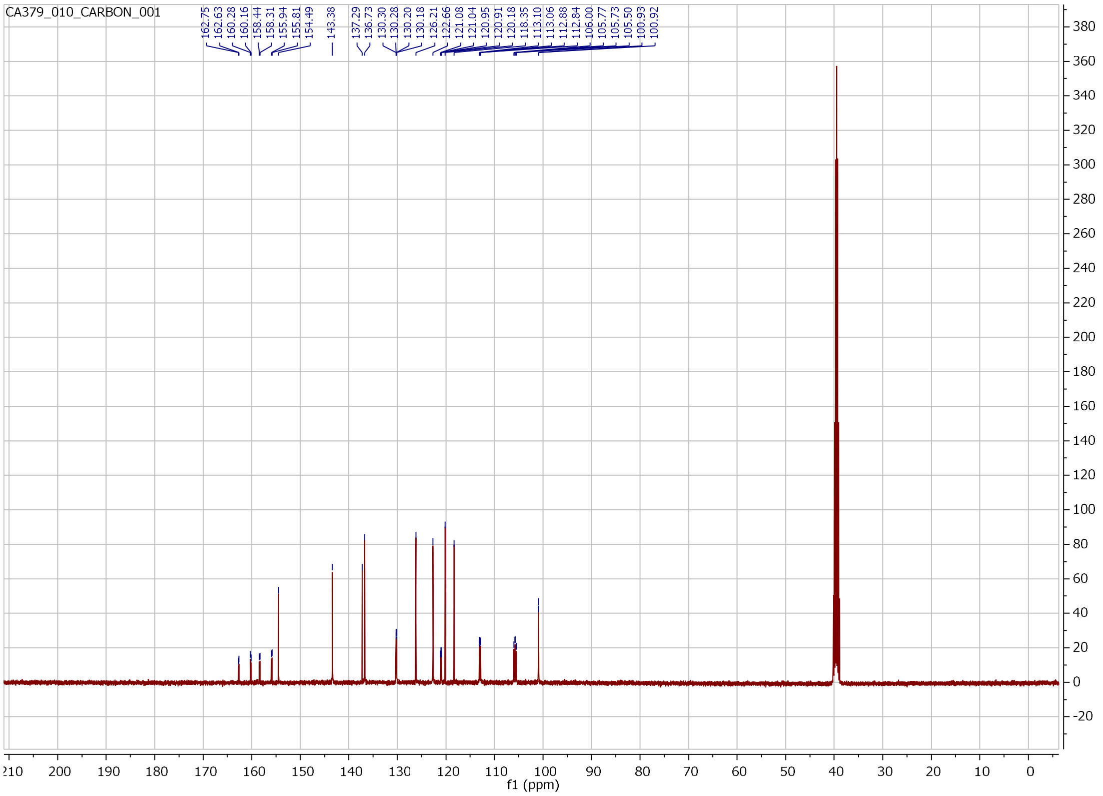
CA379_010_CARBON_001
162.75
162.63
160.28
160.16
158.44
158.31
155.94
155.81
154.49
143.38
137.29
136.73
130.30
130.28
130.20
130.18
126.21
122.66
121.08
121.04
120.95
120.91
120.18
118.35
113.10
113.06
112.88
112.84
106.00
105.77
105.73
105.50
100.93
100.92
f1 (ppm)

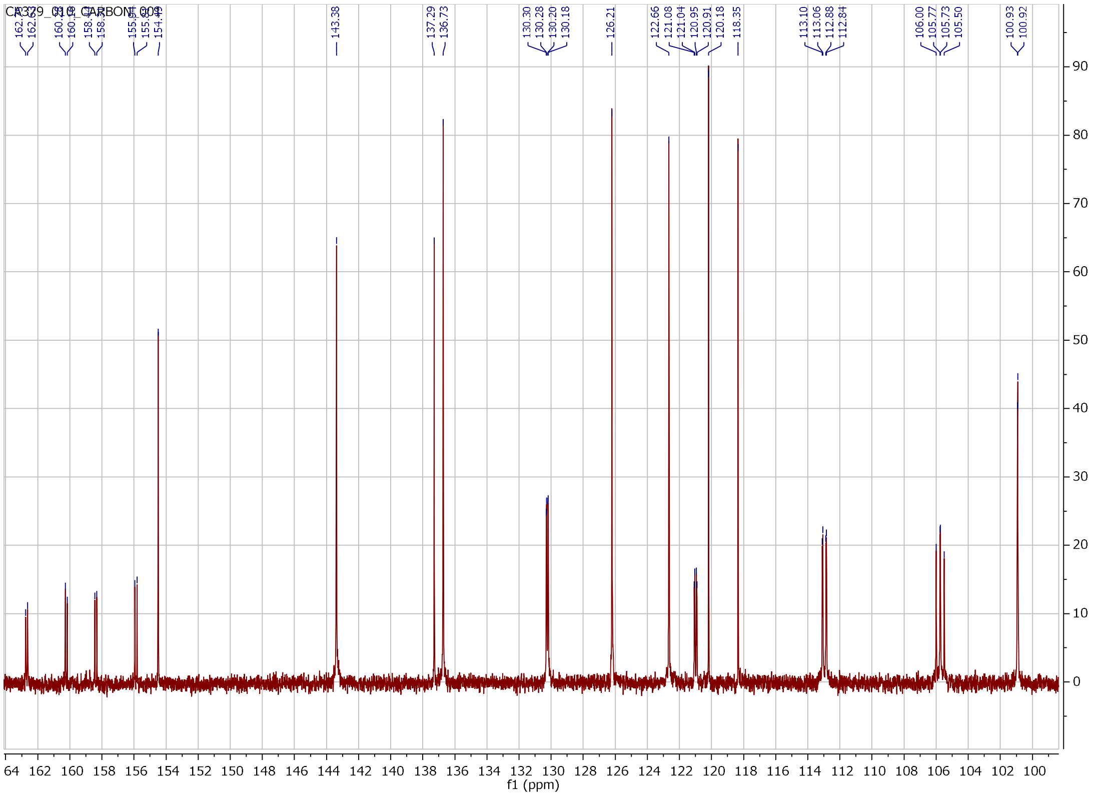
CA379_010_CARBON_001
143.38
137.29
136.73
130.30
130.28
130.20
130.18
126.21
122.66
121.08
121.04
120.95
120.91
120.18
118.35
113.10
113.06
112.88
112.84
106.00
105.77
105.73
105.50
100.93
100.92
f1 (ppm)

6-bromo-*N*-(2,4-difluorophenyl)quinolin-4-amine (**11**)

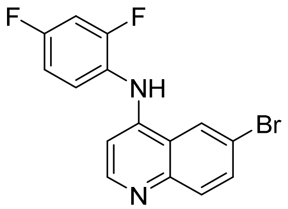

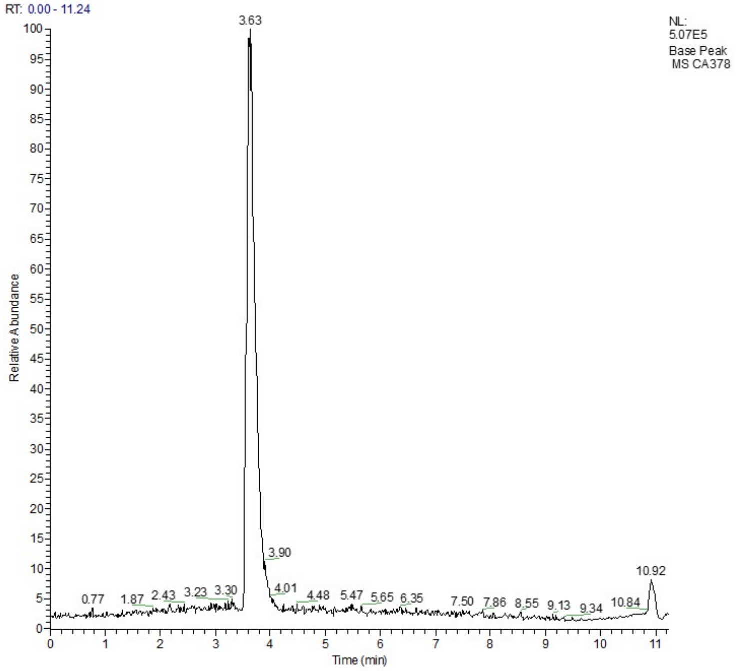

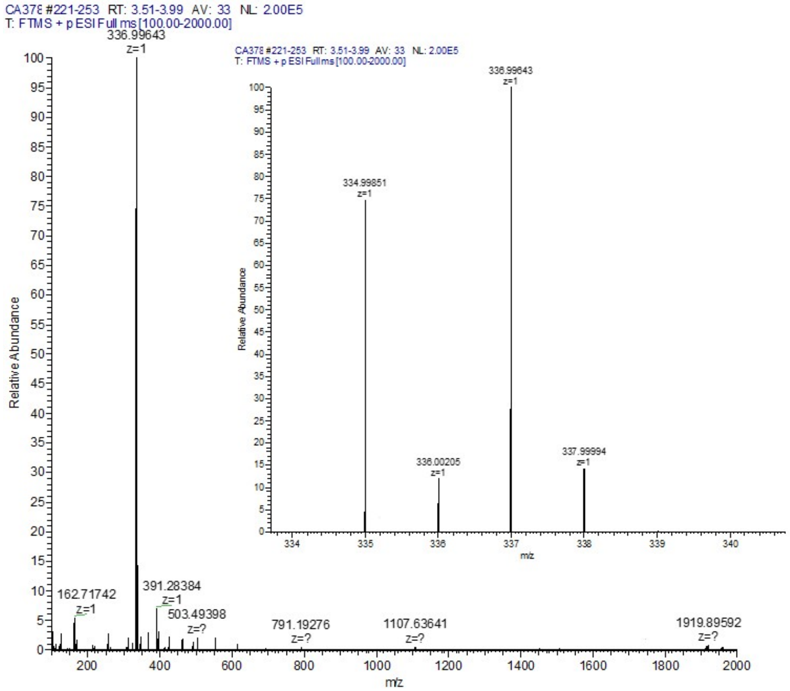

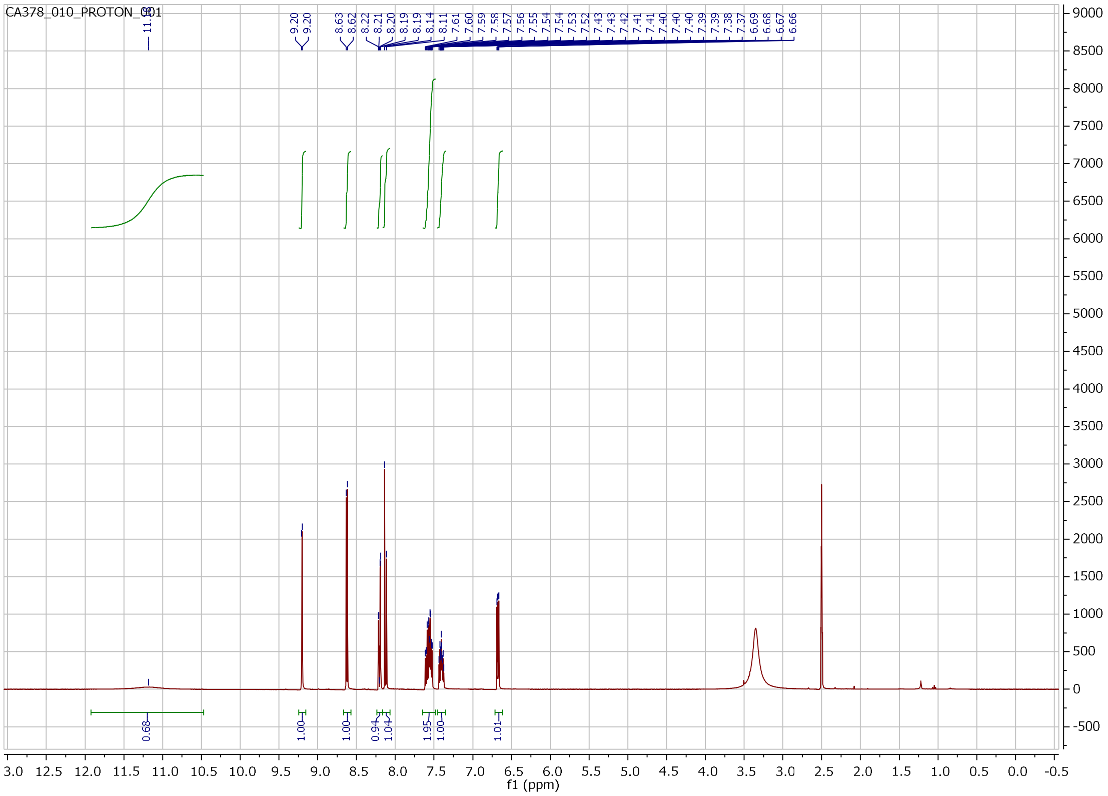
CA378_010_PROTON_001
f1 (ppm)

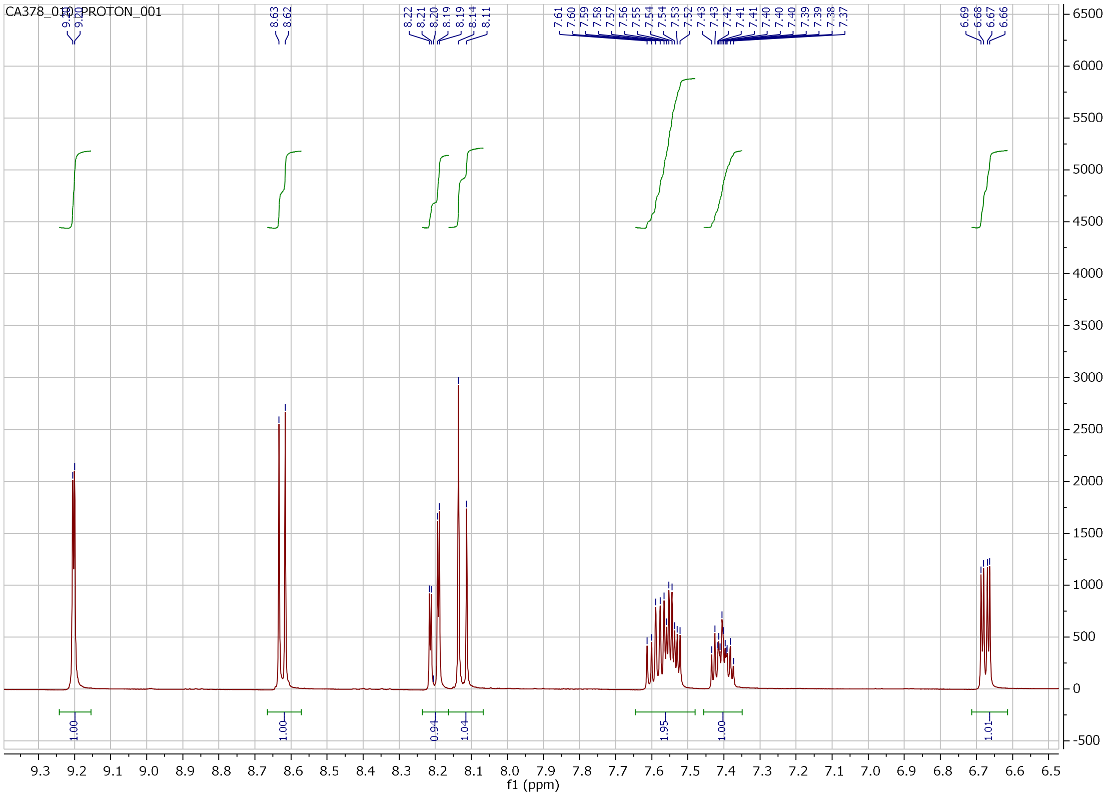
CA378_010_PROTON_001
f1 (ppm)

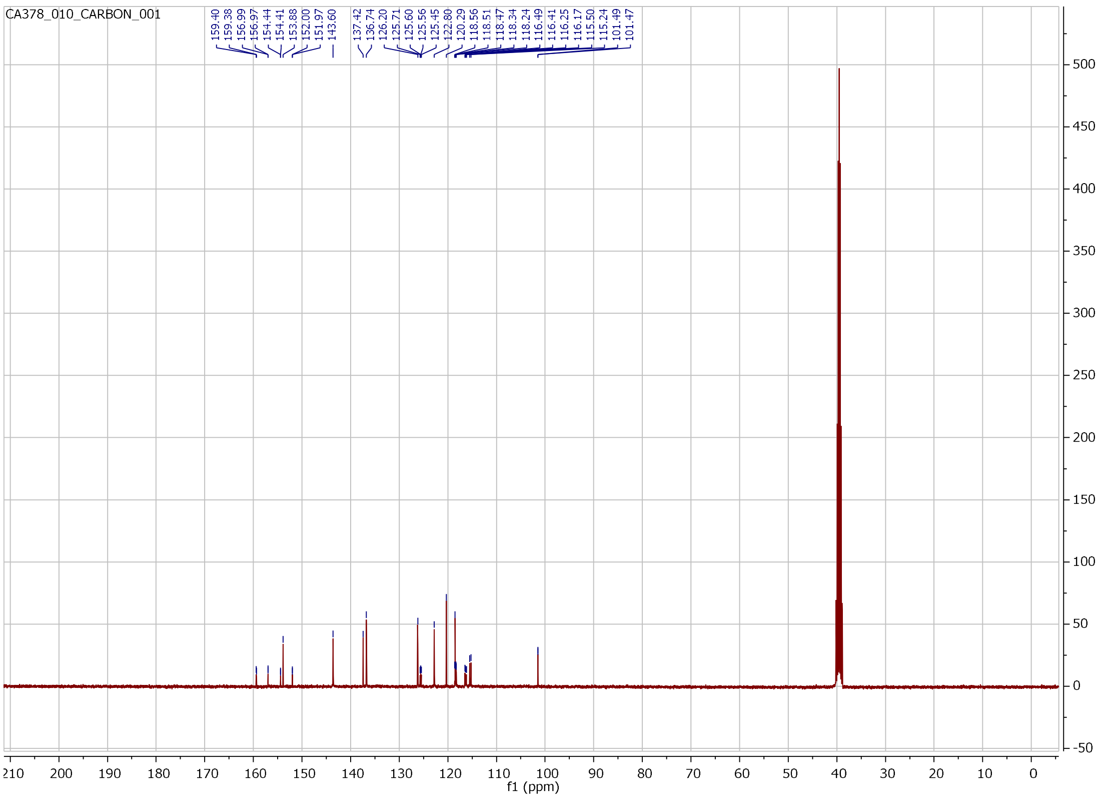

CA378_010_CARBON_001
159.40
159.38
156.99
156.97
154.44
154.41
153.88
152.00
151.97
143.60
137.42
136.74
126.20
125.71
125.60
125.56
125.45
122.80
120.29
118.56
118.51
118.47
118.34
118.24
116.49
116.41
116.25
116.17
115.50
115.24
101.49
101.47
f1 (ppm)

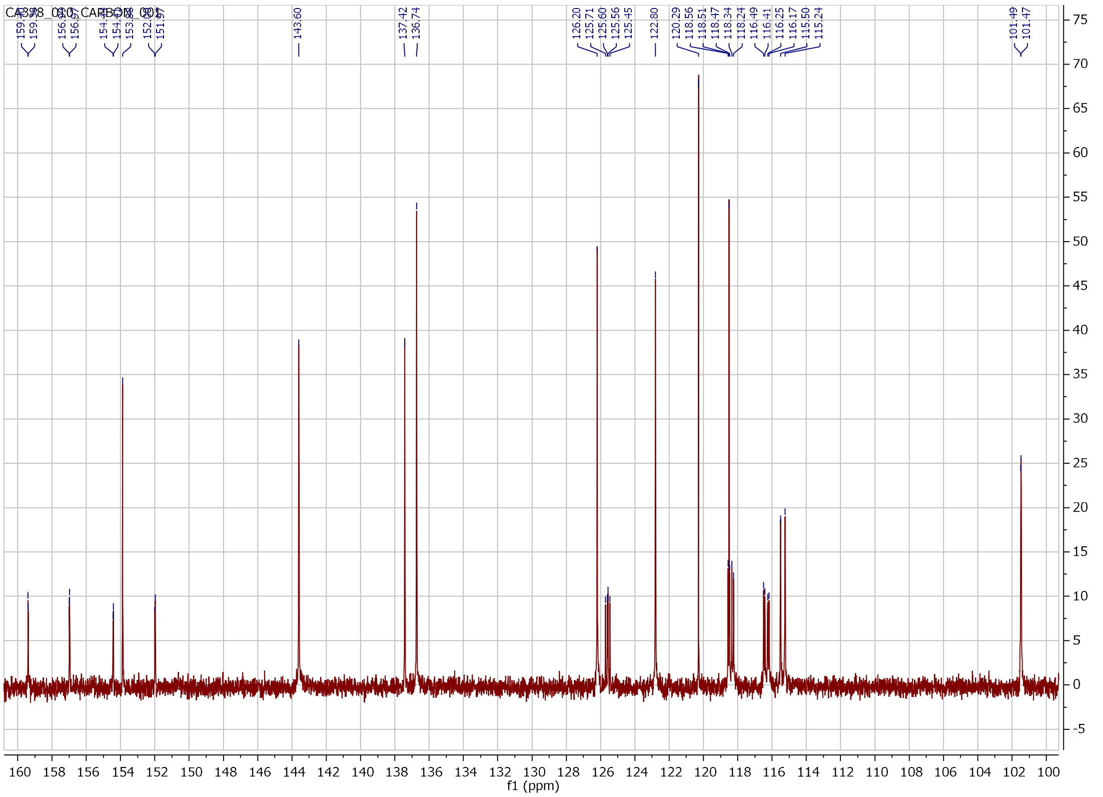

143.60
137.42
136.74
126.20
125.71
125.60
125.56
125.45
122.80
120.29
118.56
118.51
118.47
118.34
118.24
116.49
116.41
116.25
116.17
115.50
115.24
101.49
101.47
f1 (ppm)

6-bromo-*N*-(perfluorophenyl)quinolin-4-amine (**12**)

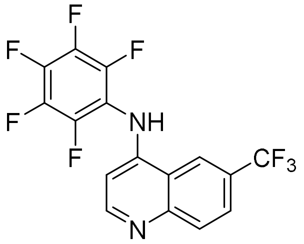

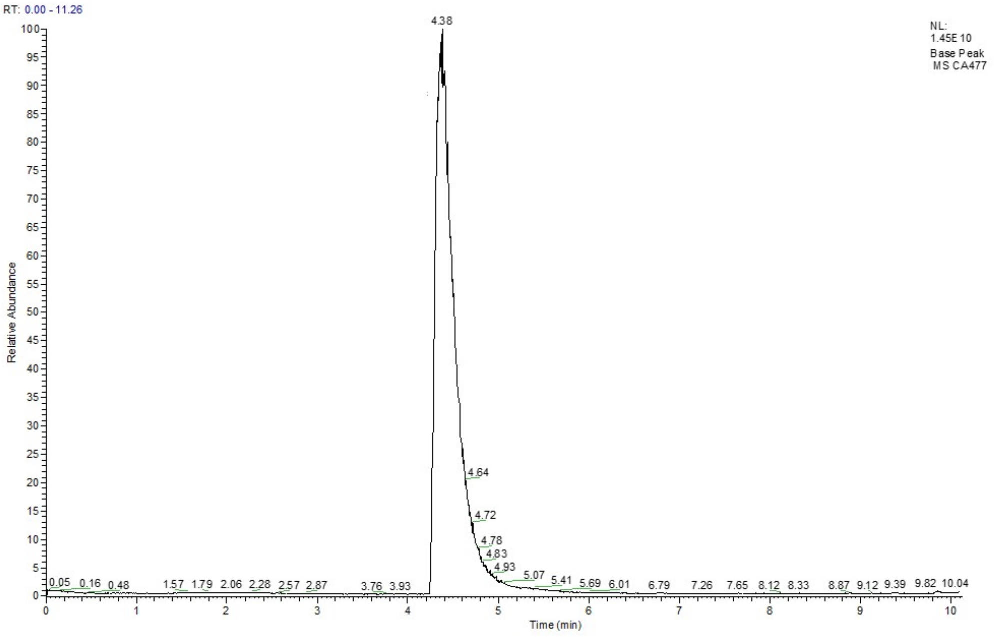

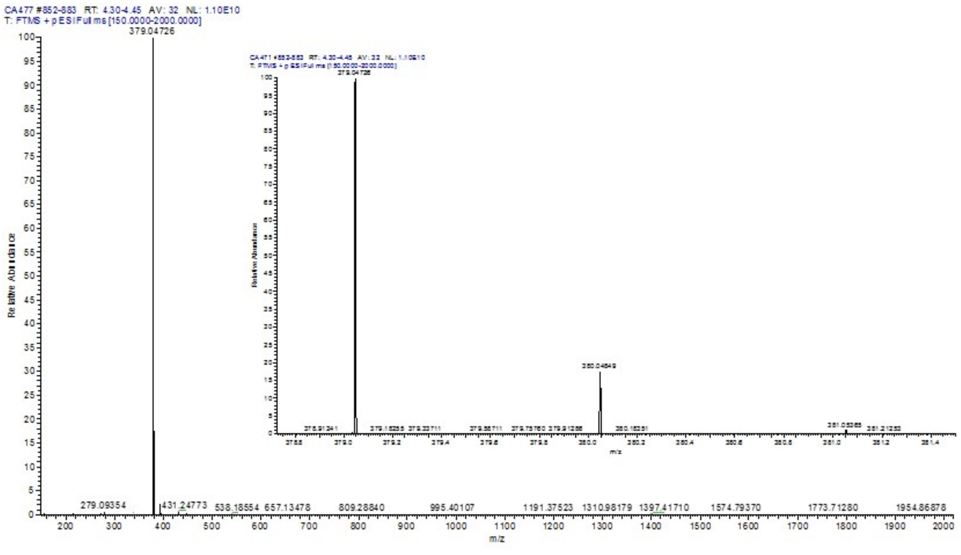

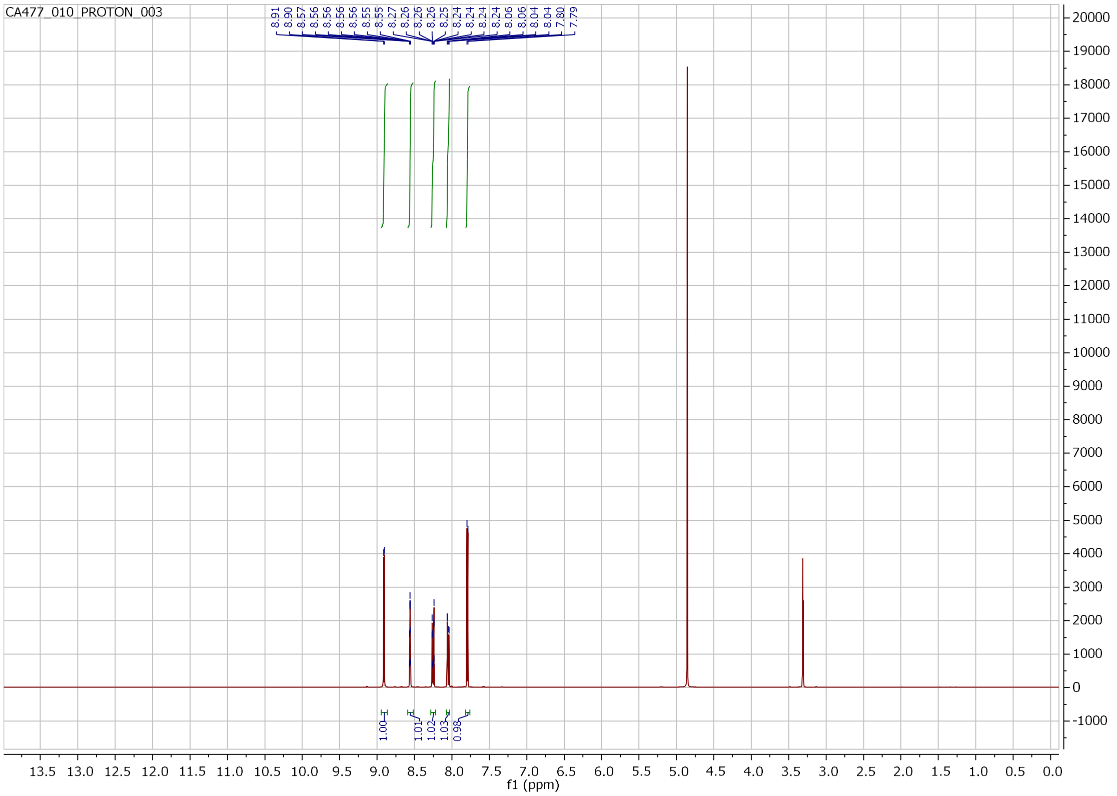
CA477_010_PROTON_003
8.91 8.90 8.57 8.56 8.56 8.56 8.56 8.55 8.55 8.27 8.26 8.26 8.26 8.25 8.24 8.24 8.24 8.24 8.06 8.06 8.04 7.80 7.79
1.00 1.01 1.02 1.03 0.98
f1 (ppm)

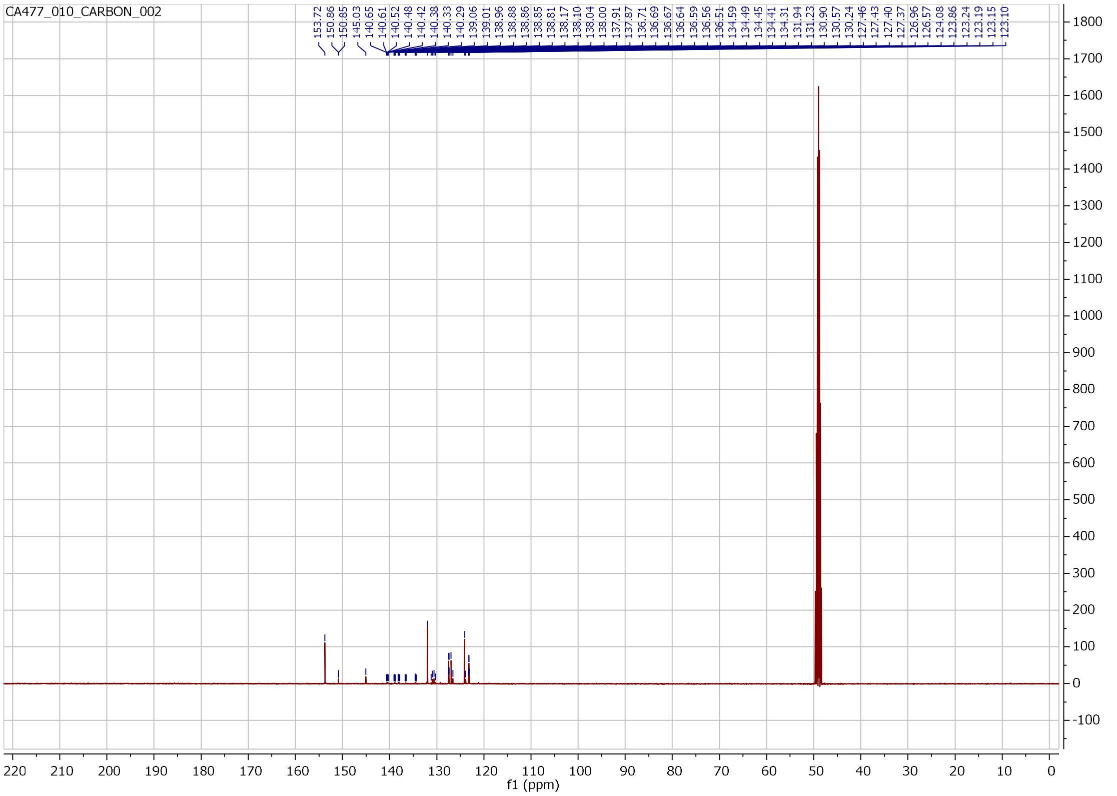
CA477_010_CARBON_002
153.72 150.86 150.85 145.03 140.65 140.61 140.52 140.48 140.42 140.38 140.33 140.29 139.06 139.01 138.96 138.88 138.86 138.85 138.81 138.17 138.10 138.04 138.00 137.91 137.87 136.71 136.69 136.67 136.64 136.59 136.56 136.51 134.59 134.49 134.45 134.41 134.31 131.94 131.23 130.90 130.57 130.24 127.46 127.43 127.40 127.37 126.96 126.57 124.08 123.86 123.24 123.19 123.15 123.10
f1 (ppm)

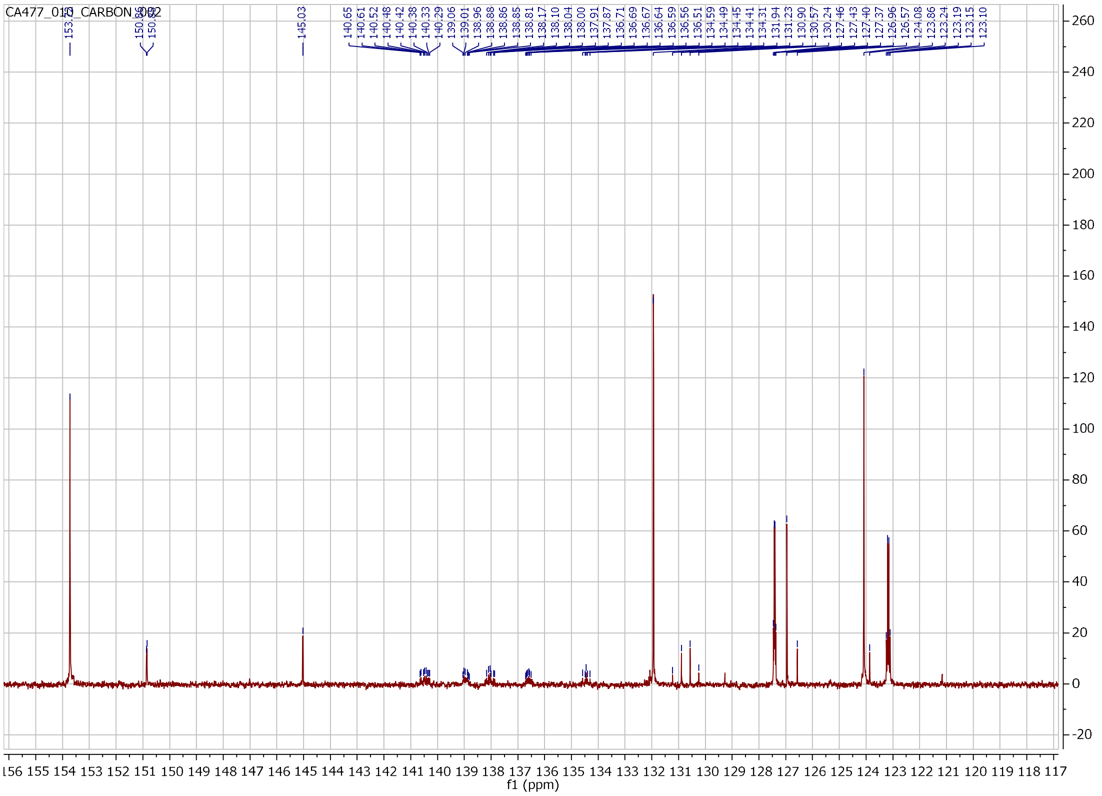

CA477_010_CARBON.002
f1 (ppm)

6-bromo-*N*-(5-fluorobenzo[*d*][1,3]dioxol-4-yl)quinolin-4-amine (**14**)

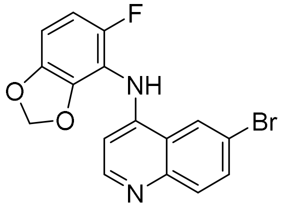

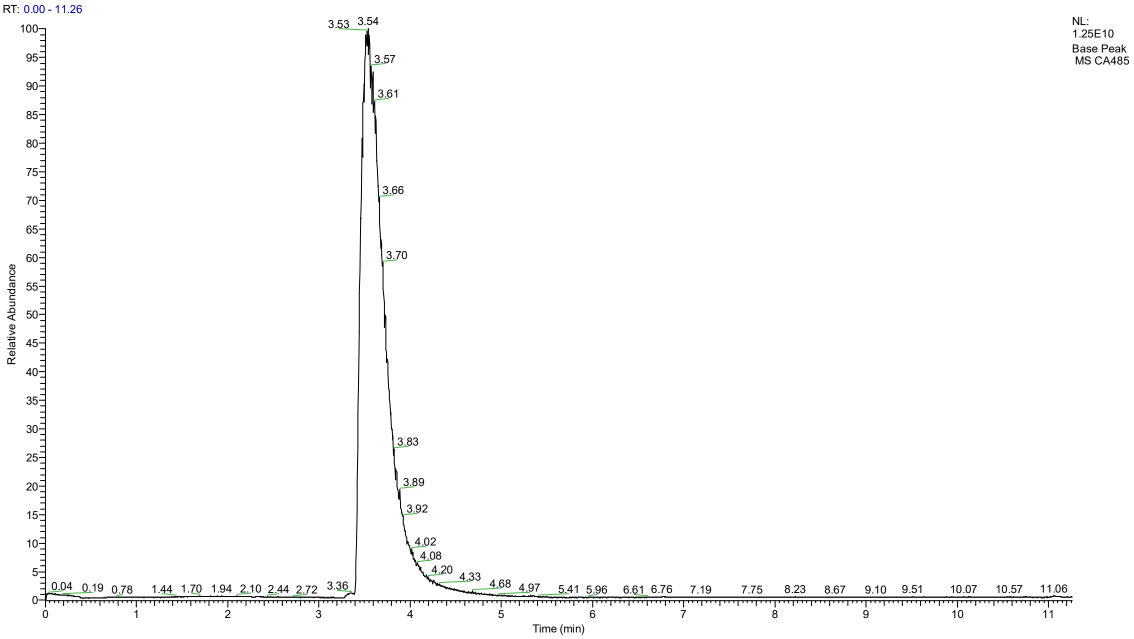

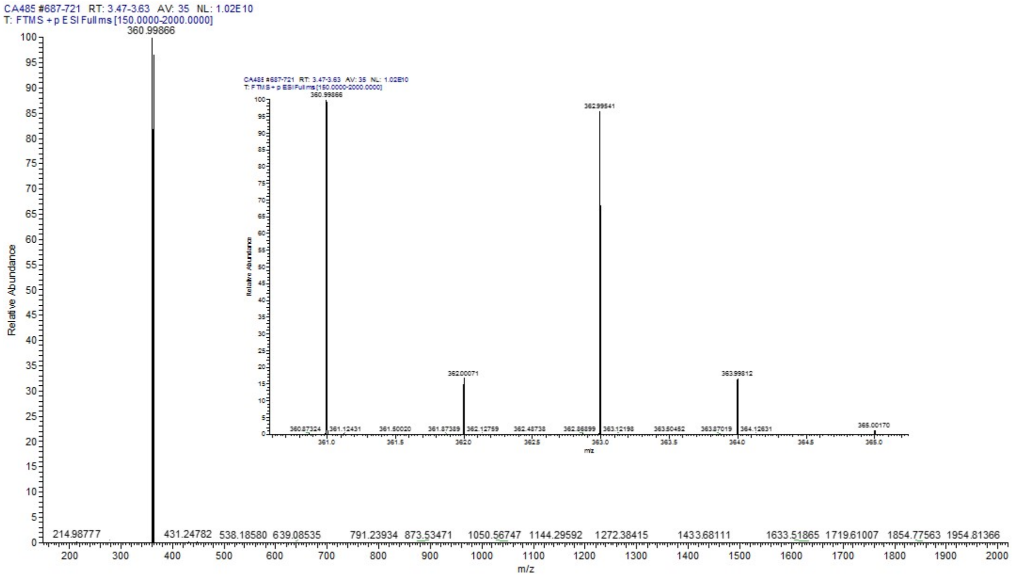

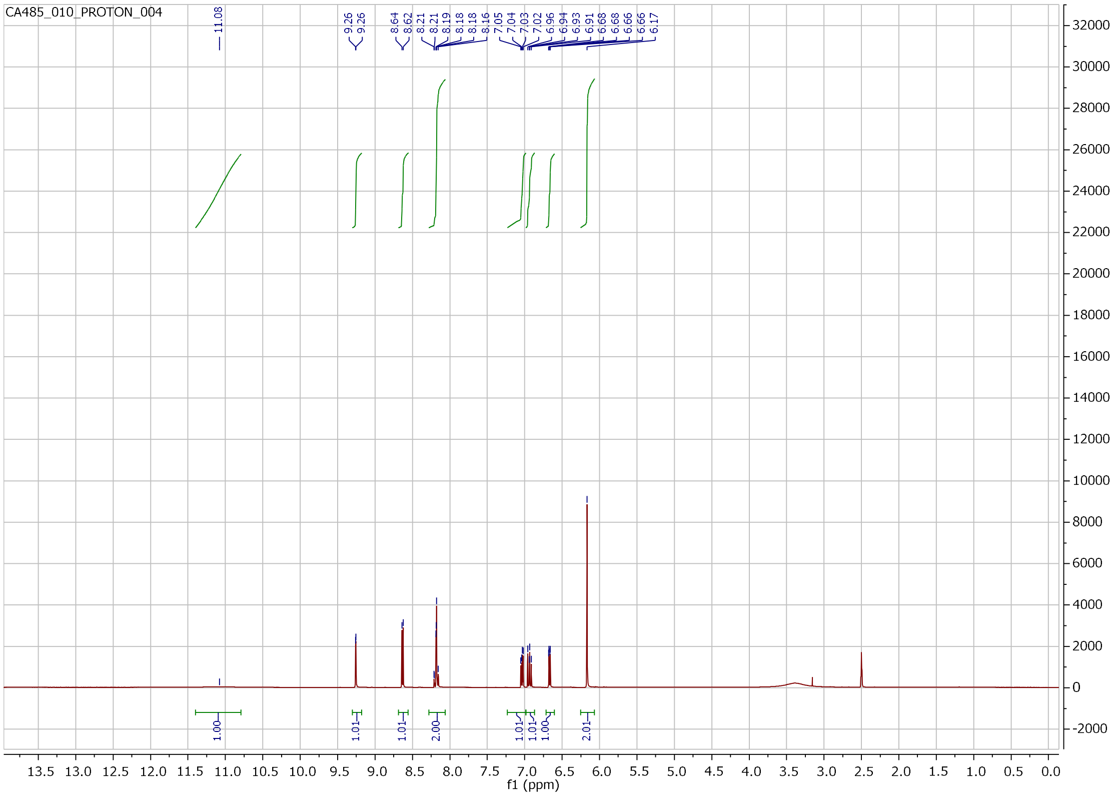
CA485_010_PROTON_004
f1 (ppm)

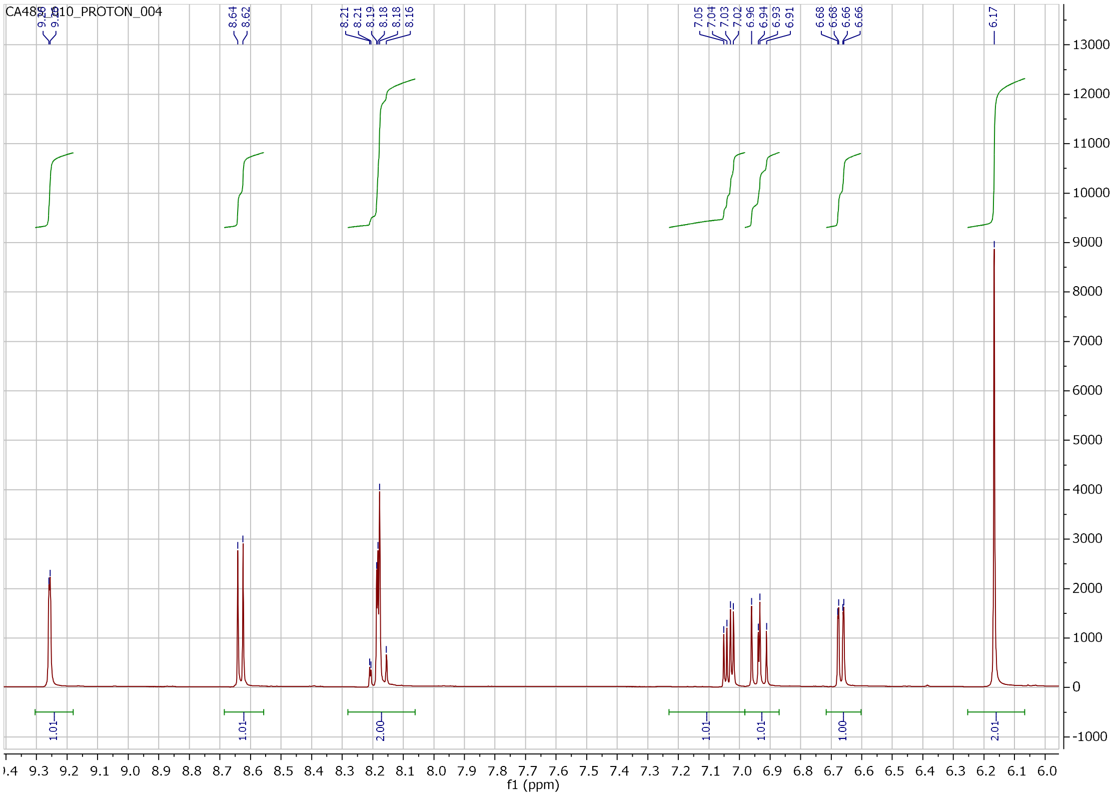
CA485_010_PROTON_004
f1 (ppm)

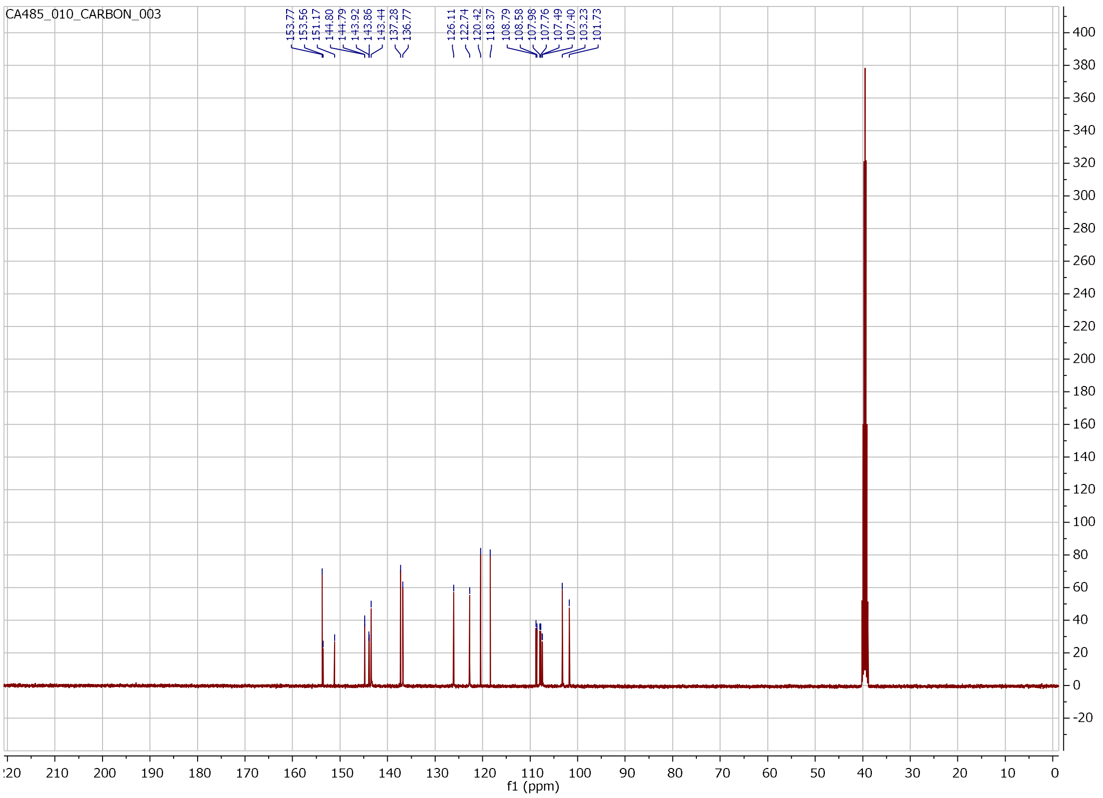
CA485_010_CARBON_003
153.77
153.56
151.17
144.80
144.79
143.92
143.86
143.44
137.28
136.77
126.11
122.74
120.42
118.37
108.79
108.58
107.98
107.76
107.49
107.40
103.23
101.73
f1 (ppm)

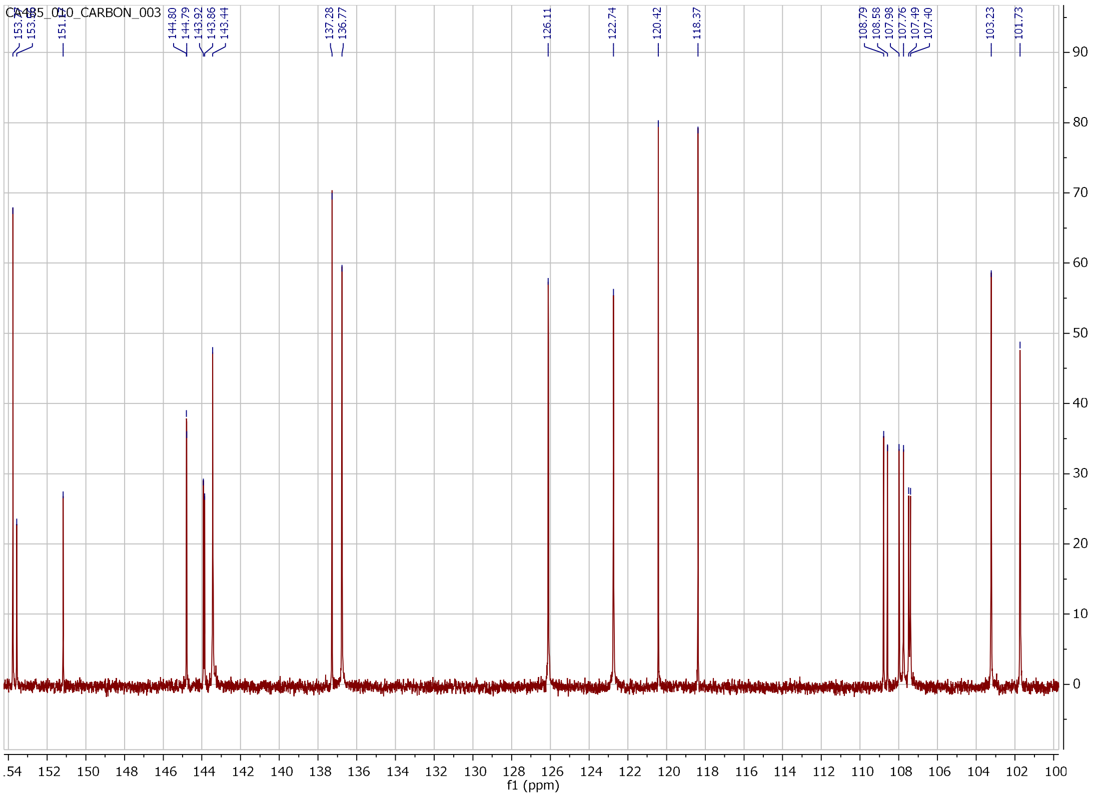
CA485_010_CARBON_003
144.80
144.79
143.92
143.86
143.44
137.28
136.77
126.11
122.74
120.42
118.37
108.79
108.58
107.98
107.76
107.49
107.40
103.23
101.73
f1 (ppm)

6-bromo-*N*-(2,3-dimethoxyphenyl)quinolin-4-amine (**15**)

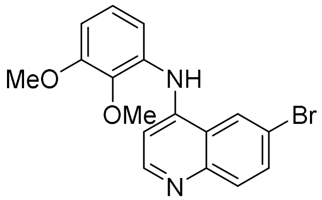

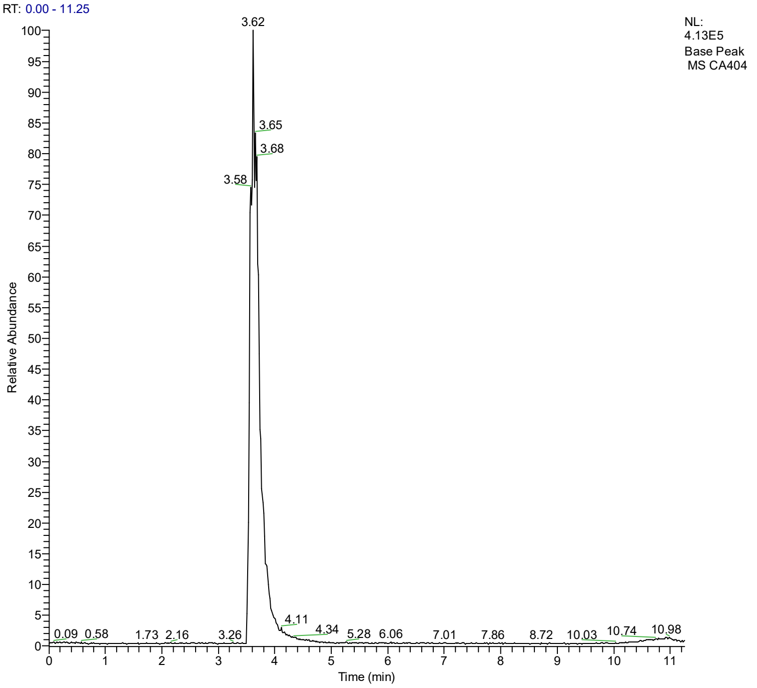

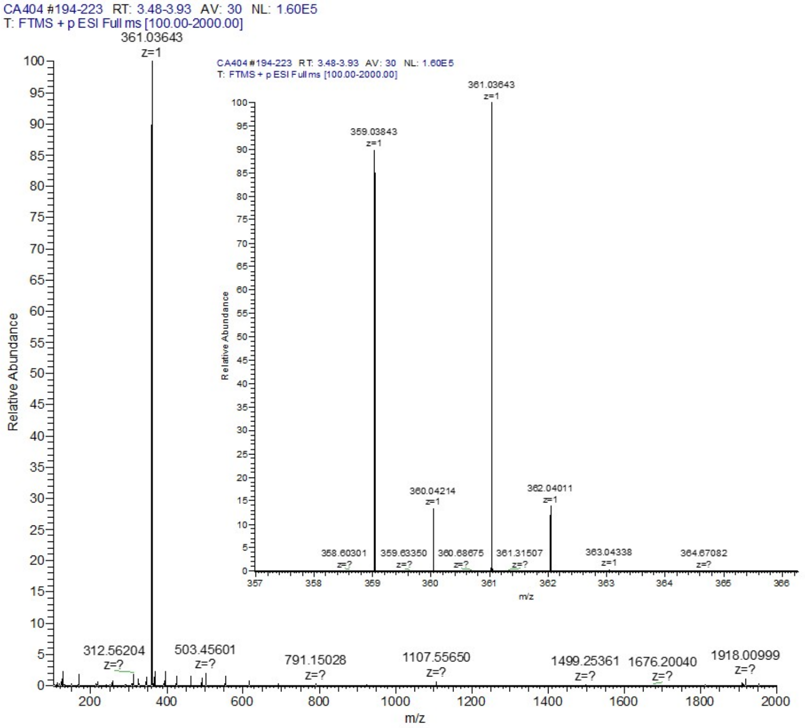

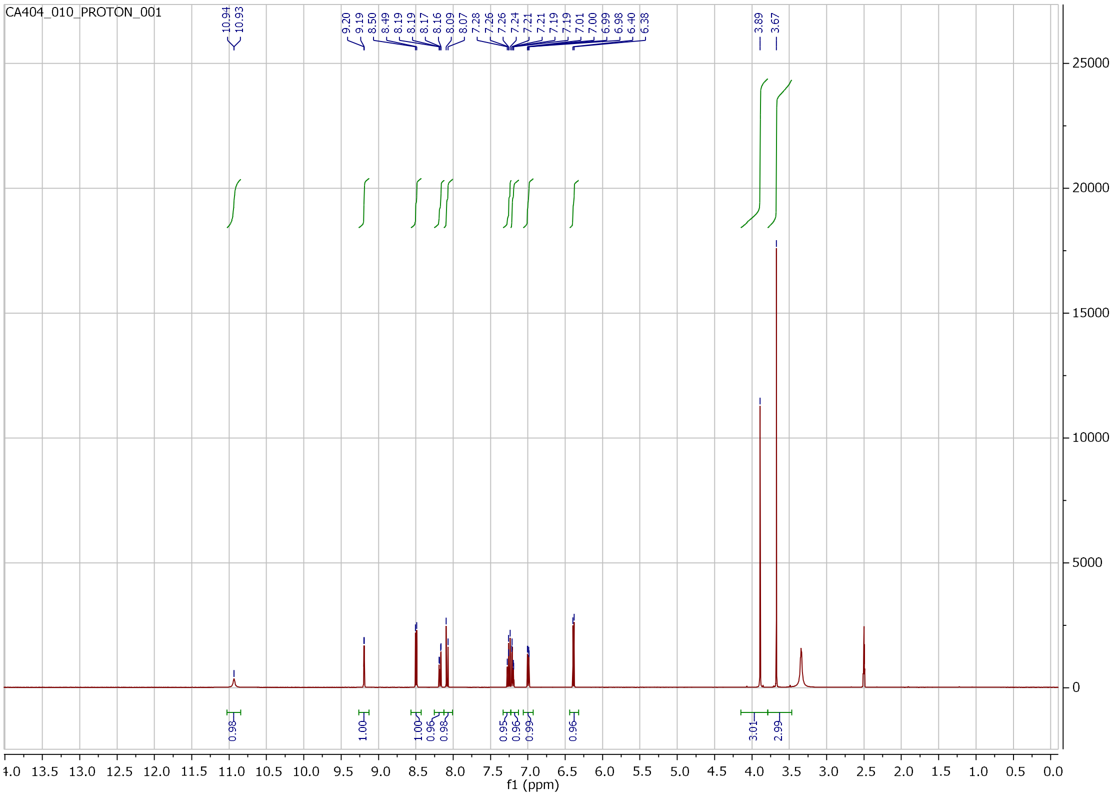
CA404_010_PROTON_001
f1 (ppm)

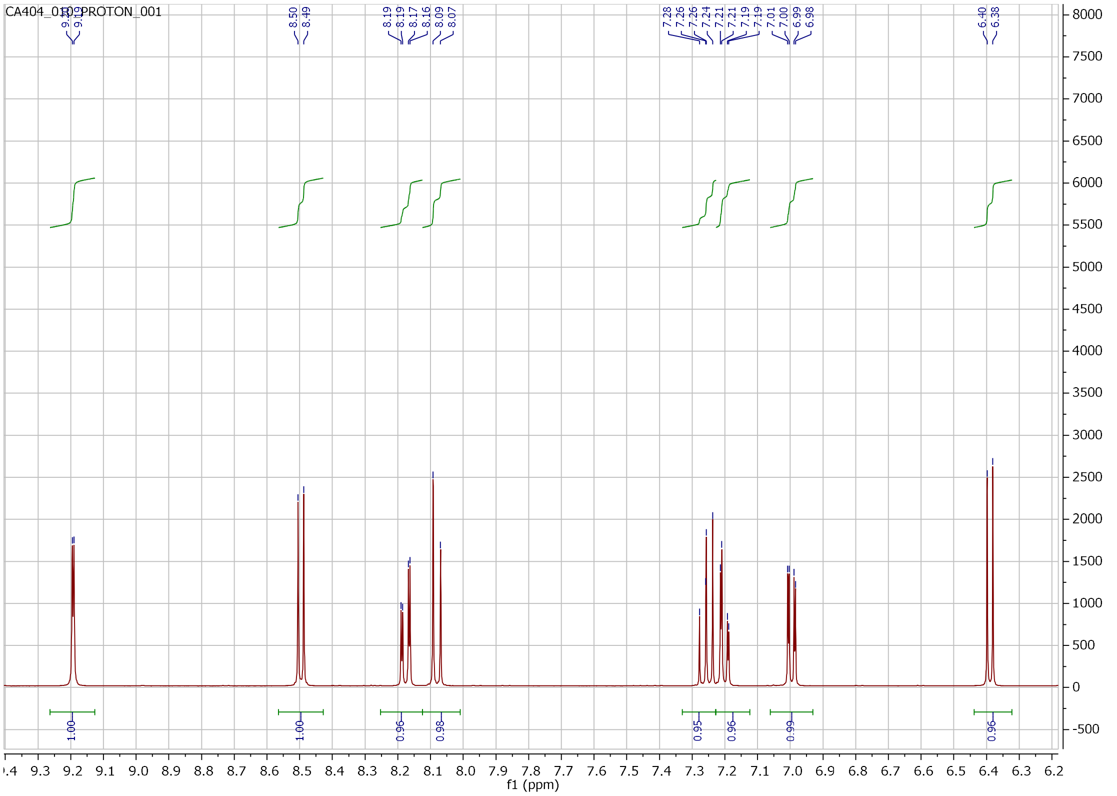
CA404_010_PROTON_001
f1 (ppm)

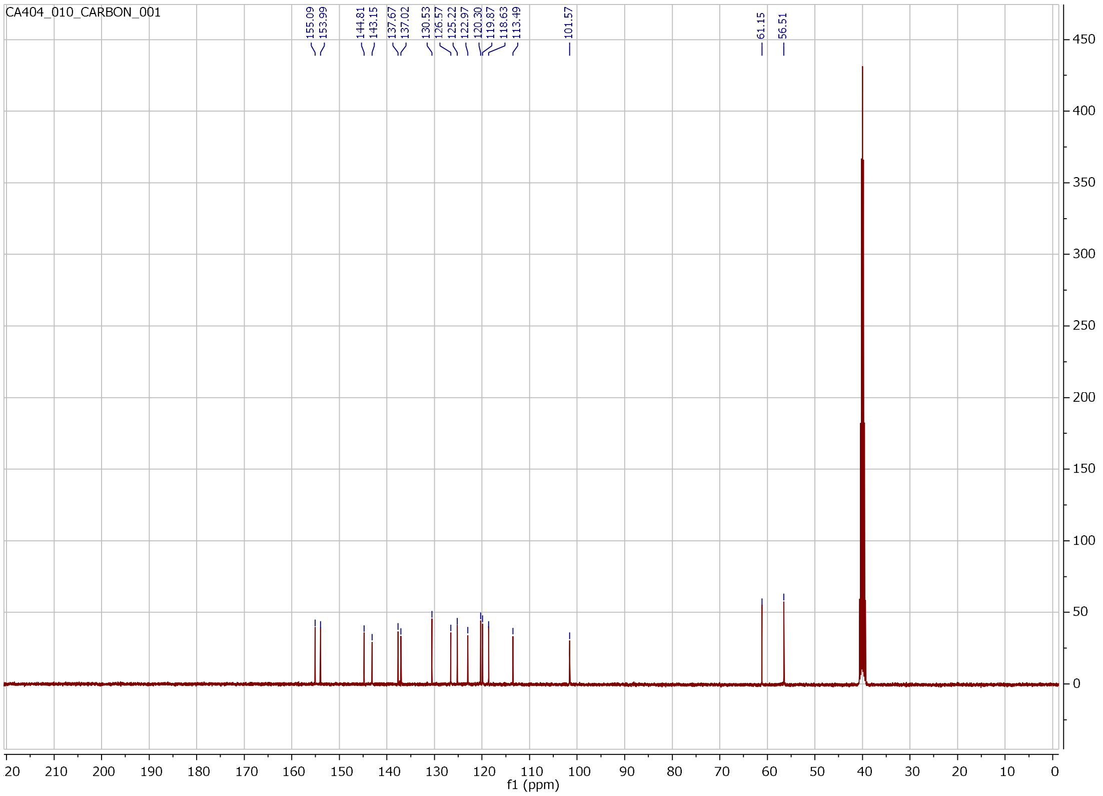
CA404_010_CARBON_001
155.09
153.99
144.81
143.15
137.67
137.02
130.53
126.57
125.22
122.97
120.30
119.87
118.63
113.49
101.57
61.15
56.51
f1 (ppm)

6-bromo-*N*-(2,2-difluorobenzo[*d*][1,3]dioxol-4-yl)quinolin-4-amine (**16**)

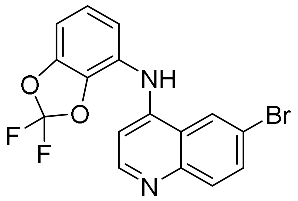

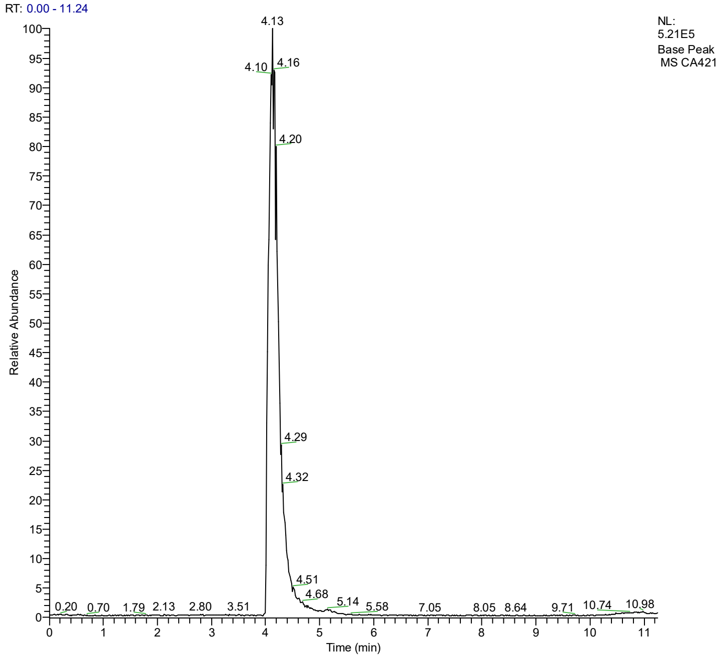

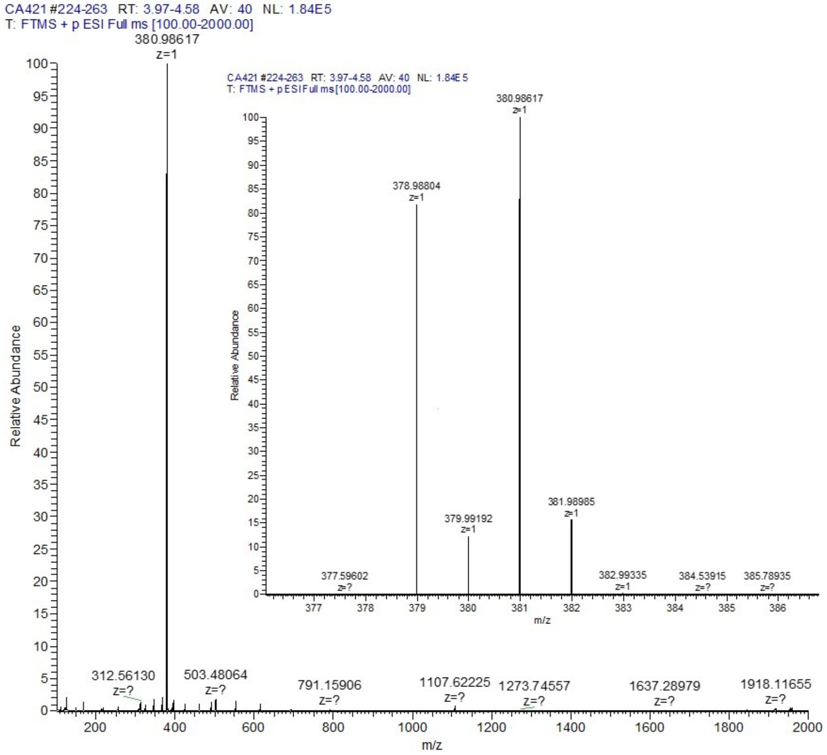

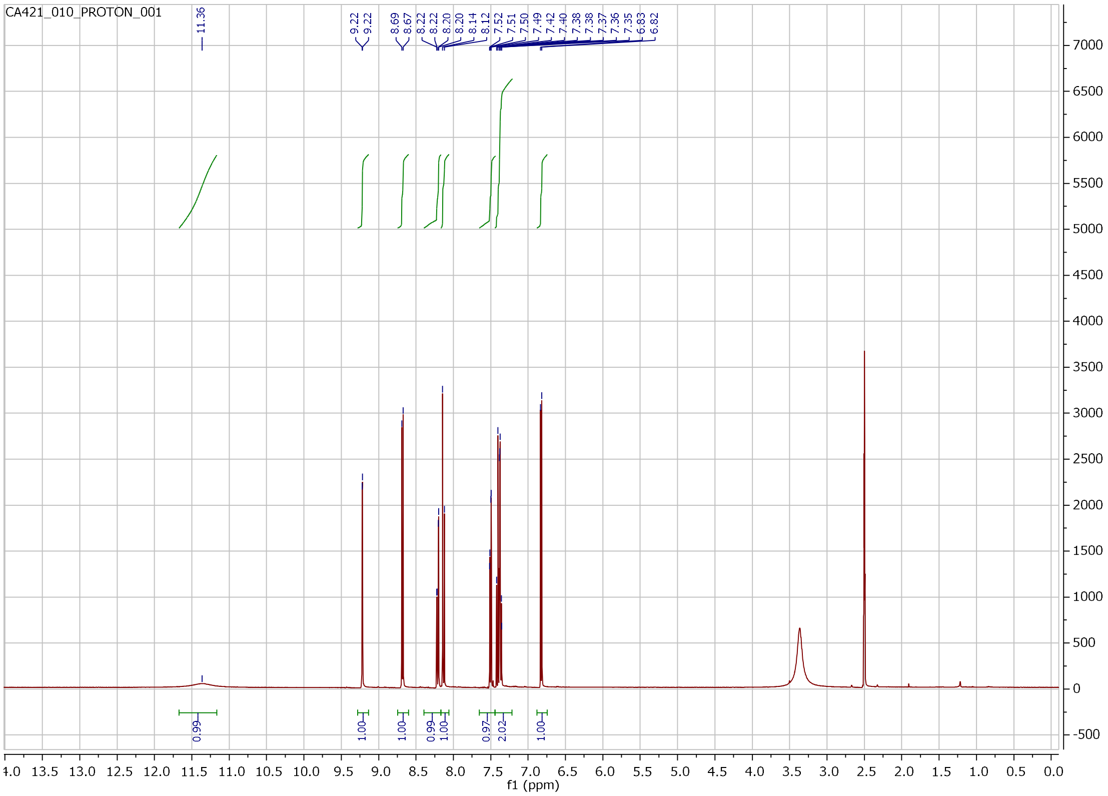
CA421_010_PROTON_001
11.36
9.22
9.22
8.69
8.67
8.22
8.22
8.20
8.20
8.14
8.12
7.52
7.51
7.50
7.49
7.42
7.40
7.38
7.38
7.37
7.36
7.35
6.83
6.82
0.99
1.00
1.00
0.99
1.00
0.97
2.02
1.00
f1 (ppm)

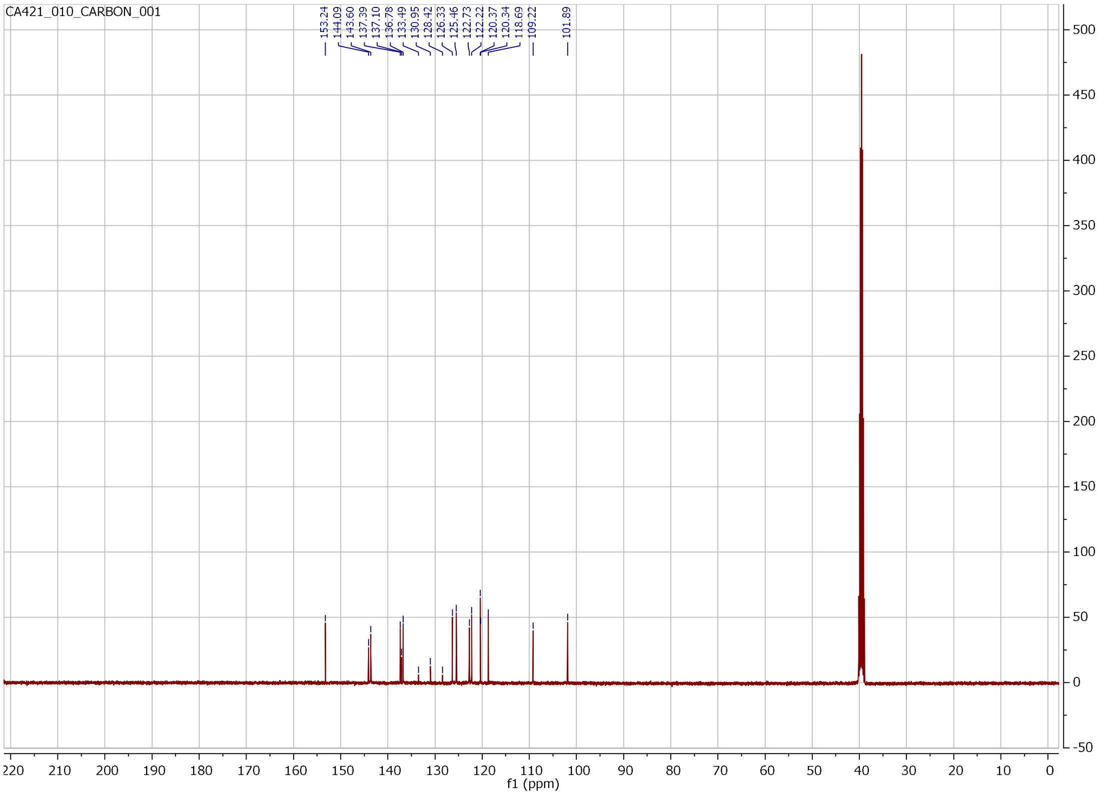
CA421_010_CARBON_001
153.24
144.09
143.60
137.39
137.10
136.78
133.49
130.95
128.42
126.33
125.46
122.73
122.22
120.37
120.34
118.69
109.22
101.89
f1 (ppm)

6-bromo-*N*-(2,3-dihydrobenzofuran-4-yl)quinolin-4-amine (**17**)

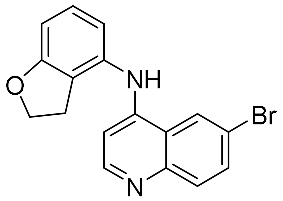

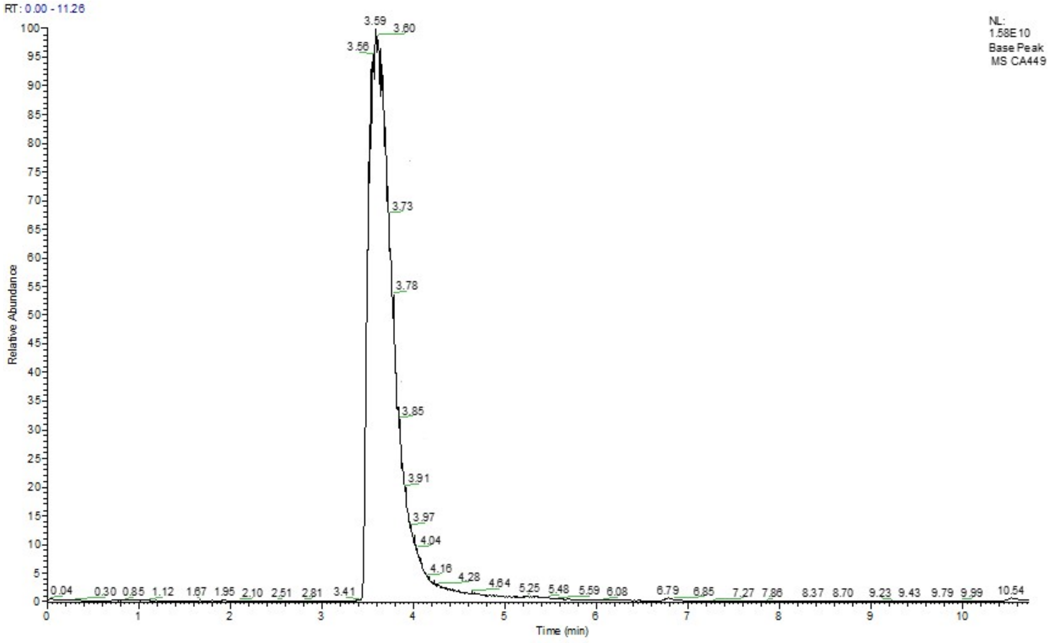

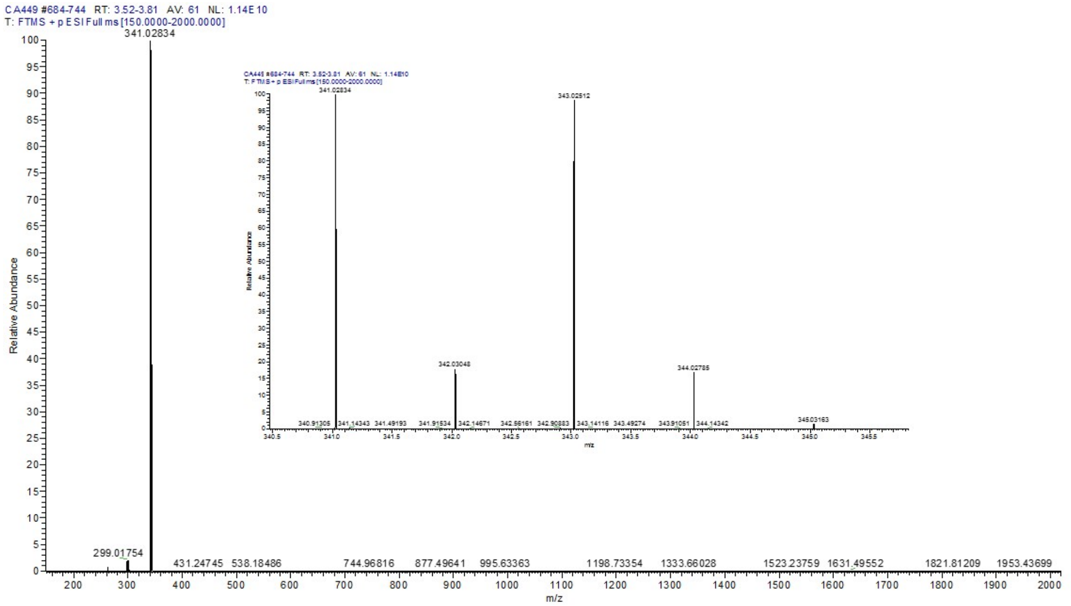

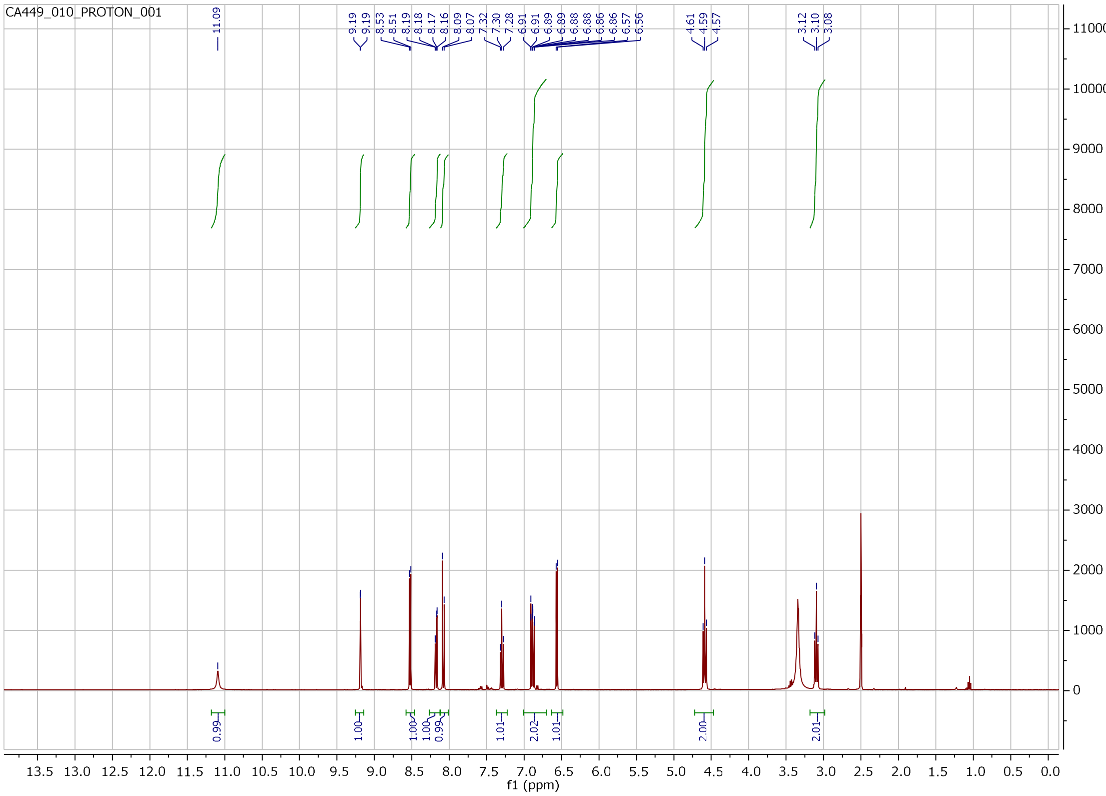
CA449_010_PROTON_001
11.09
9.19 9.19 8.53 8.51 8.19 8.18 8.17 8.16 8.09 8.07 7.32 7.30 7.28 6.91 6.91 6.89 6.89 6.88 6.88 6.86 6.86 6.57 6.56
4.61 4.59 4.57
3.12 3.10 3.08
0.99 1.00 1.00 1.00 0.99 1.01 2.02 1.01 2.00 2.01
f1 (ppm)

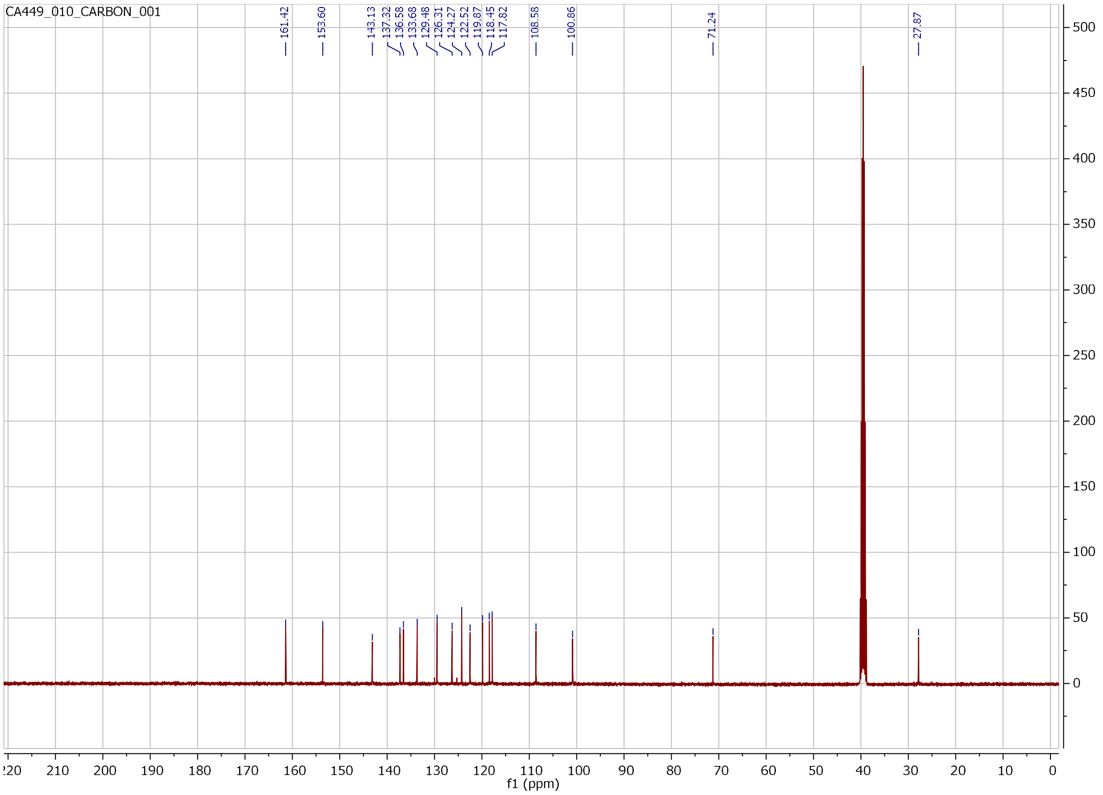
CA449_010_CARBON_001
161.42
153.60
143.13 137.32 136.58 133.68 129.48 126.31 124.27 122.52 119.87 118.45 117.82
108.58
100.86
71.24
27.87
f1 (ppm)

6-bromo-*N*-(2,3-dihydrobenzofuran-7-yl)quinolin-4-amine (**18**)

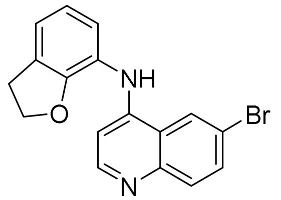

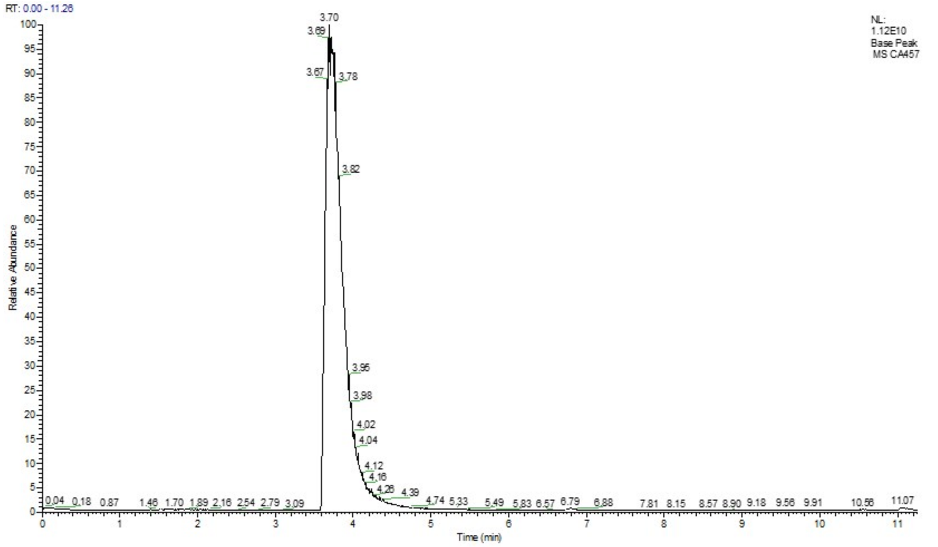

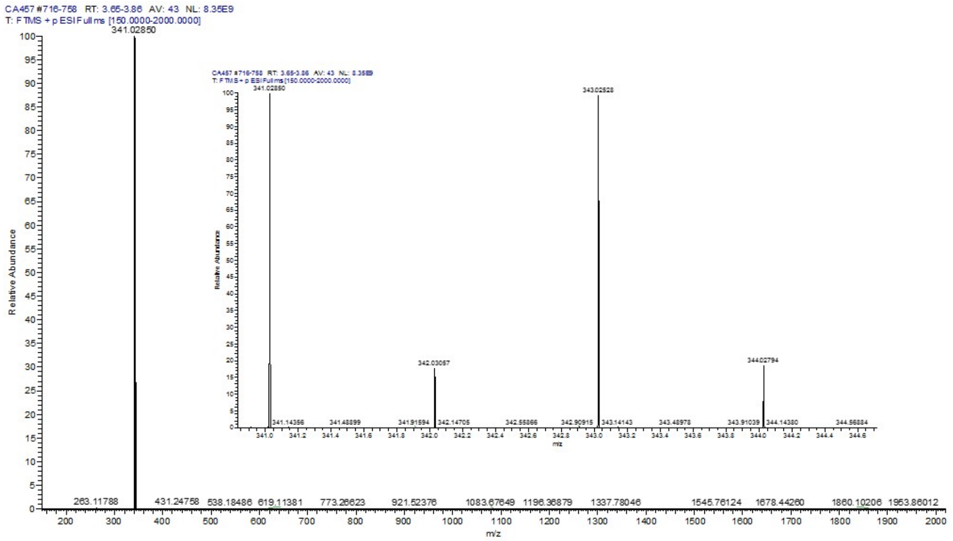

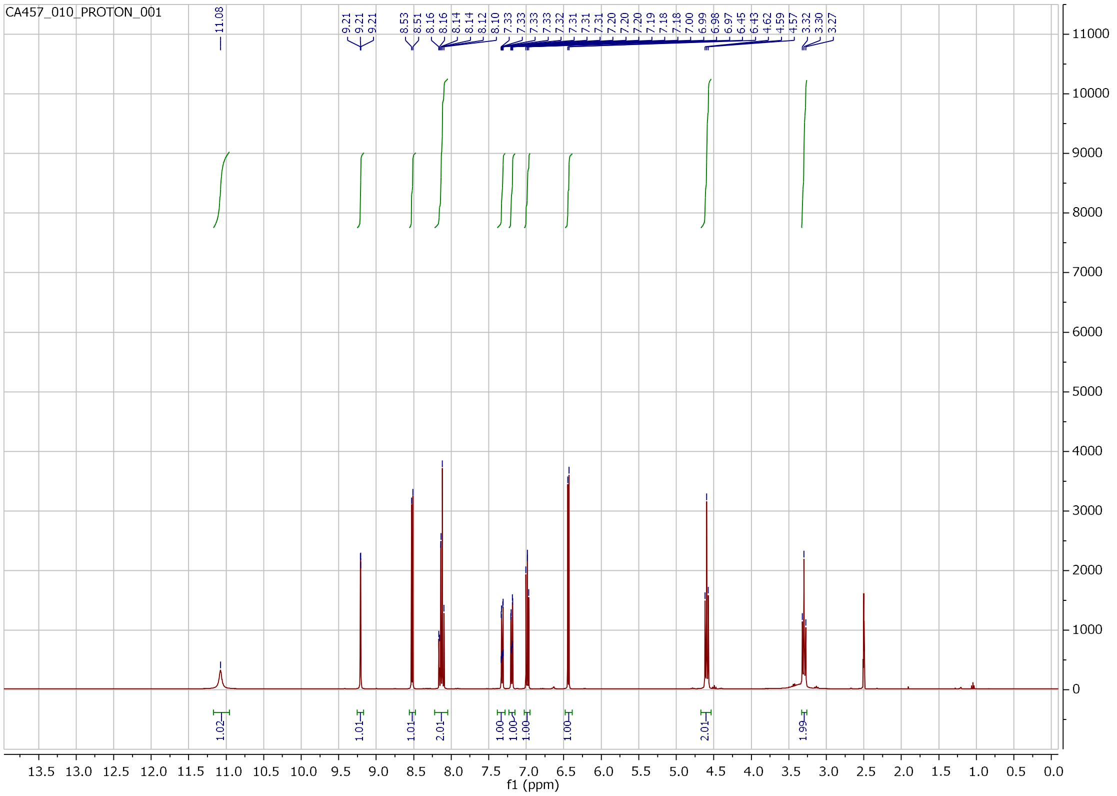
CA457_010_PROTON_001
f1 (ppm)

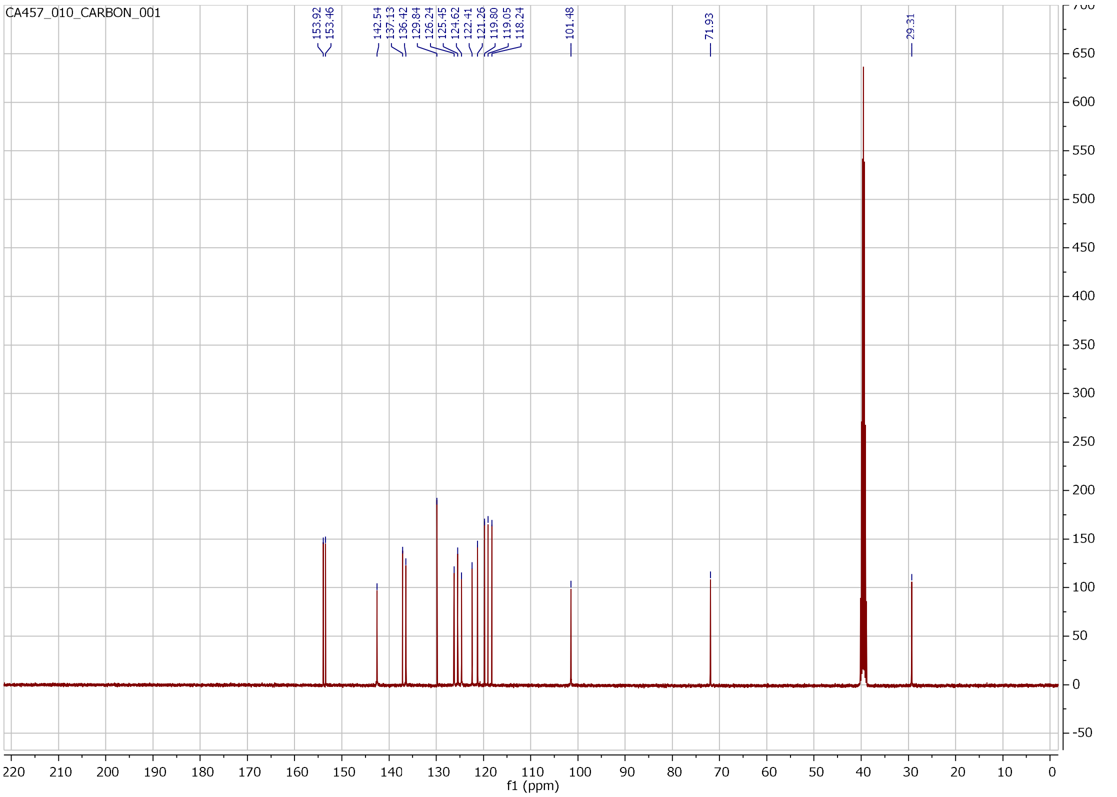
CA457_010_CARBON_001
f1 (ppm)

N-(benzofuran-4-yl)-6-bromoquinolin-4-amine (**19**)

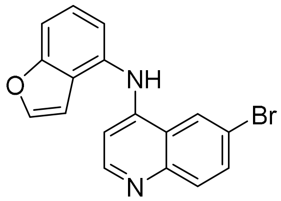

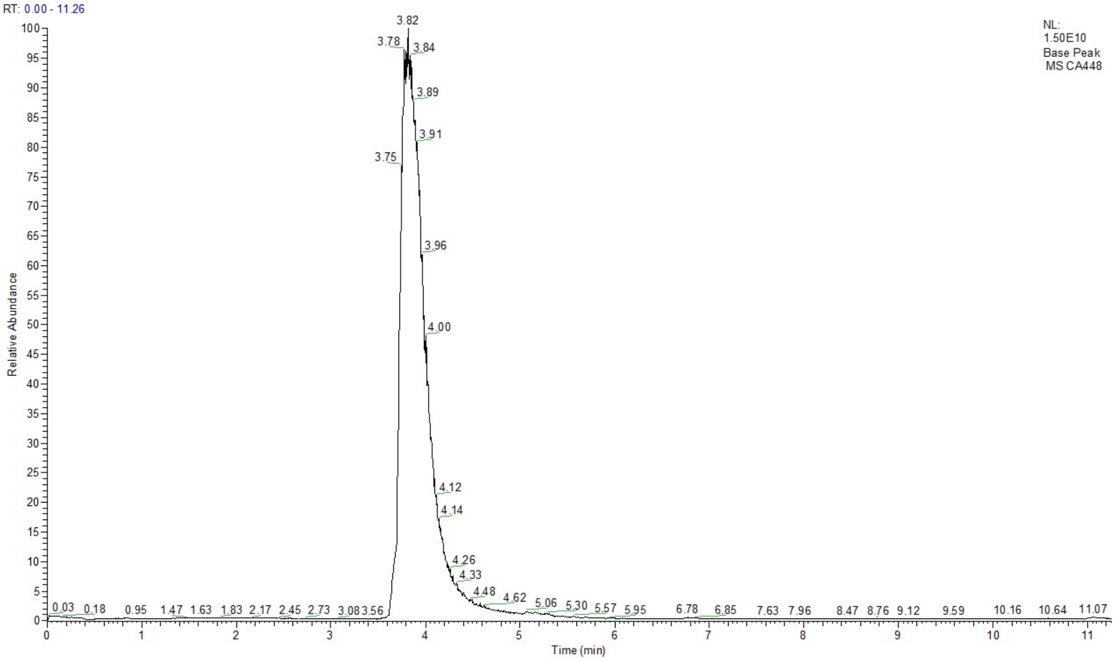

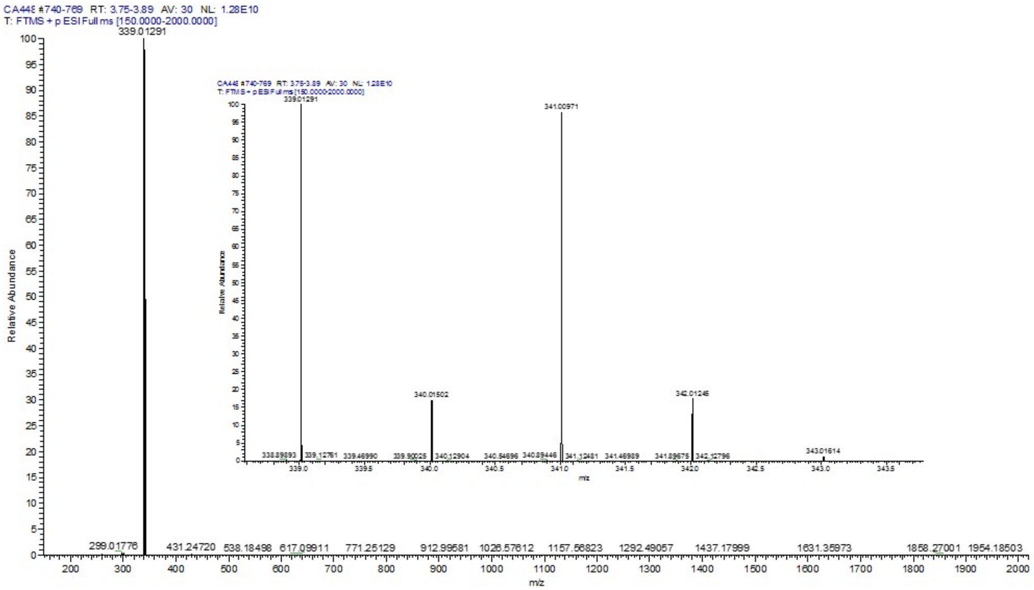

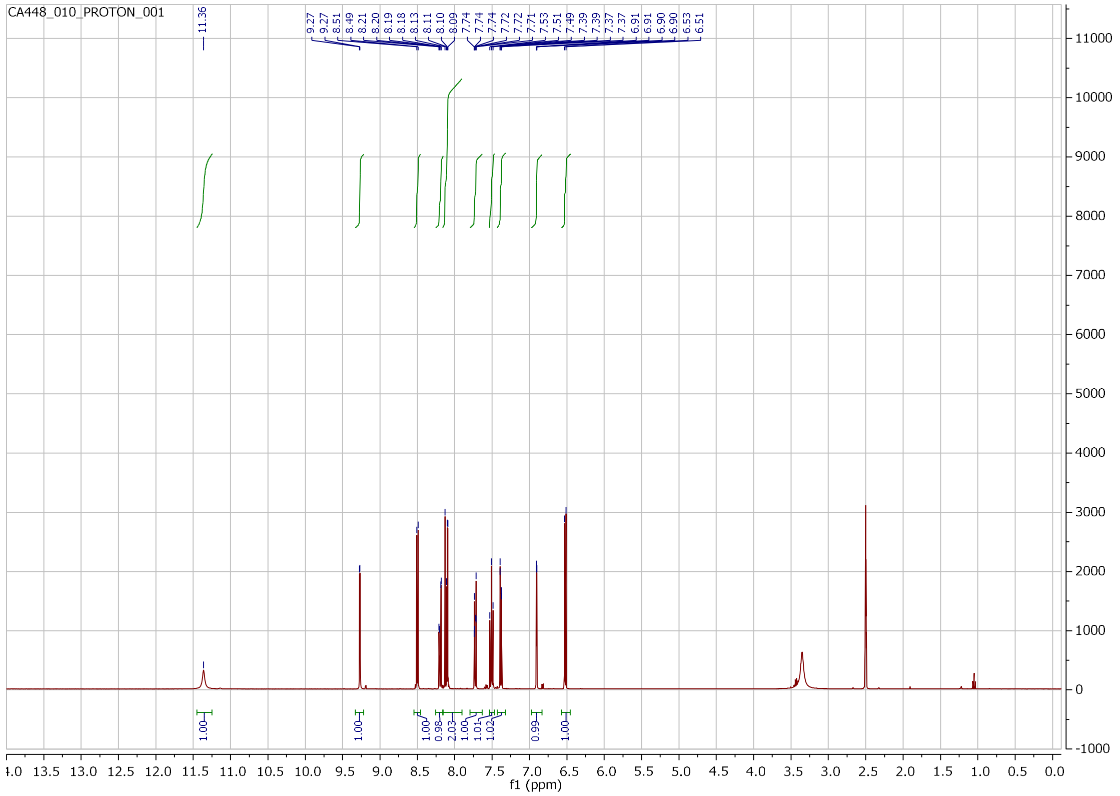
CA448_010_PROTON_001
f1 (ppm)

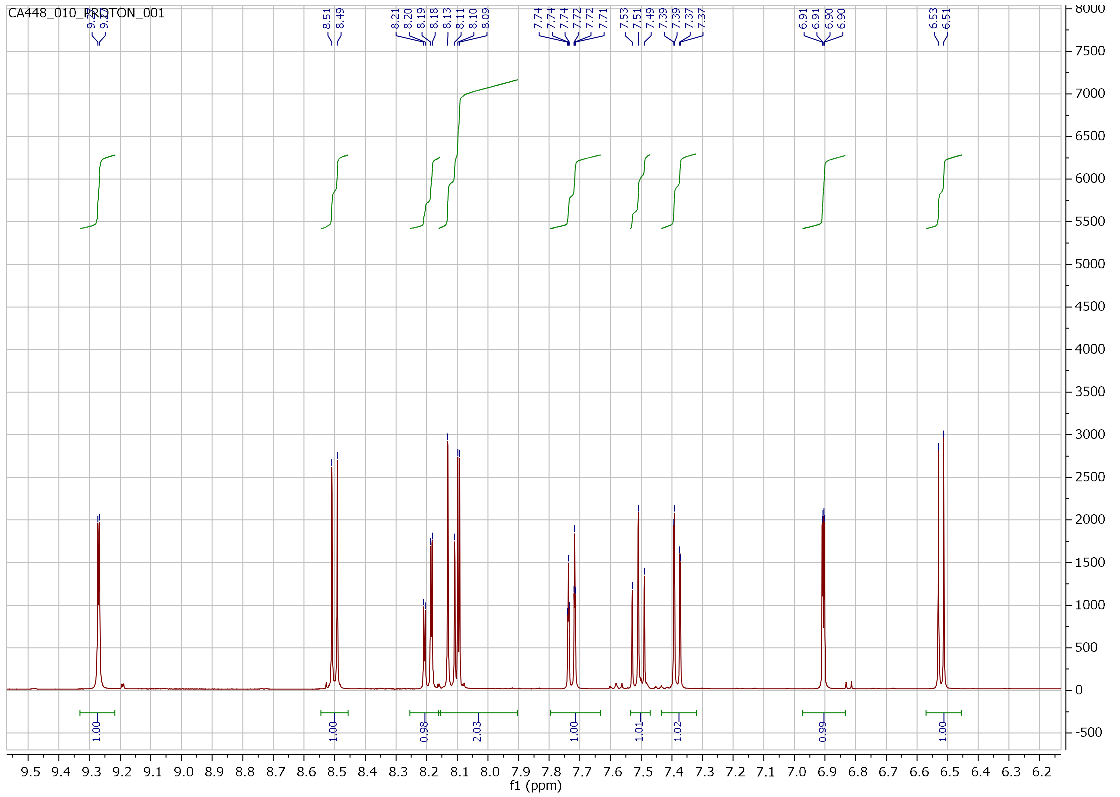
CA448_010_PROTON_001
f1 (ppm)

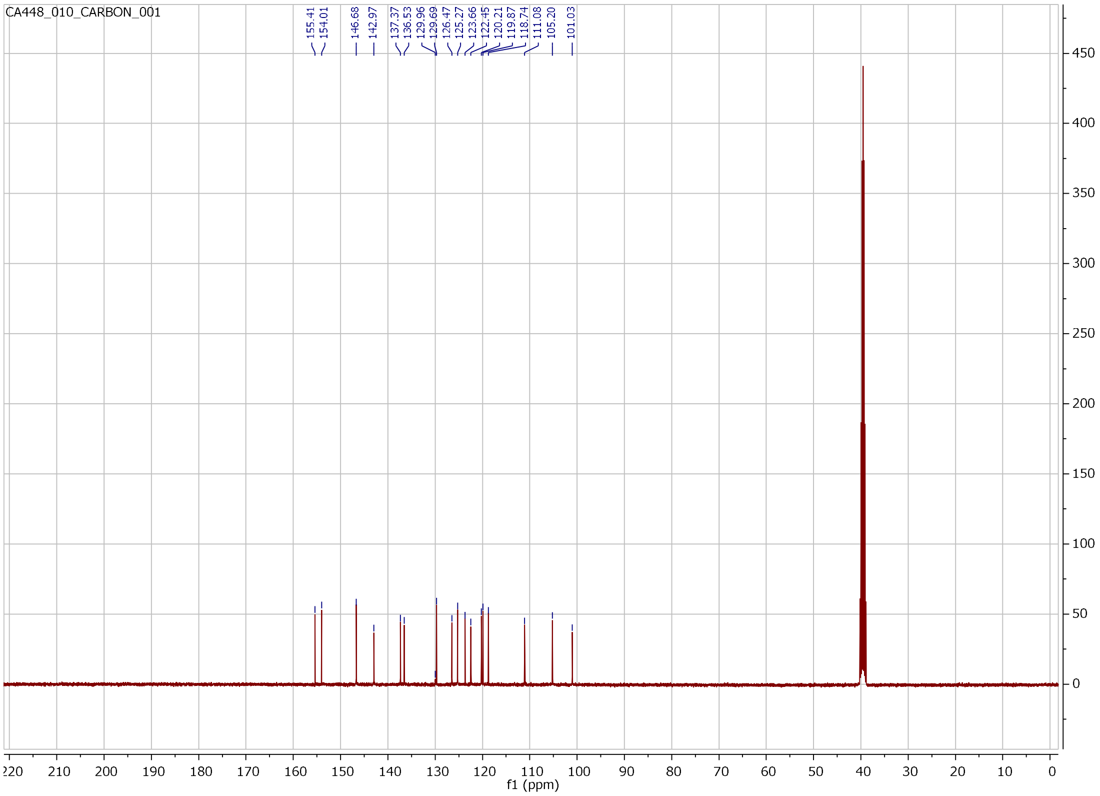
CA448_010_CARBON_001
155.41
154.01
146.68
142.97
137.37
136.53
129.96
129.69
126.47
125.27
123.66
122.45
120.21
119.87
118.74
111.08
105.20
101.03
f1 (ppm)

N-(benzofuran-7-yl)-6-bromoquinolin-4-amine (**20**)

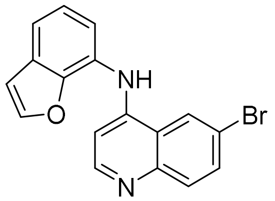

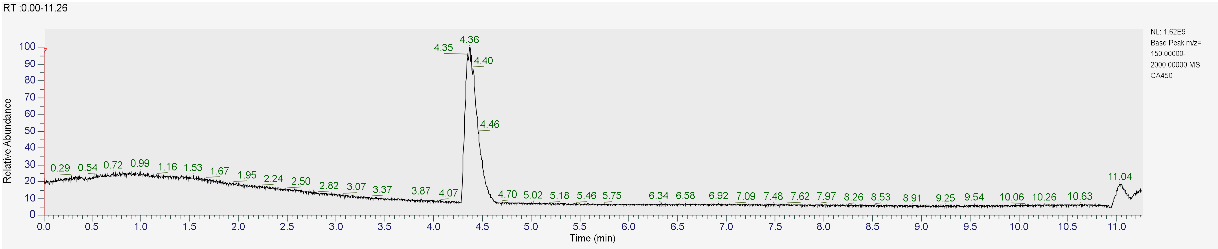

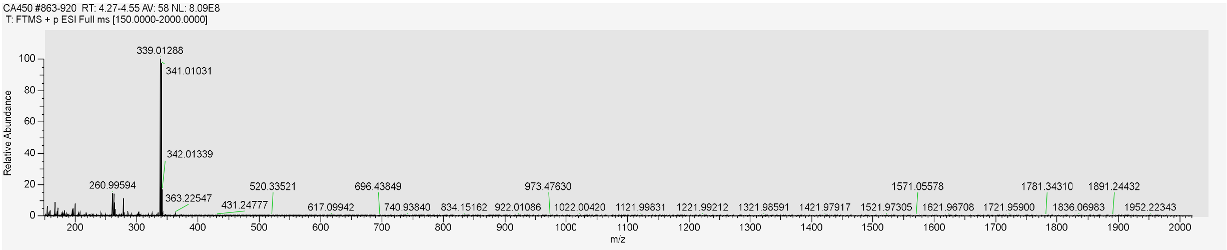

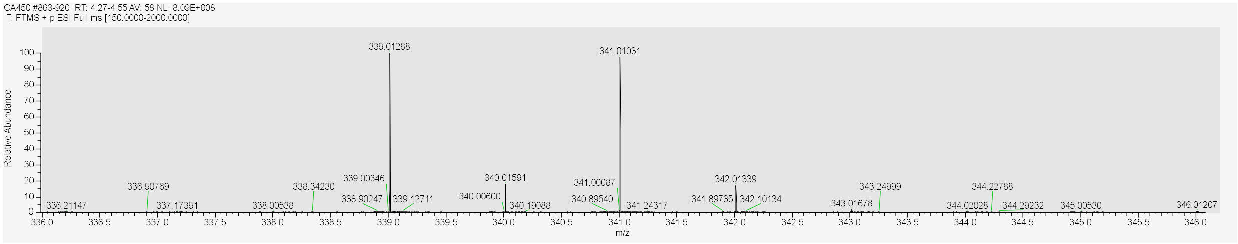

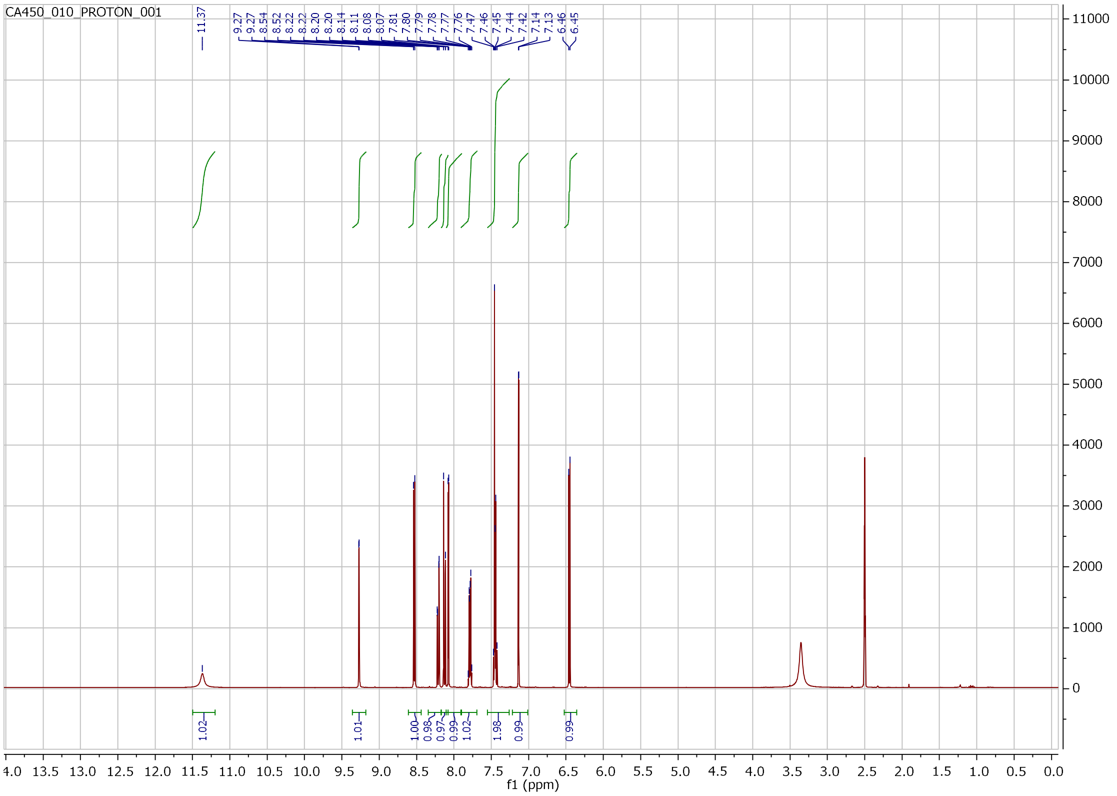
CA450_010_PROTON_001
11.37
9.27
9.27
8.54
8.52
8.22
8.22
8.20
8.20
8.14
8.11
8.08
8.07
7.81
7.80
7.79
7.78
7.77
7.76
7.47
7.46
7.45
7.44
7.42
7.14
7.13
6.46
6.45
1.02
1.01
1.00
0.98
0.97
0.99
1.02
1.98
0.99
0.99
f1 (ppm)

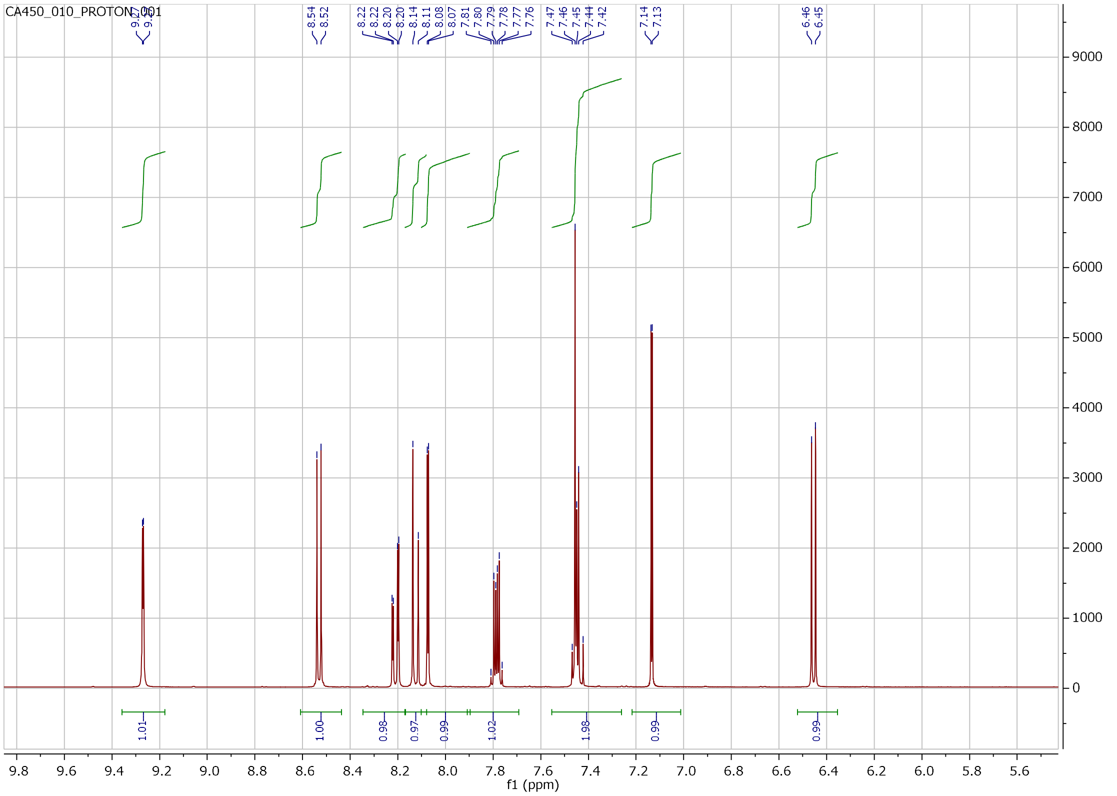
CA450_010_PROTON_001
8.54
8.52
8.22
8.22
8.20
8.20
8.14
8.11
8.08
8.07
7.81
7.80
7.79
7.78
7.77
7.76
7.47
7.46
7.45
7.44
7.42
7.14
7.13
6.46
6.45
1.01
1.00
0.98
0.97
0.99
1.02
1.98
0.99
0.99
f1 (ppm)

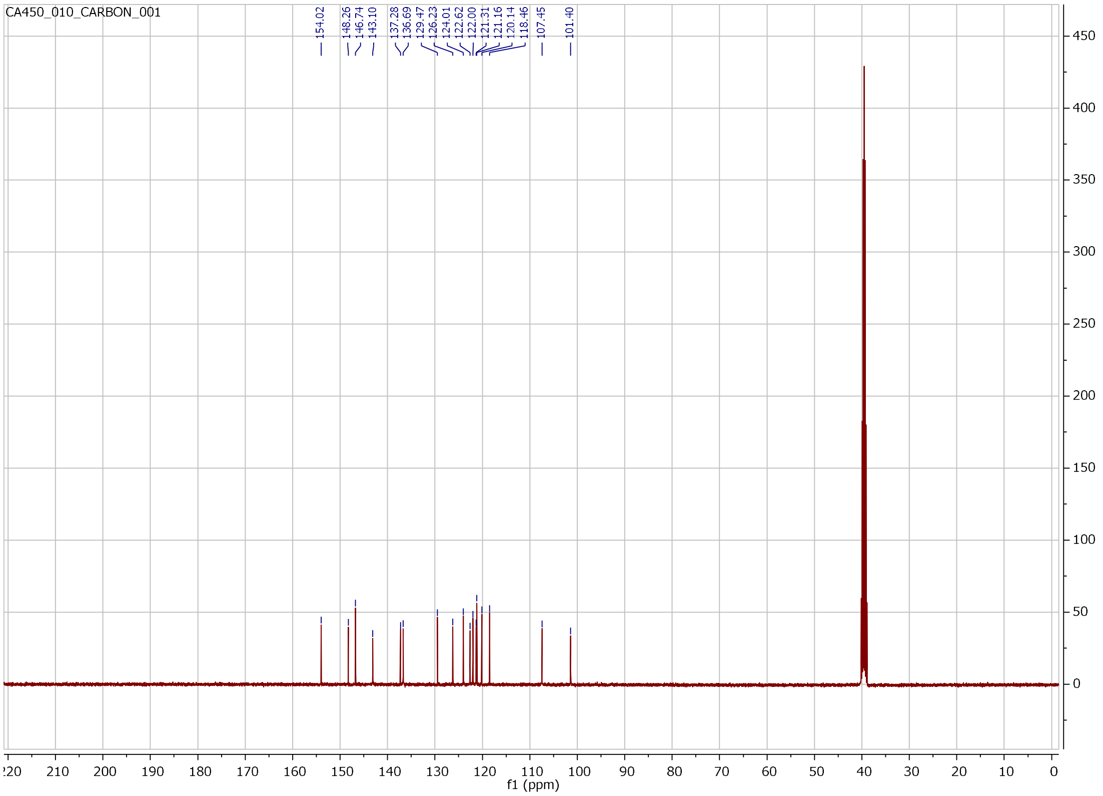

CA450_010_CARBON_001
154.02
148.26
146.74
143.10
137.28
136.69
129.47
126.23
124.01
122.62
122.00
121.31
121.16
120.14
118.46
107.45
101.40
f1 (ppm)

N-(6-bromoquinolin-4-yl)benzo[*d*]isoxazol-7-amine (**21**)

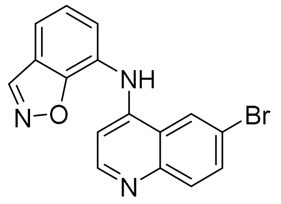

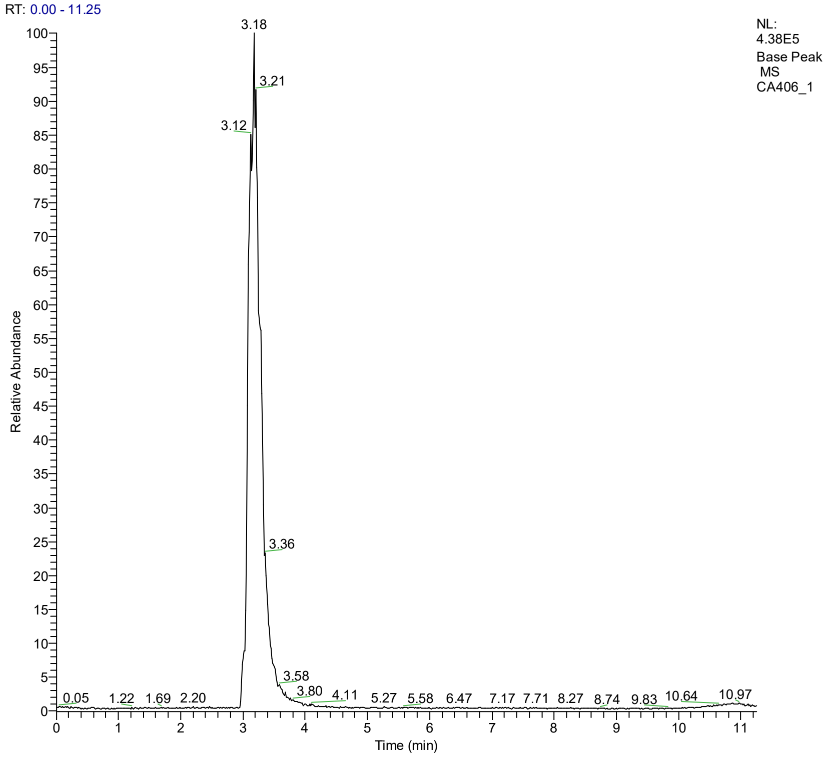

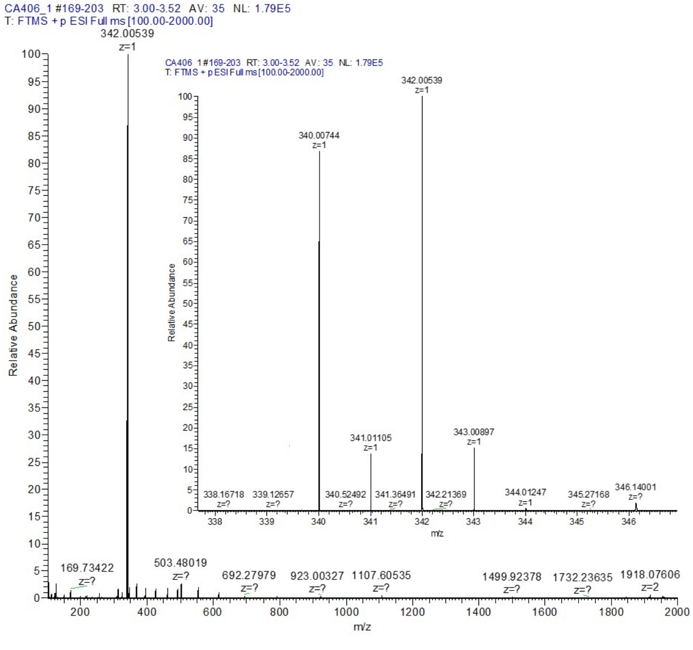

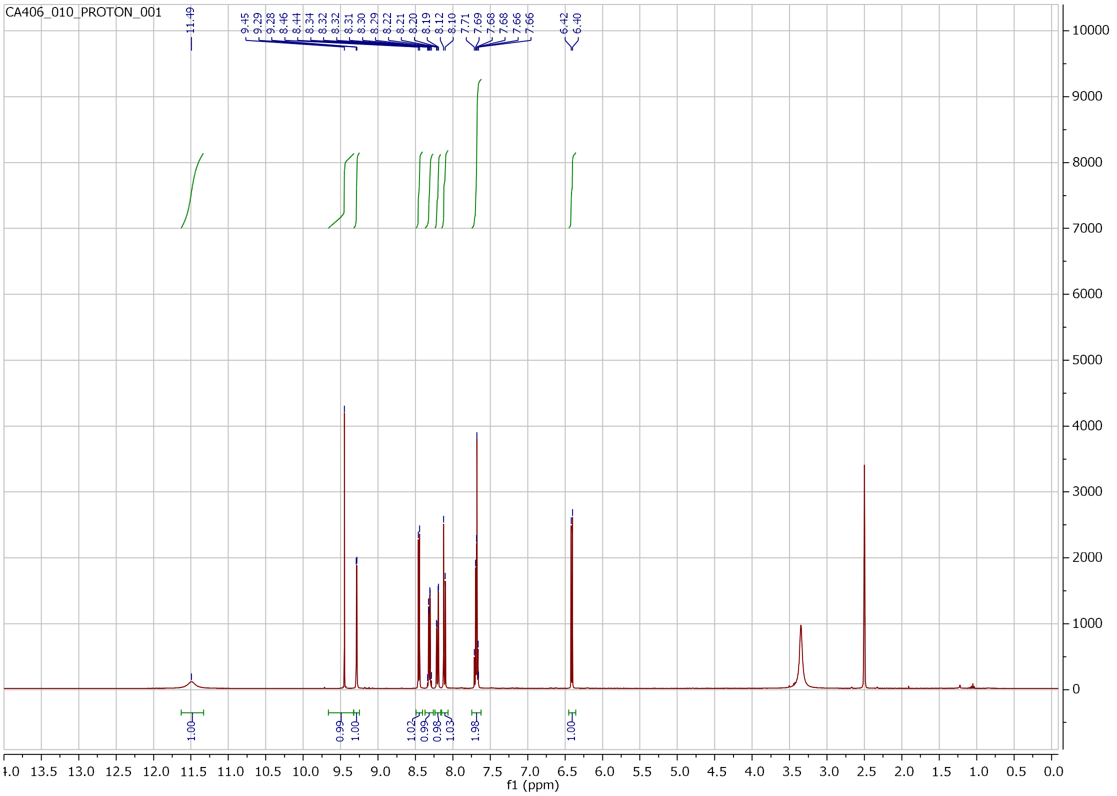
CA406_010_PROTON_001
f1 (ppm)

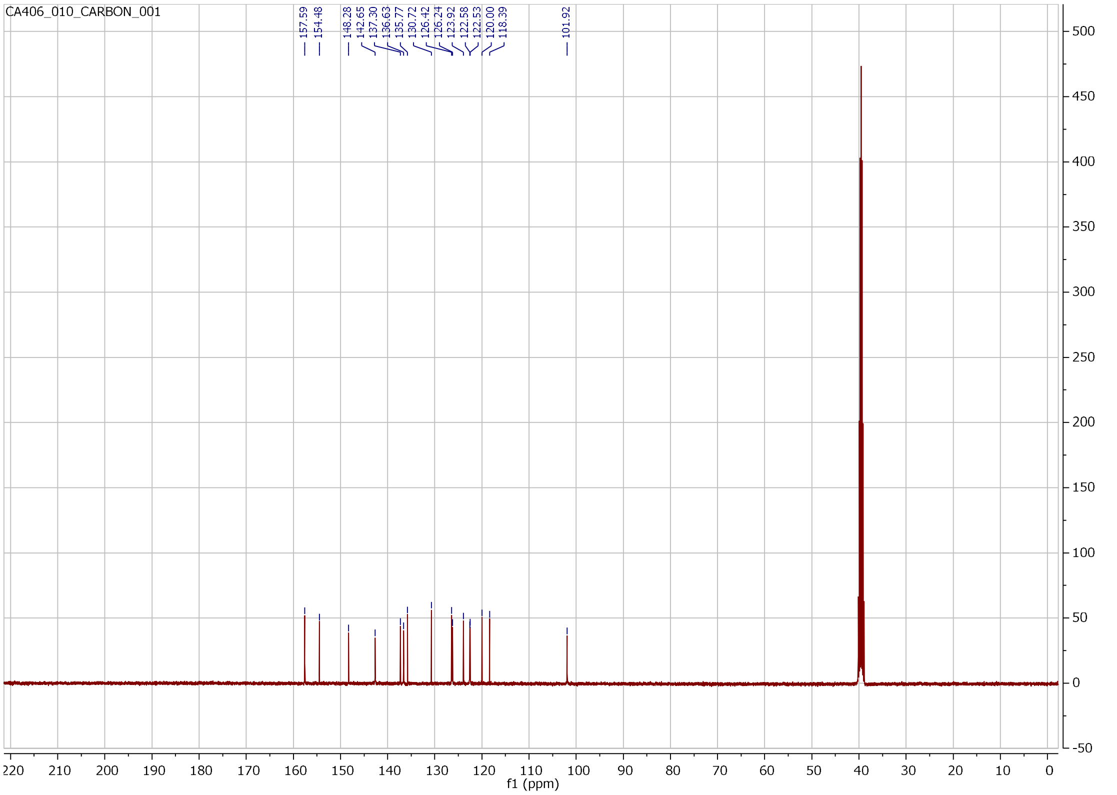
CA406_010_CARBON_001
f1 (ppm)

N-(6-bromoquinolin-4-yl)benzo[d]isoxazol-4-amine (**22**)

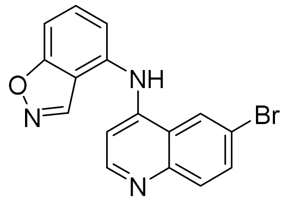

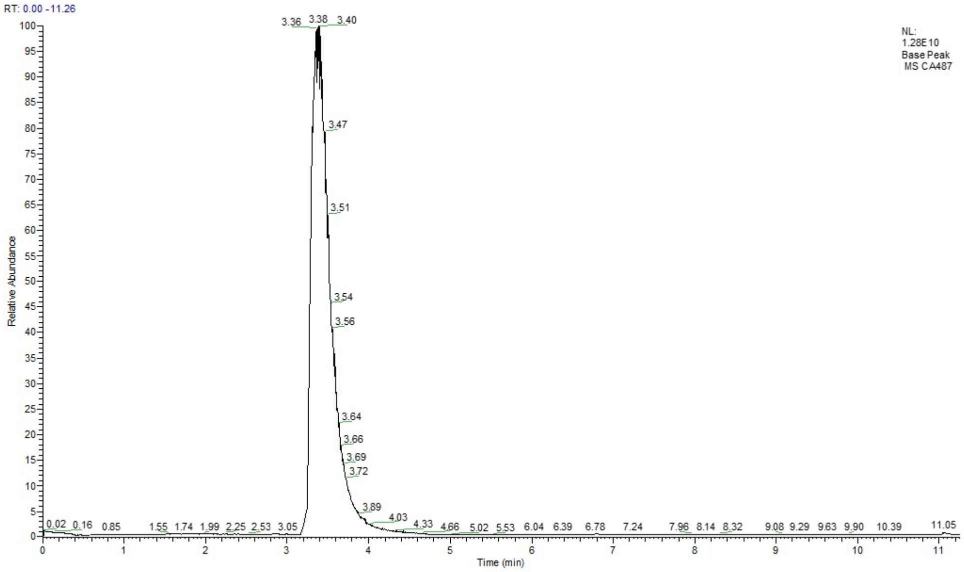

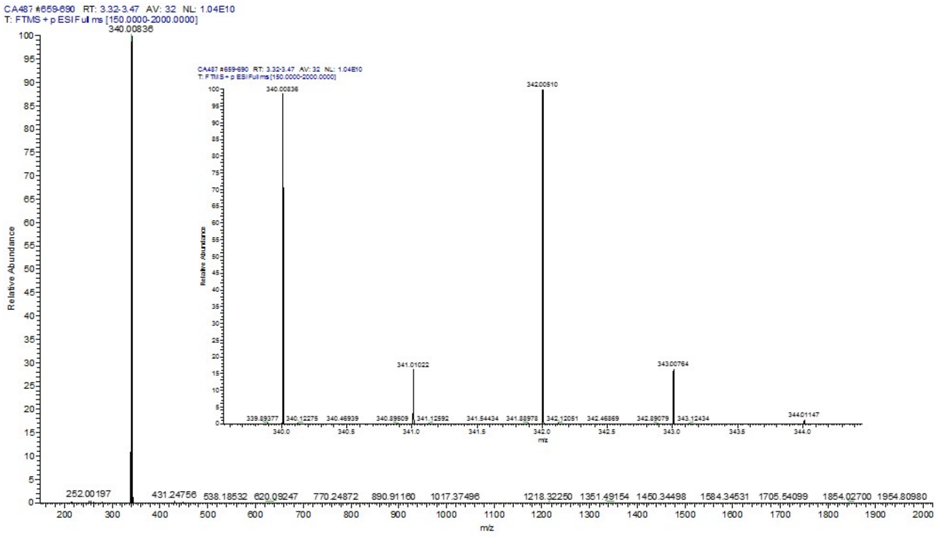

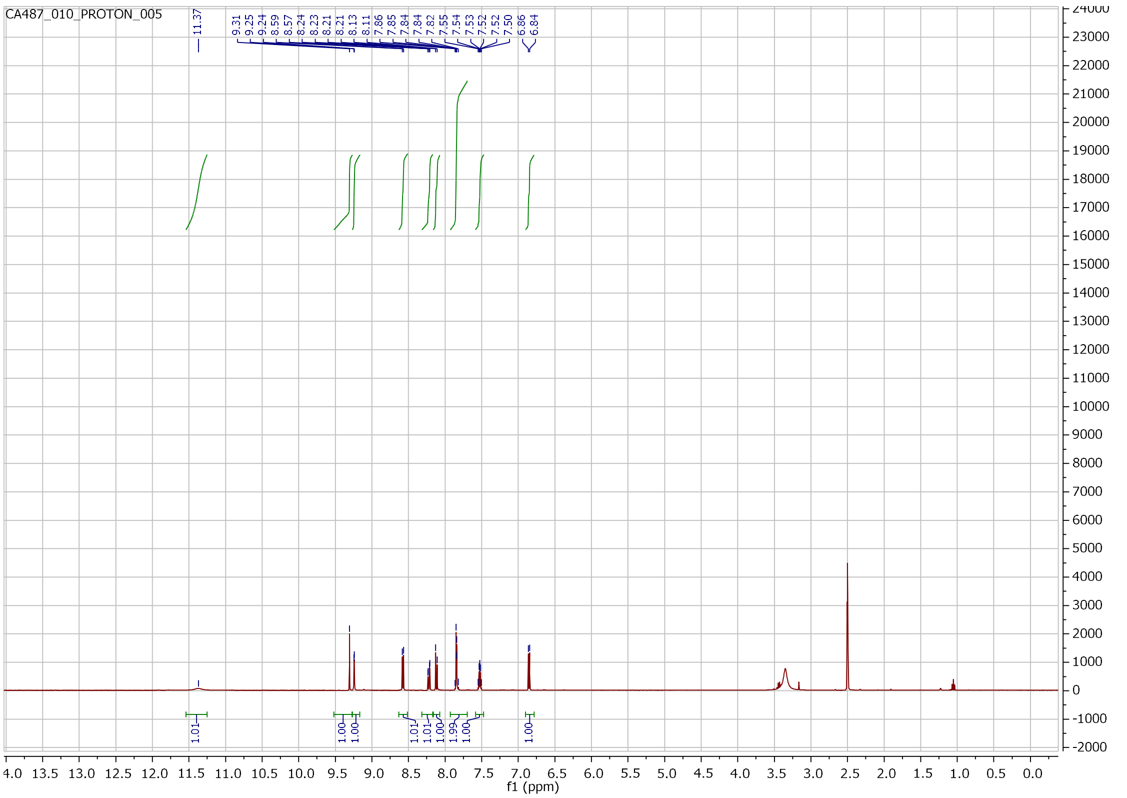
CA487_010_PROTON_005
f1 (ppm)

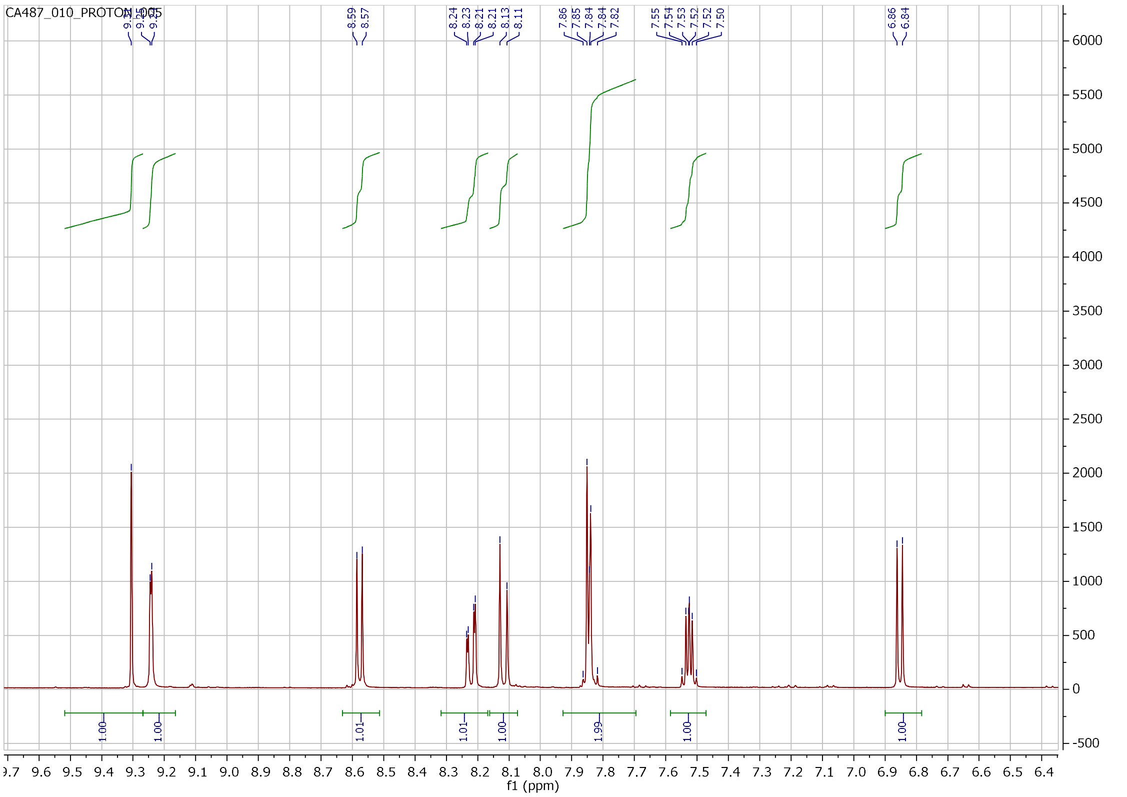
CA487_010_PROTON_005
f1 (ppm)

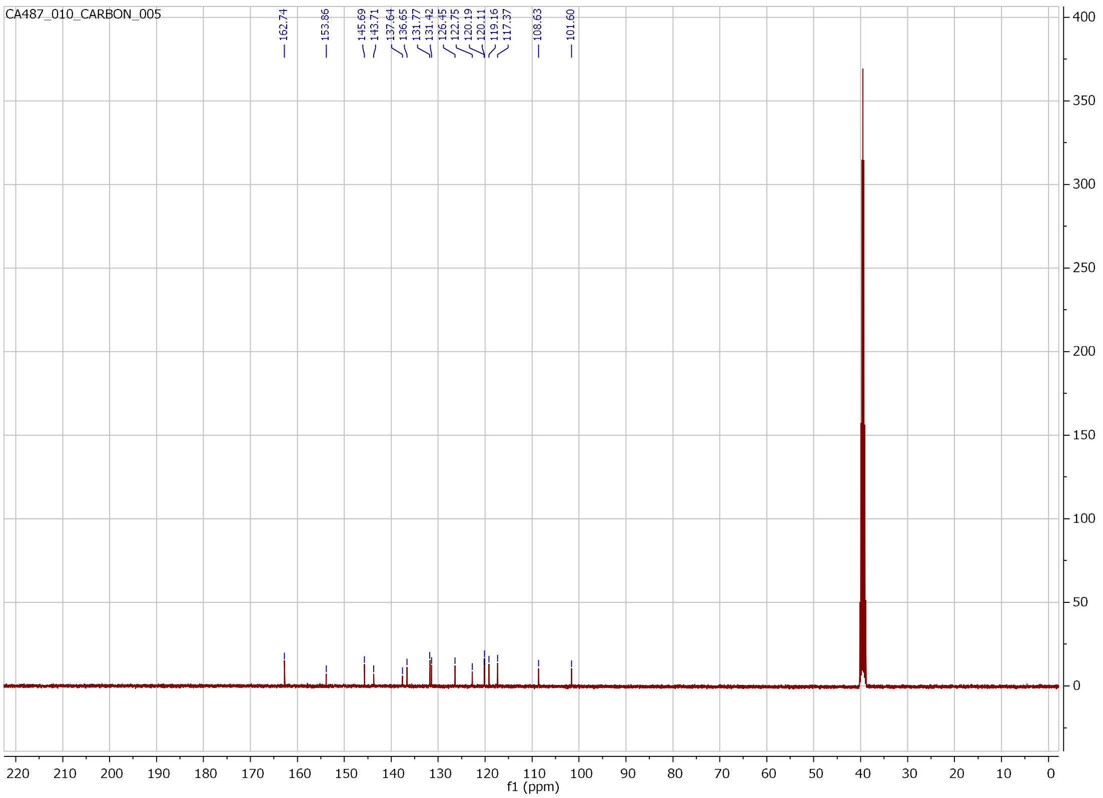

CA487_010_CARBON_005
162.74
153.86
145.69
143.71
137.64
136.65
131.77
131.42
126.45
122.75
120.19
120.11
119.16
117.37
108.63
101.60
f1 (ppm)

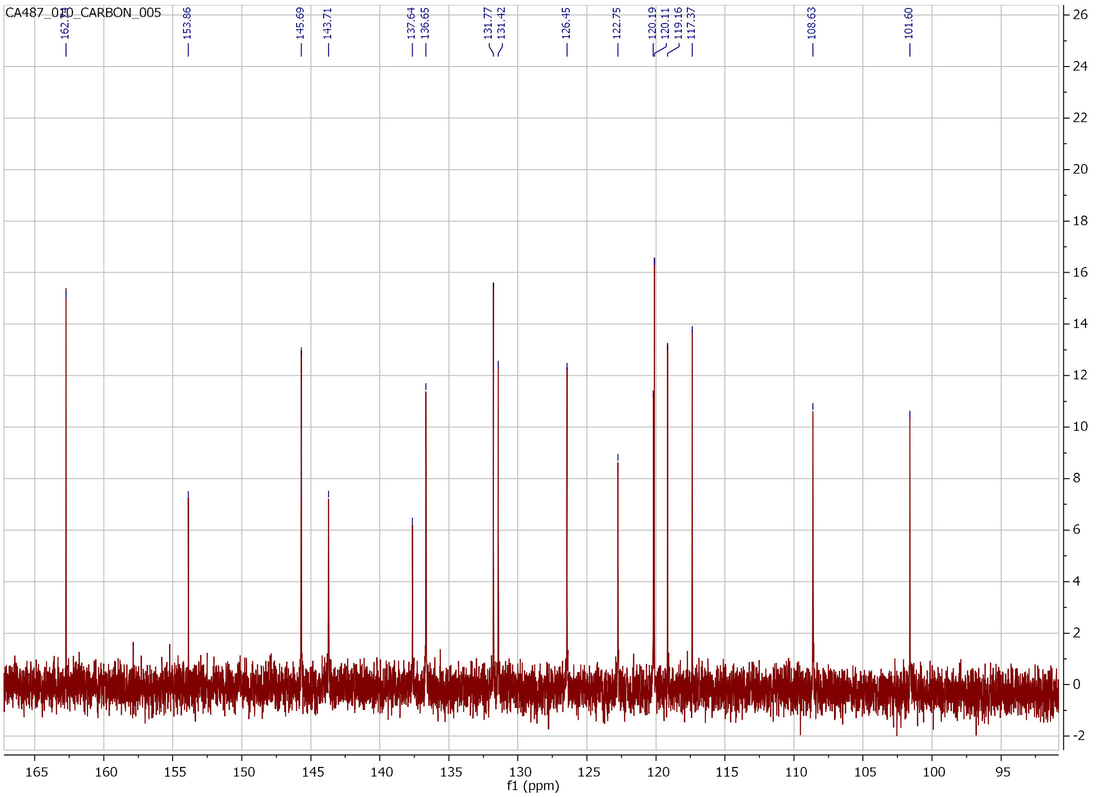

CA487_010_CARBON_005
162.74
153.86
145.69
143.71
137.64
136.65
131.77
131.42
126.45
122.75
120.19
120.11
119.16
117.37
108.63
101.60
f1 (ppm)

N-(6-bromoquinolin-4-yl)benzo[*d*]oxazol-7-amine (**23**)

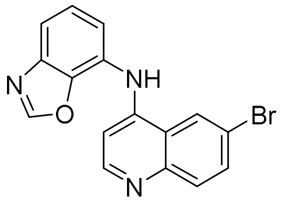

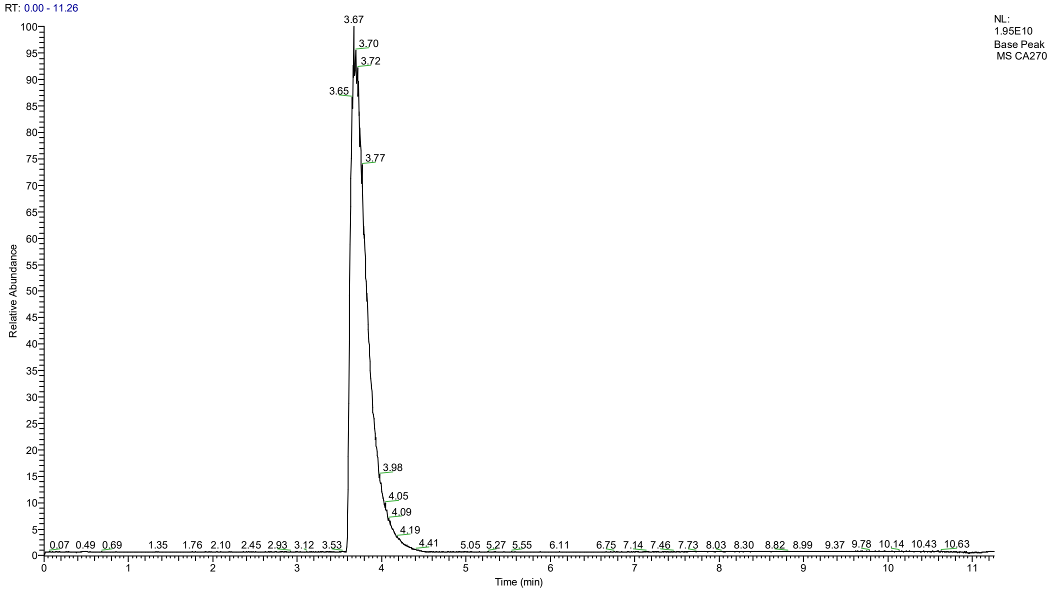

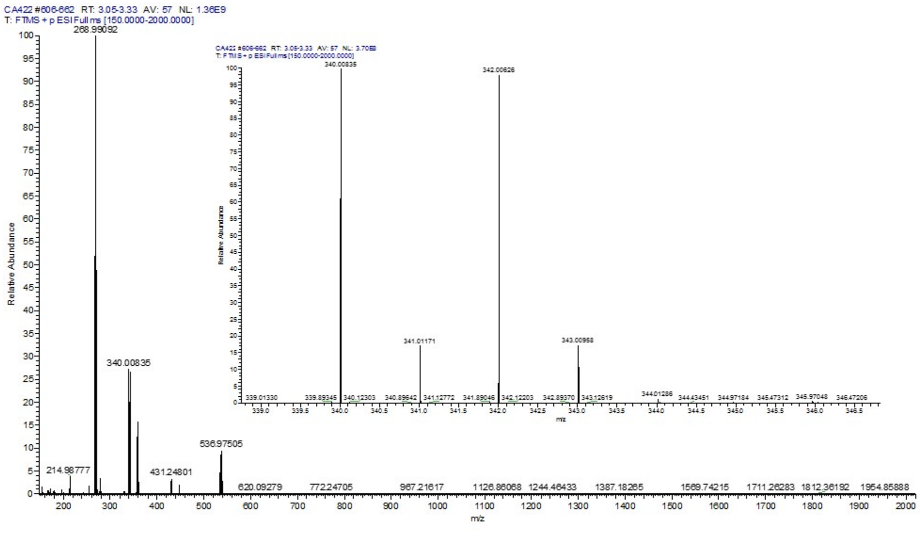

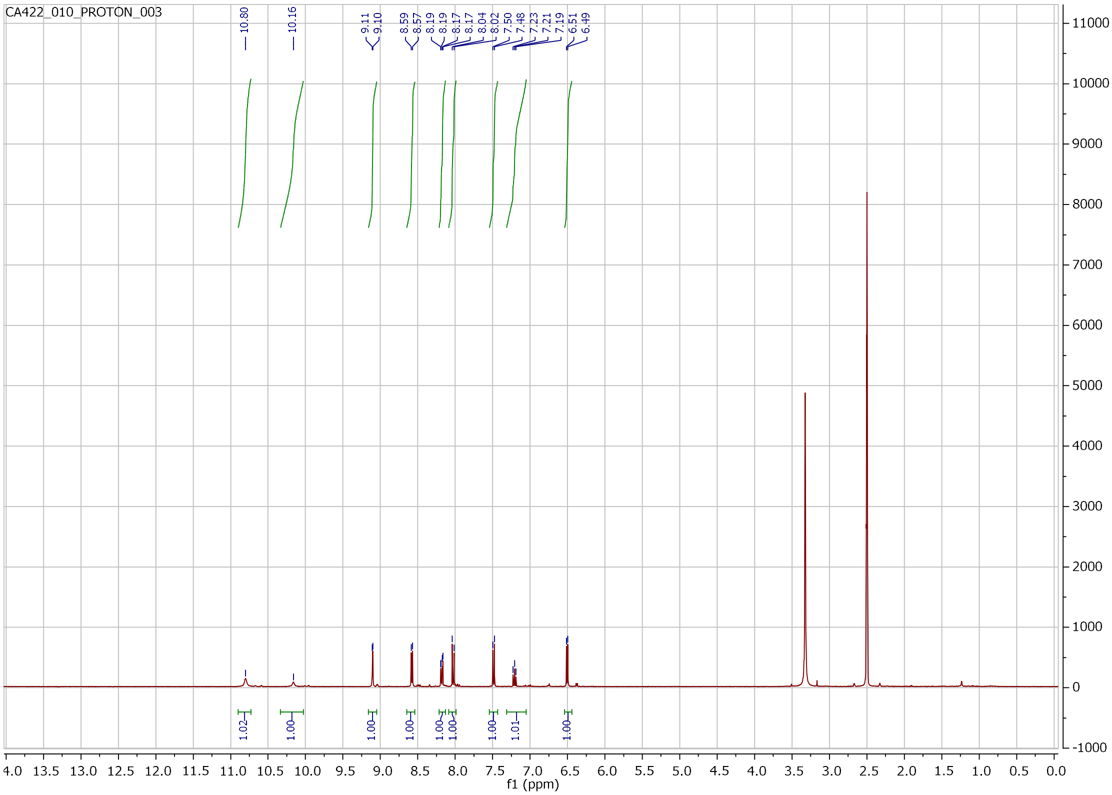
CA422_010_PROTON_003
10.80
10.16
9.11
9.10
8.59
8.57
8.19
8.19
8.17
8.17
8.04
8.02
7.50
7.48
7.23
7.21
7.19
6.51
6.49
1.02
1.00
1.00
1.00
1.00
1.00
1.00
1.01
1.00
f1 (ppm)

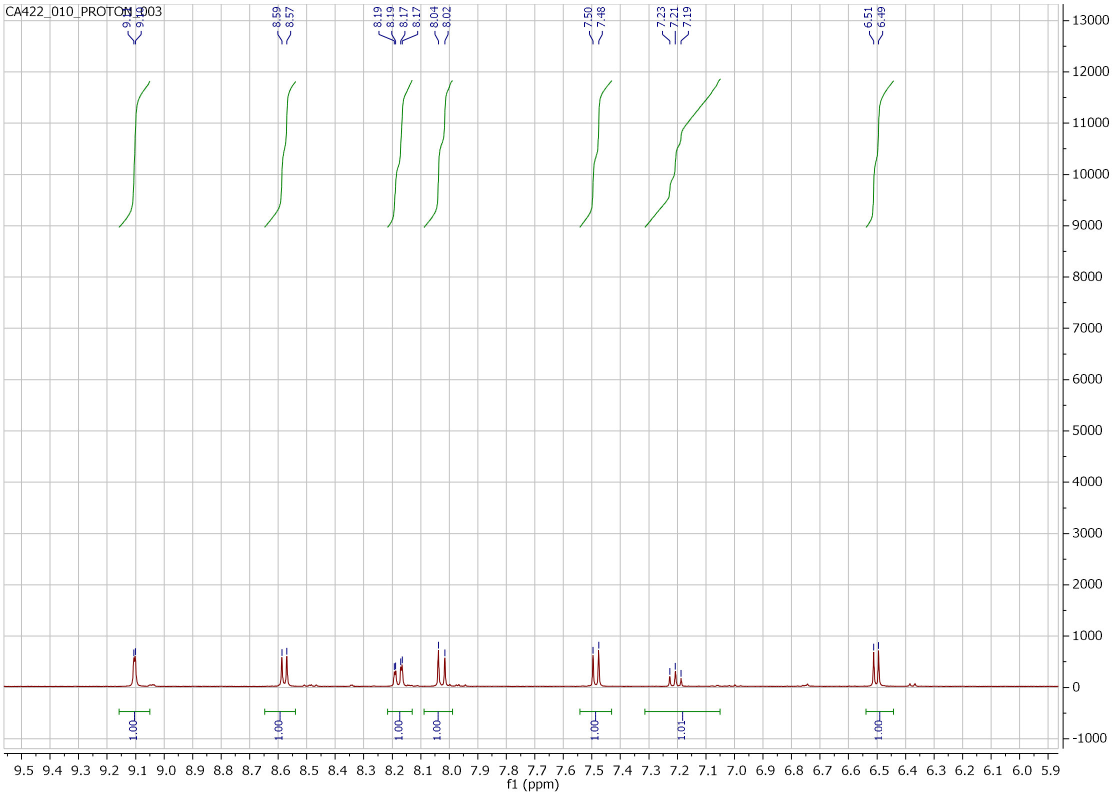
CA422_010_PROTON_003
9.11
9.10
8.59
8.57
8.19
8.19
8.17
8.17
8.04
8.02
7.50
7.48
7.23
7.21
7.19
6.51
6.49
1.00
1.00
1.00
1.00
1.00
1.01
1.00
f1 (ppm)

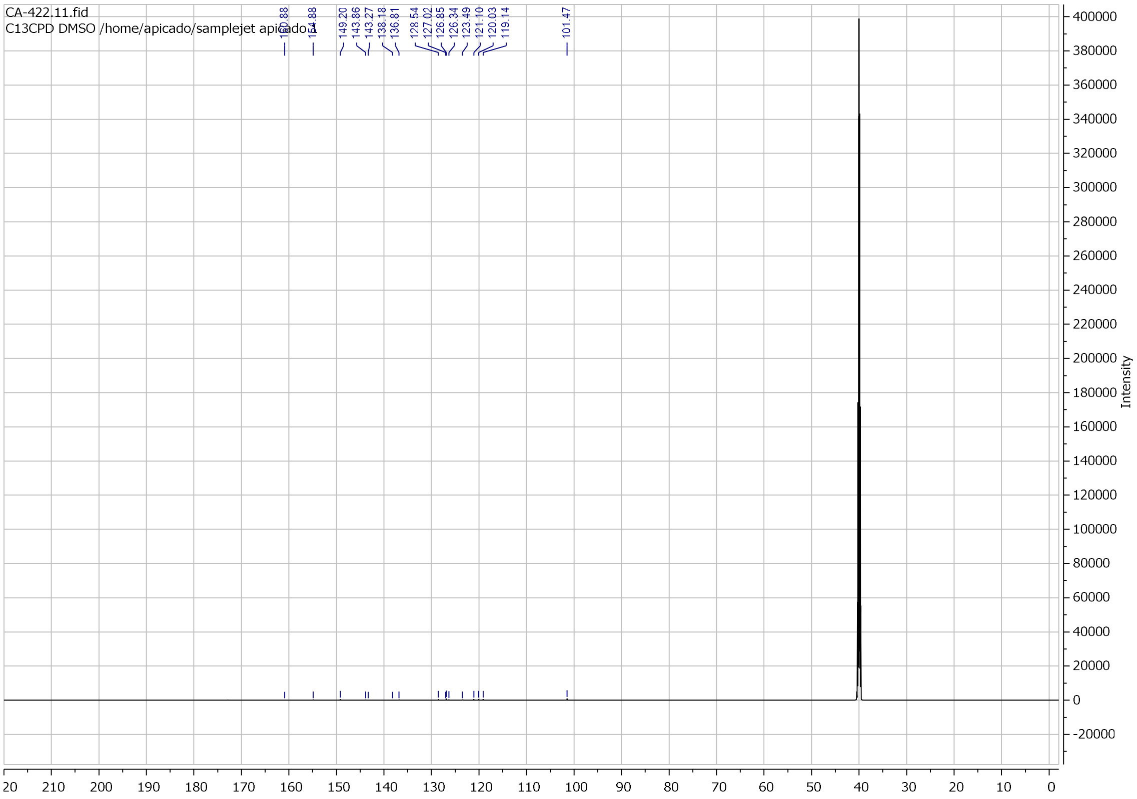
CA-422.11.fid
C13CPD DMSO /home/apicado/samplejet apicado 1
160.88
154.88
149.20
143.86
143.27
138.18
136.81
128.54
127.02
126.85
126.34
123.49
121.10
120.03
119.14
101.47
Intensity

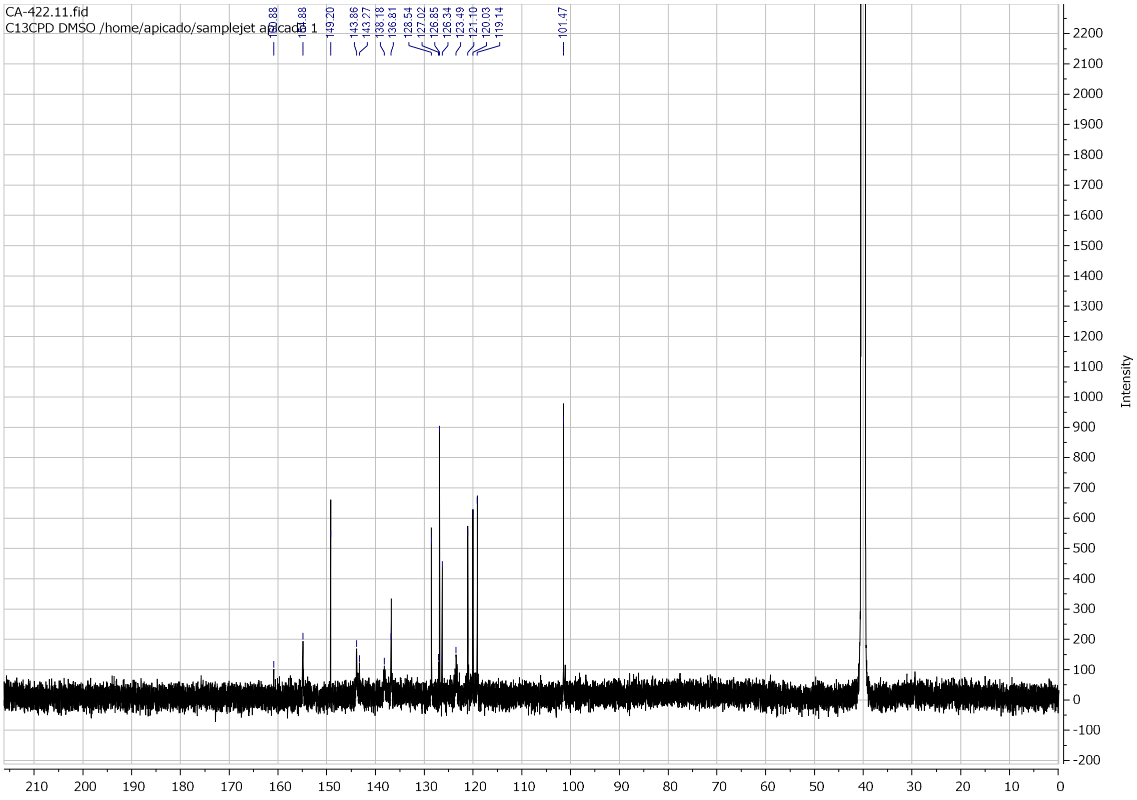
CA-422.11.fid
C13CPD DMSO /home/apicado/samplejet apicado 1
160.88
154.88
149.20
143.86
143.27
138.18
136.81
128.54
127.02
126.85
126.34
123.49
121.10
120.03
119.14
101.47
Intensity

N-(6-bromoquinolin-4-yl)benzo[*d*]thiazol-7-amine (**24**)

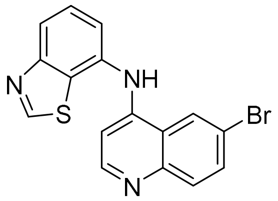

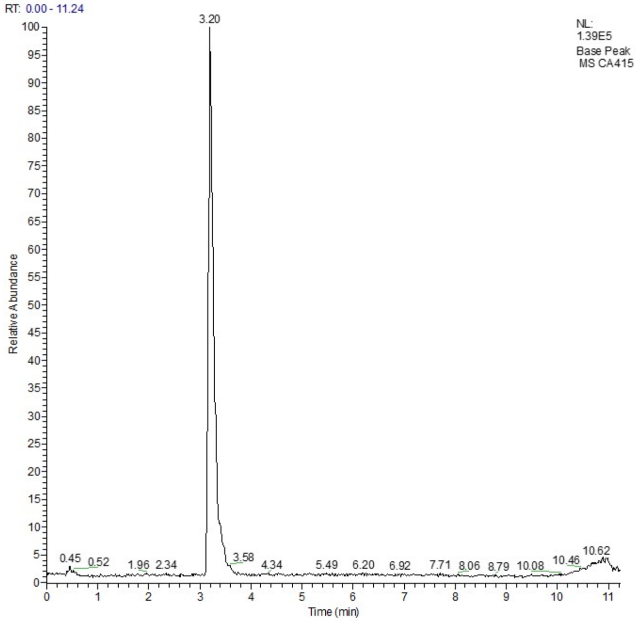

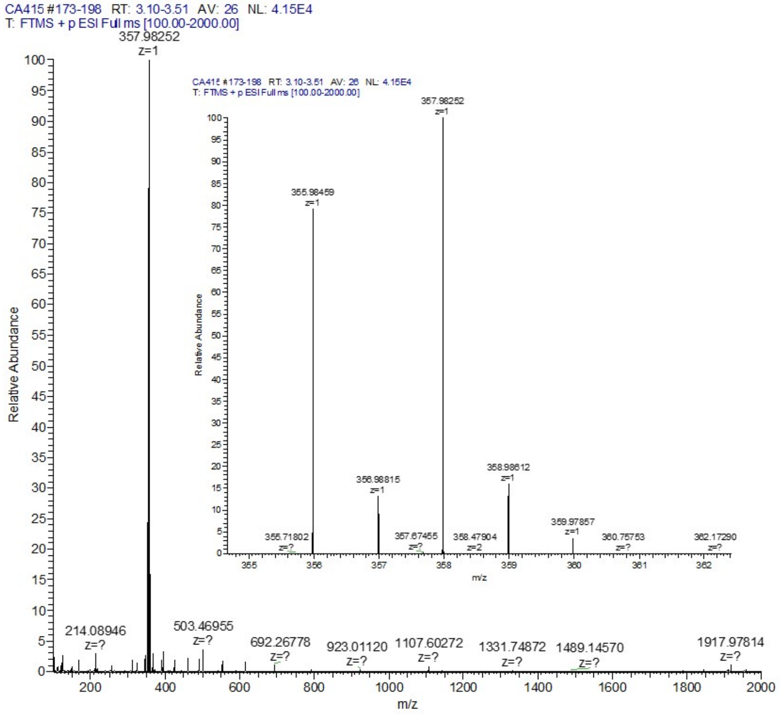

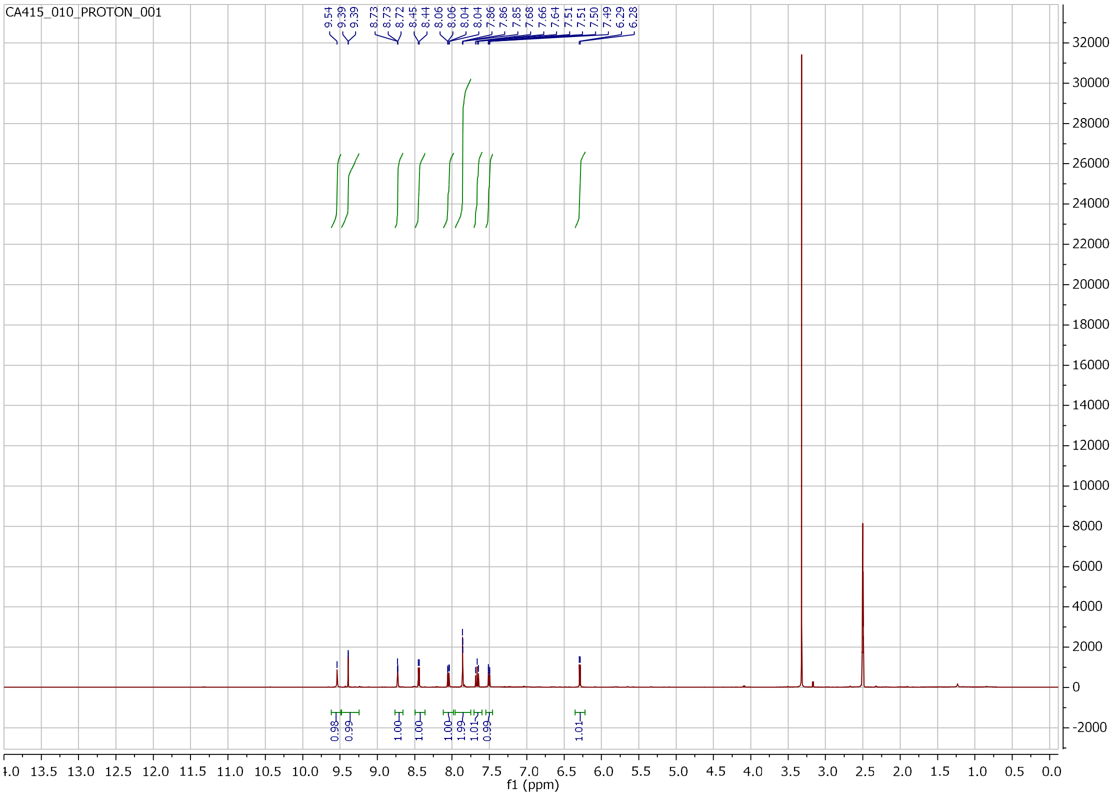

CA415_010_PROTON_001
9.54
9.39
9.39
8.73
8.72
8.45
8.44
8.06
8.04
8.04
7.86
7.85
7.68
7.66
7.64
7.51
7.50
7.49
6.29
6.28
0.98
0.99
1.00
1.00
1.00
1.99
1.01
0.99
1.01
f1 (ppm)

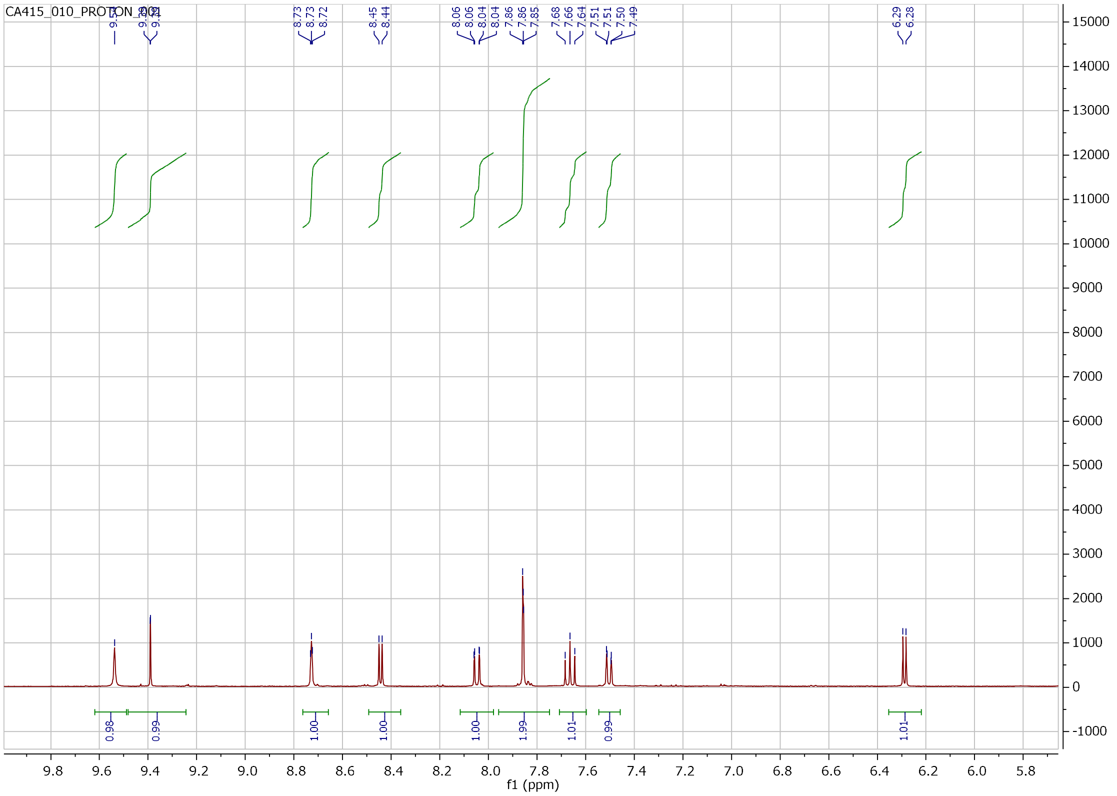

CA415_010_PROTON_001
8.73
8.73
8.72
8.45
8.44
8.06
8.06
8.04
7.86
7.86
7.85
7.68
7.66
7.64
7.51
7.51
7.50
7.49
6.29
6.28
0.98
0.99
1.00
1.00
1.00
1.99
1.01
0.99
1.01
f1 (ppm)

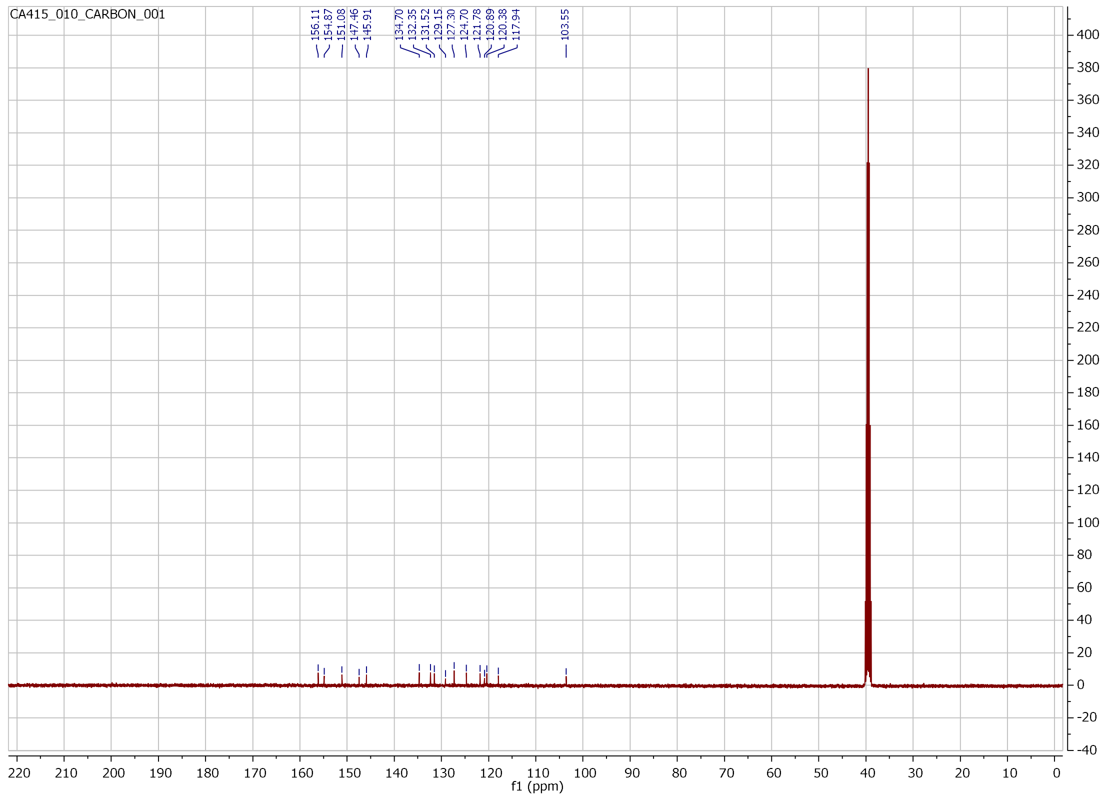
CA415_010_CARBON_001
156.11
154.87
151.08
147.46
145.91
134.70
132.35
131.52
129.15
127.30
124.70
121.78
120.89
120.38
117.94
103.55
f1 (ppm)

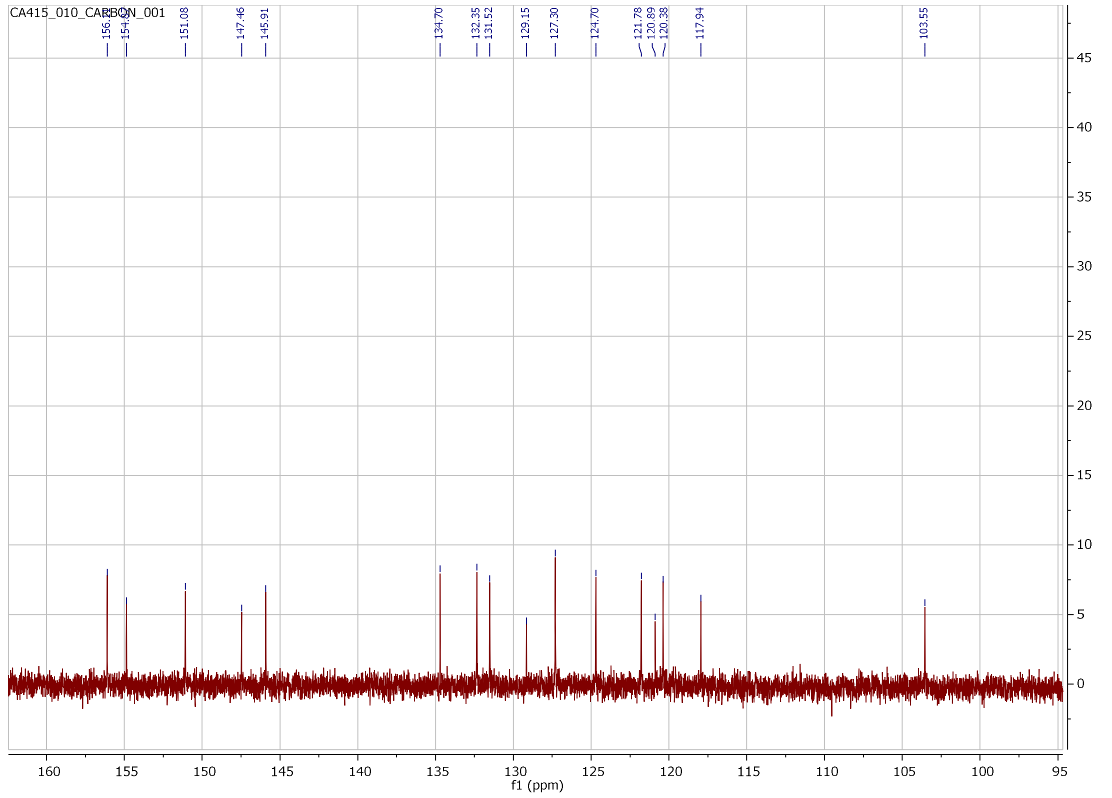
CA415_010_CARBON_001
151.08
147.46
145.91
134.70
132.35
131.52
129.15
127.30
124.70
121.78
120.89
120.38
117.94
103.55
f1 (ppm)

N-(6-bromoquinolin-4-yl)benzo[*d*]thiazol-4-amine (**25**)

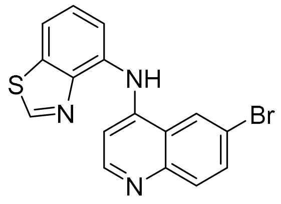

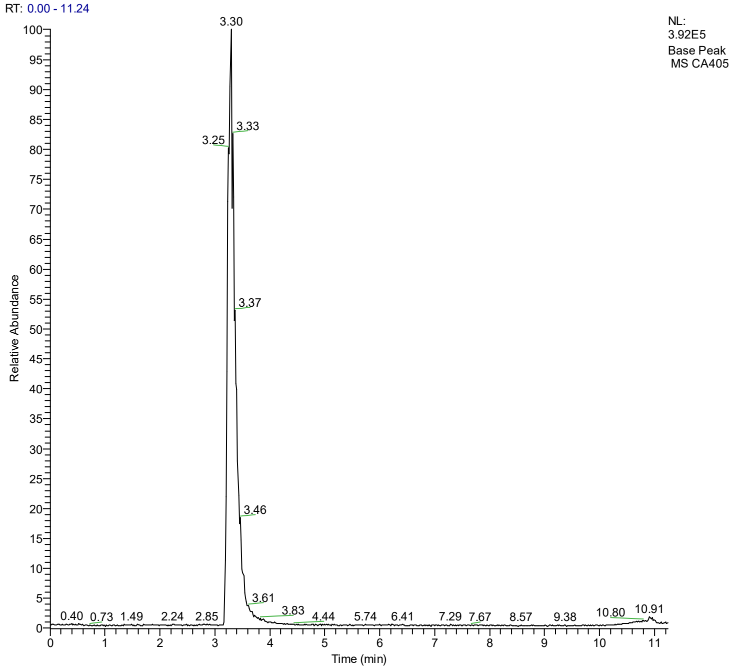

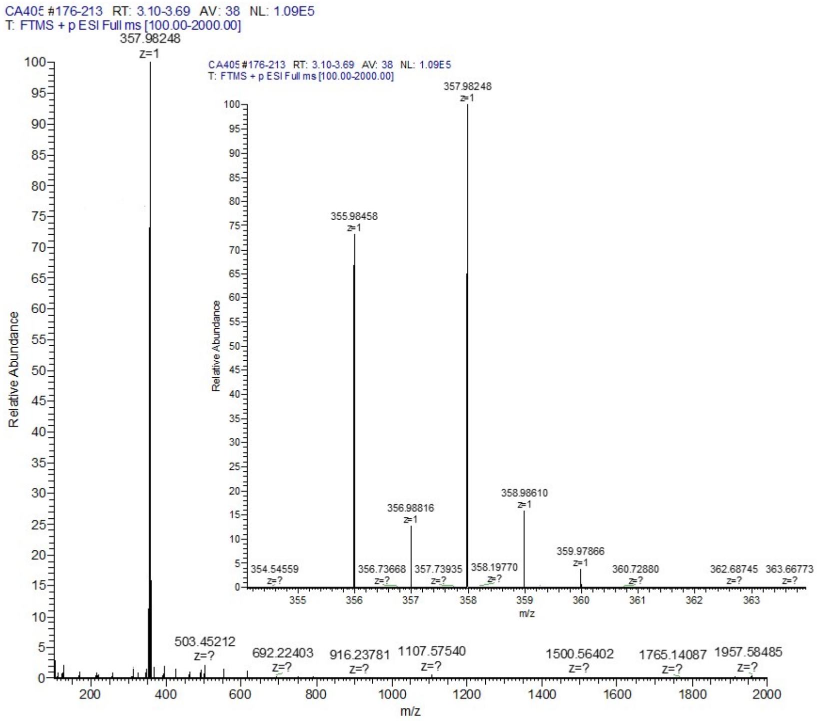

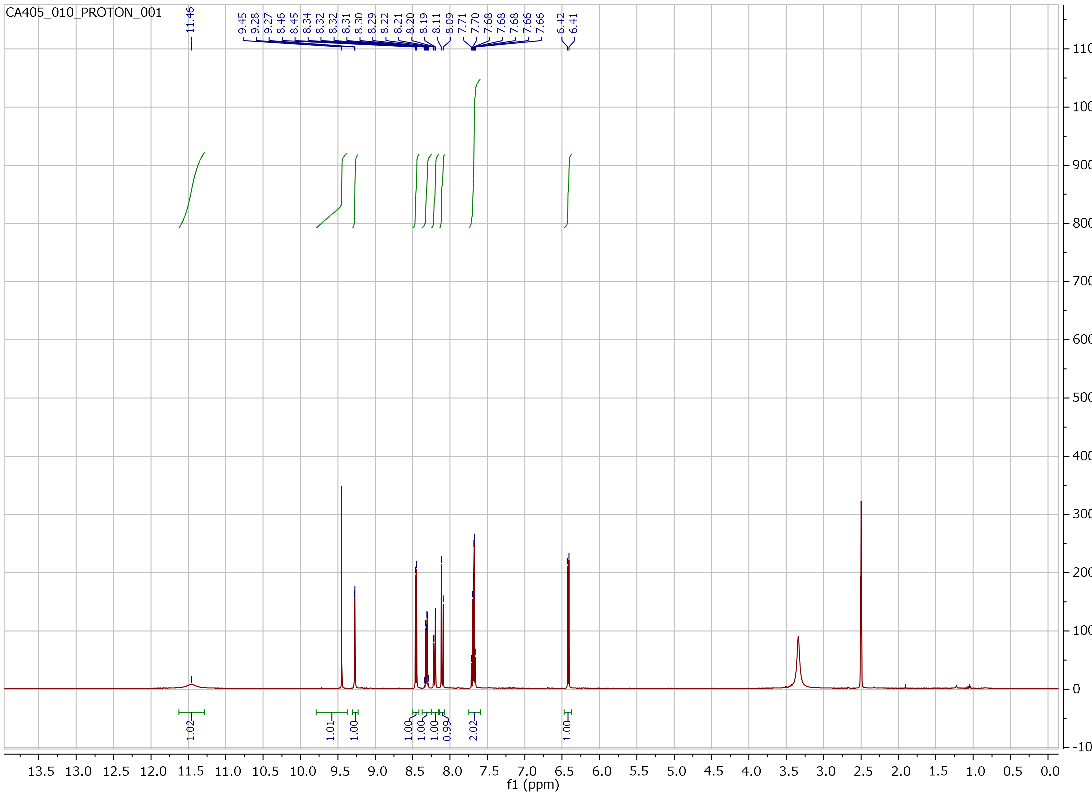
CA405_010_PROTON_001
f1 (ppm)

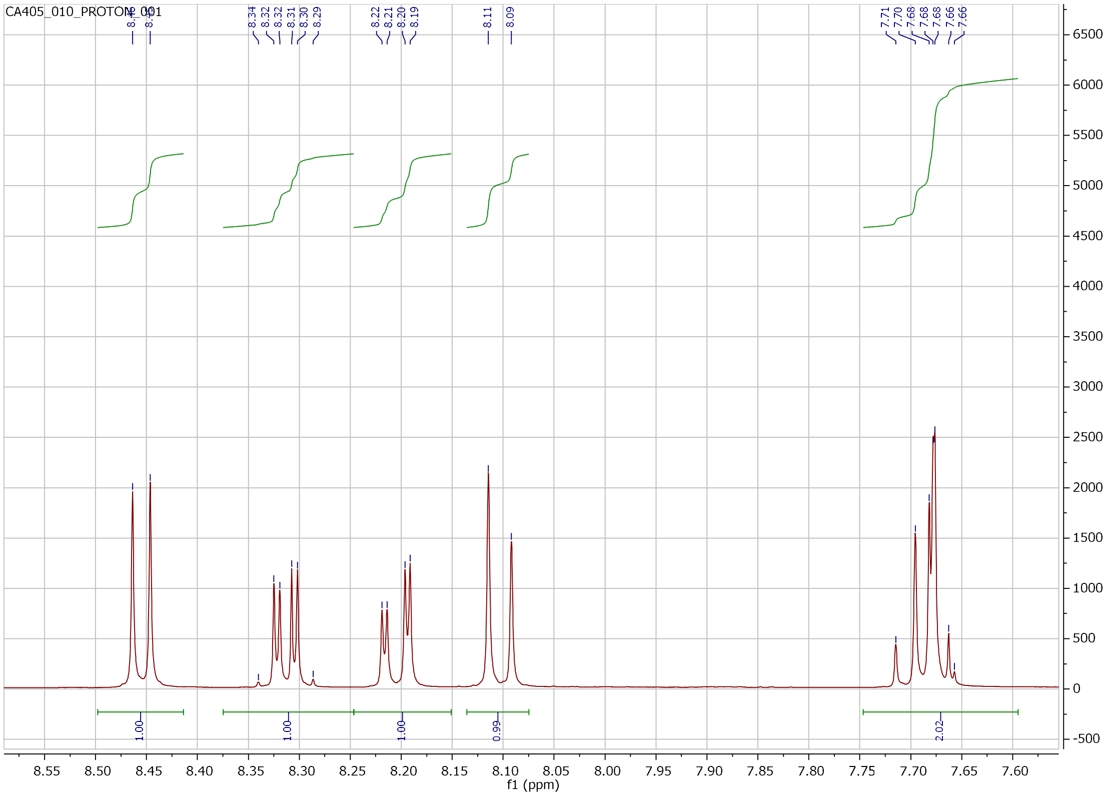
CA405_010_PROTON_001
f1 (ppm)

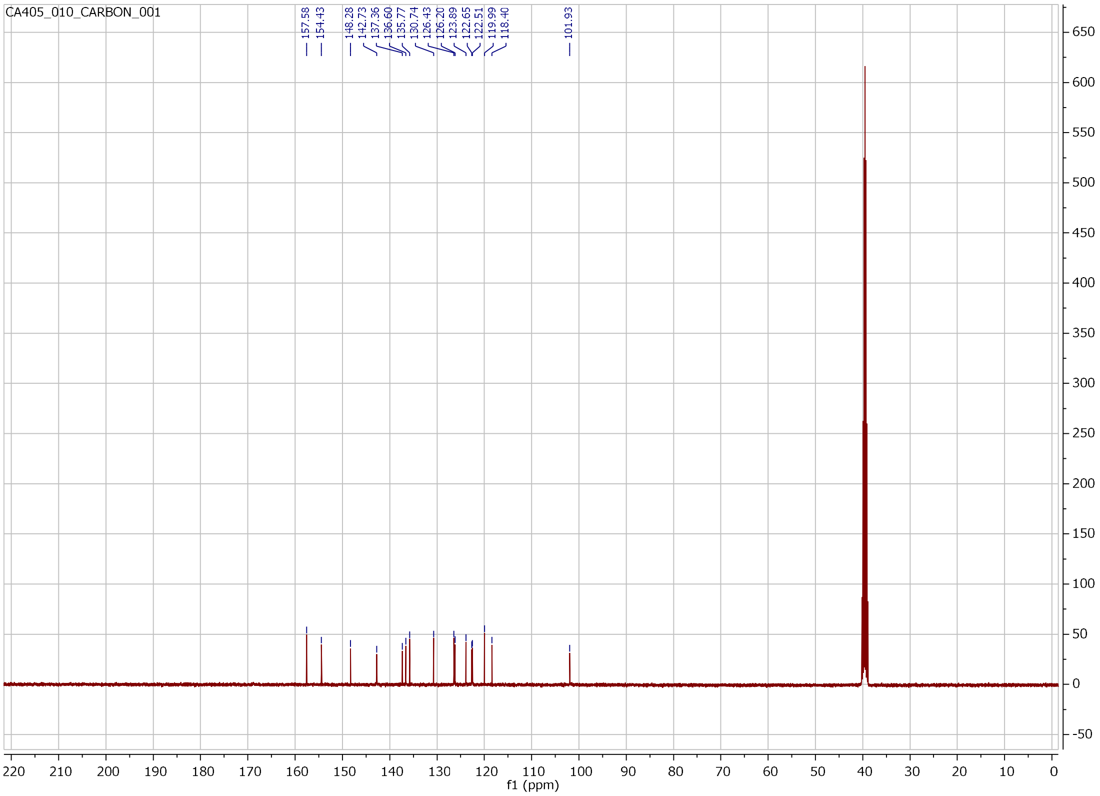

CA405_010_CARBON_001
157.58
154.43
148.28
142.73
137.36
136.60
135.77
130.74
126.43
126.20
123.89
122.65
122.51
119.99
118.40
101.93
f1 (ppm)

N-(benzo[*b*]thiophen-4-yl)-6-bromoquinolin-4-amine (**26**)

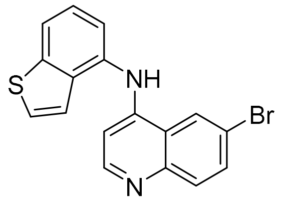

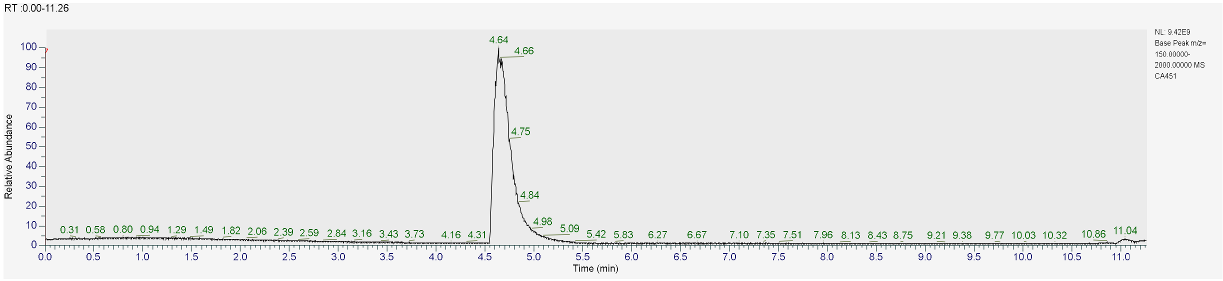

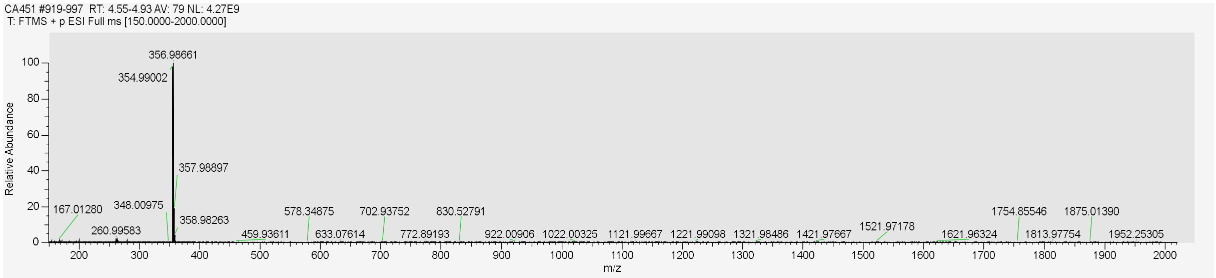

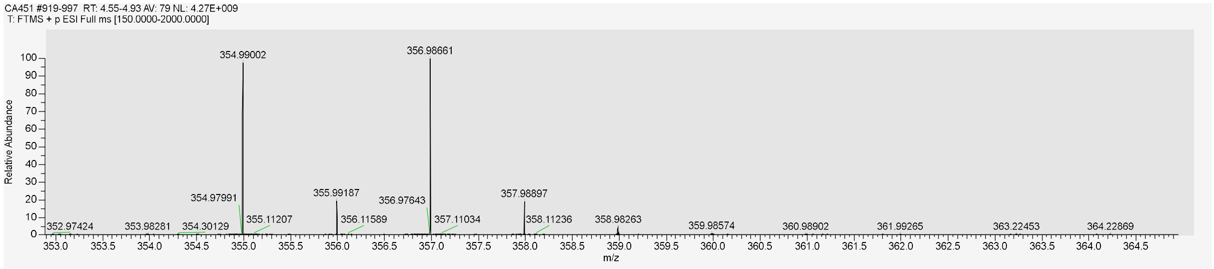

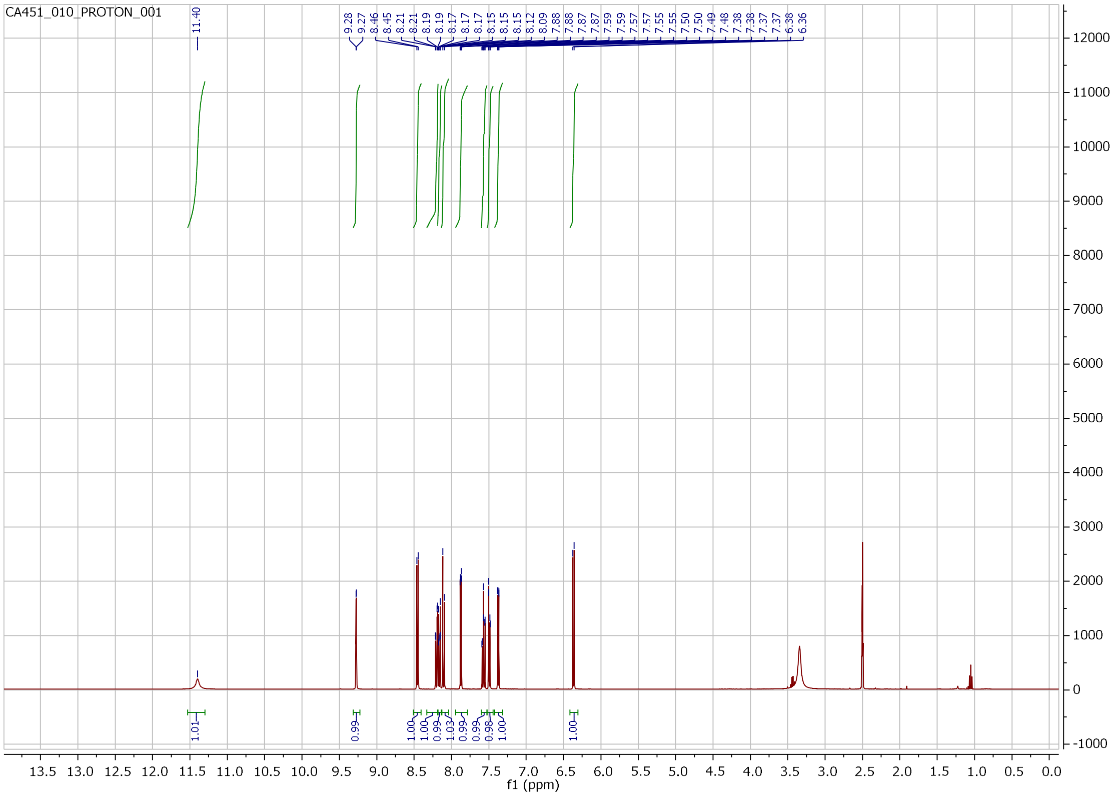
CA451_010_PROTON_001
f1 (ppm)

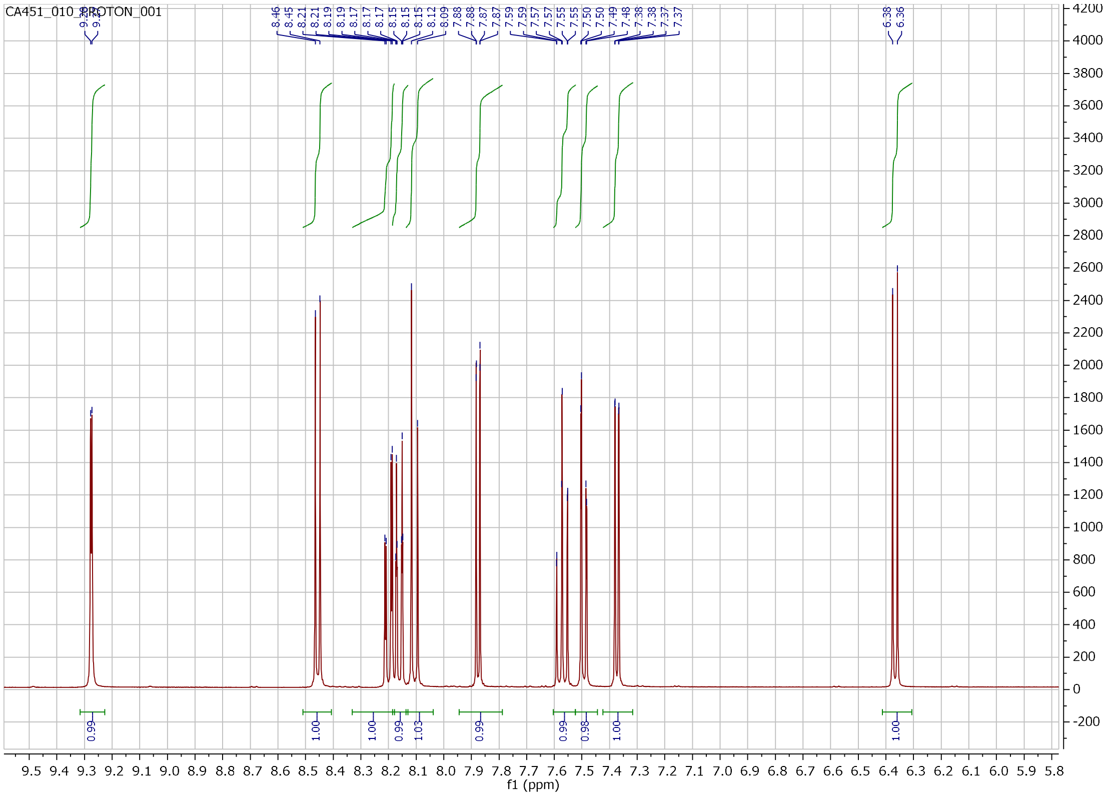
CA451_010_PROTON_001
f1 (ppm)

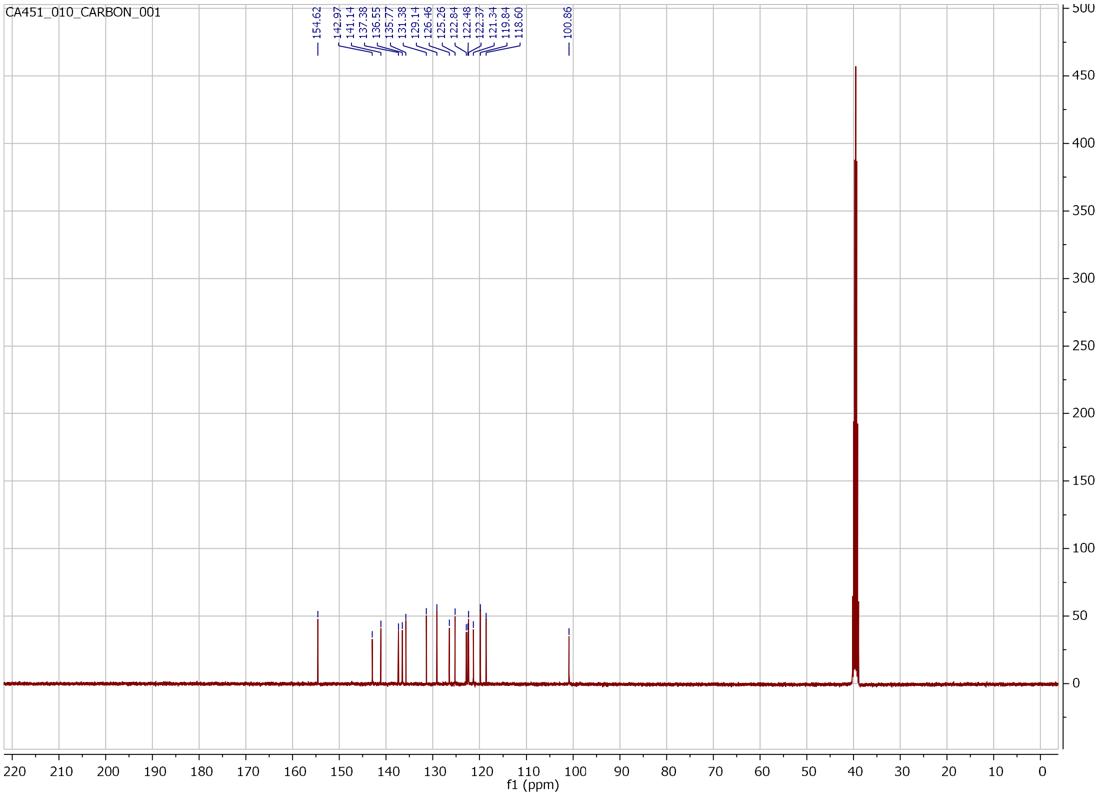

CA451_010_CARBON_001
154.62
142.97
141.14
137.38
136.55
135.77
131.38
129.14
126.46
125.26
122.84
122.48
122.37
121.34
119.84
118.60
100.86
f1 (ppm)

N-(1*H*-benzo[*d*]imidazol-7-yl)-6-bromoquinolin-4-amine (**27**)

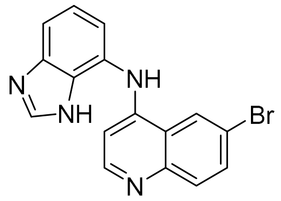

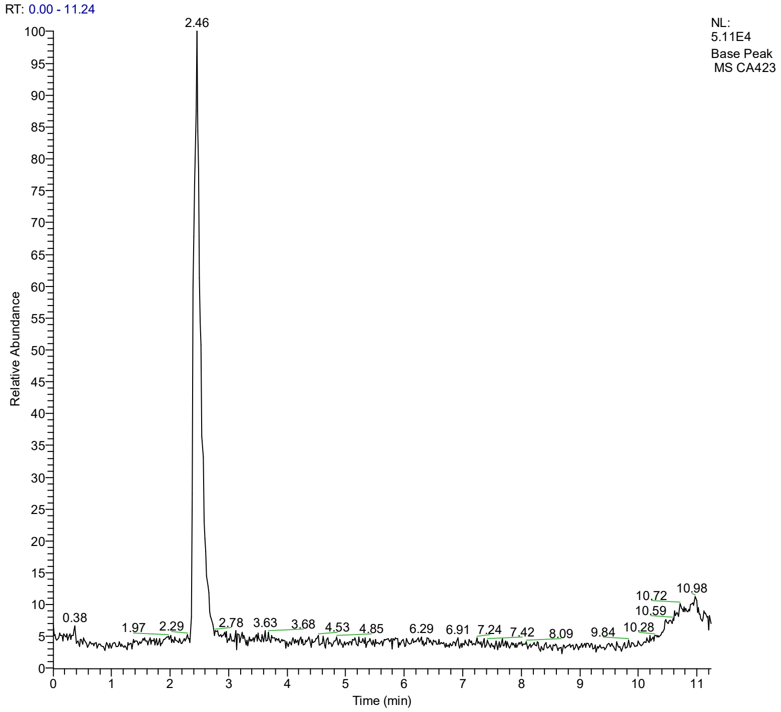

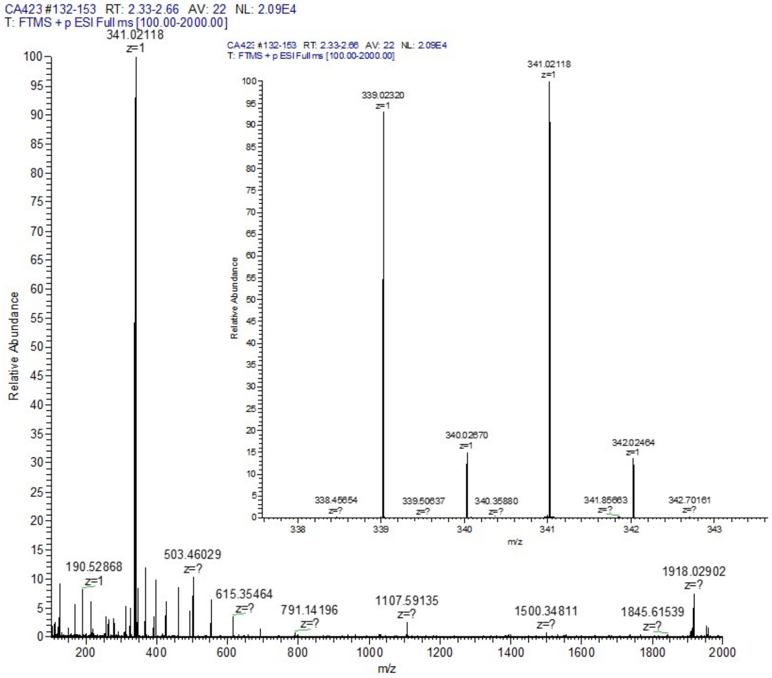

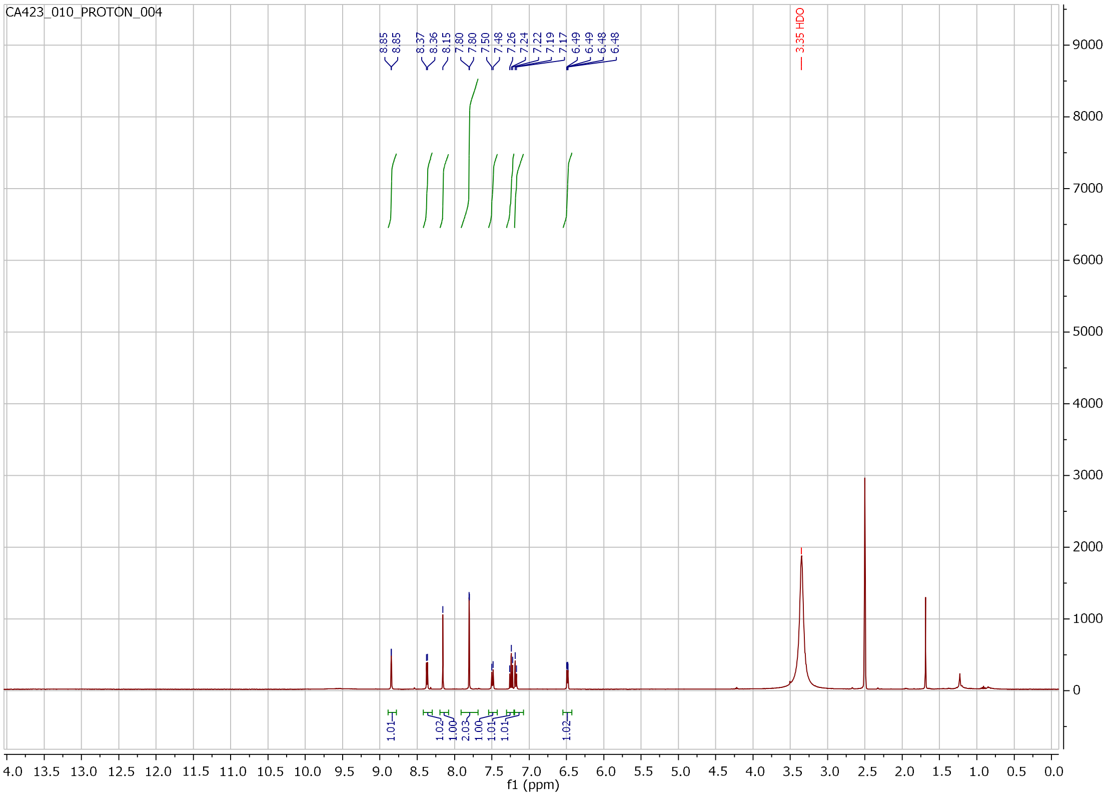
CA423_010_PROTON_004
8.85
8.85
8.37
8.36
8.15
7.80
7.80
7.50
7.48
7.26
7.24
7.22
7.19
7.17
6.49
6.49
6.48
6.48
3.35 HDO
1.01
1.02
1.00
2.03
1.00
1.01
1.01
1.02
f1 (ppm)

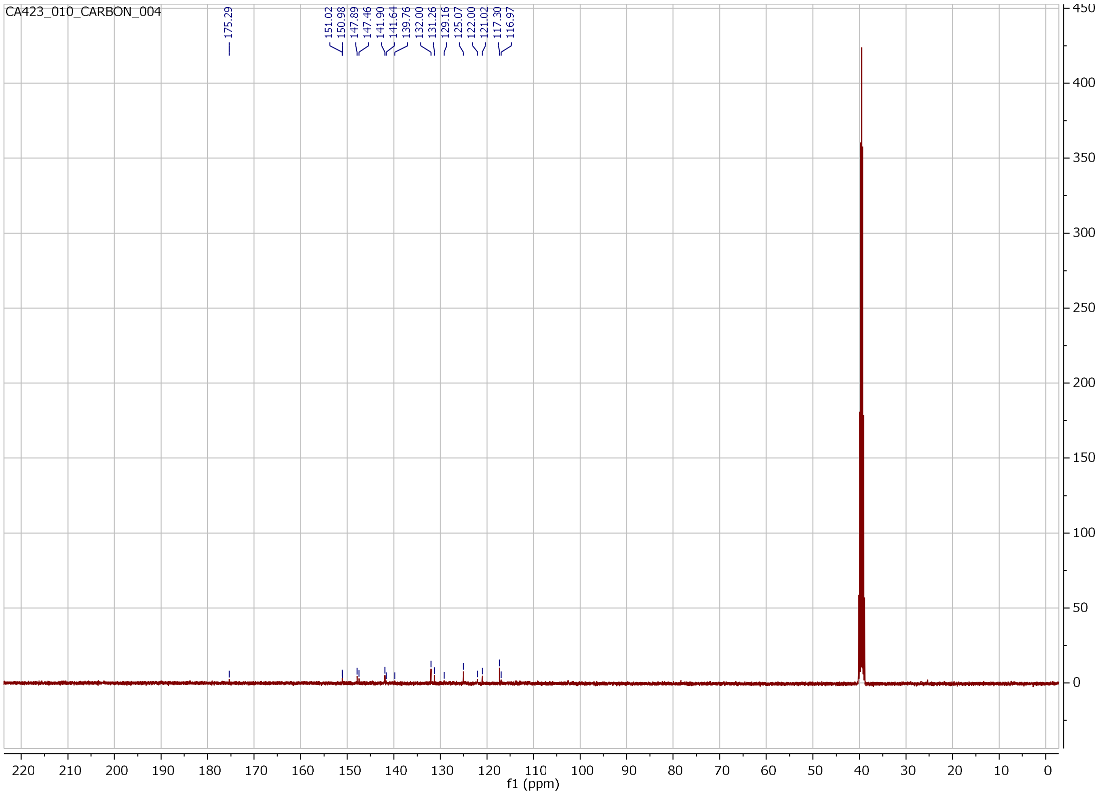
CA423_010_CARBON_004
175.29
151.02
150.98
147.89
147.46
141.90
141.64
139.76
132.00
131.26
129.16
125.07
122.00
121.02
117.30
116.97
f1 (ppm)

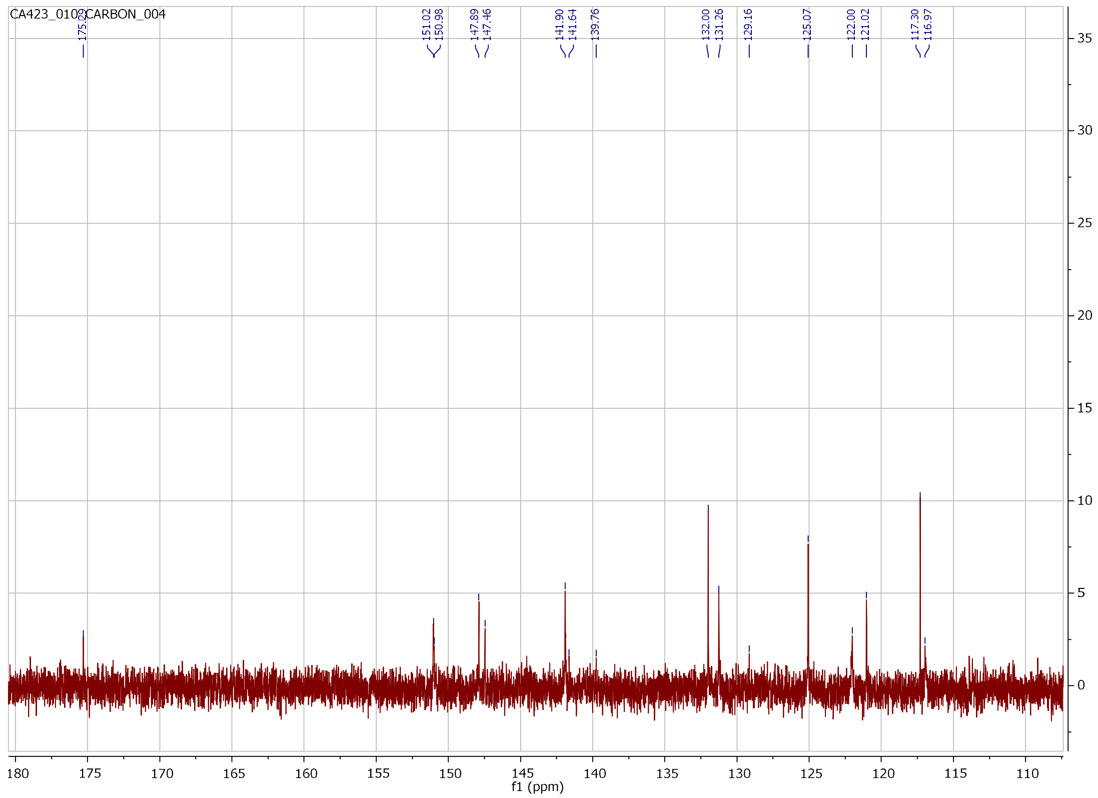
CA423_0102CARBON_004
175.23
151.02
150.98
147.89
147.46
141.90
141.64
139.76
132.00
131.26
129.16
125.07
122.00
121.02
117.30
116.97
f1 (ppm)

N-(6-bromoquinolin-4-yl)benzo[*c*][1,2,5]oxadiazol-4-amine (**28**)

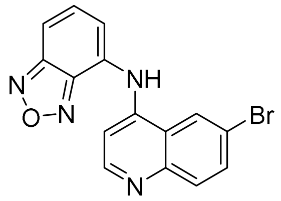

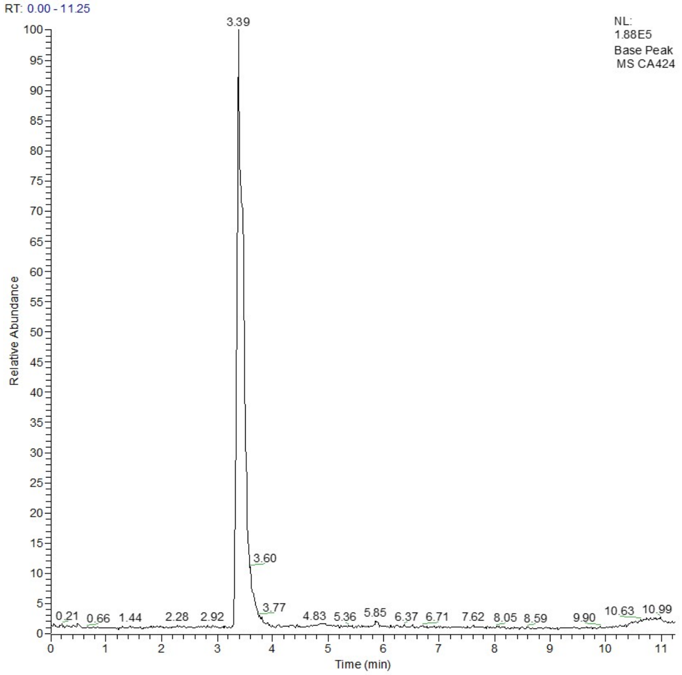

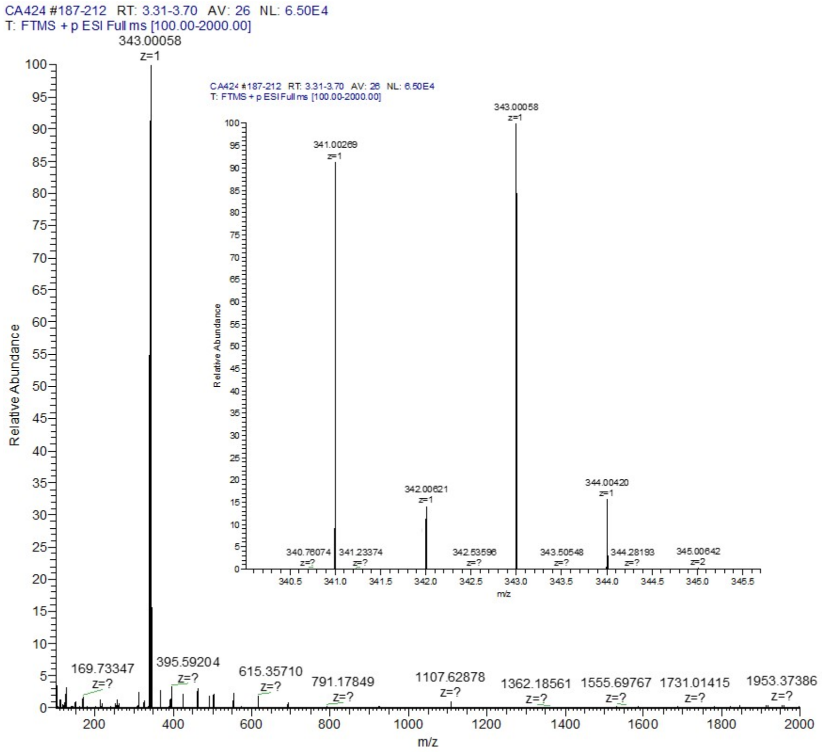

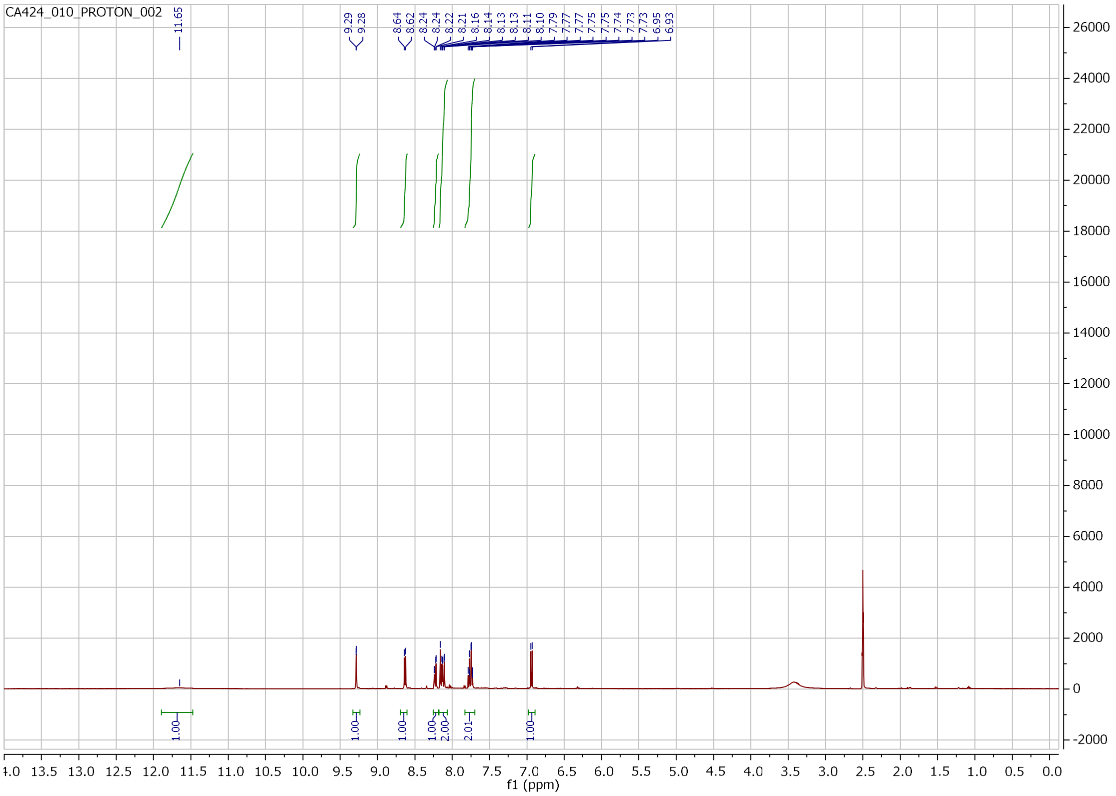

CA424_010_PROTON_002
11.65
9.29
9.28
8.64
8.62
8.24
8.24
8.22
8.21
8.16
8.14
8.13
8.13
8.11
8.10
7.79
7.77
7.77
7.75
7.75
7.74
7.73
7.73
6.95
6.93
1.00
1.00
1.00
1.00
2.00
2.01
1.00
f1 (ppm)

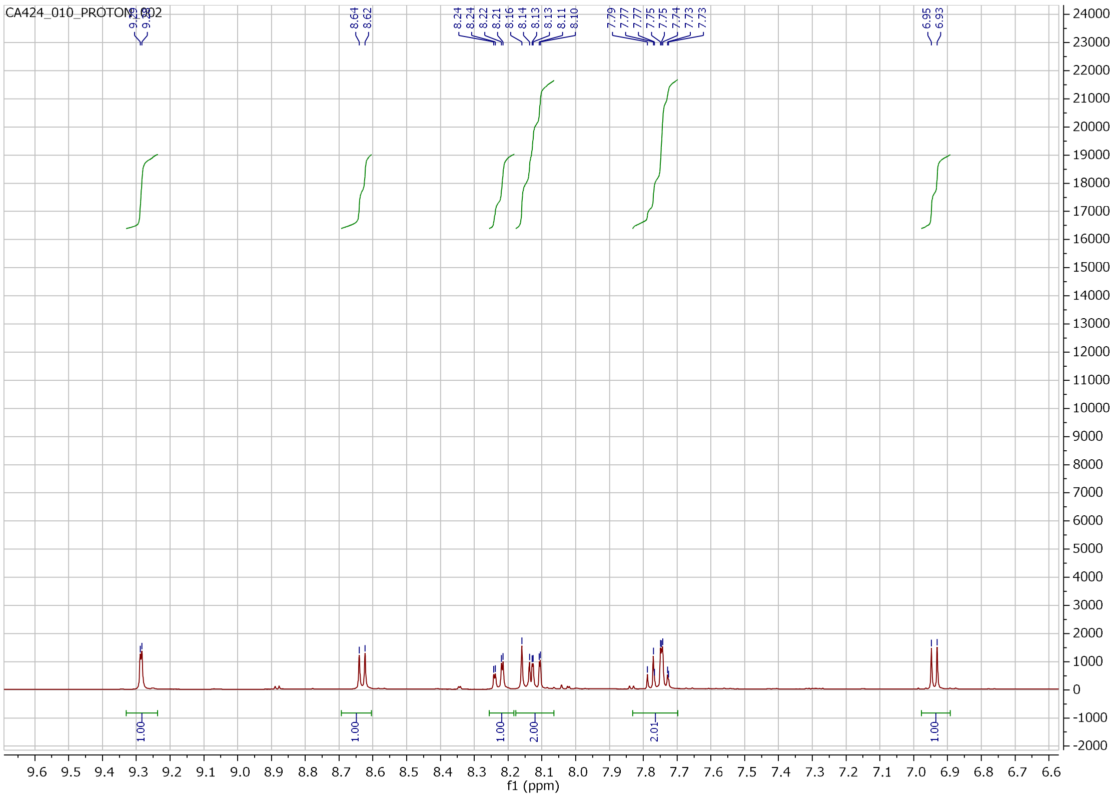

CA424_010_PROTON_002
8.64
8.62
8.24
8.24
8.22
8.21
8.16
8.14
8.13
8.13
8.11
8.10
7.79
7.77
7.77
7.75
7.75
7.74
7.73
7.73
6.95
6.93
1.00
1.00
1.00
2.00
2.01
1.00
f1 (ppm)

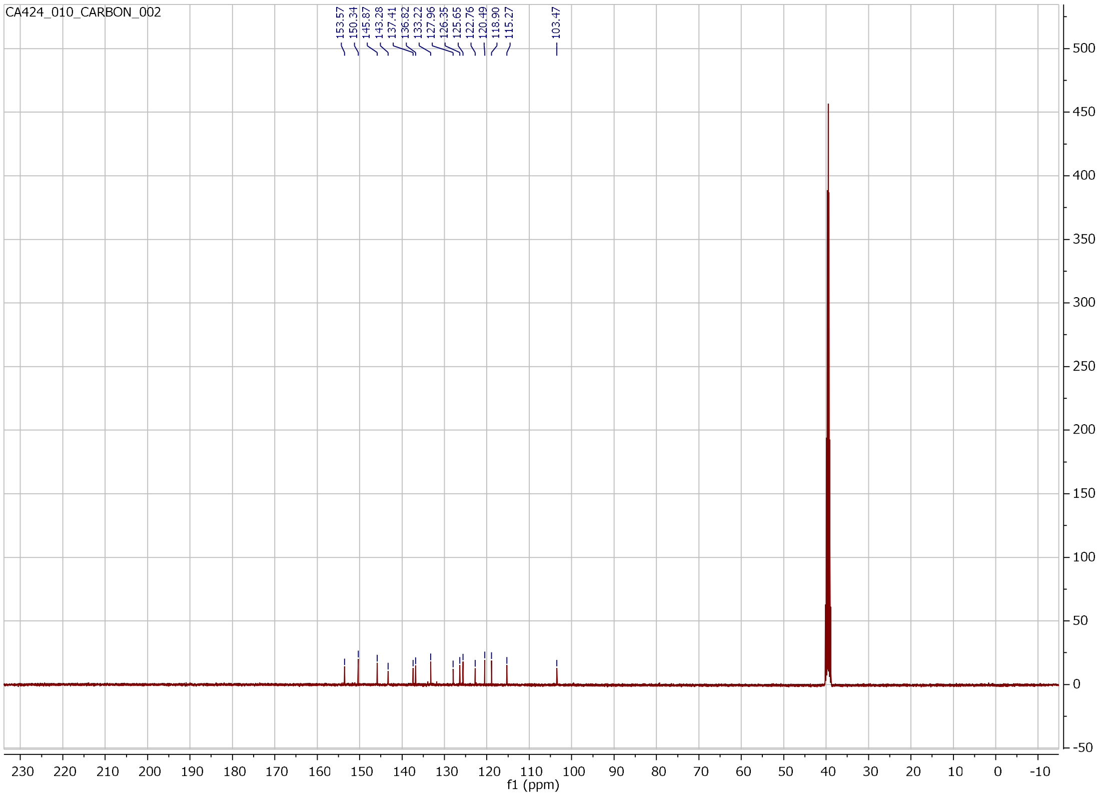
CA424_010_CARBON_002
153.57
150.34
145.87
143.28
137.41
136.82
133.22
127.96
126.35
125.65
122.76
120.49
118.90
115.27
103.47
f1 (ppm)

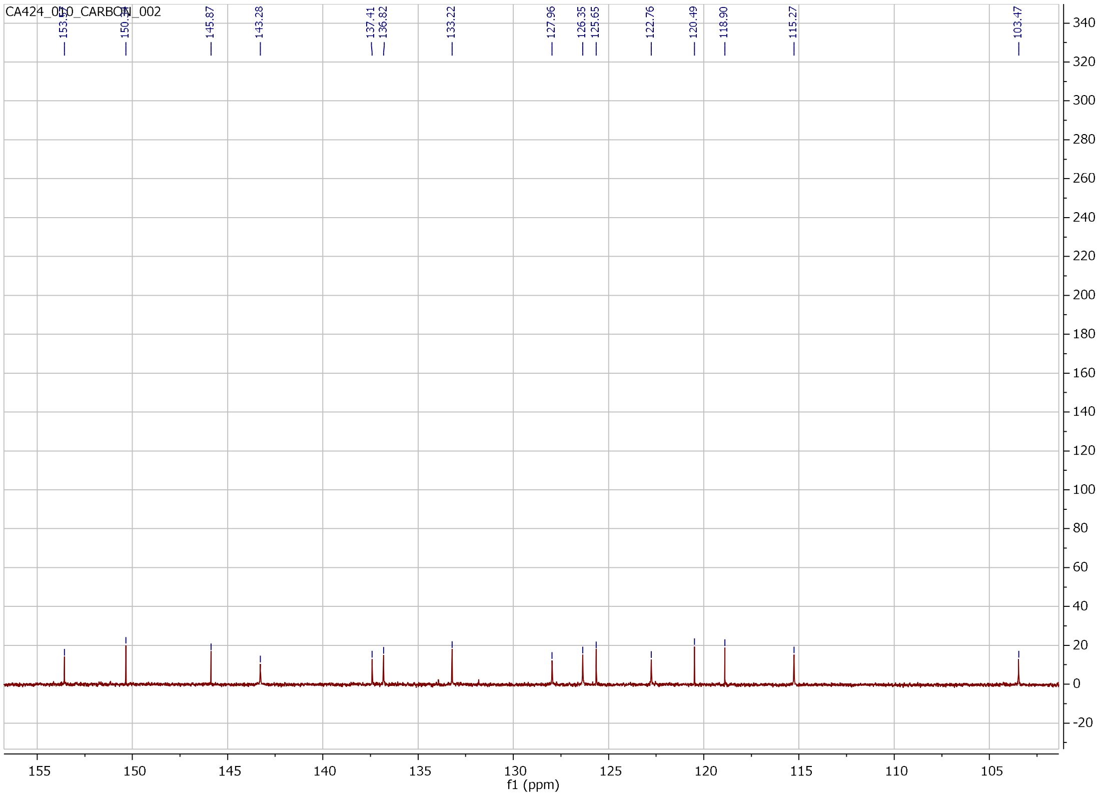
CA424_010_CARBON_002
145.87
143.28
137.41
136.82
133.22
127.96
126.35
125.65
122.76
120.49
118.90
115.27
103.47
f1 (ppm)

N-(6-bromoquinolin-4-yl)benzo[*c*][1,2,5]thiadiazol-4-amine (**29**)

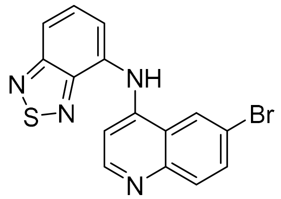

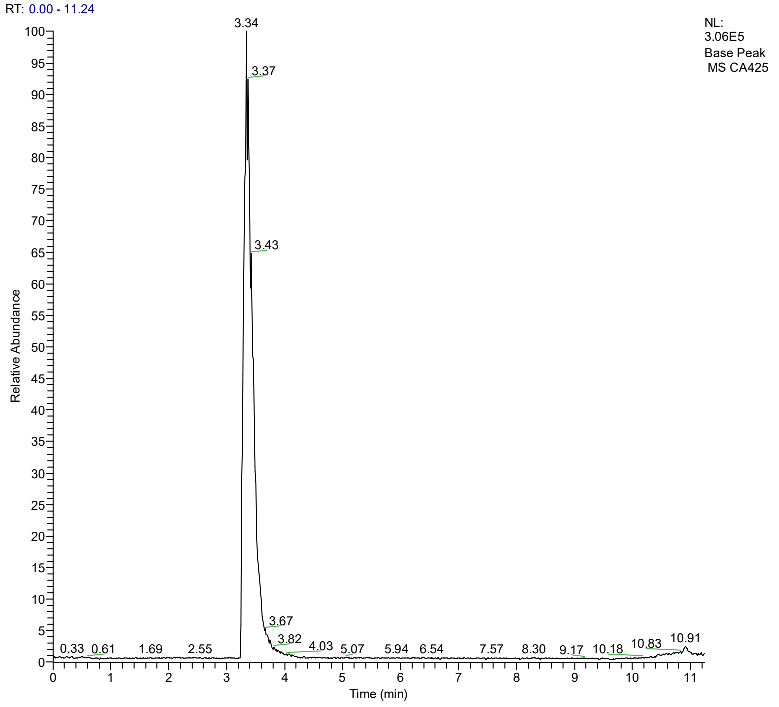

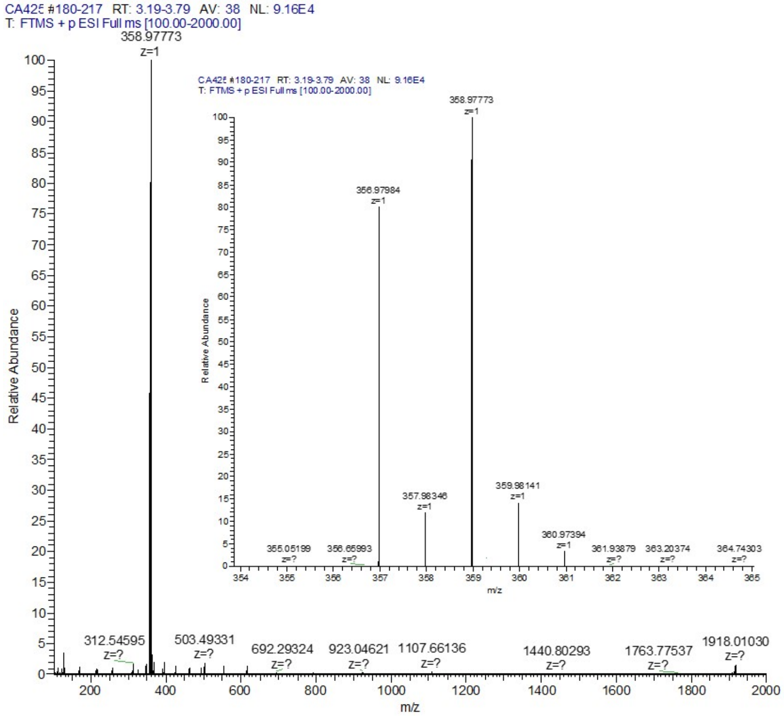

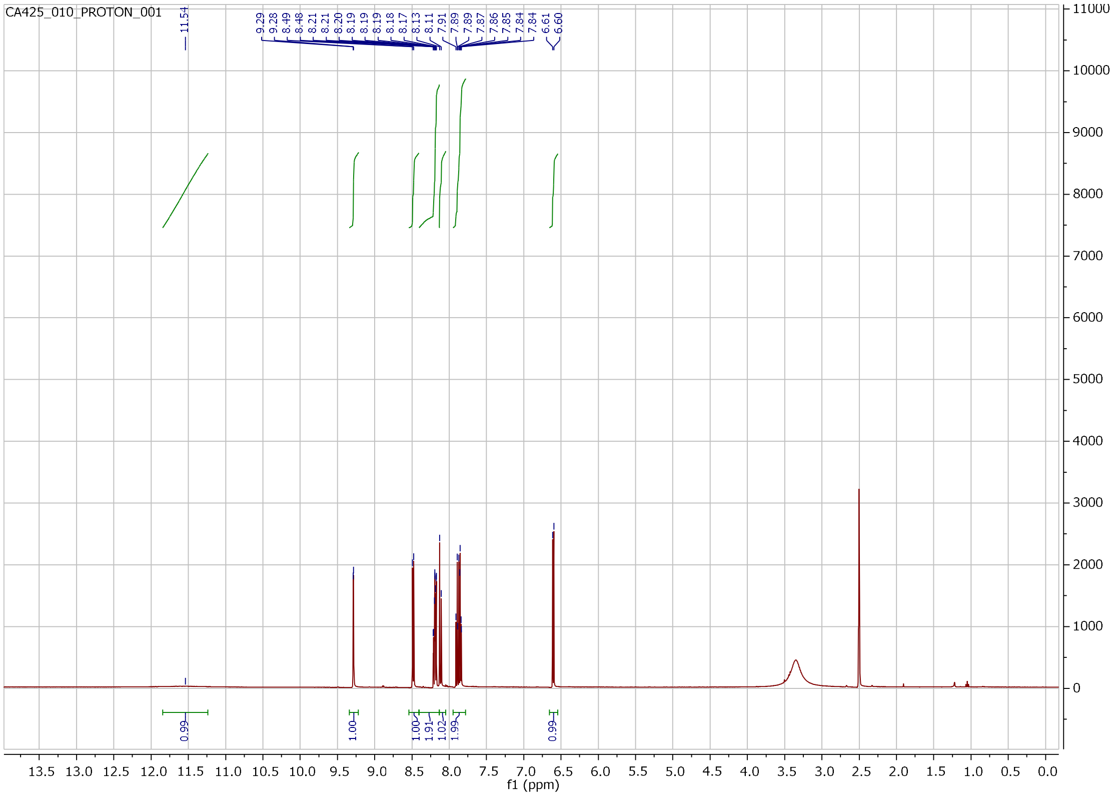
CA425_010_PROTON_001
f1 (ppm)

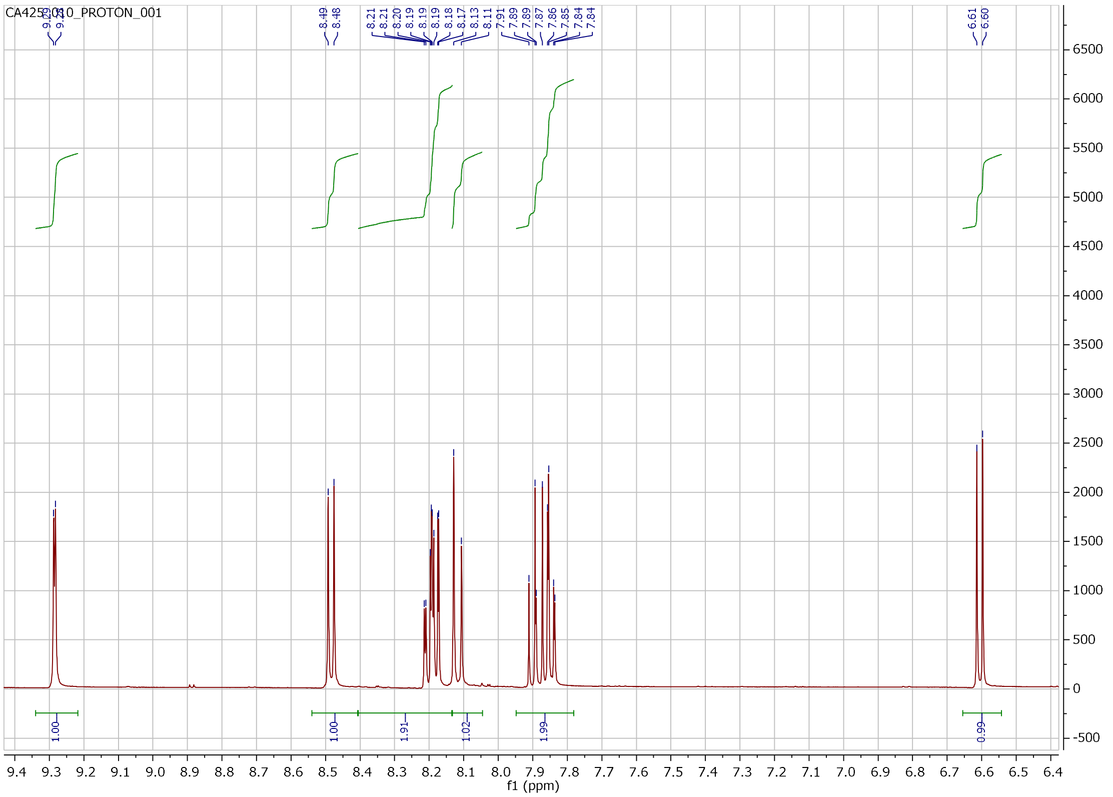
CA425_010_PROTON_001
f1 (ppm)

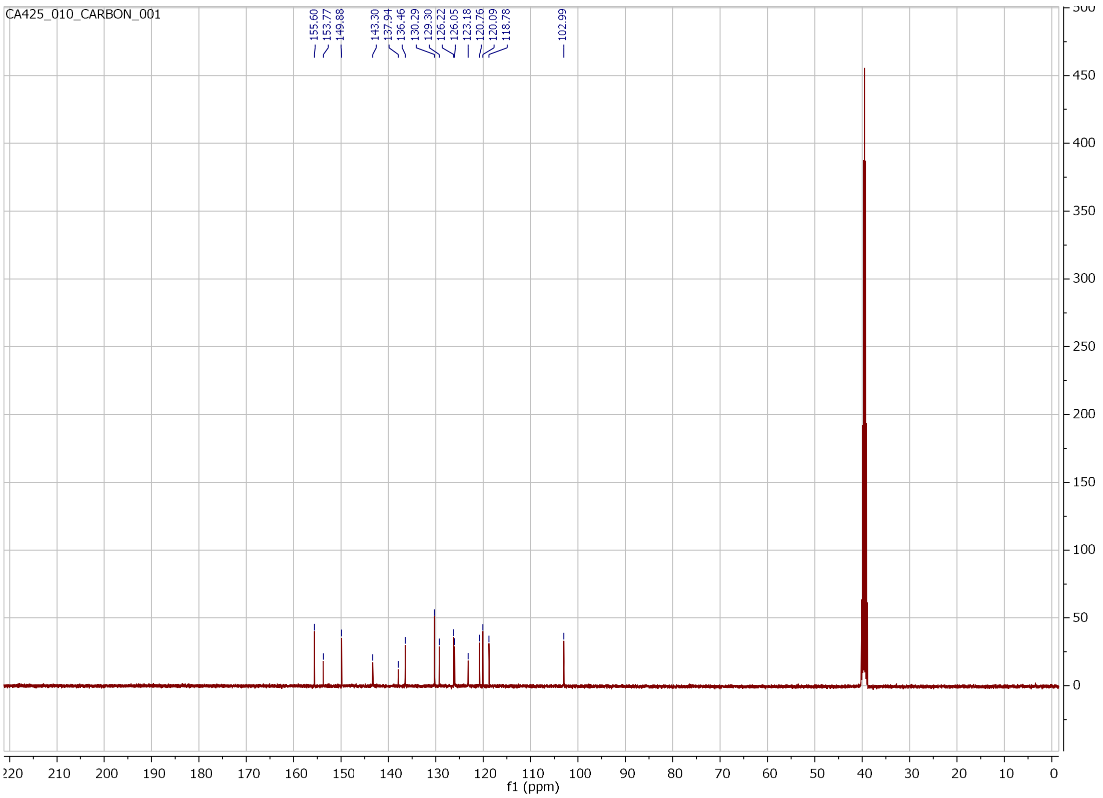

CA425_010_CARBON_001
155.60
153.77
149.88
143.30
137.94
136.46
130.29
129.30
126.22
126.05
123.18
120.76
120.09
118.78
102.99
f1 (ppm)

6-bromo-*N*-(1-methyl-1H-benzo[*d*][1,2,3]triazol-4-yl)quinolin-4-amine (**30**)

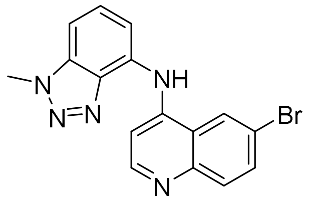

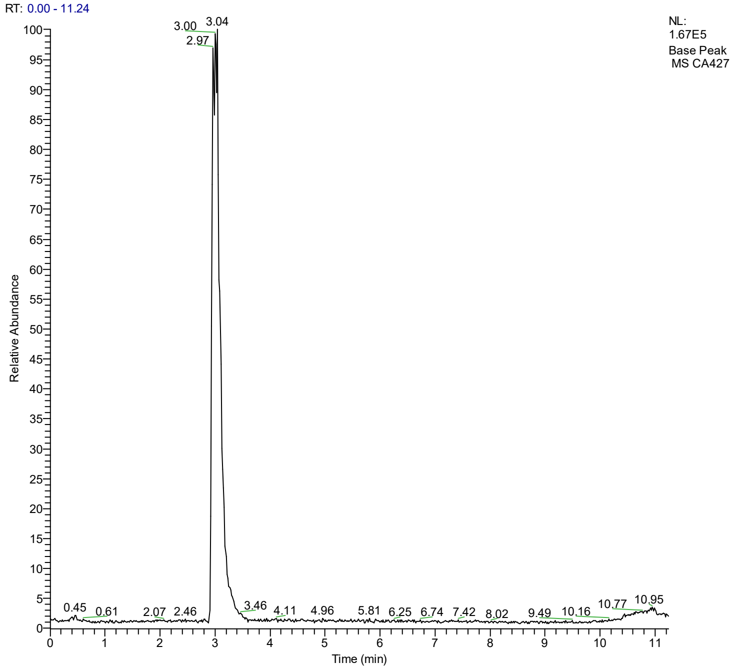

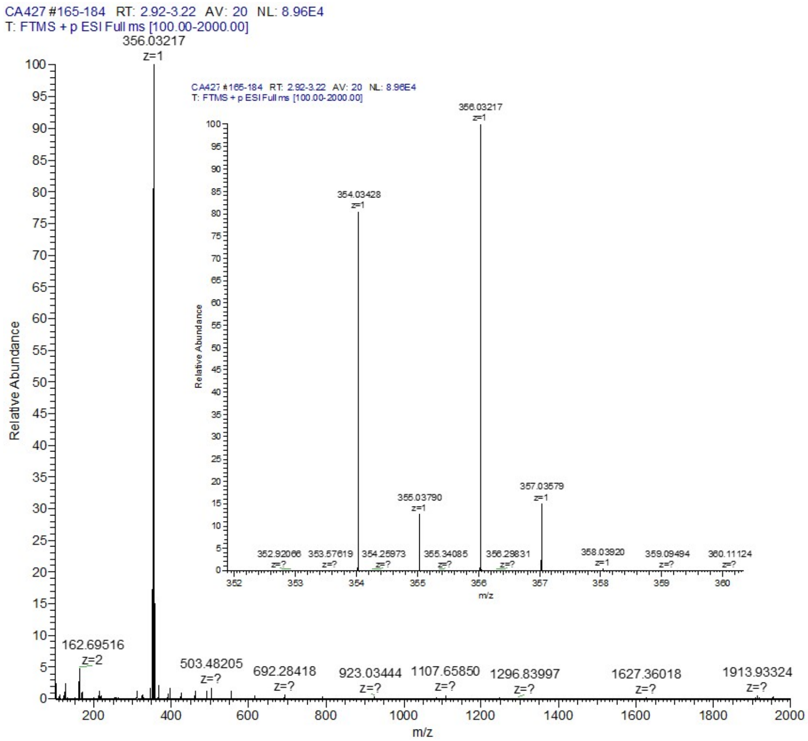

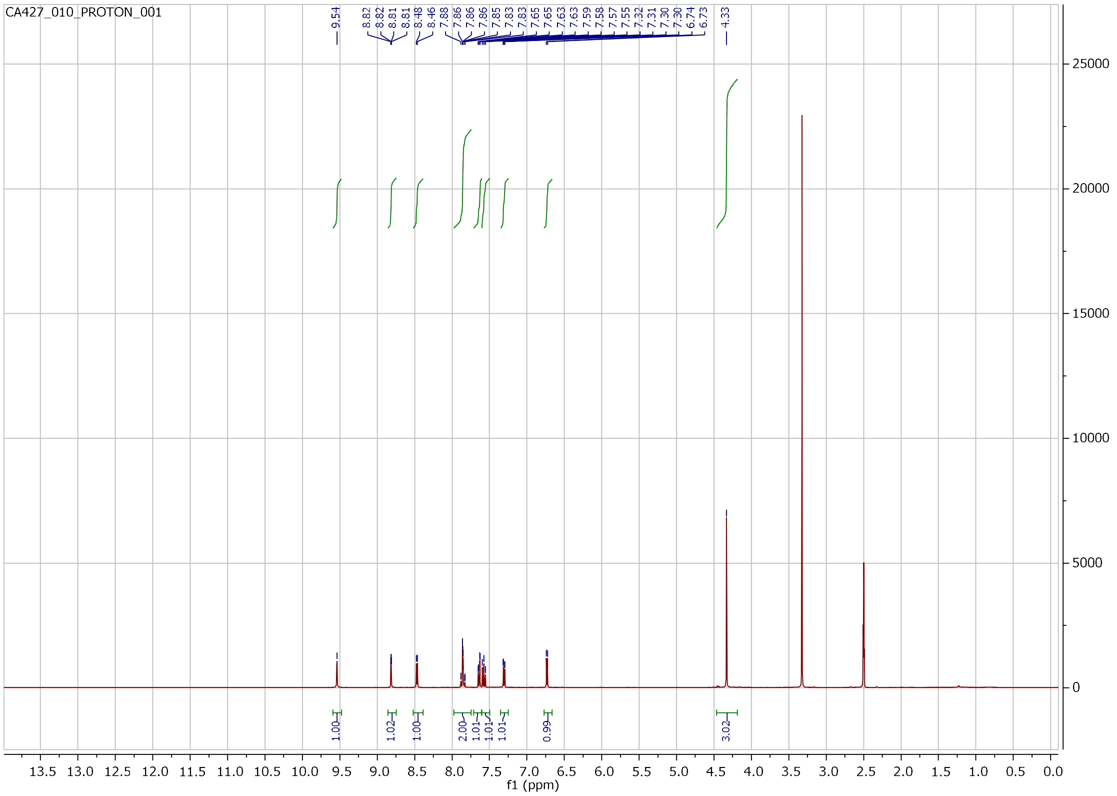
CA427_010_PROTON_001
f1 (ppm)

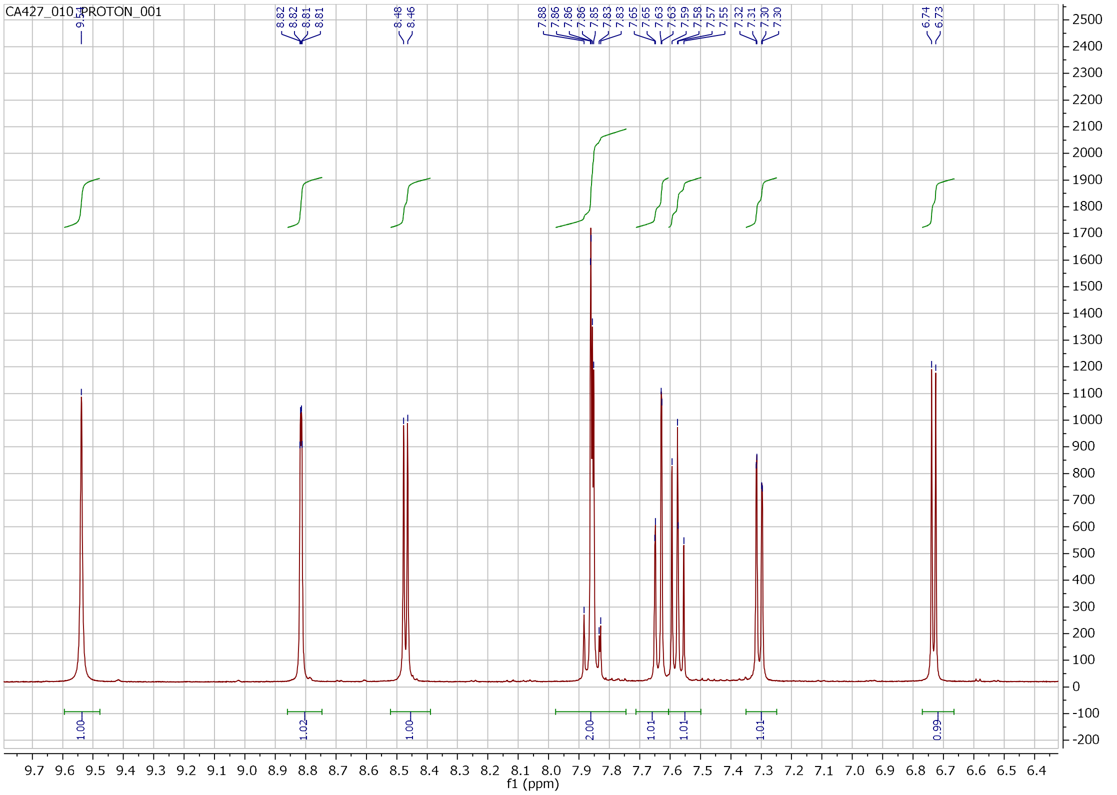
CA427_010_PROTON_001
f1 (ppm)

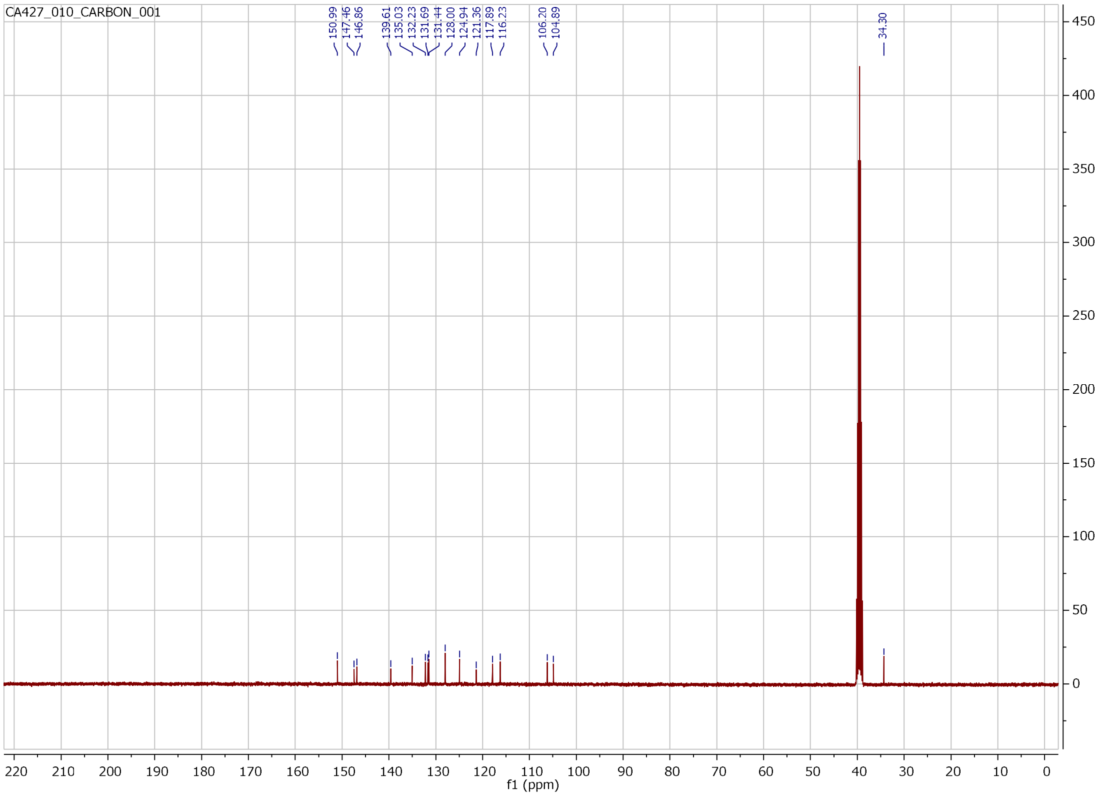

CA427_010_CARBON_001
150.99
147.46
146.86
139.61
135.03
132.23
131.69
131.44
128.00
124.94
121.36
117.89
116.23
106.20
104.89
34.30
f1 (ppm)

6-bromo-*N*-(1-methyl-1*H*-indazol-4-yl)quinolin-4-amine (**31**)

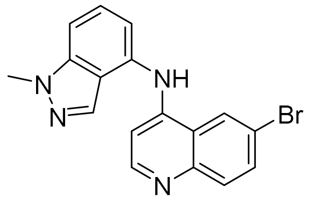

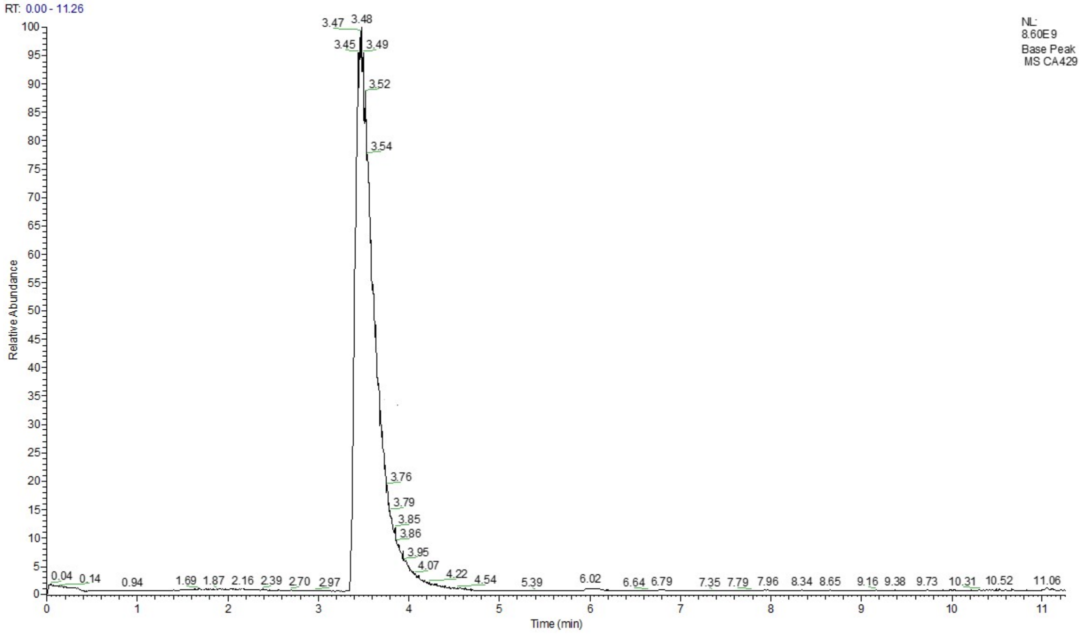

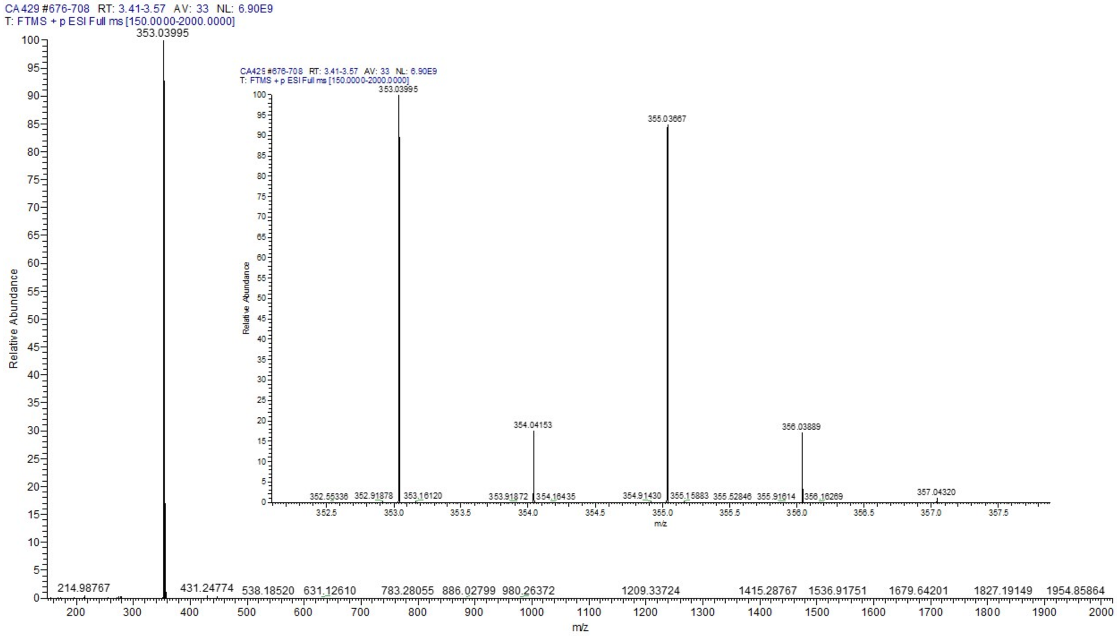

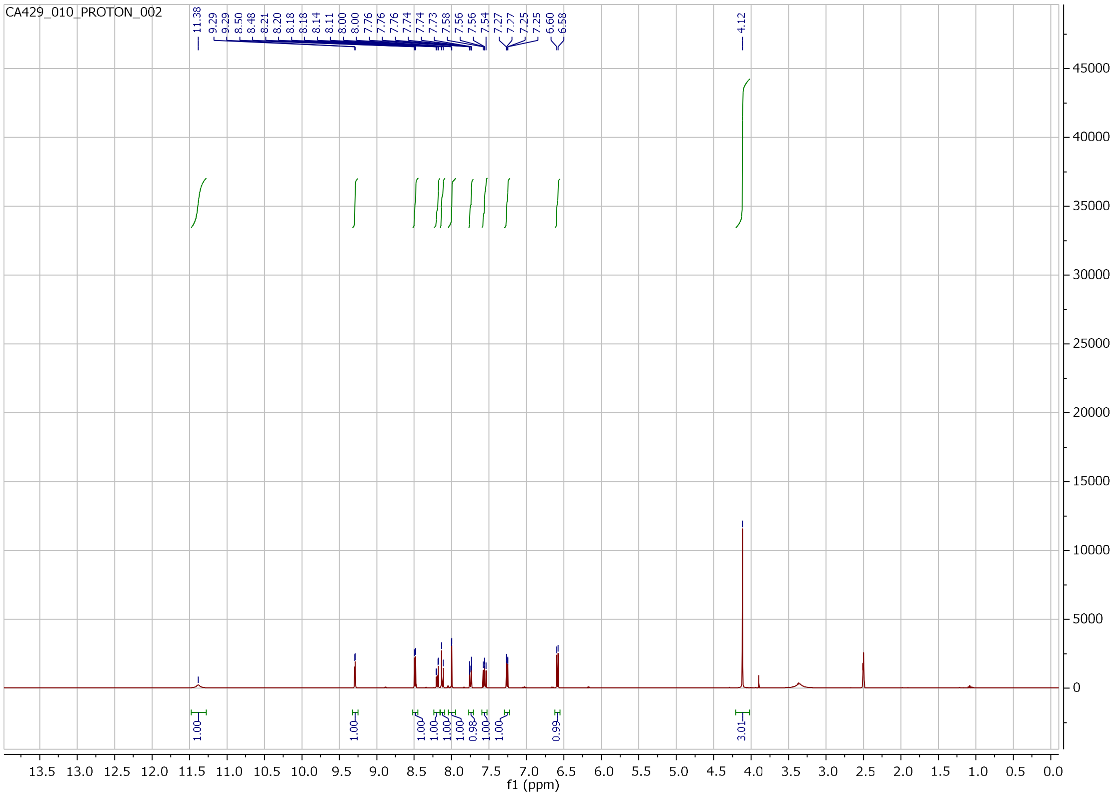
CA429_010_PROTON_002
f1 (ppm)

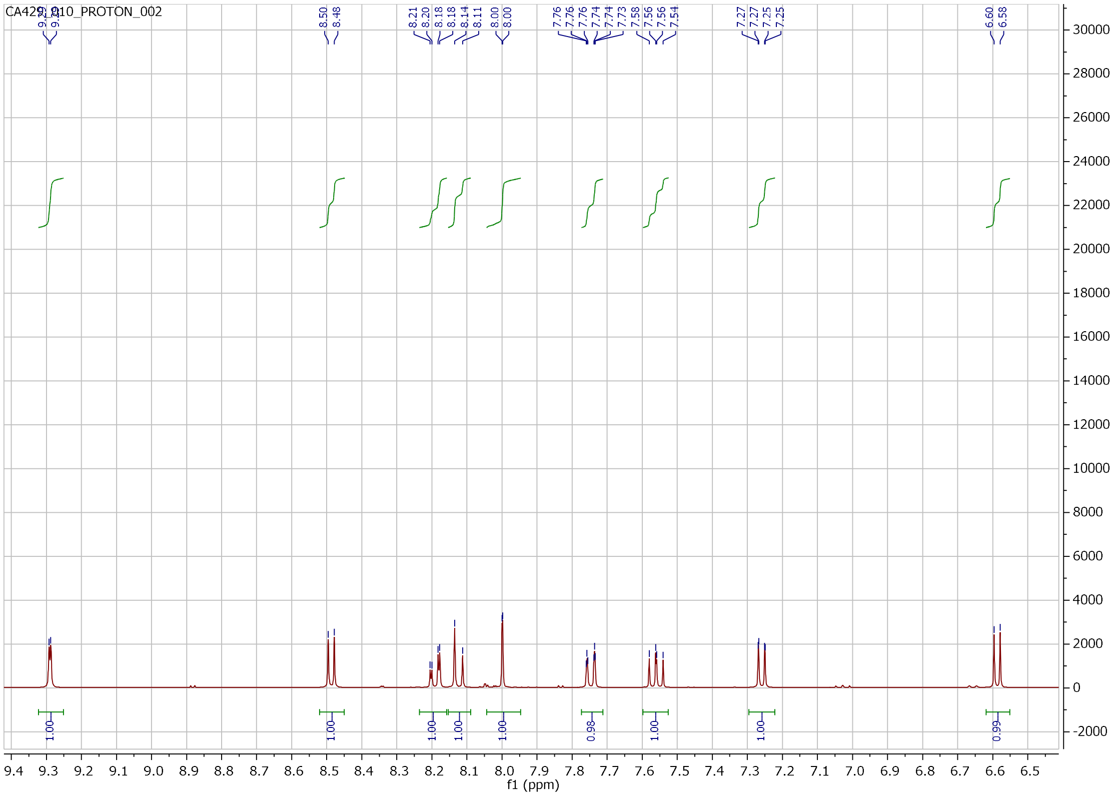
CA429_010_PROTON_002
f1 (ppm)

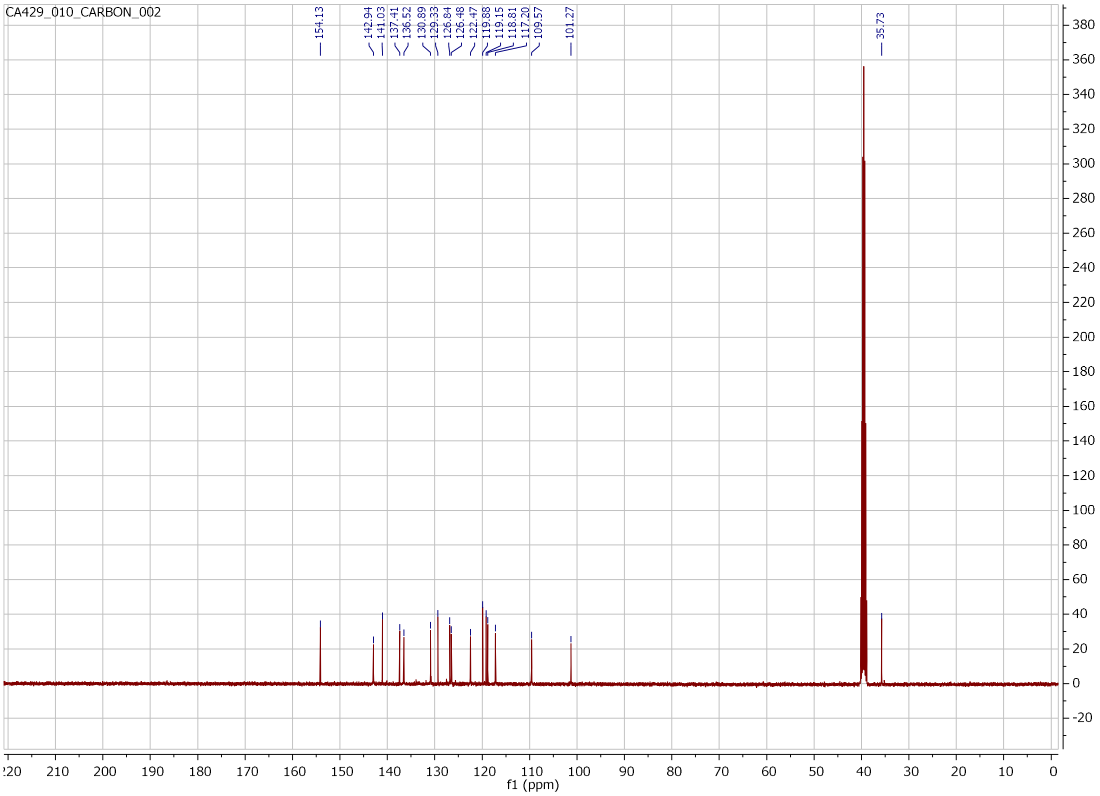
CA429_010_CARBON_002
154.13
142.94
141.03
137.41
136.52
130.89
129.33
126.84
126.48
122.47
119.88
119.15
118.81
117.20
109.57
101.27
35.73
f1 (ppm)

6-bromo-*N*-(1*H*-indazol-4-yl)quinolin-4-amine (**32**)

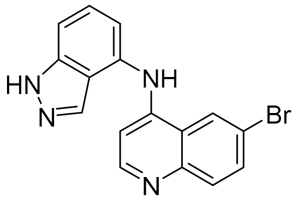

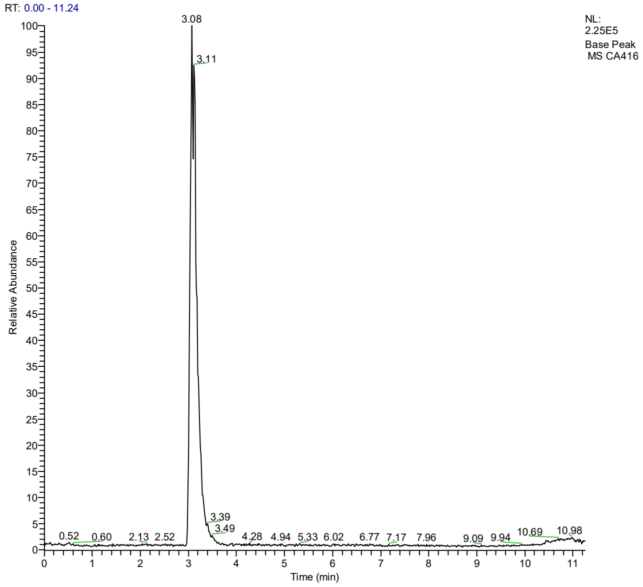

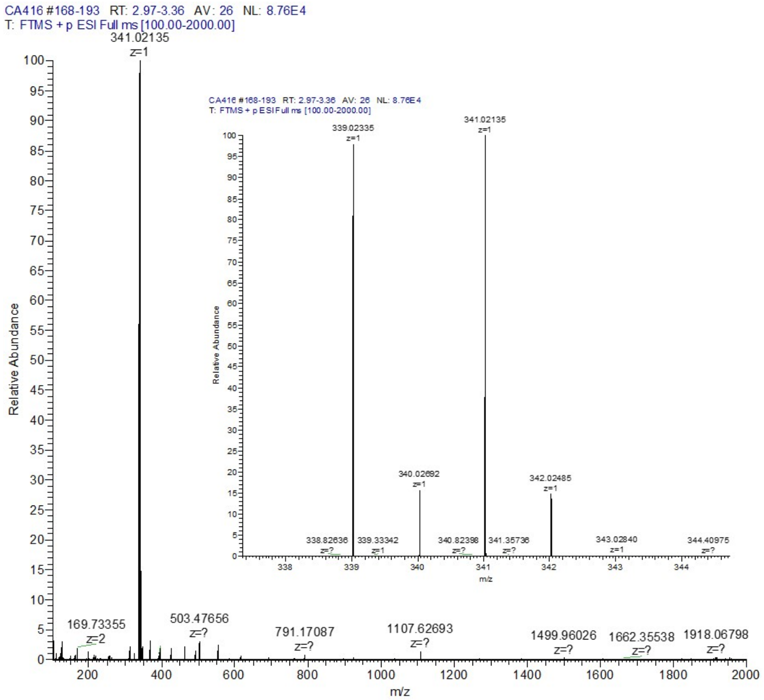

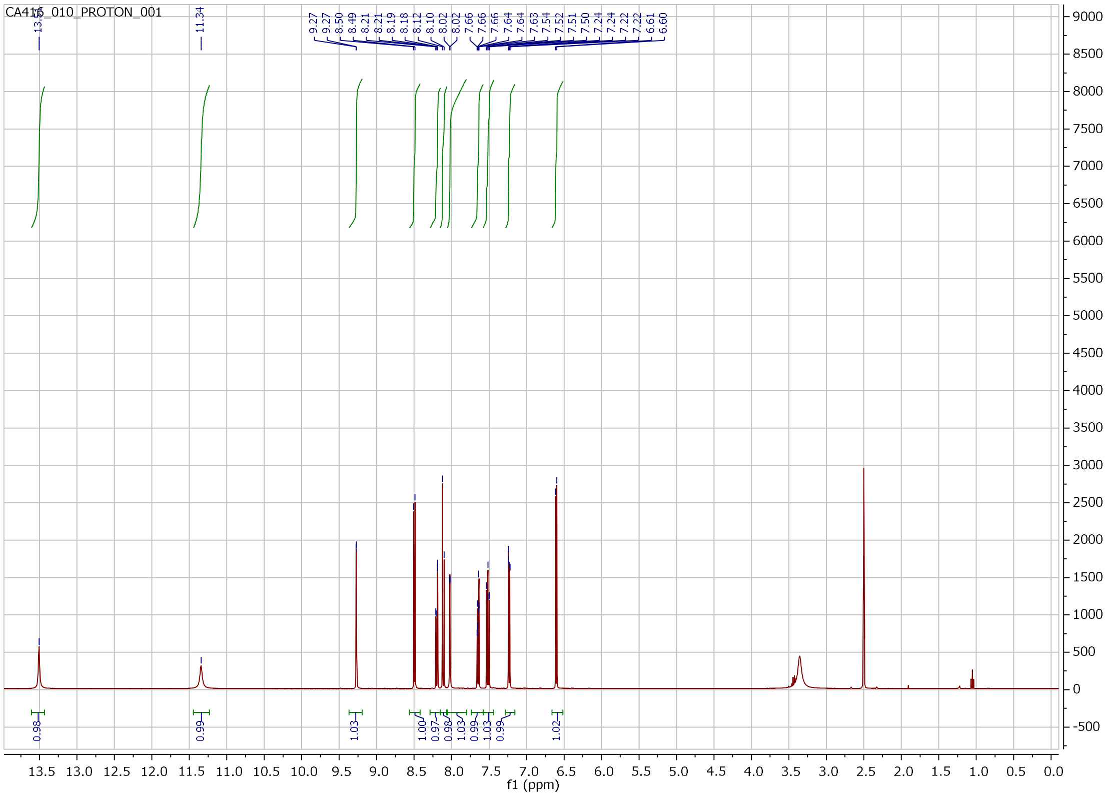

CA416_010_PROTON_001
f1 (ppm)

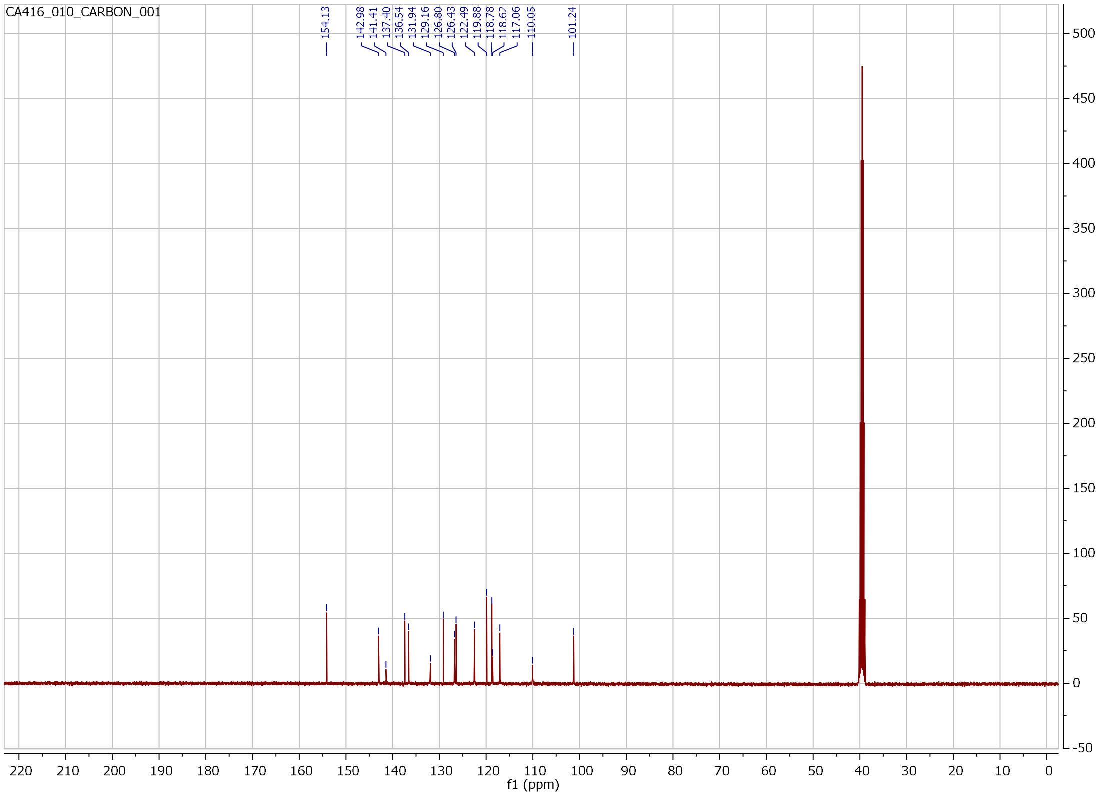

CA416_010_CARBON_001
154.13
142.98
141.41
137.40
136.54
131.94
129.16
126.80
126.43
122.49
119.88
118.78
118.62
117.06
110.05
101.24
f1 (ppm)

N-(1*H*-indazol-4-yl)-6-(trifluoromethyl)quinolin-4-amine (**33**)

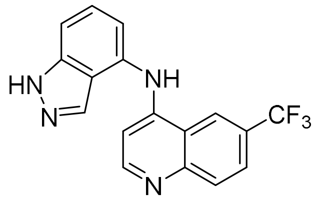

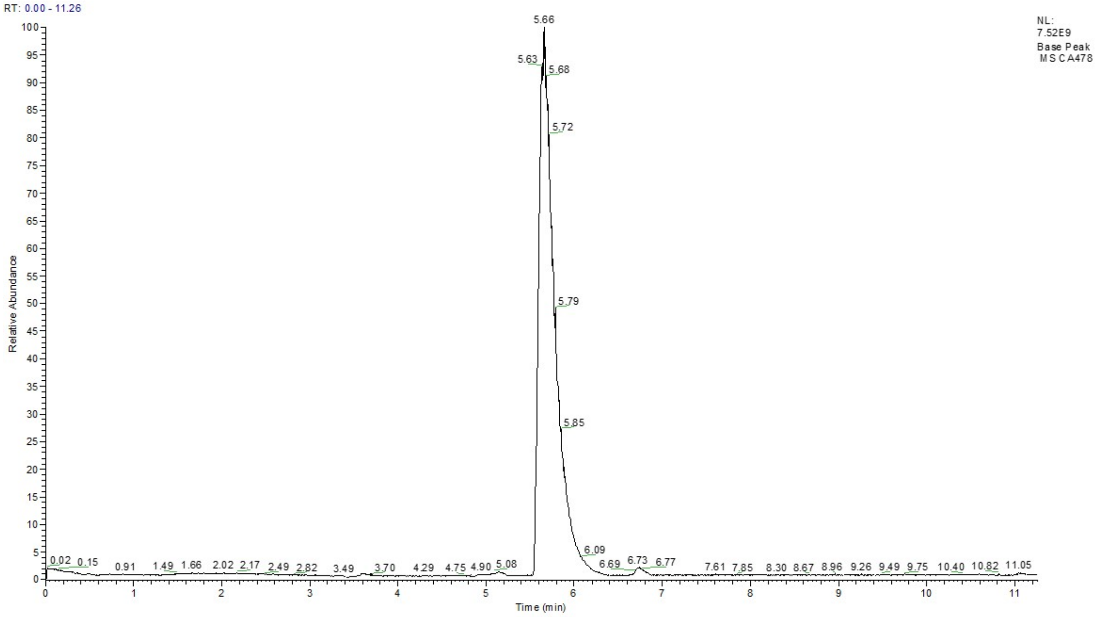

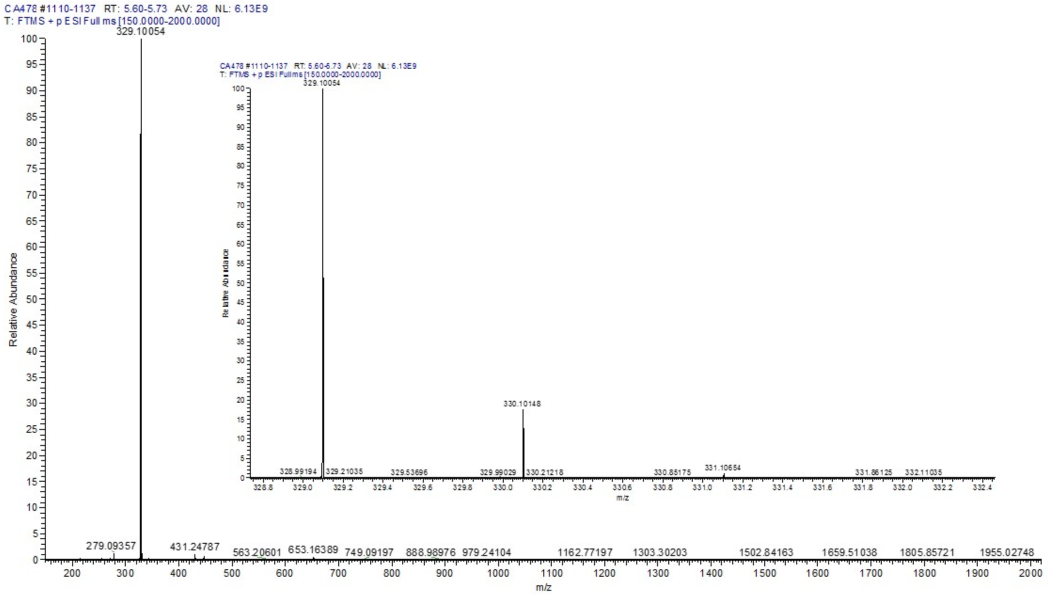

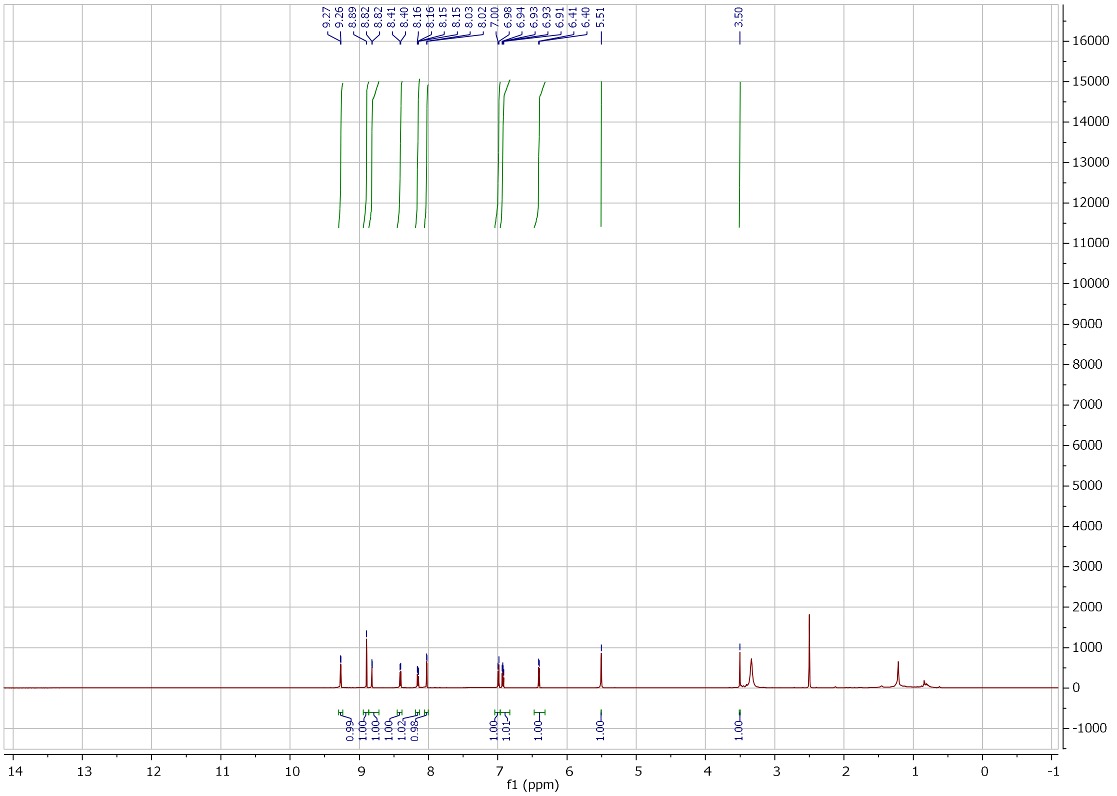

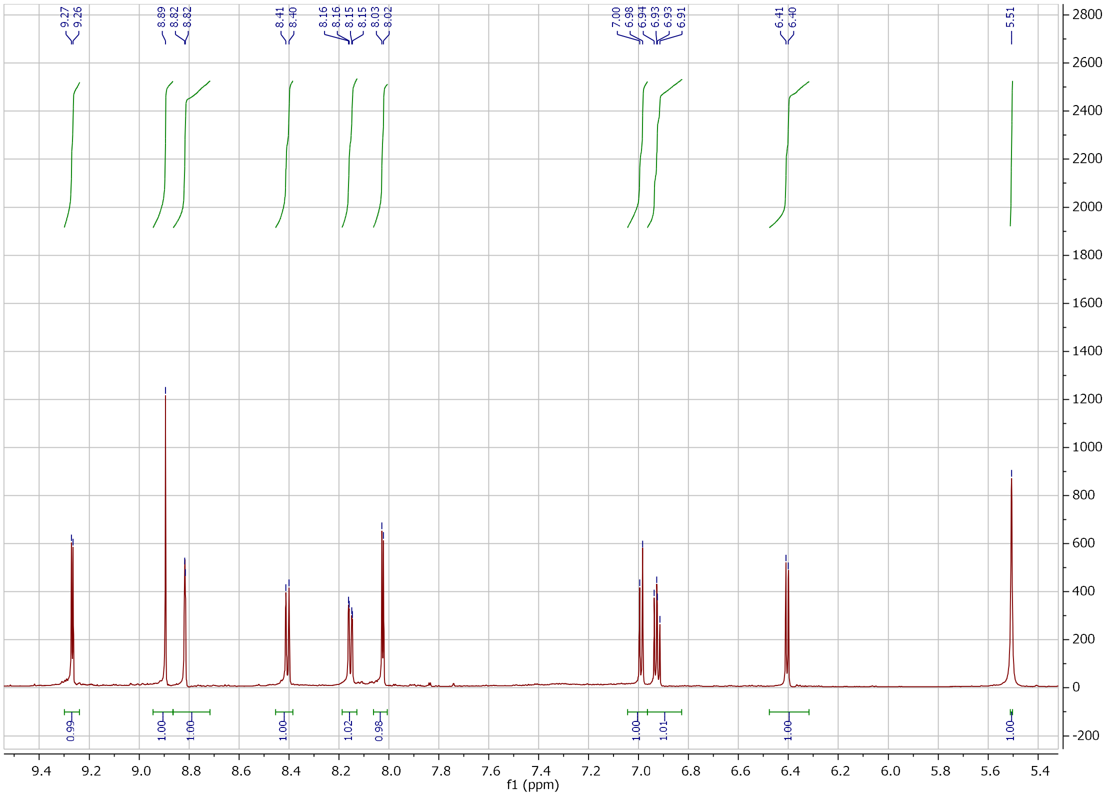

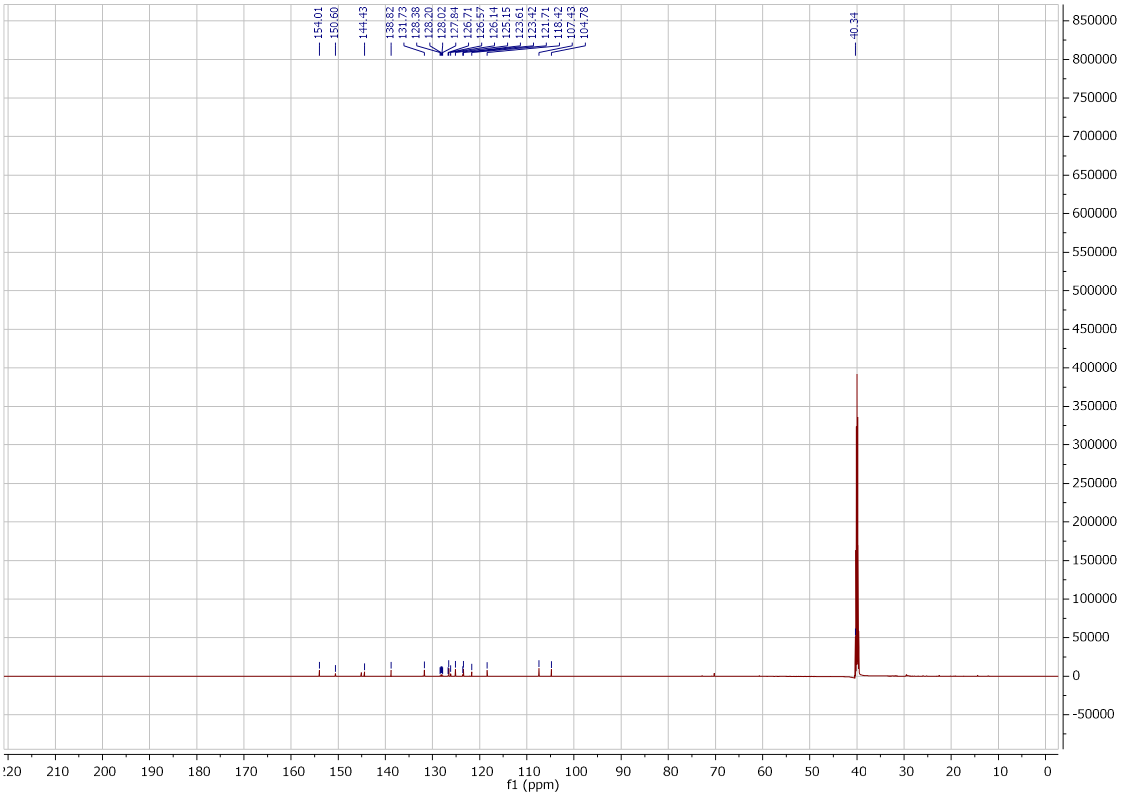

154.01
150.60
144.43
138.82
131.73
128.38
128.20
128.02
127.84
126.71
126.57
126.14
125.15
123.61
123.42
121.71
118.42
107.43
104.78
40.34
f1 (ppm)

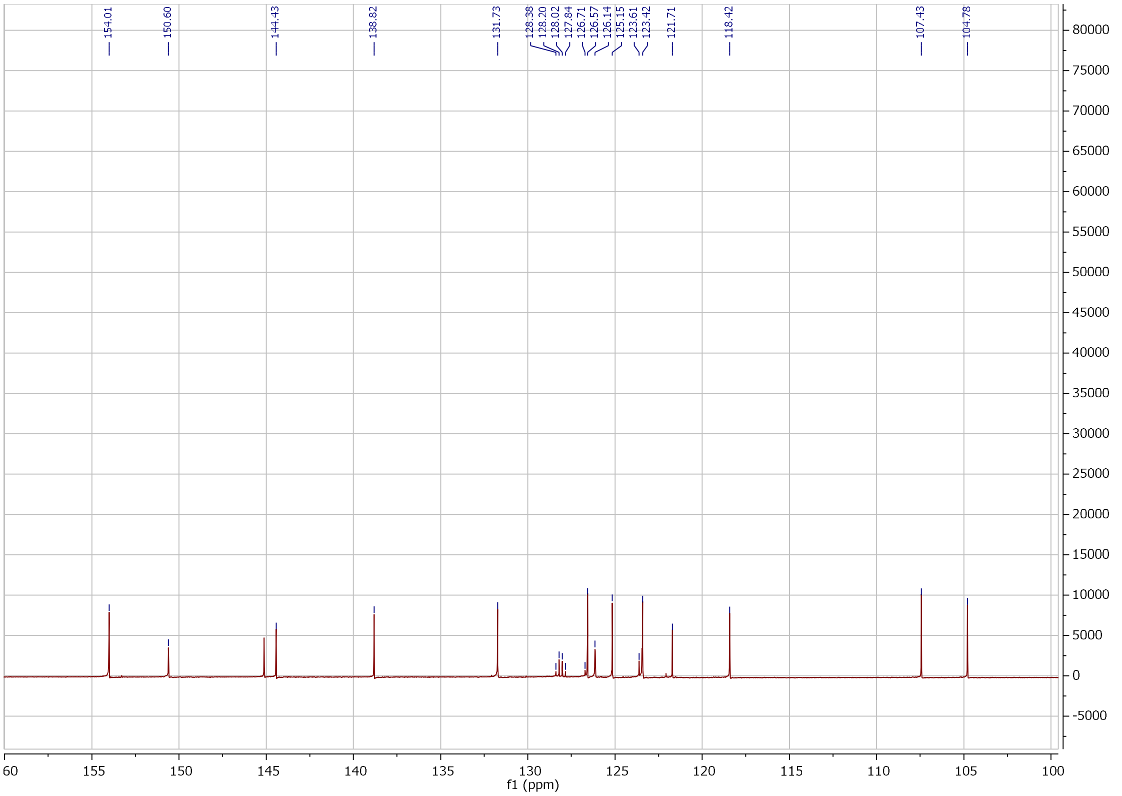

154.01
150.60
144.43
138.82
131.73
128.38
128.20
128.02
127.84
126.71
126.57
126.14
125.15
123.61
123.42
121.71
118.42
107.43
104.78
f1 (ppm)

N-(1*H*-indazol-7-yl)-6-(trifluoromethyl)quinolin-4-amine (**34**)

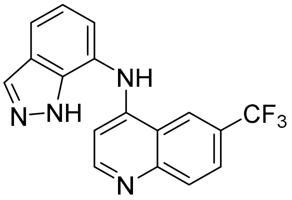

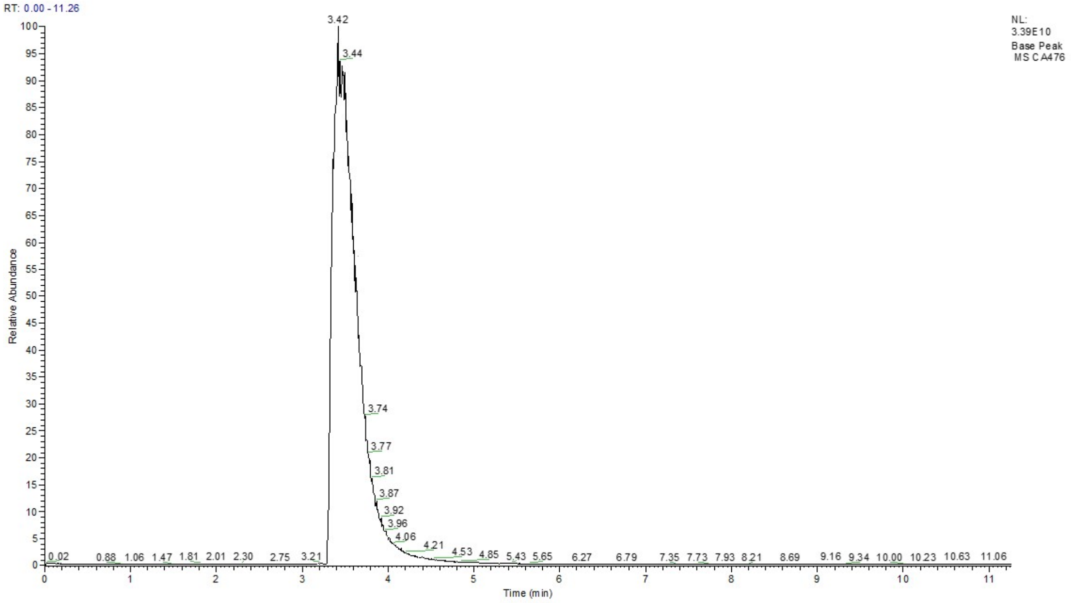

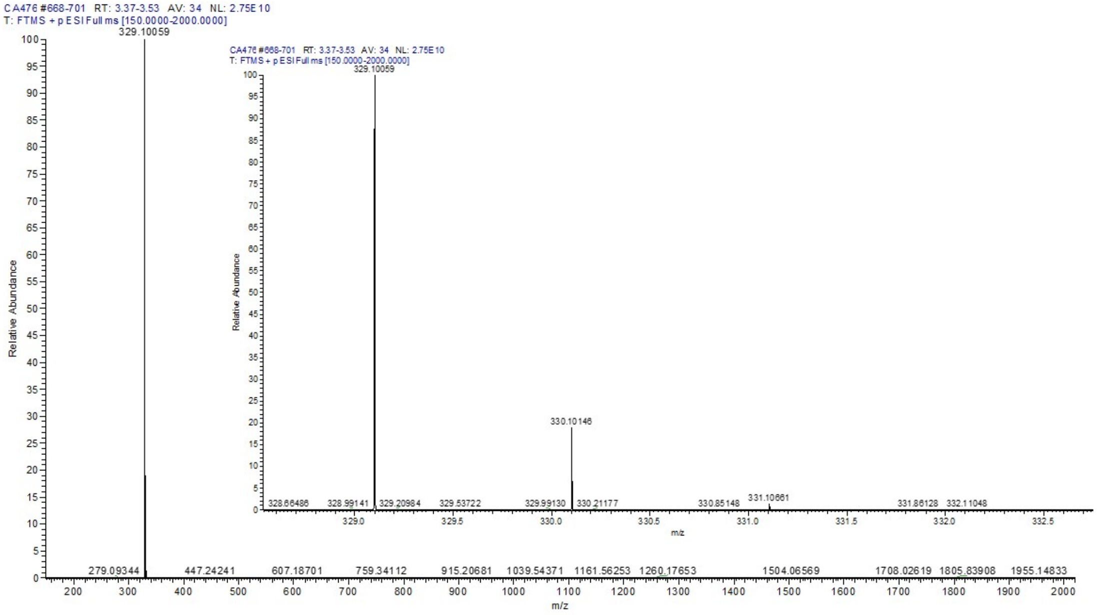

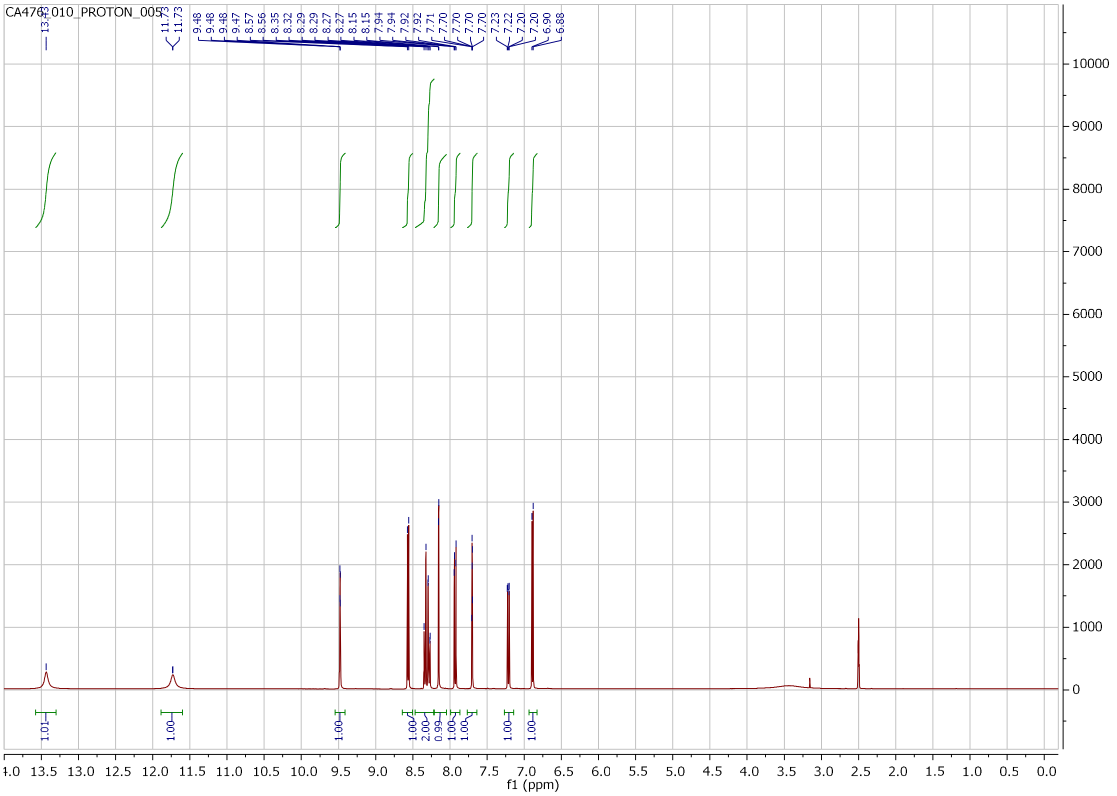

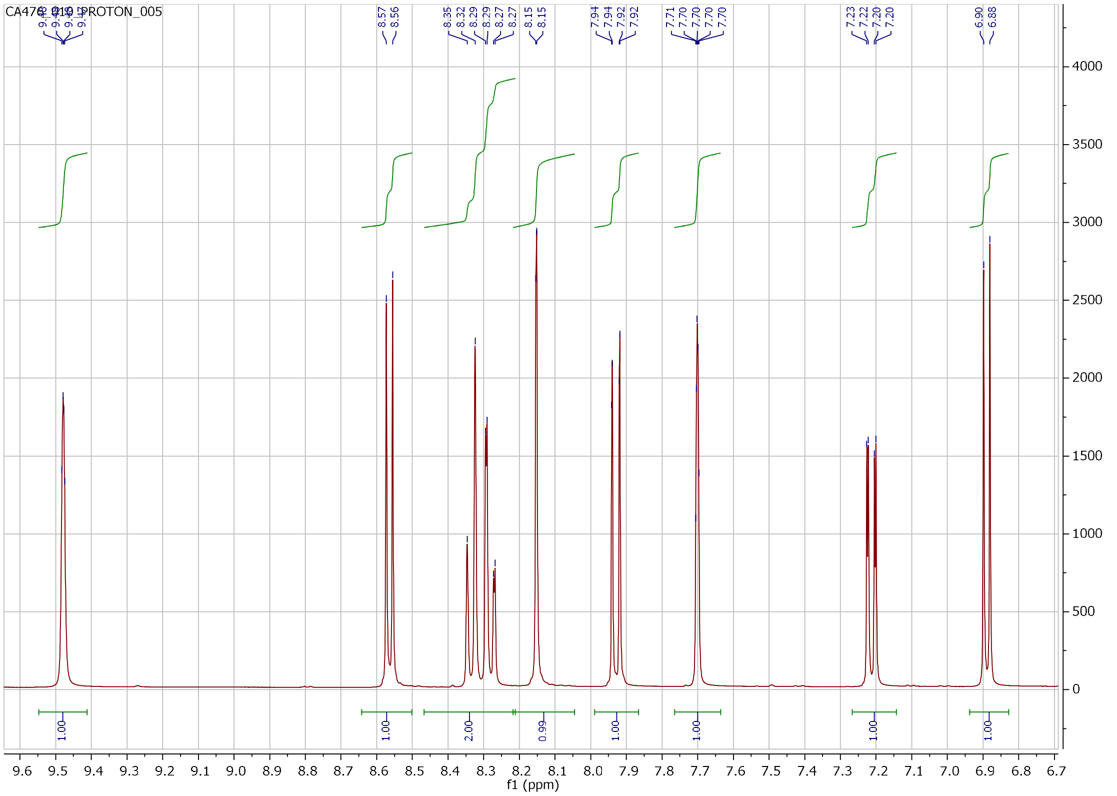

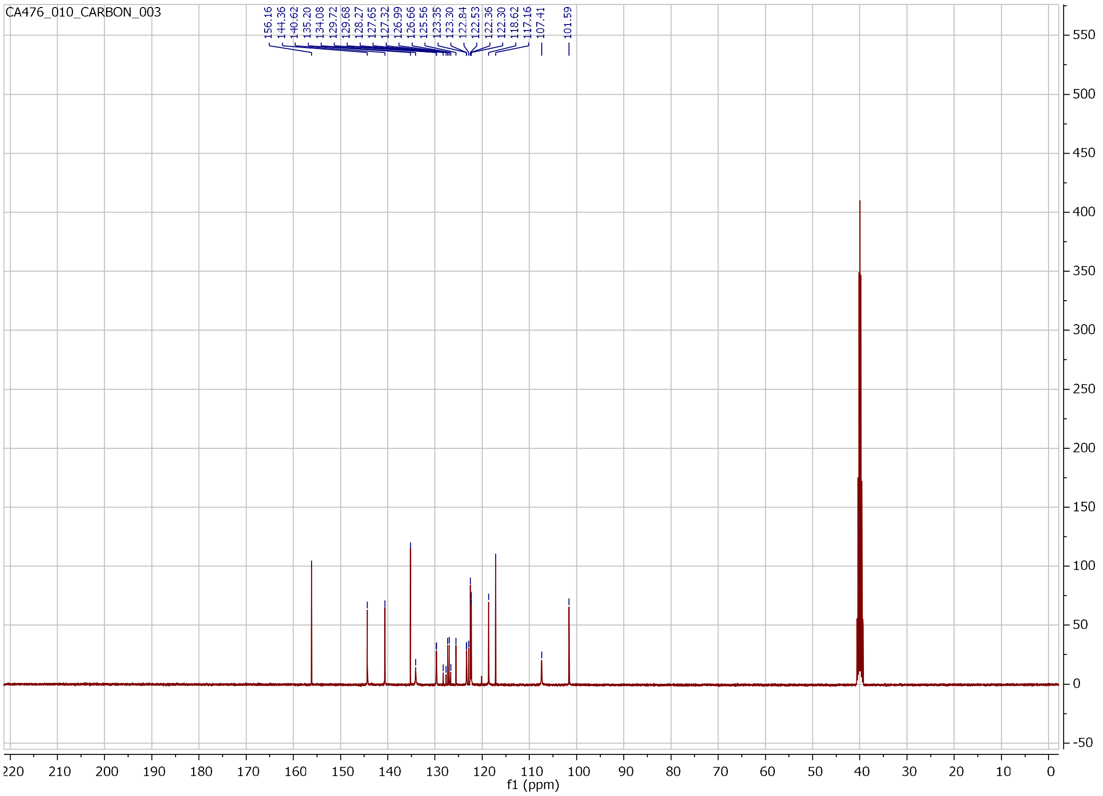
CA476_010_CARBON_003
156.16
144.36
140.62
135.20
134.08
129.72
129.68
128.27
127.65
127.32
126.99
126.66
125.56
123.35
123.30
122.84
122.53
122.36
122.30
118.62
117.16
107.41
101.59
f1 (ppm)

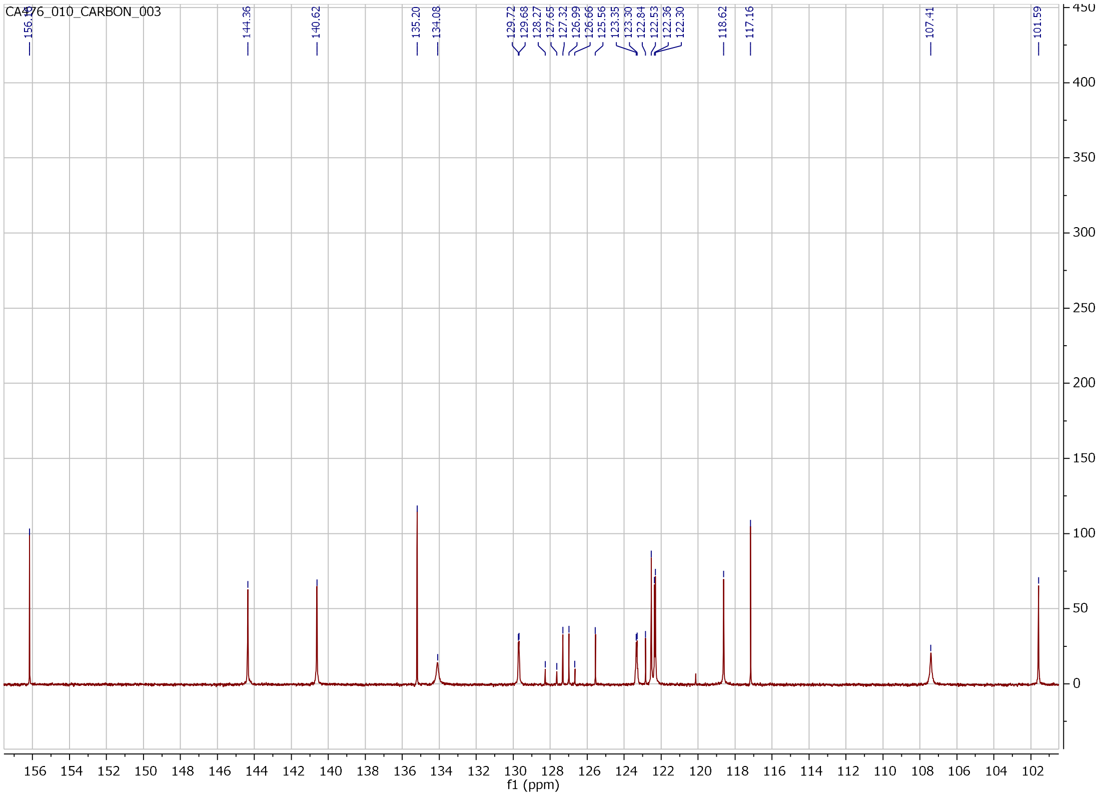
CA476_010_CARBON_003
144.36
140.62
135.20
134.08
129.72
129.68
128.27
127.65
127.32
126.99
126.66
125.56
123.35
123.30
122.84
122.53
122.36
122.30
118.62
117.16
107.41
101.59
f1 (ppm)

6-bromo-*N*-(1*H*-indazol-6-yl)quinolin-4-amine (**35**)

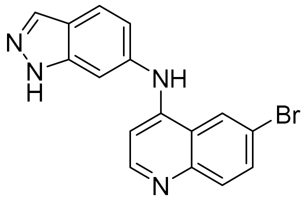

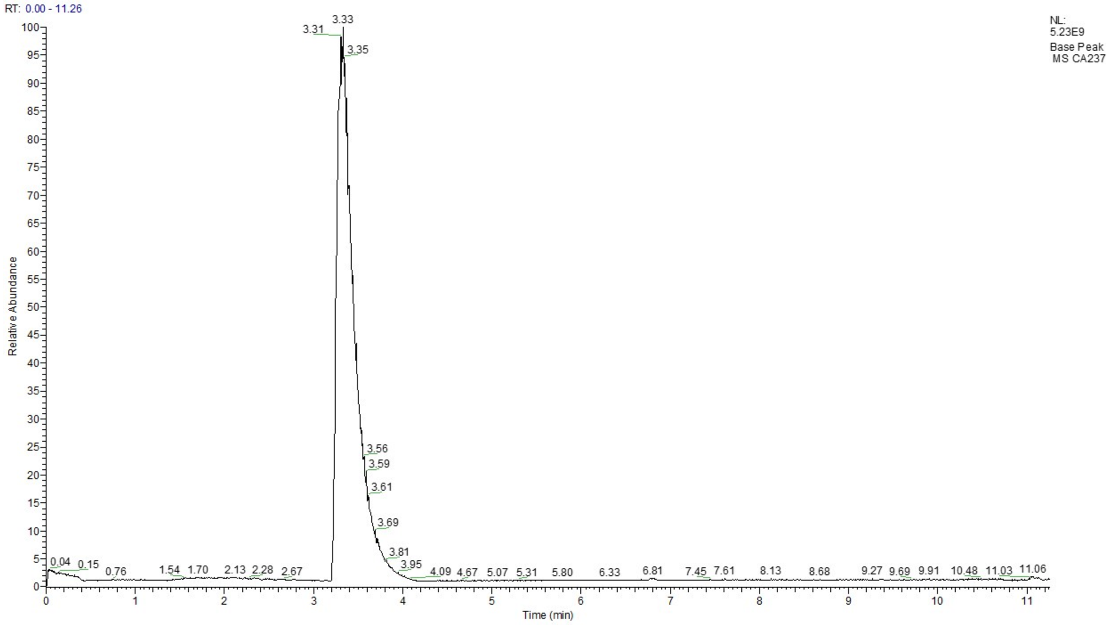

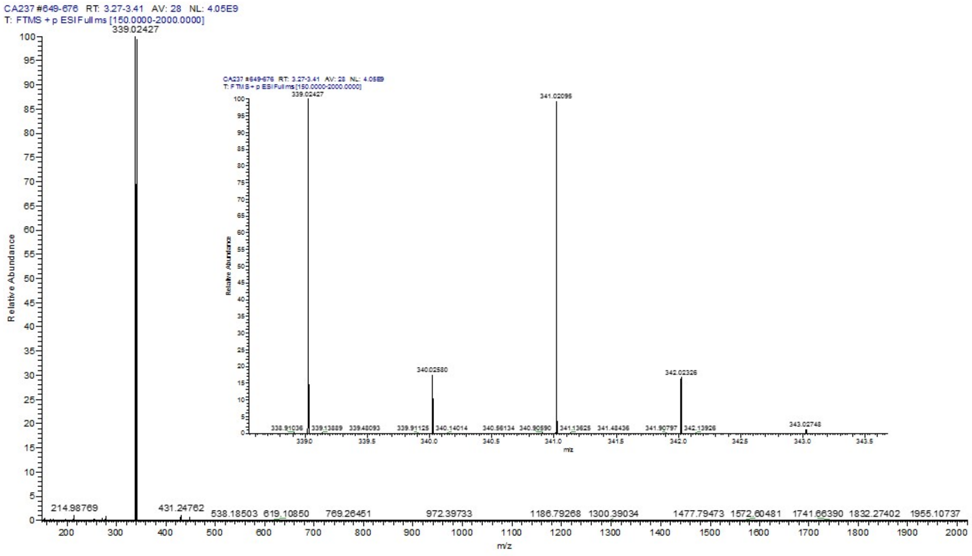

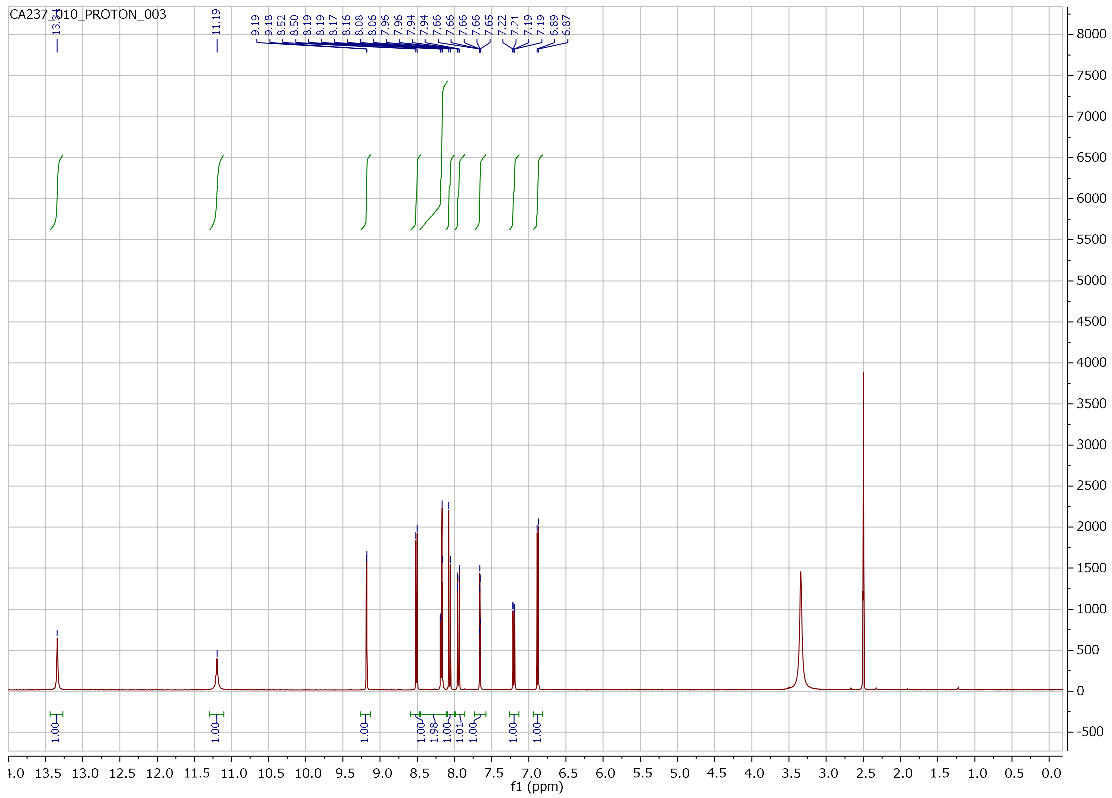
CA237_010_PROTON_003
f1 (ppm)

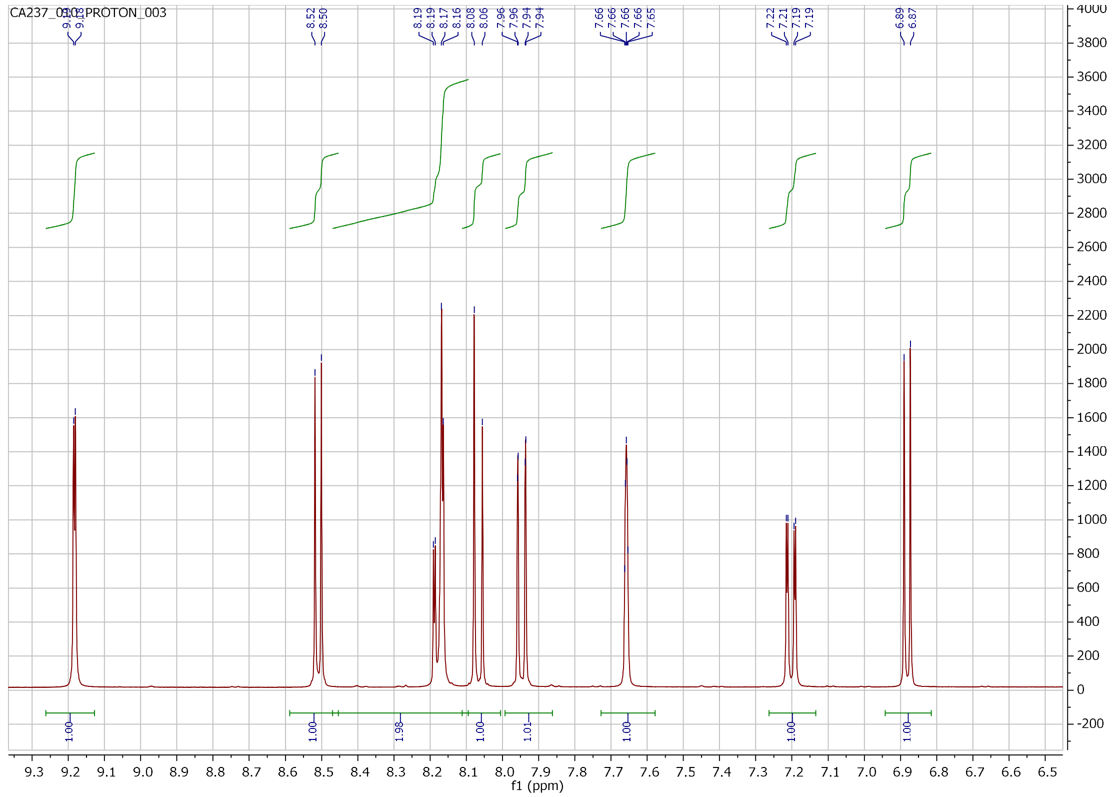
CA237_010_PROTON_003
f1 (ppm)

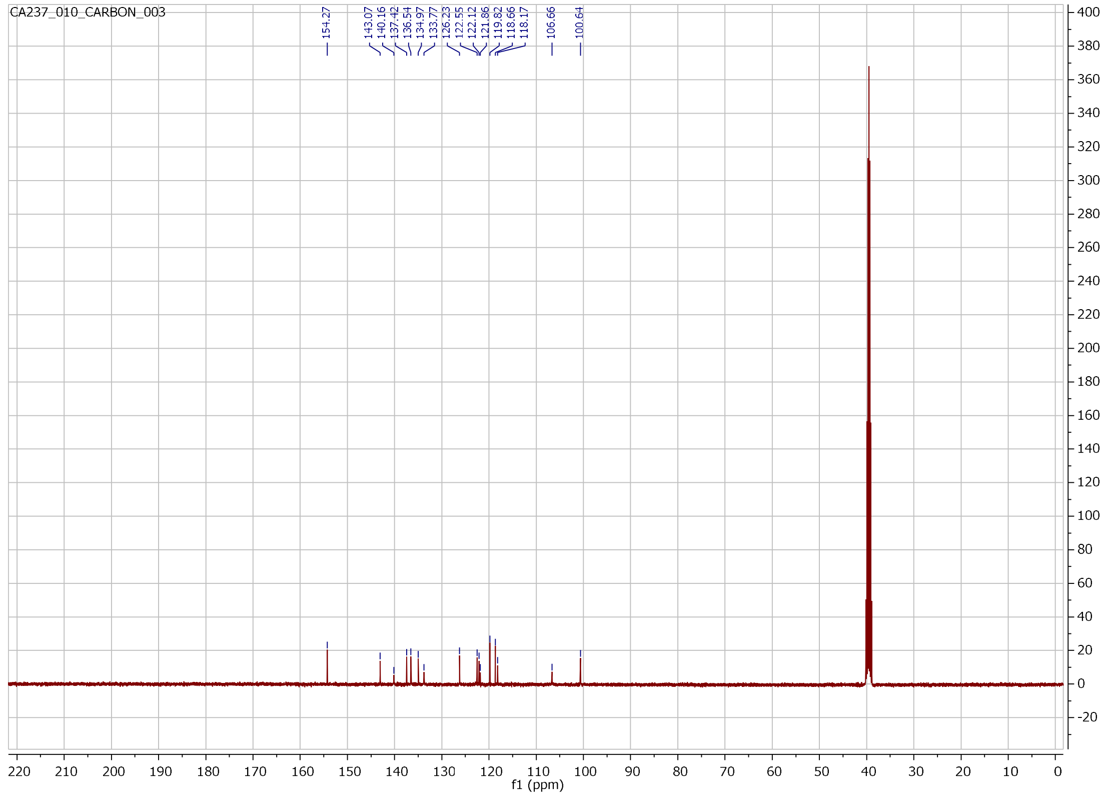
CA237_010_CARBON_003
154.27
143.07
140.16
137.42
136.54
134.97
133.77
126.23
122.55
122.12
121.86
119.82
118.66
118.17
106.66
100.64
f1 (ppm)

6-bromo-*N*-(5-fluoro-1*H*-indazol-6-yl)quinolin-4-amine (**36**)

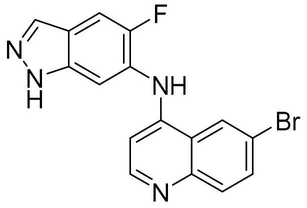

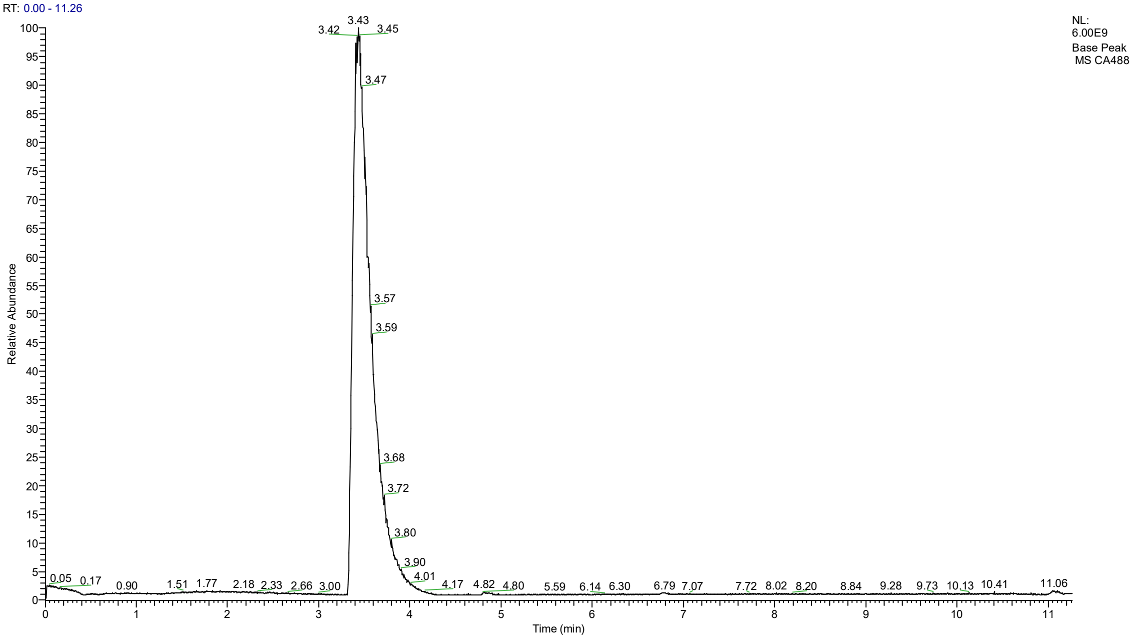

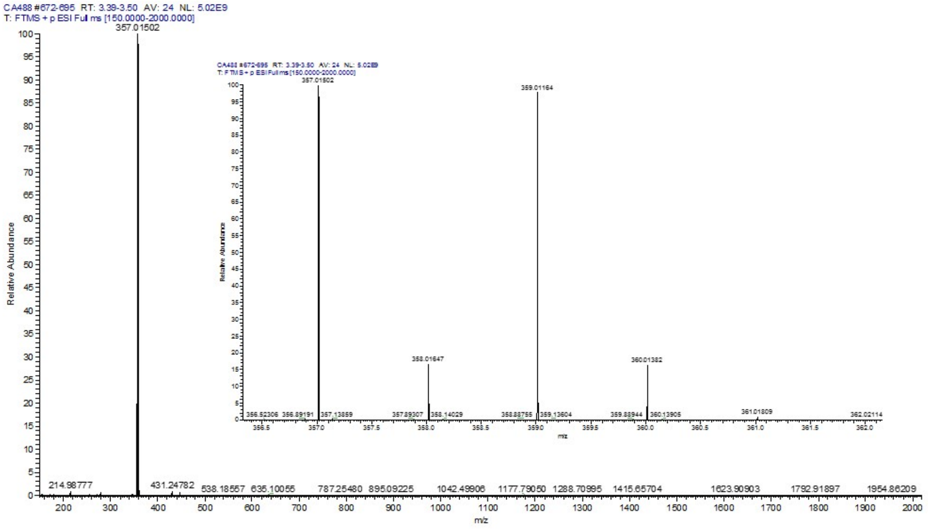

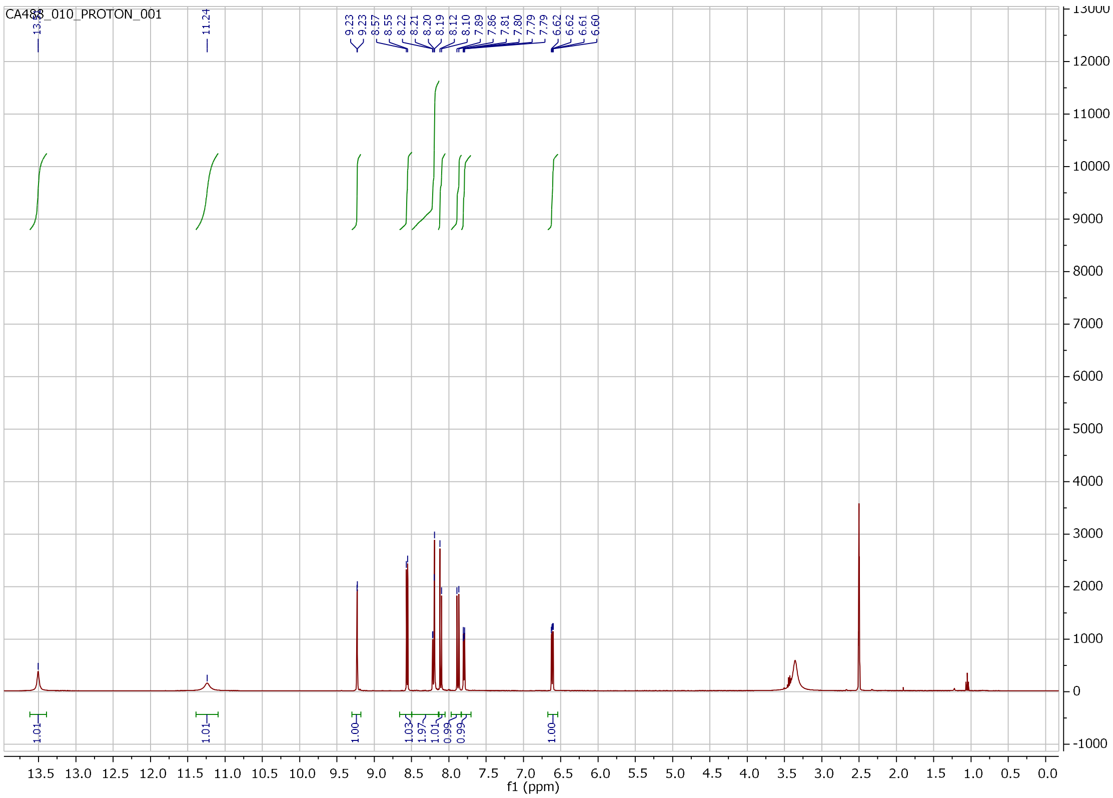

CA488_010_PROTON_001
f1 (ppm)

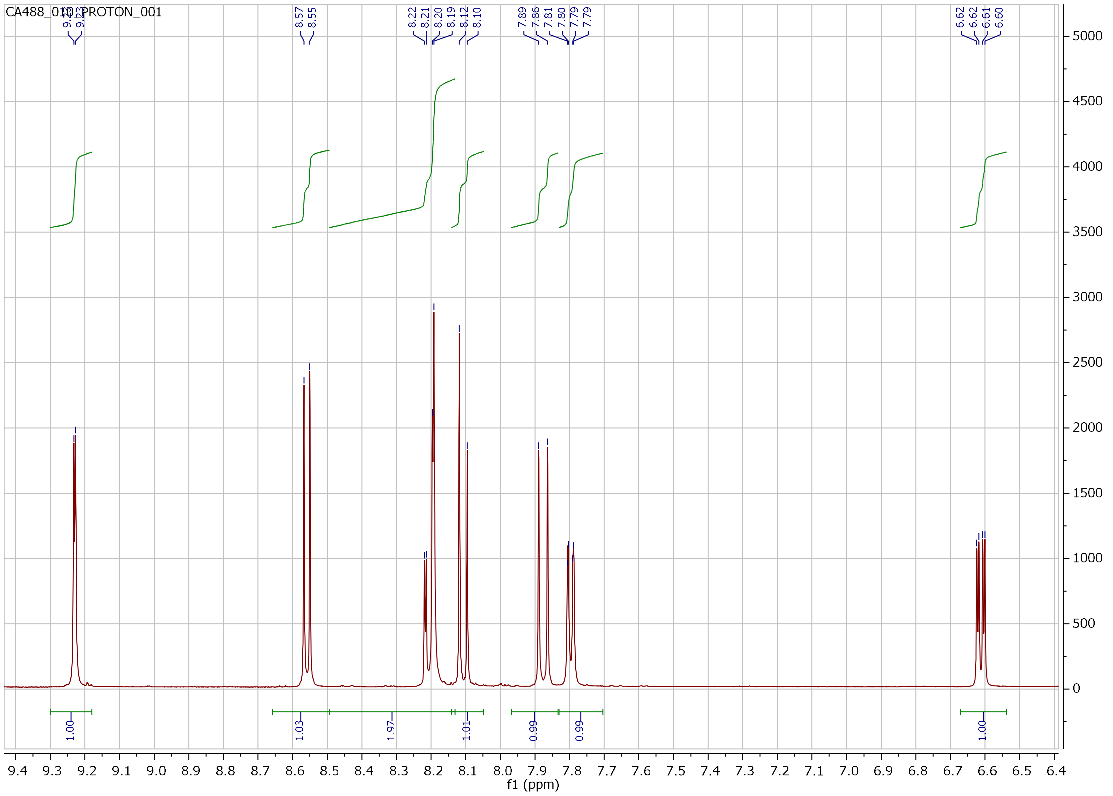

CA488_010_PROTON_001
f1 (ppm)

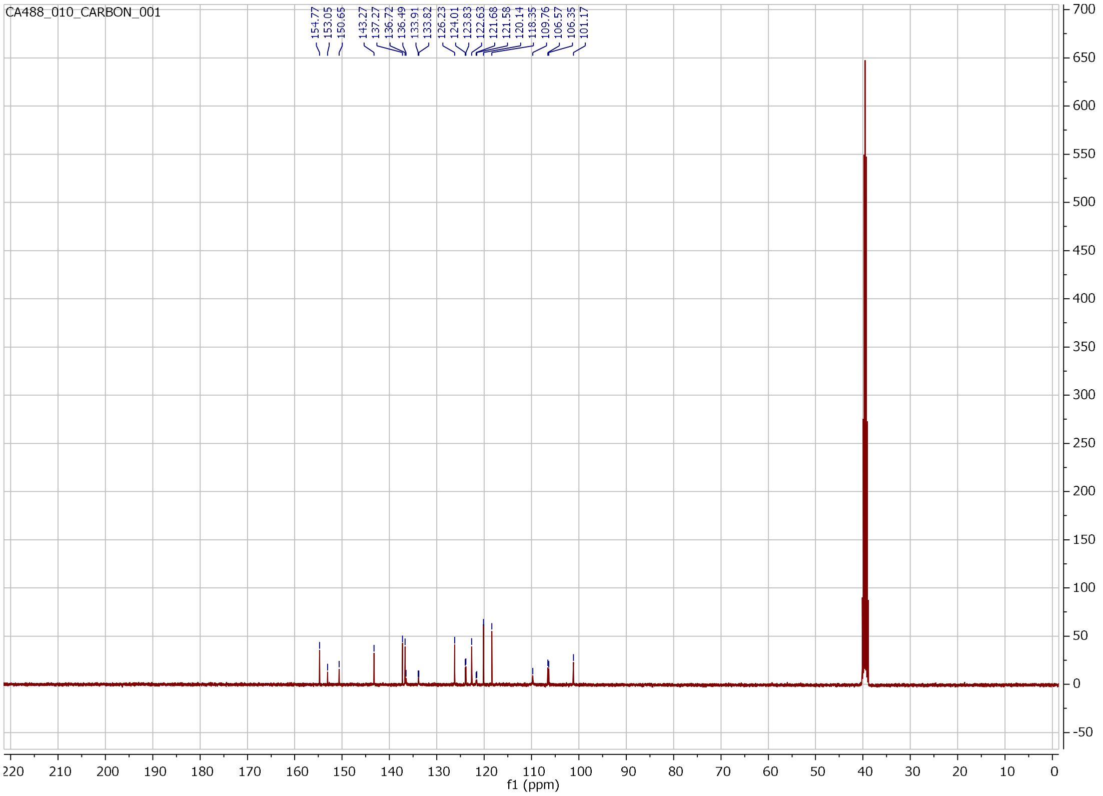
CA488_010_CARBON_001
154.77
153.05
150.65
143.27
137.27
136.72
136.49
133.91
133.82
126.23
124.01
123.83
122.63
121.68
121.58
120.14
118.35
109.76
106.57
106.35
101.17
f1 (ppm)

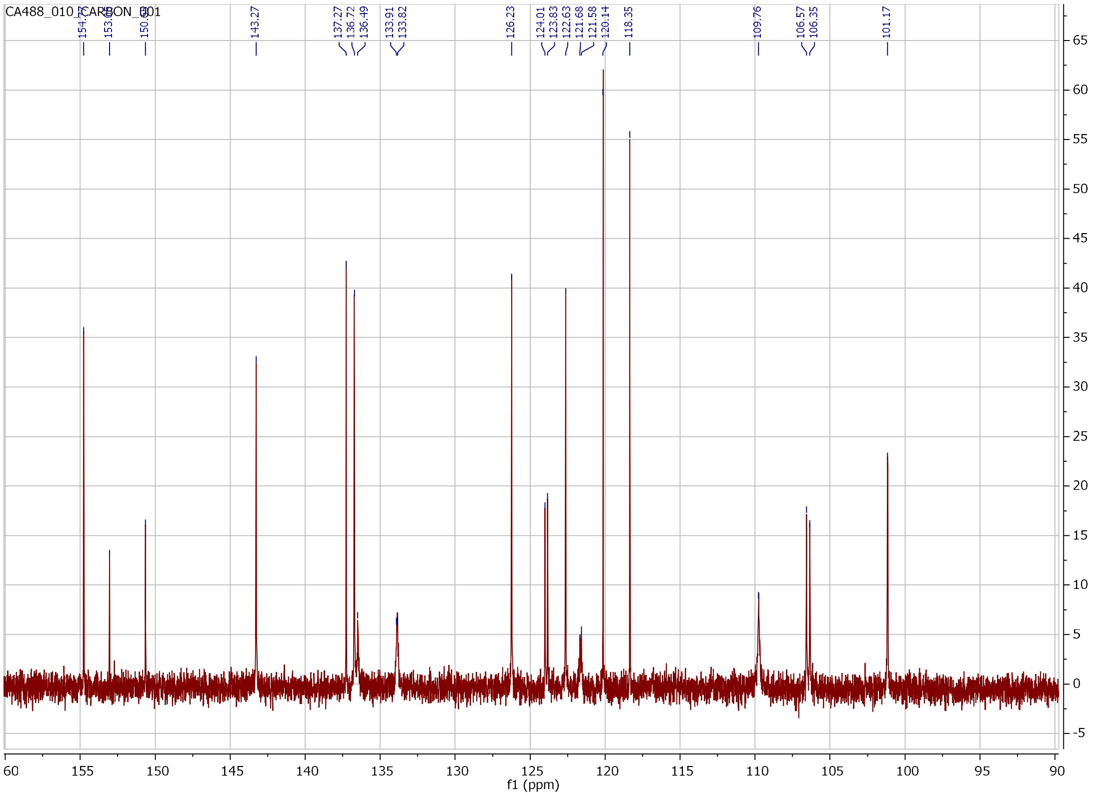
CA488_010_CARBON_001
143.27
137.27
136.72
136.49
133.91
133.82
126.23
124.01
123.83
122.63
121.68
121.58
120.14
118.35
109.76
106.57
106.35
101.17
f1 (ppm)

6-bromo-*N*-(1*H*-indazol-5-yl)quinolin-4-amine (**37**)

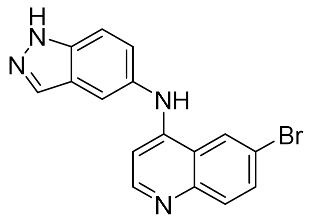

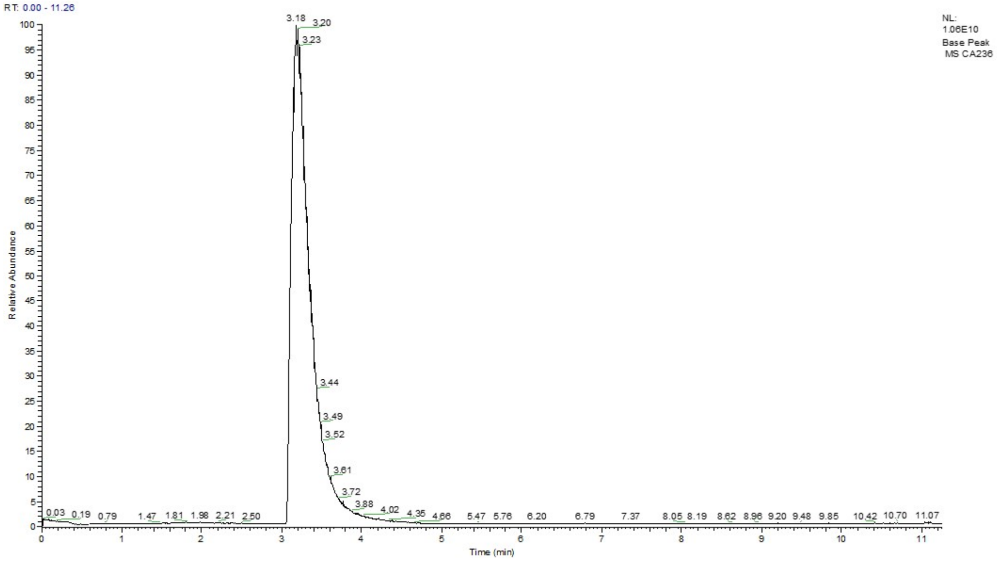

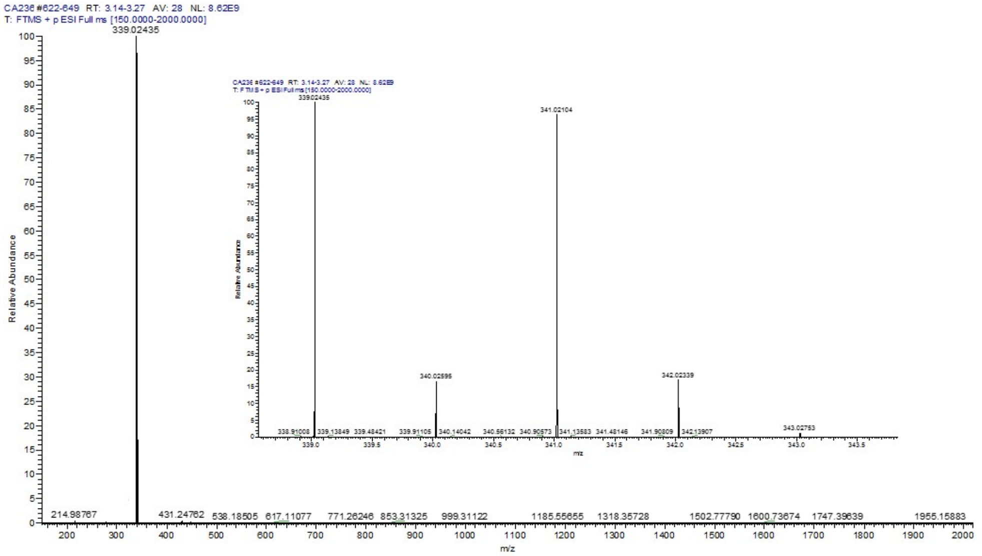

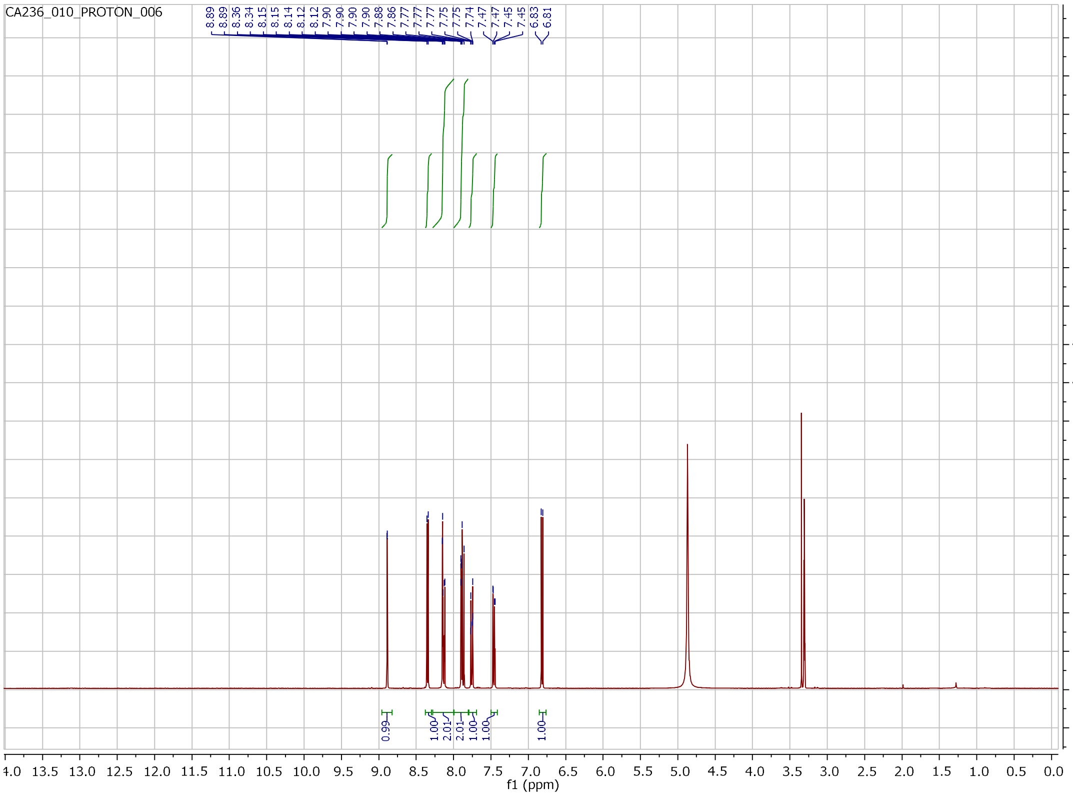
CA236_010_PROTON_006
f1 (ppm)

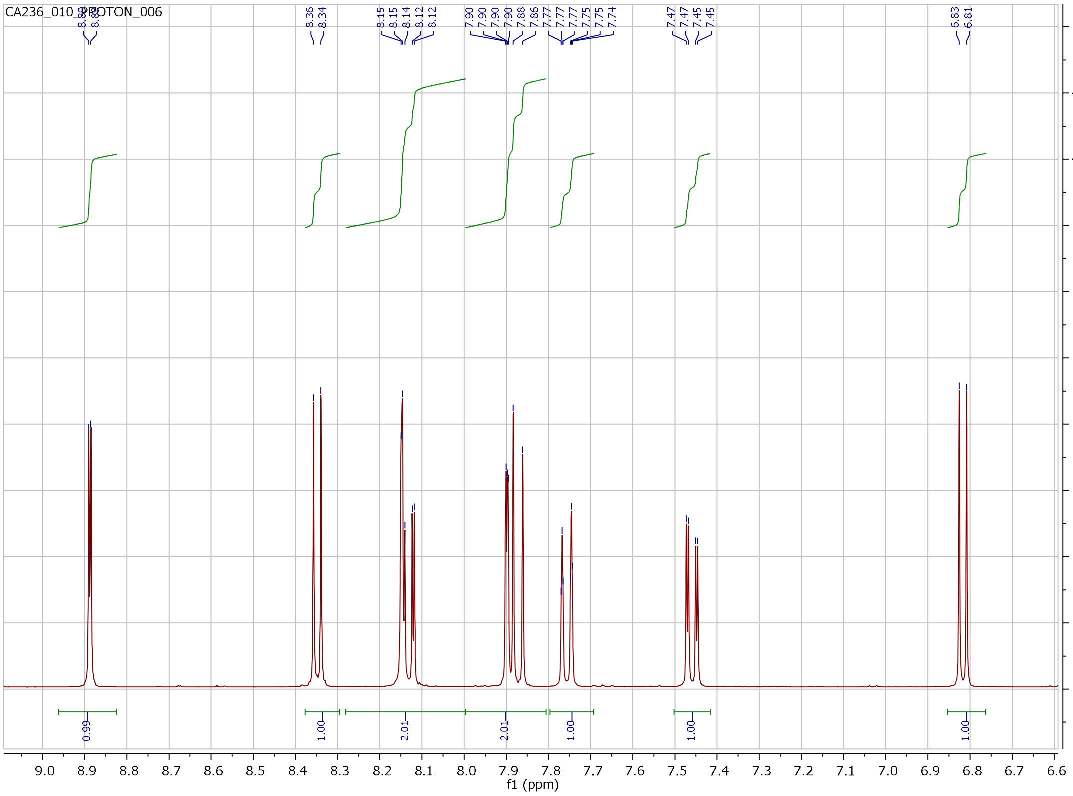
CA236_010_PROTON_006
f1 (ppm)

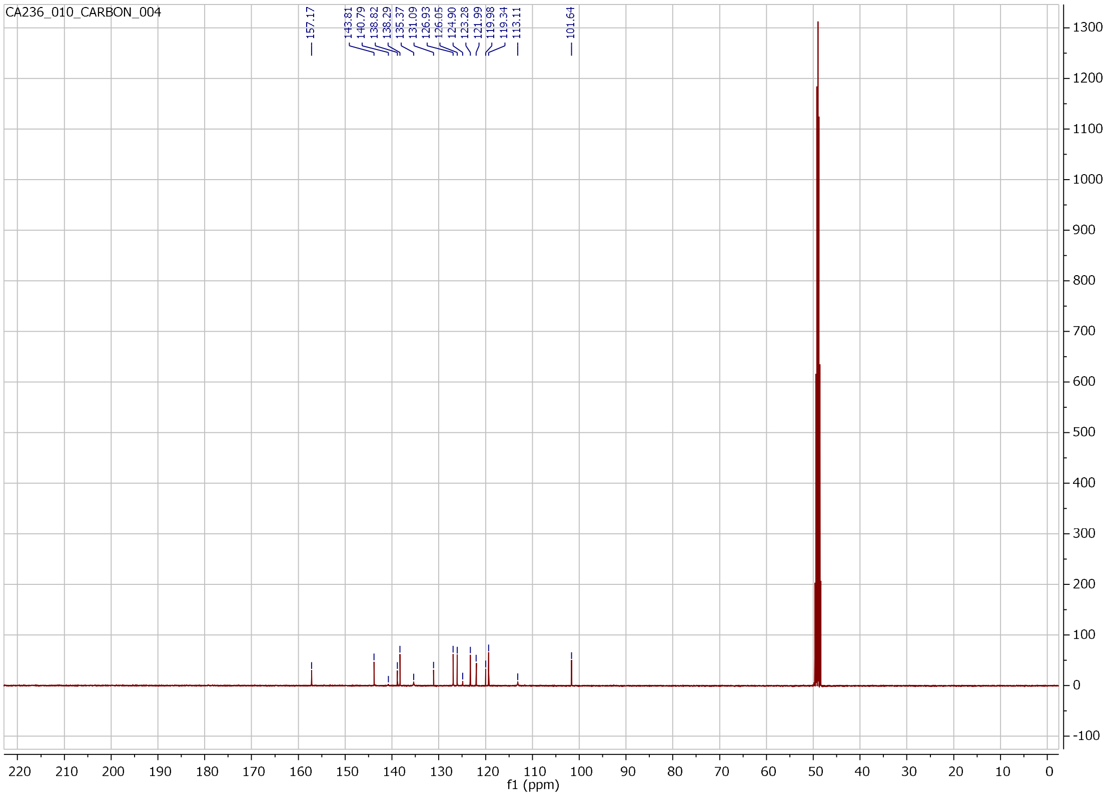
CA236_010_CARBON_004
157.17
143.81
140.79
138.82
138.29
135.37
131.09
126.93
126.05
124.90
123.28
121.99
119.98
119.34
113.11
101.64
f1 (ppm)

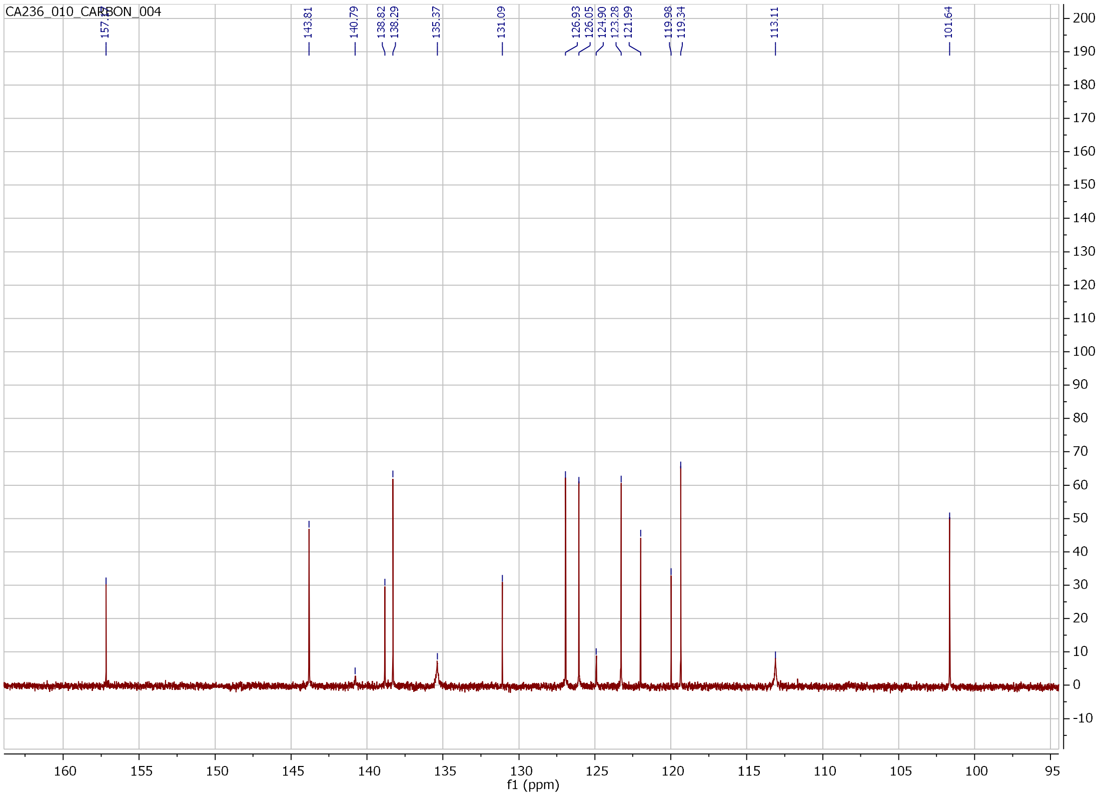
CA236_010_CARBON_004
143.81
140.79
138.82
138.29
135.37
131.09
126.93
126.05
124.90
123.28
121.99
119.98
119.34
113.11
101.64
f1 (ppm)

6-[(6-bromoquinolin-4-yl)amino]-2,3-dihydro-1*H*-isoindol-1-one (**38**)

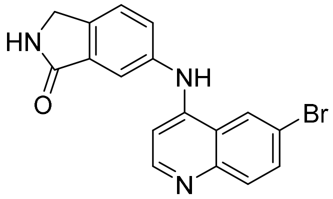

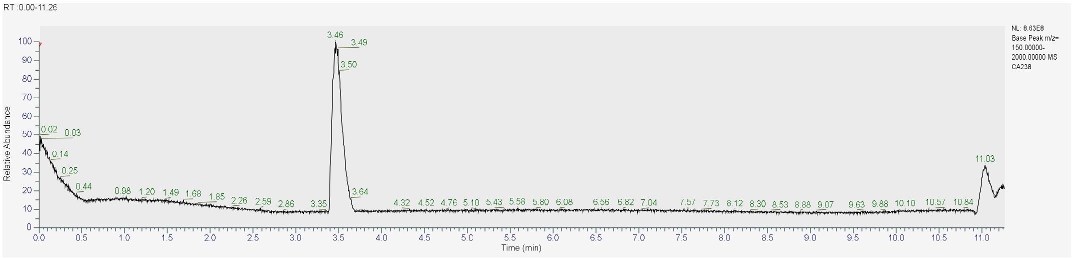

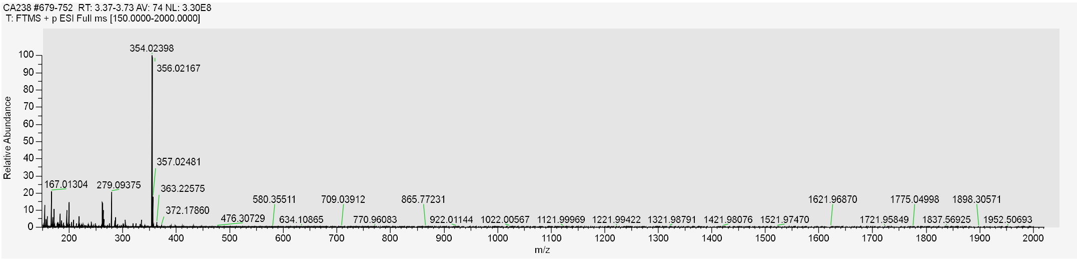

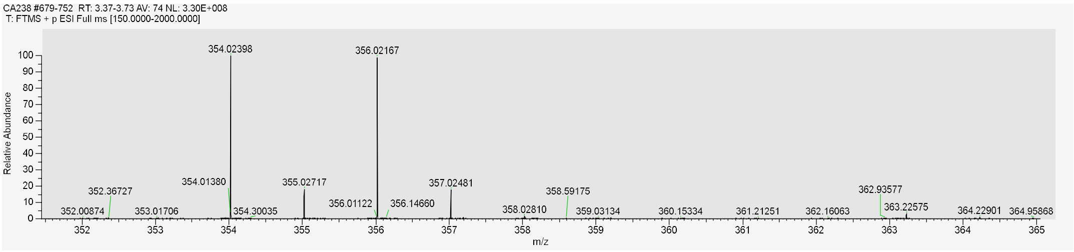

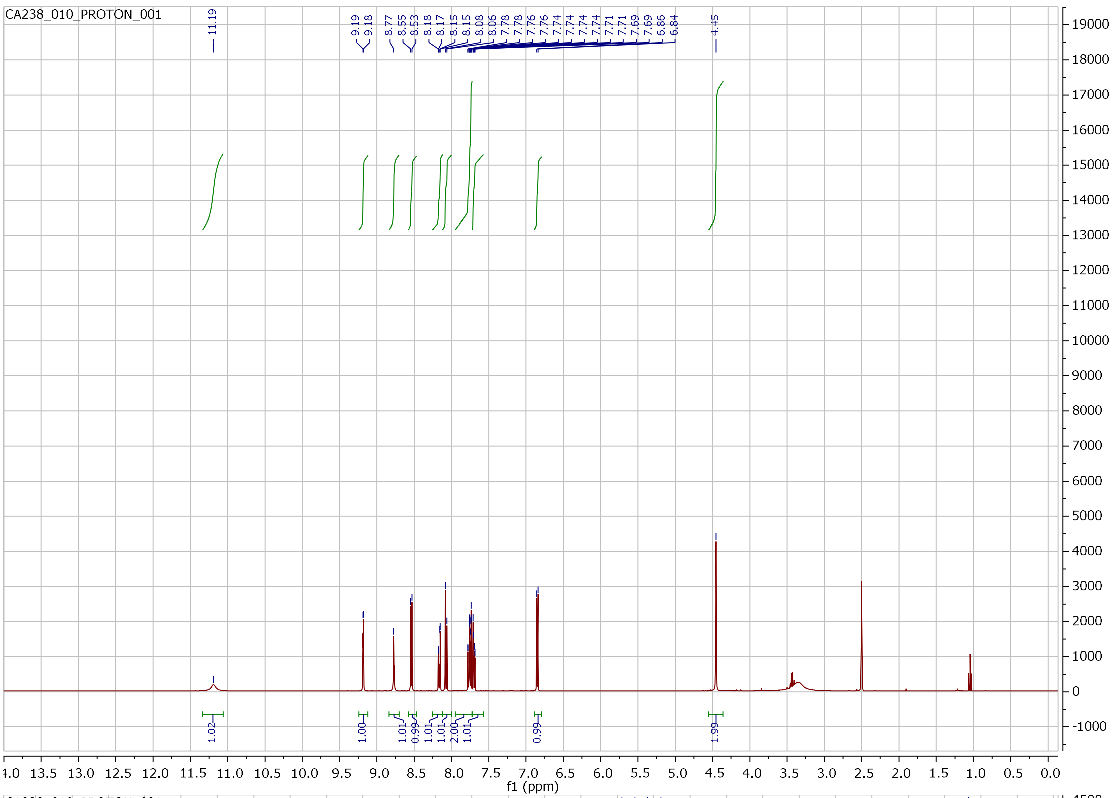
CA238_010_PROTON_001
f1 (ppm)

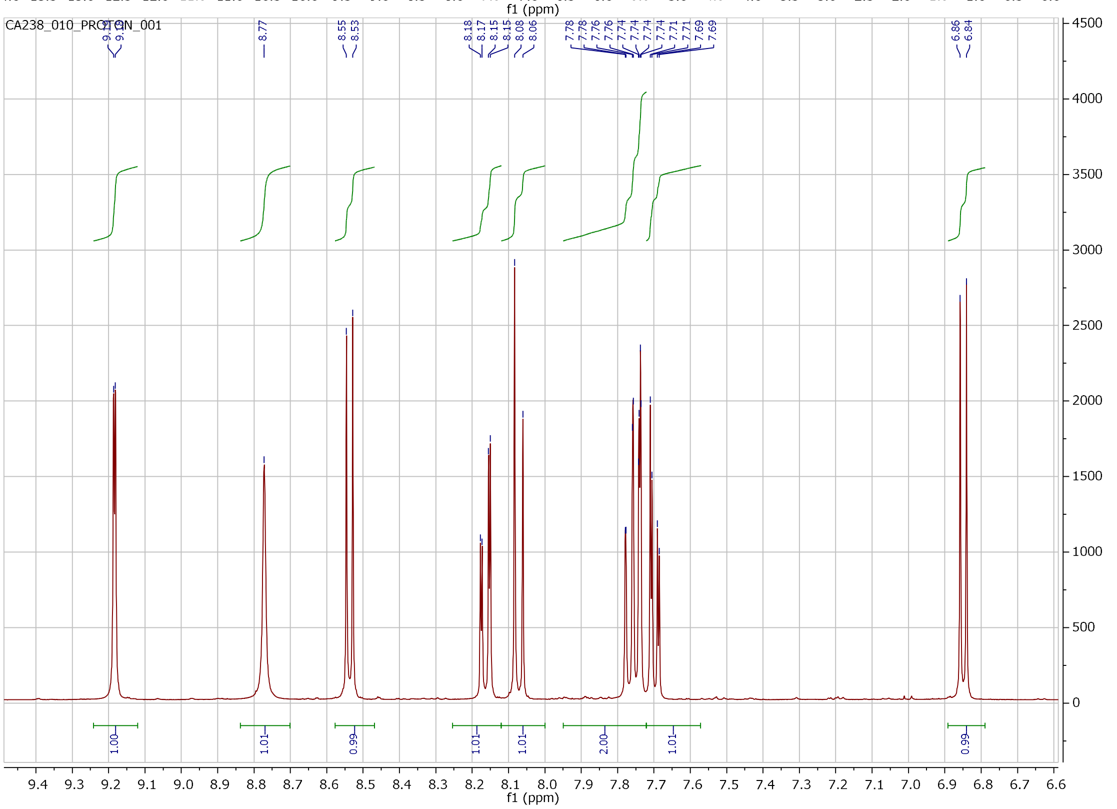
CA238_010_PROTON_001
f1 (ppm)

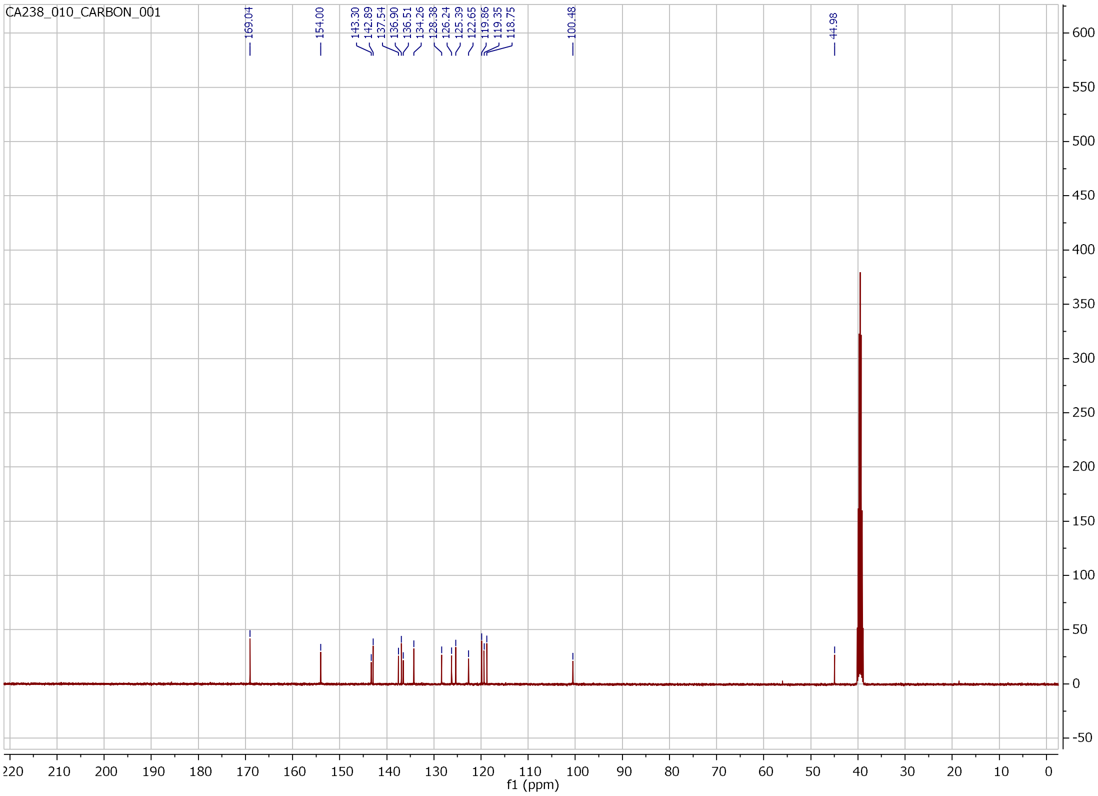

CA238_010_CARBON_001
169.04
154.00
143.30
142.89
137.54
136.90
136.51
134.26
128.38
126.24
125.39
122.65
119.86
119.35
118.75
100.48
44.98
f1 (ppm)

5-[(6-bromoquinolin-4-yl)amino]-2,3-dihydro-1*H*-1,3-benzodiazol-2-one (**39**)

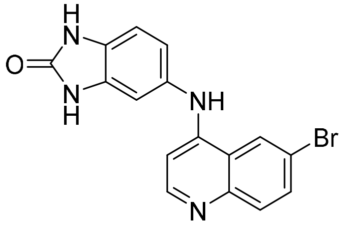

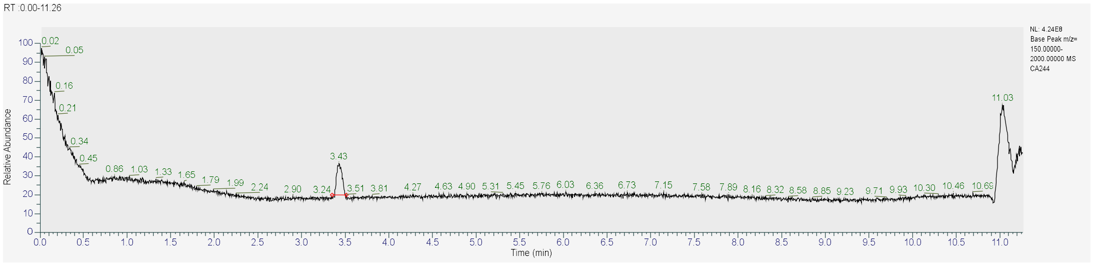

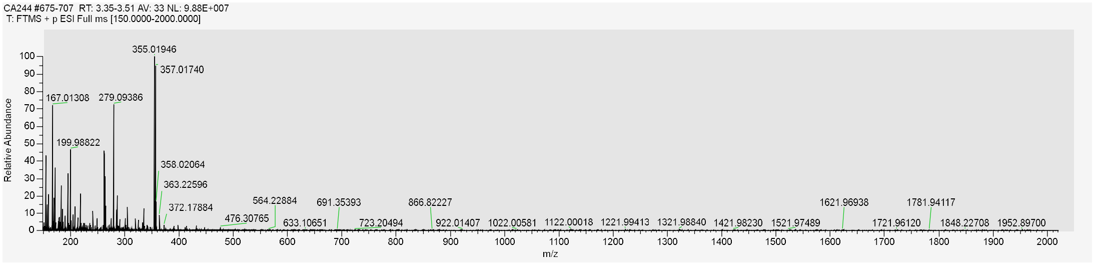

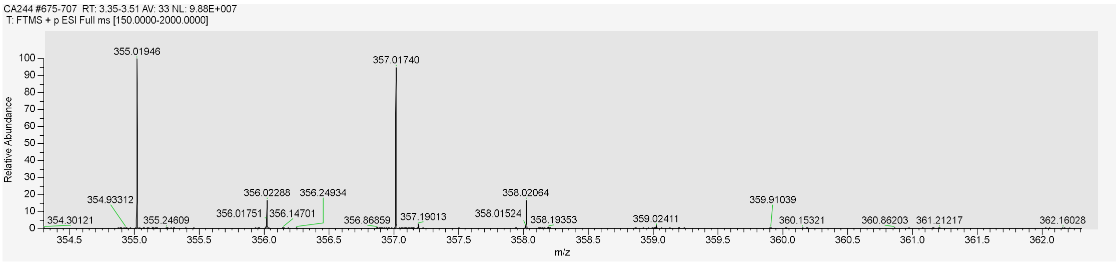

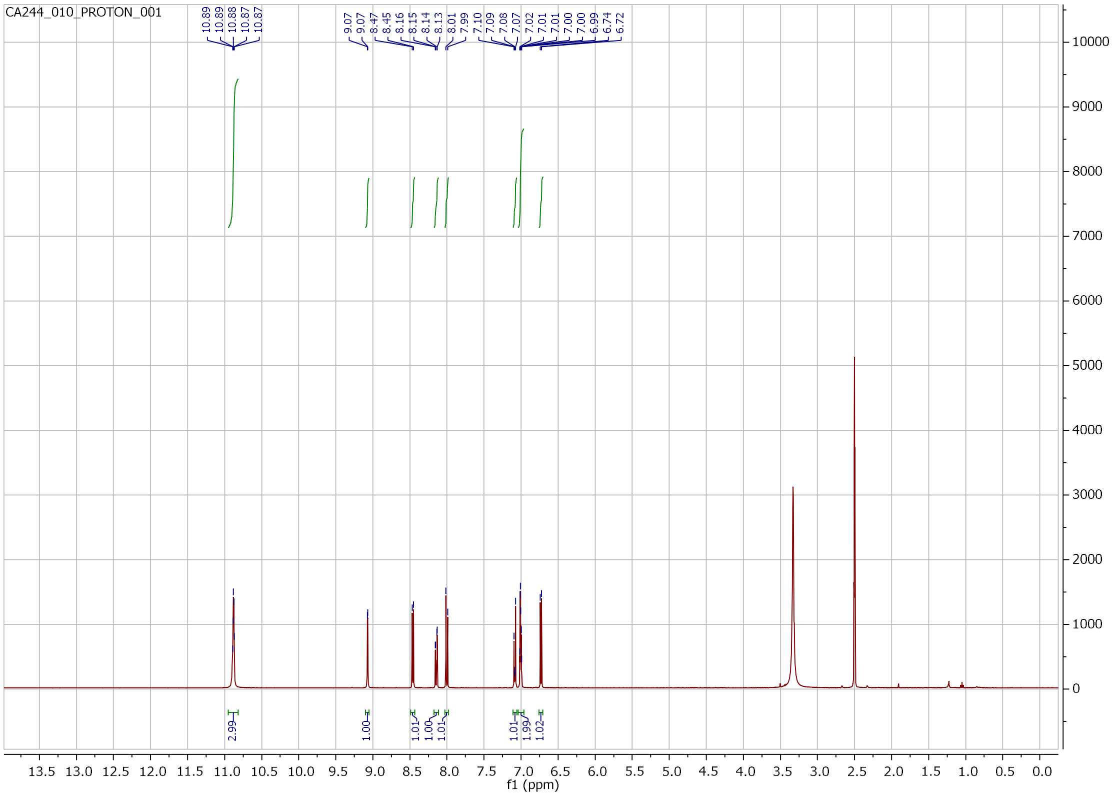

CA244_010_PROTON_001
10.89 10.89 10.88 10.87 10.87
9.07 9.07 8.47 8.45 8.16 8.15 8.14 8.13 8.01 7.99 7.10 7.09 7.08 7.07 7.02 7.01 7.01 7.00 7.00 6.99 6.74 6.72
2.99
1.00
1.01
1.00
1.01
1.01
1.99
1.02
f1 (ppm)

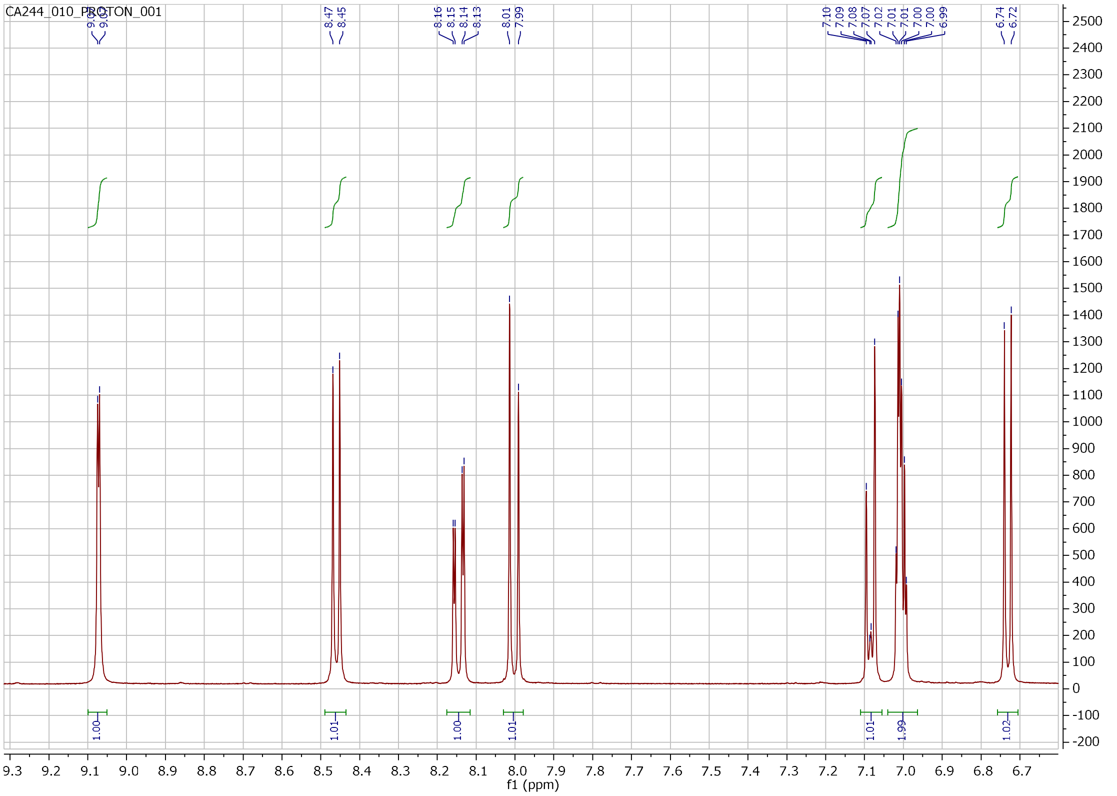

CA244_010_PROTON_001
8.47 8.45
8.16 8.15 8.14 8.13
8.01 7.99
7.10 7.09 7.08 7.07 7.02 7.01 7.01 7.00 7.00 6.99
6.74 6.72
1.00
1.01
1.00
1.01
1.01
1.99
1.02
f1 (ppm)

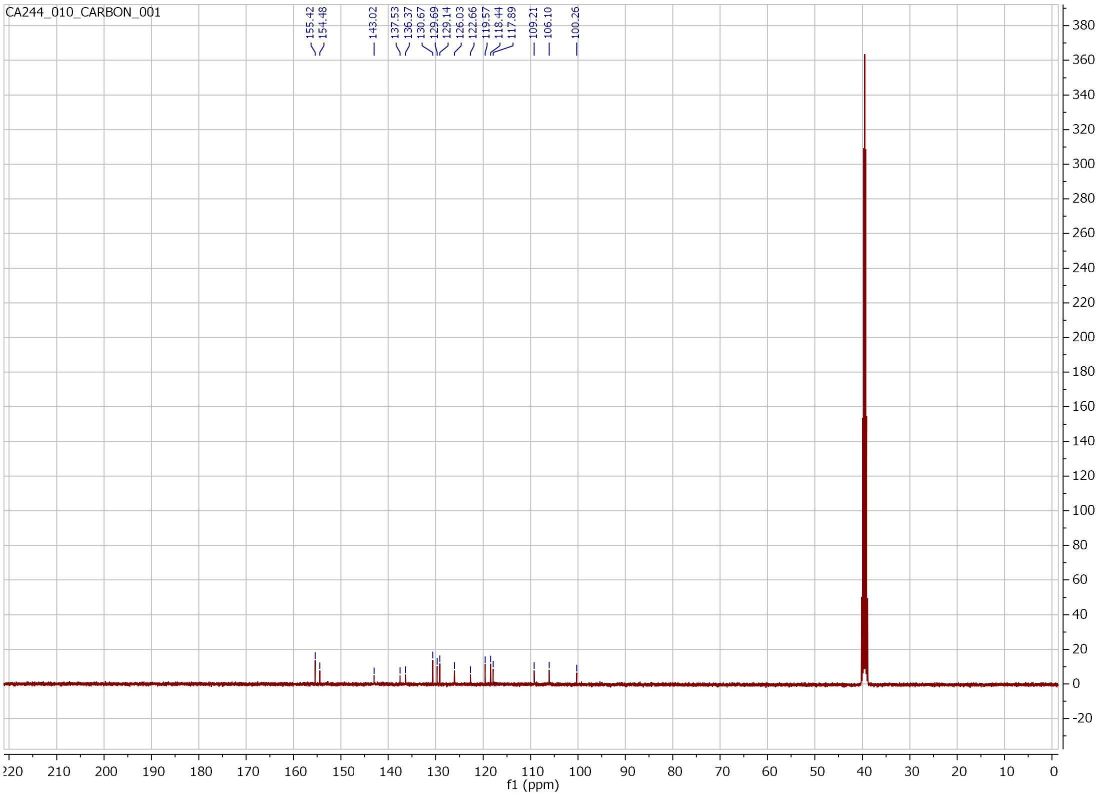

CA244_010_CARBON_001
155.42
154.48
143.02
137.53
136.37
130.67
129.69
129.14
126.03
122.66
119.57
118.44
117.89
109.21
106.10
100.26
f1 (ppm)

CA246 6-[(6-bromoquinolin-4-yl)amino]-2,3-dihydro-1,3-benzoxazol-2-one (**40**)

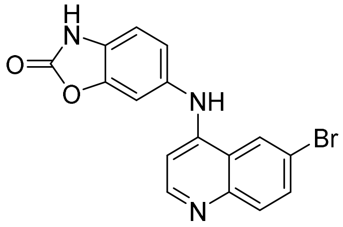

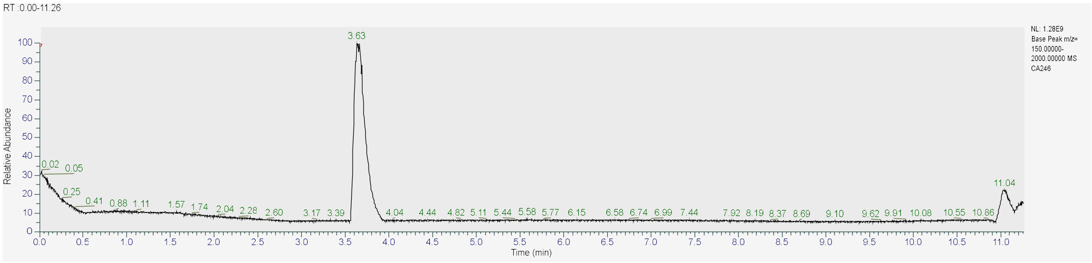

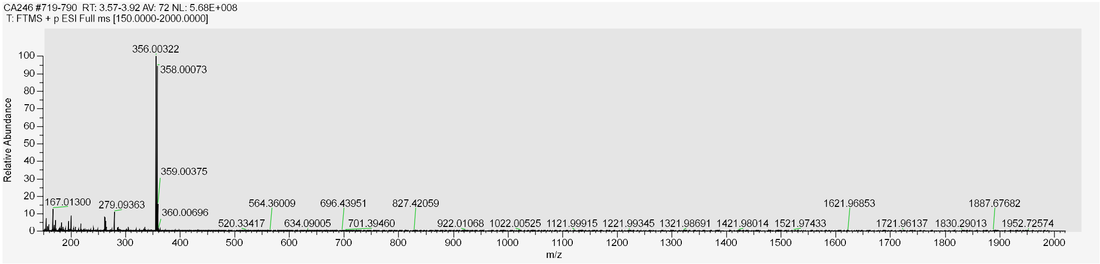

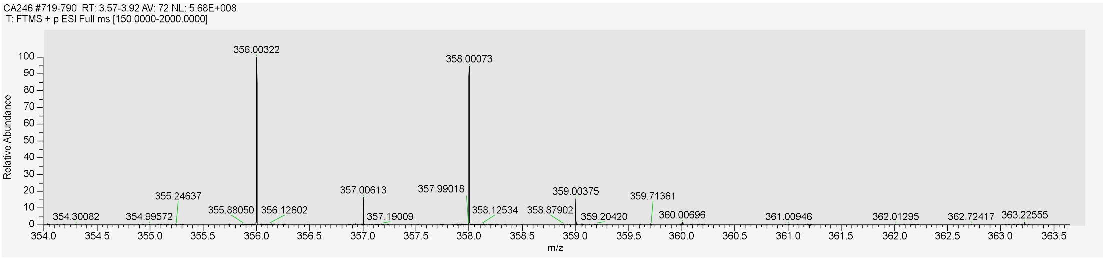

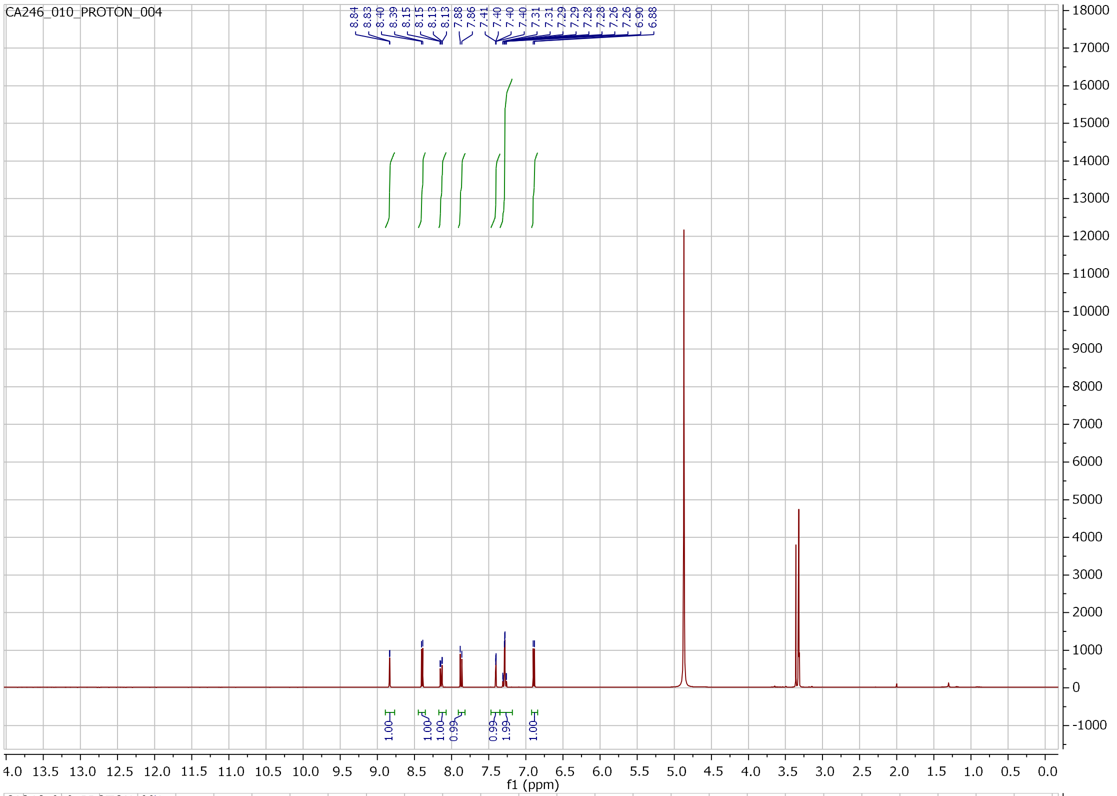

CA246_010_PROTON_004
8.84
8.83
8.40
8.39
8.15
8.15
8.13
8.13
7.88
7.86
7.41
7.40
7.40
7.40
7.31
7.29
7.29
7.28
7.28
7.26
7.26
6.90
6.88
1.00
1.00
1.00
0.99
0.99
1.99
1.00
f1 (ppm)

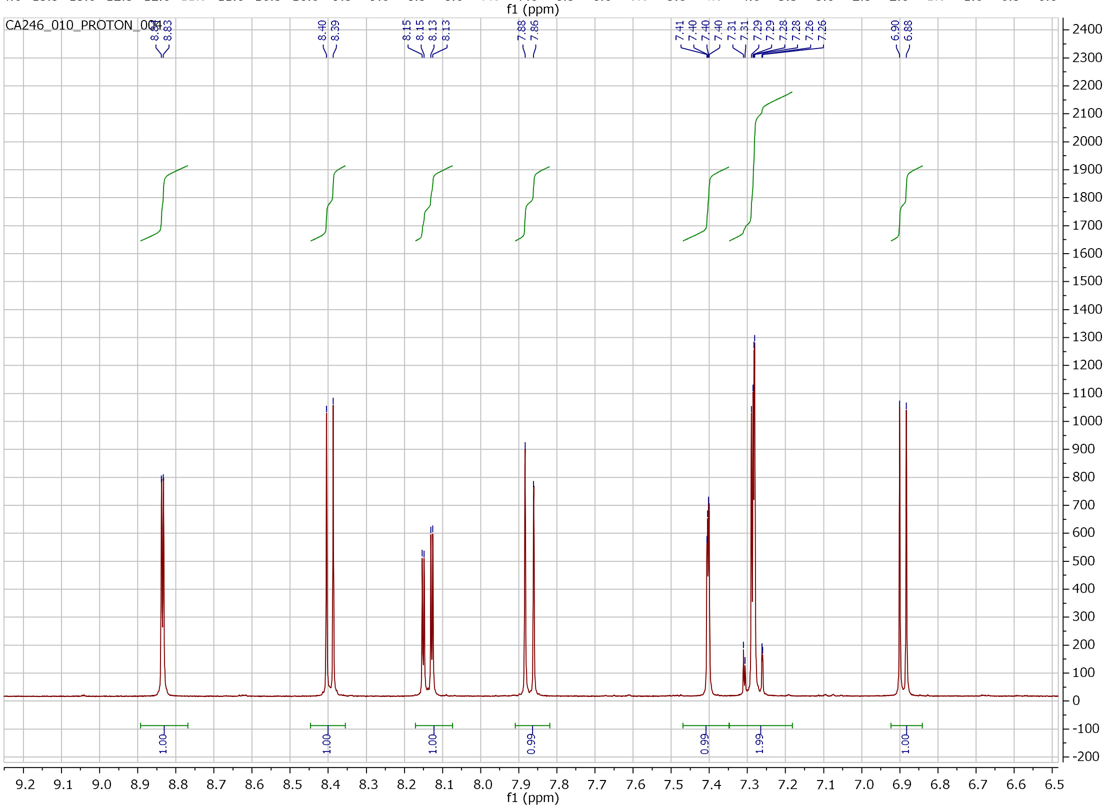

CA246_010_PROTON_004
8.84
8.83
8.40
8.39
8.15
8.15
8.13
8.13
7.88
7.86
7.41
7.40
7.40
7.40
7.31
7.29
7.29
7.28
7.28
7.26
7.26
6.90
6.88
1.00
1.00
1.00
0.99
0.99
1.99
1.00
f1 (ppm)

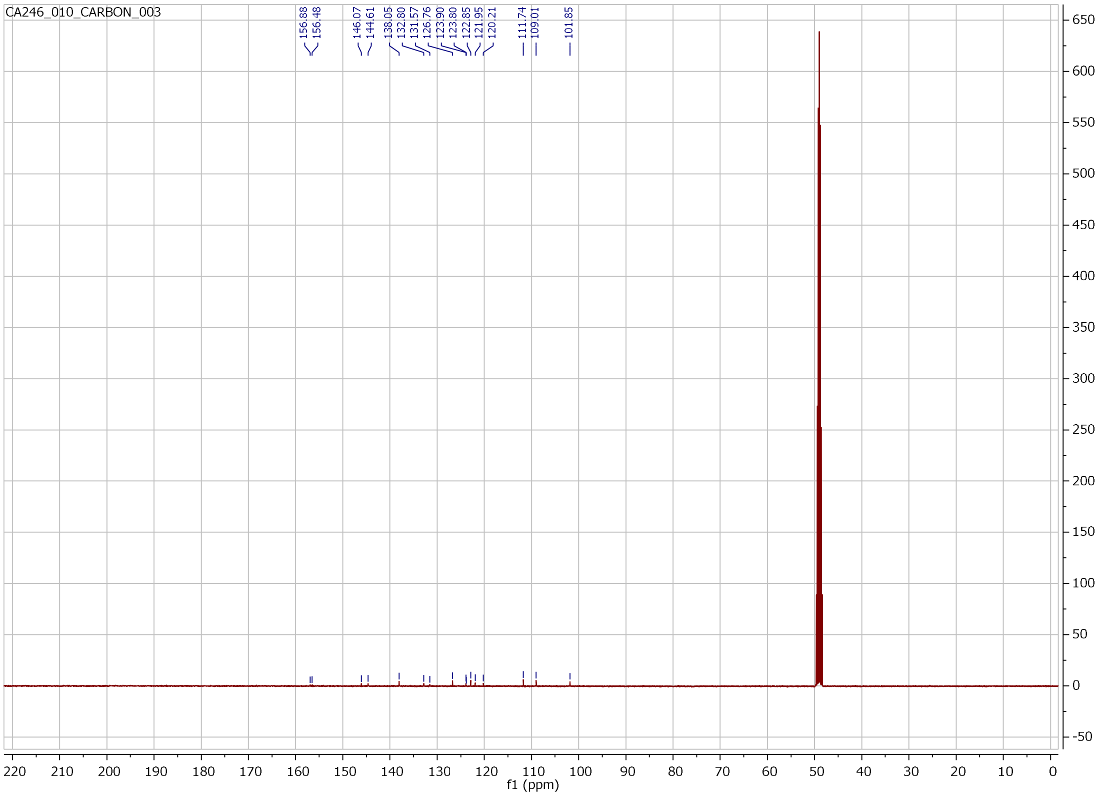
CA246_010_CARBON_003
156.88
156.48
146.07
144.61
138.05
132.80
131.57
126.76
123.90
123.80
122.85
121.95
120.21
111.74
109.01
101.85
f1 (ppm)

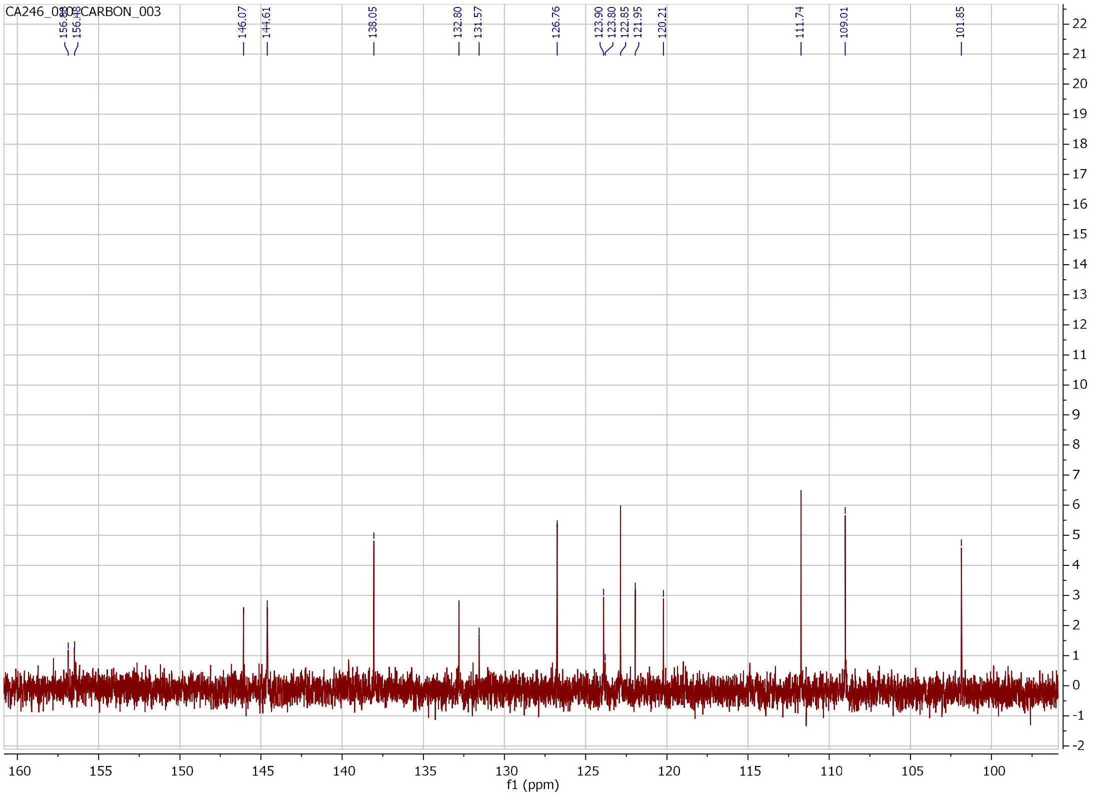
CA246_010_CARBON_003
146.07
144.61
138.05
132.80
131.57
126.76
123.90
123.80
122.85
121.95
120.21
111.74
109.01
101.85
f1 (ppm)

5-[(6-bromoquinolin-4-yl)amino]-2,3-dihydro-1,3-benzoxazol-2-one (**41**)

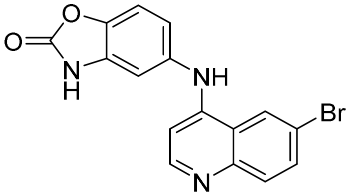

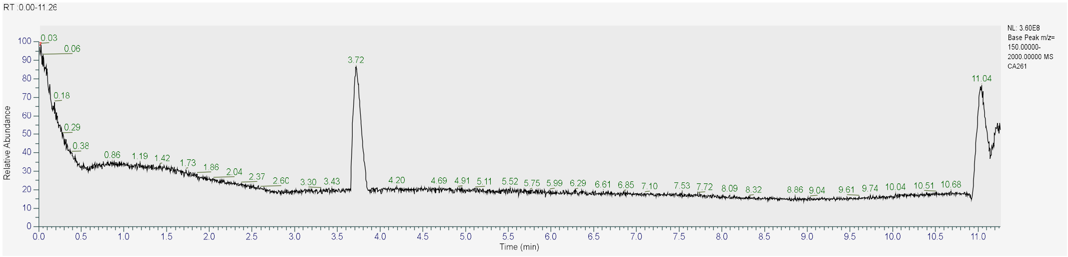

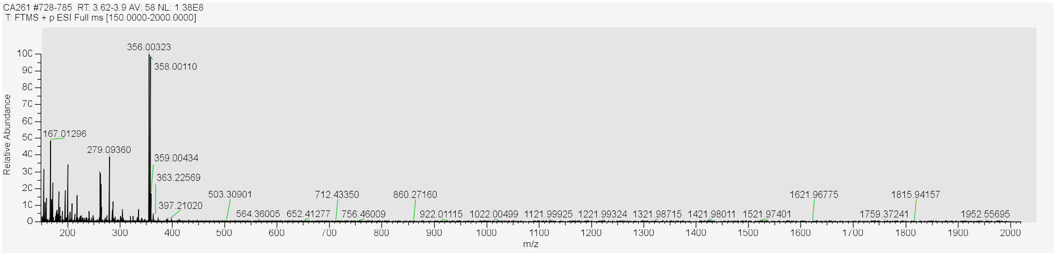

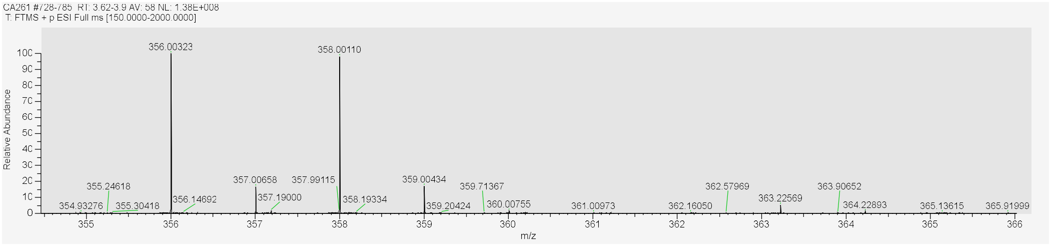

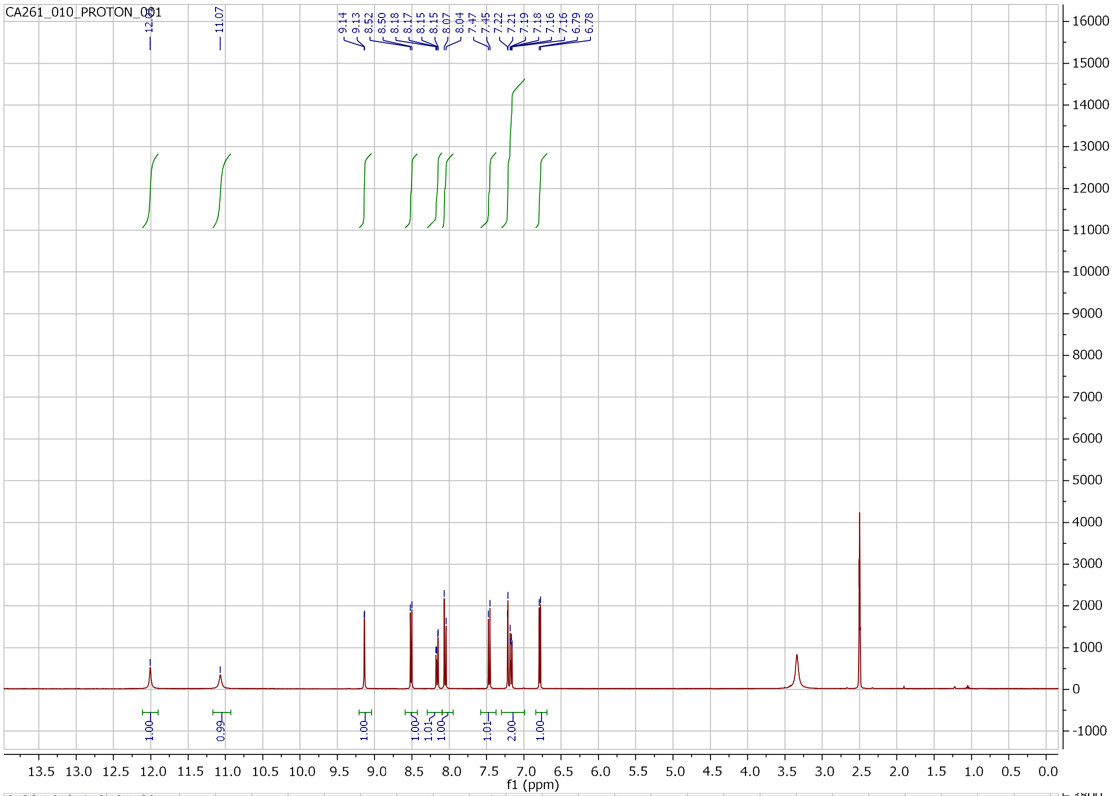
CA261_010_PROTON_001
12.00
11.07
9.14
9.13
8.52
8.50
8.18
8.17
8.15
8.15
8.07
8.04
7.47
7.45
7.22
7.21
7.19
7.18
7.16
6.79
6.78
1.00
0.99
1.00
1.00
1.01
1.00
1.01
2.00
1.00
f1 (ppm)

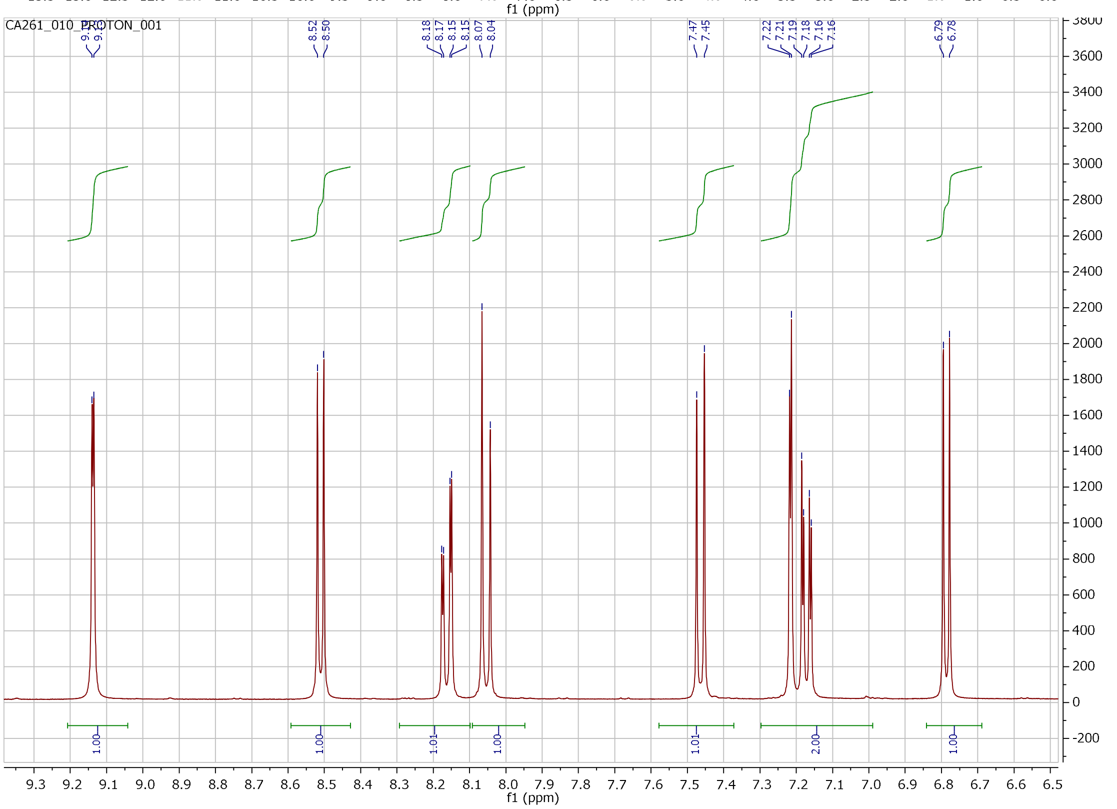
CA261_010_PROTON_001
8.52
8.50
8.18
8.17
8.15
8.15
8.07
8.04
7.47
7.45
7.22
7.21
7.19
7.18
7.16
7.16
6.79
6.78
1.00
1.00
1.01
1.00
1.01
2.00
1.00
f1 (ppm)

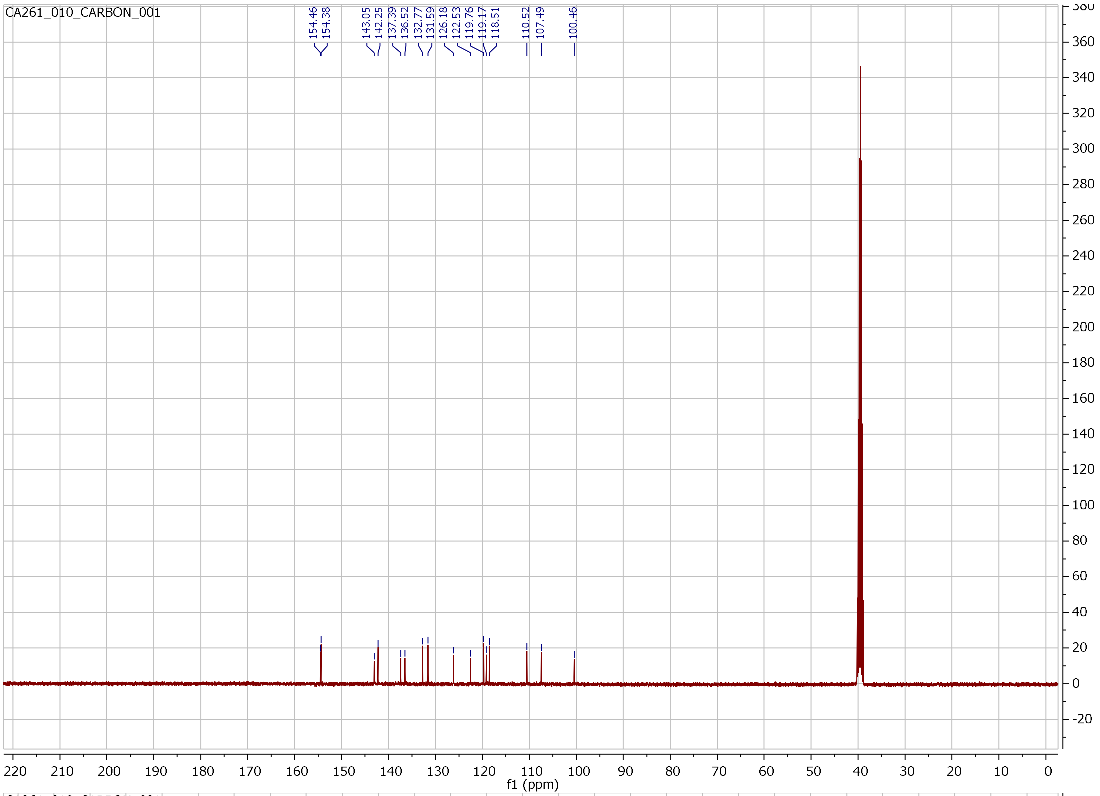
CA261_010_CARBON_001
154.46
154.38
143.05
142.25
137.39
136.52
132.77
131.59
126.18
122.53
119.76
119.17
118.51
110.52
107.49
100.46
f1 (ppm)

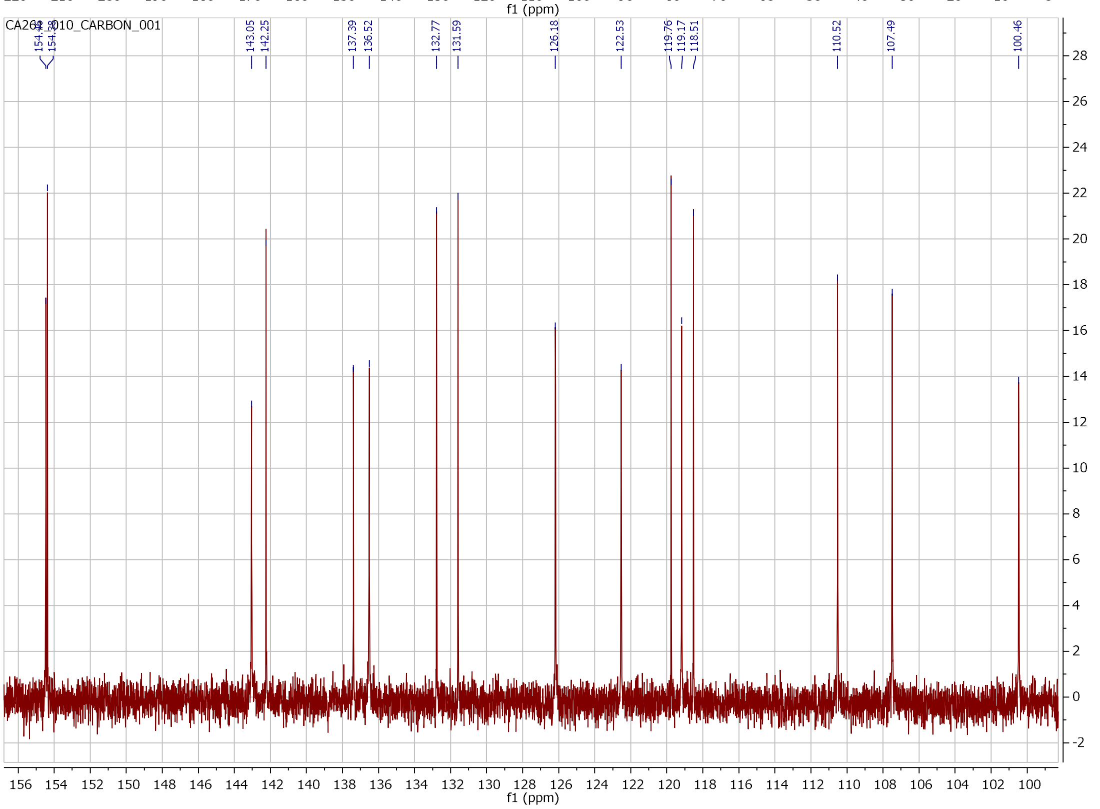
CA261_010_CARBON_001
154.46
154.38
143.05
142.25
137.39
136.52
132.77
131.59
126.18
122.53
119.76
119.17
118.51
110.52
107.49
100.46
f1 (ppm)

5-[(6-bromoquinolin-4-yl)amino]-1-methyl-2,3-dihydro-1*H*-1,3-benzodiazol-2-one (**42**)

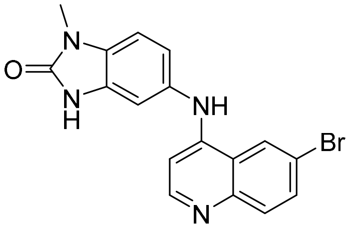

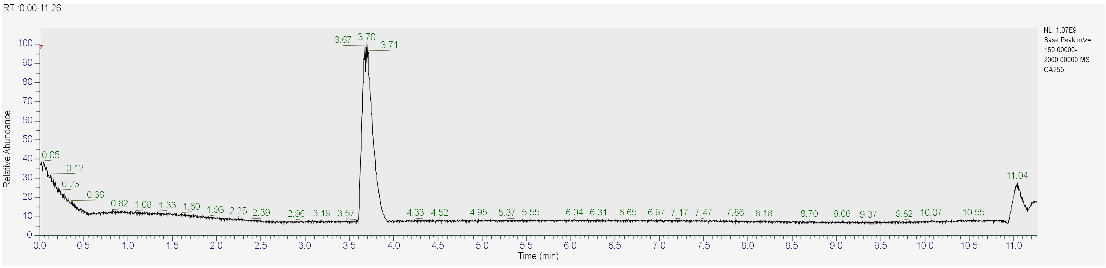

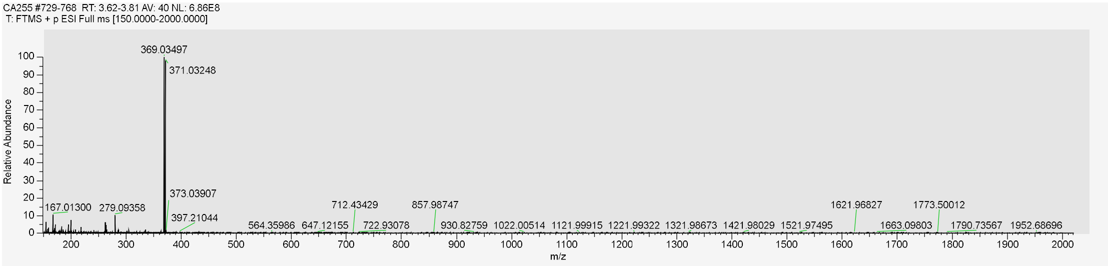

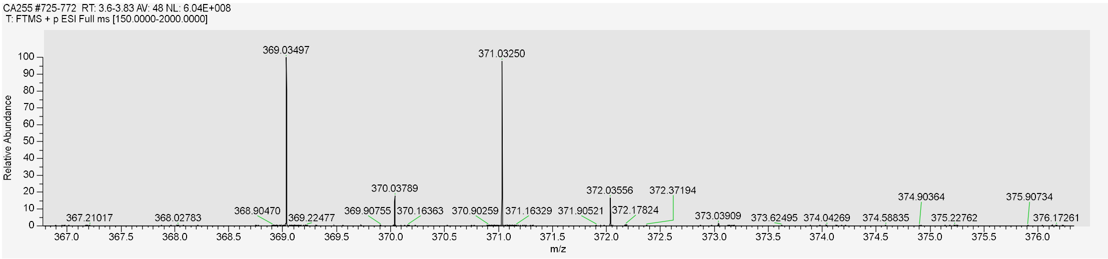

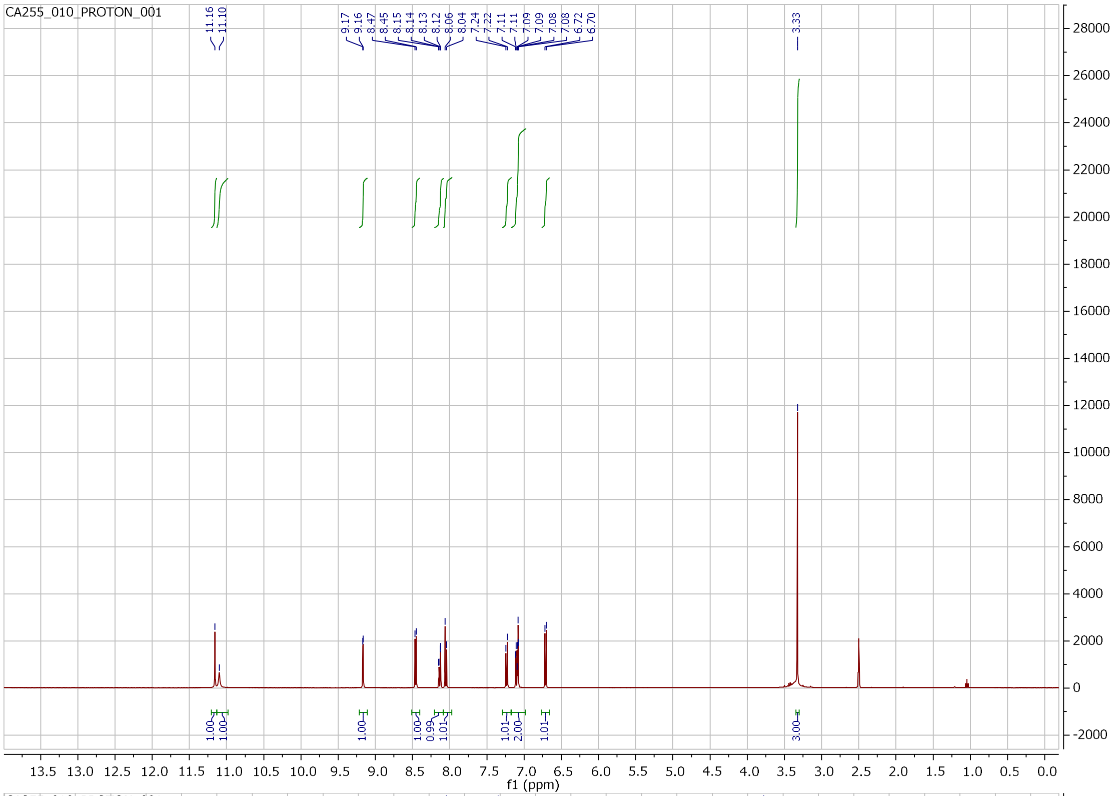
CA255_010_PROTON_001
f1 (ppm)

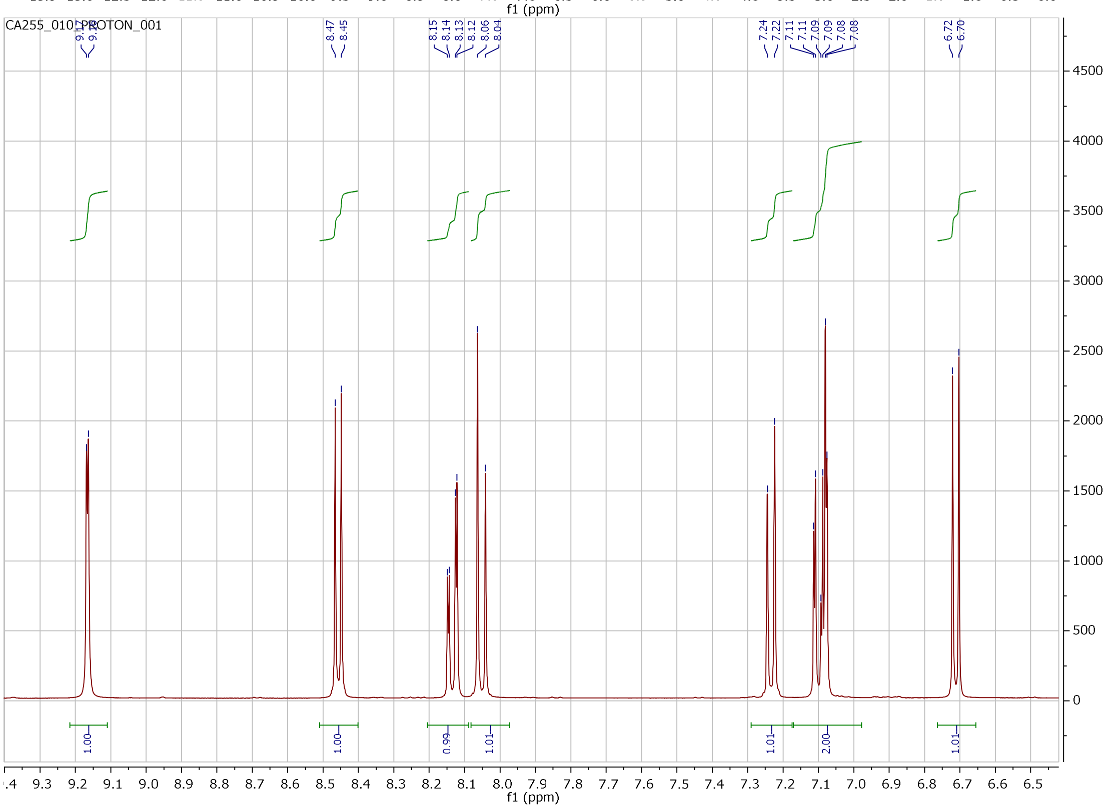
CA255_010_PROTON_001
f1 (ppm)

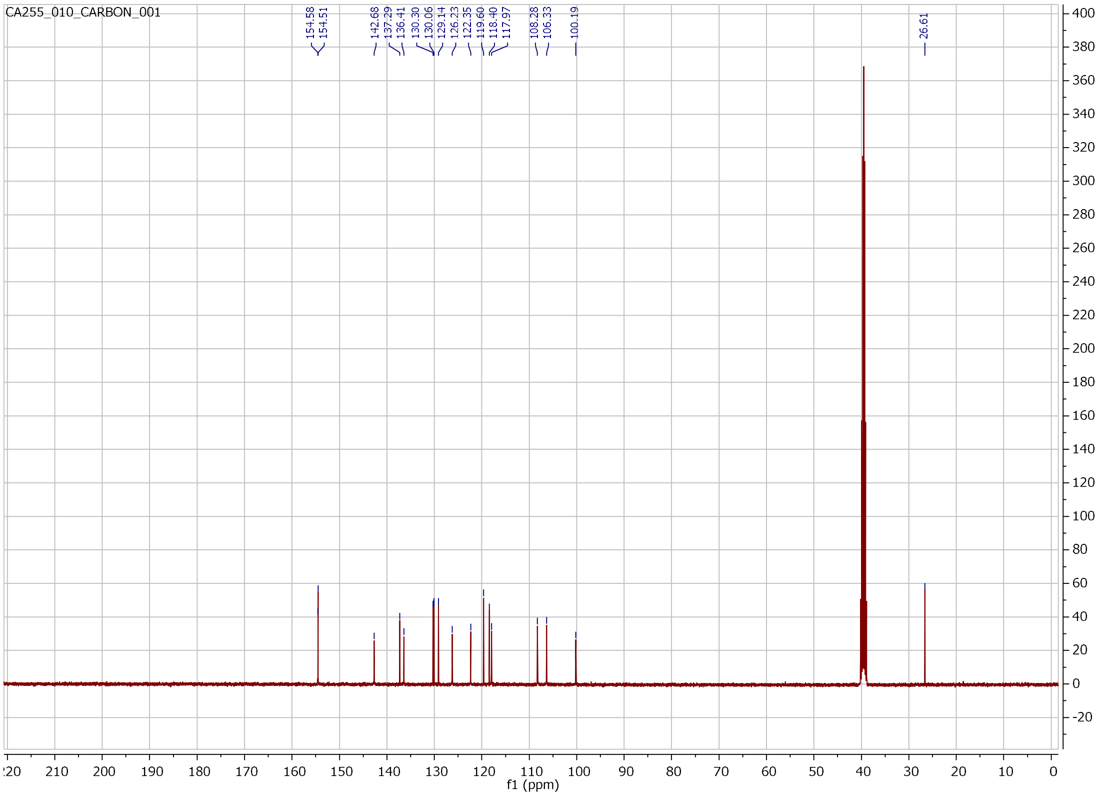

CA255_010_CARBON_001
154.58
154.51
142.68
137.29
136.41
130.30
130.06
129.14
126.23
122.35
119.60
118.40
117.97
108.28
106.33
100.19
26.61
f1 (ppm)

5-((6-bromoquinolin-4-yl)amino)-1,3-dihydrobenzo[c]thiophene 2,2-dioxide (**43**)

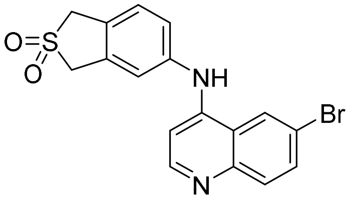

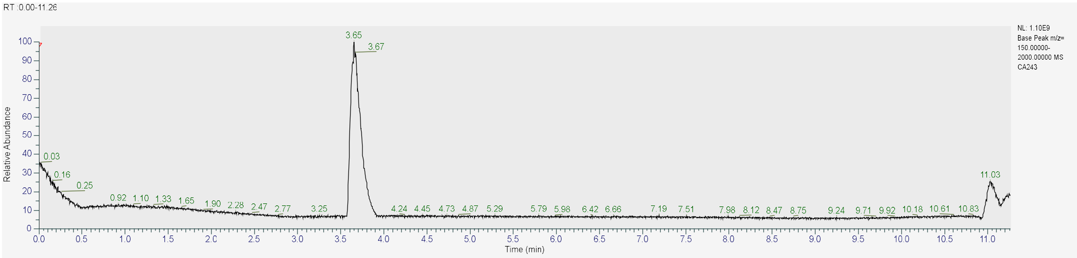

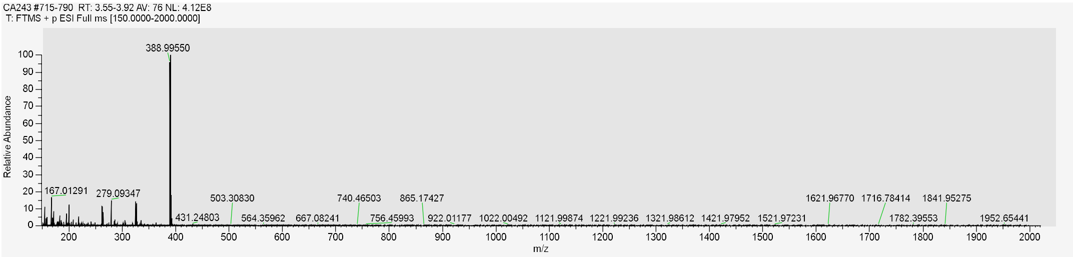

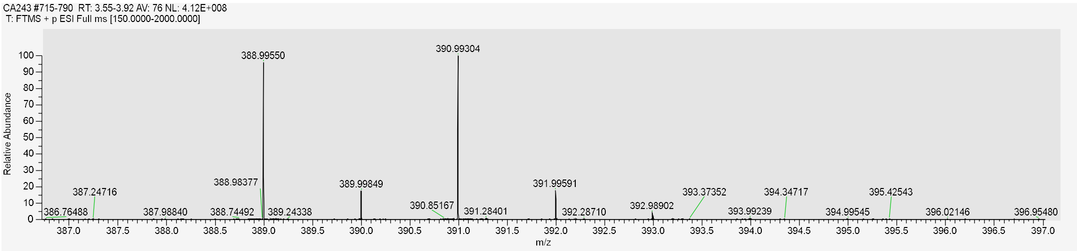

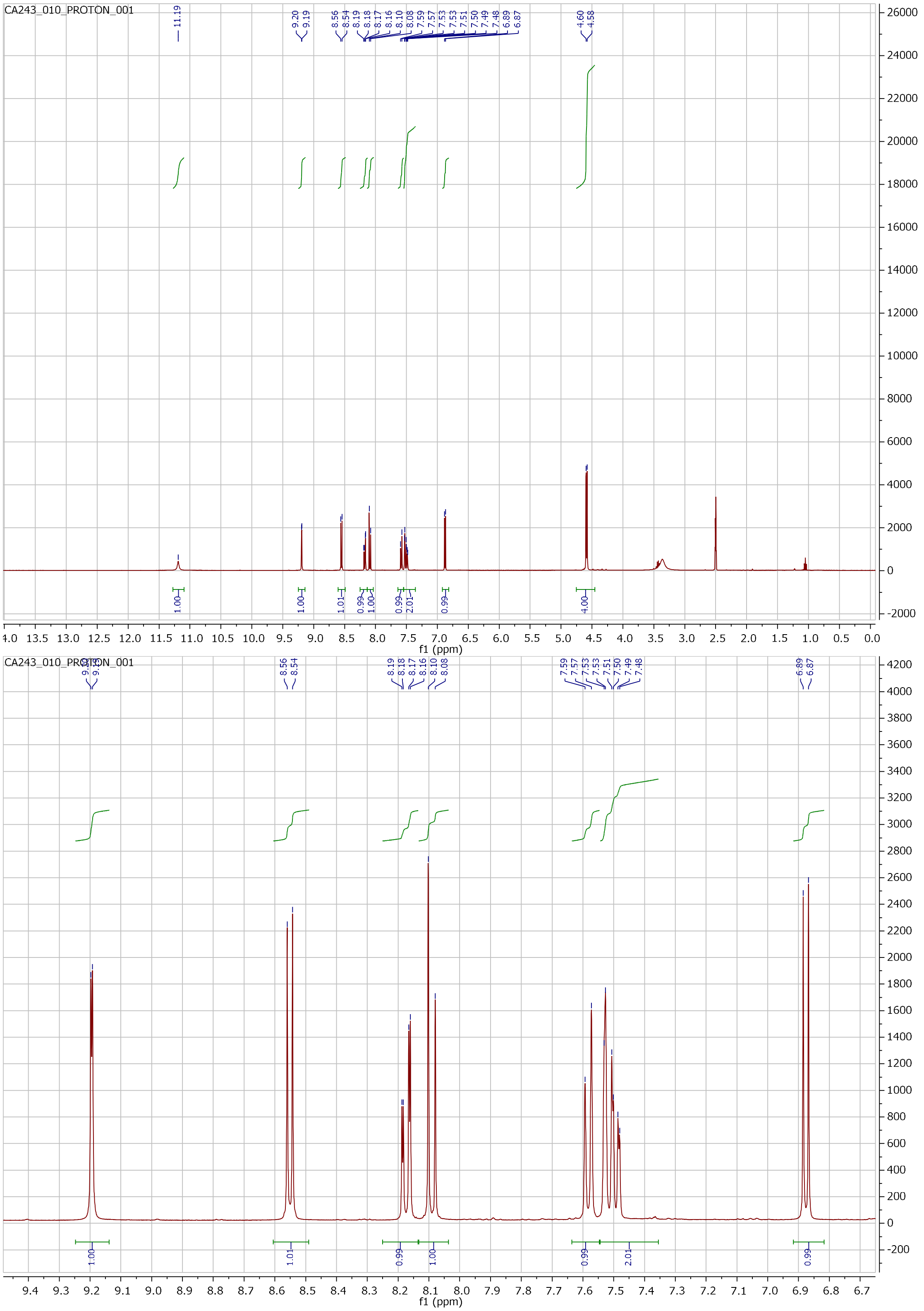
CA243_010_PROTON_001
f1 (ppm)

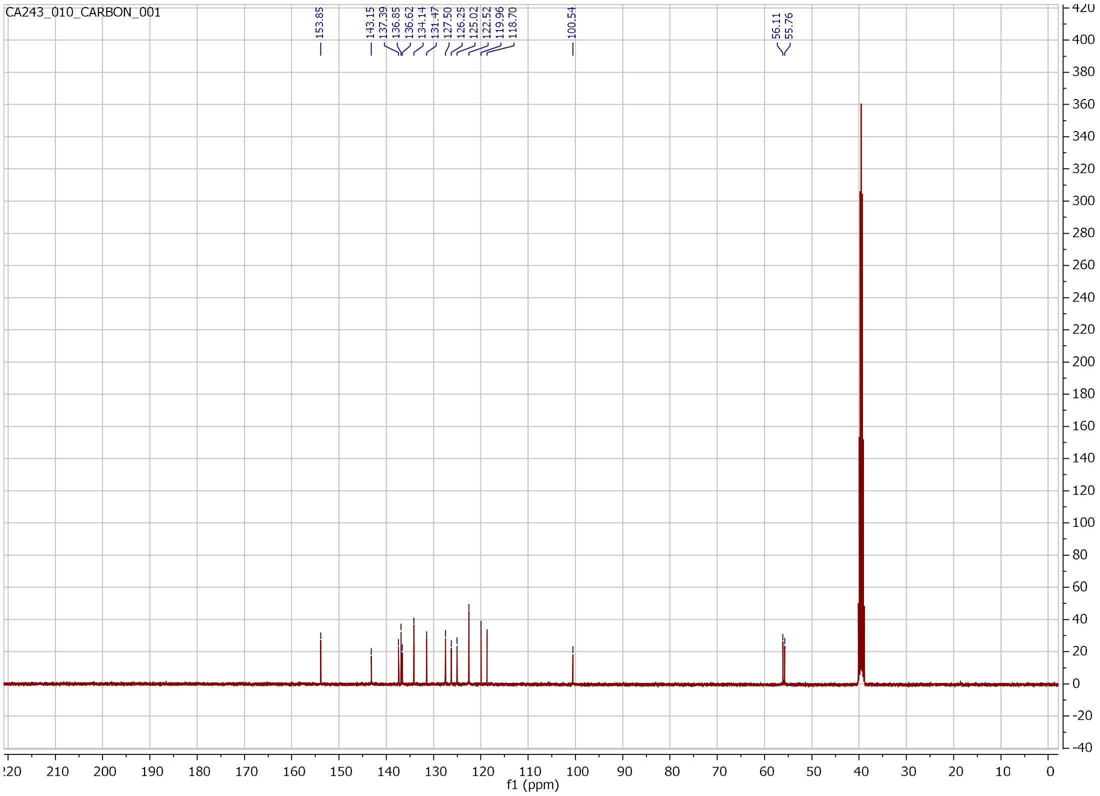

CA243_010_CARBON_001
153.85
143.15
137.39
136.85
136.62
134.14
131.47
127.50
126.25
125.02
122.52
119.96
118.70
100.54
56.11
55.76
f1 (ppm)

11. KINOMEscan® results of 35

Table S13. KINOMEscan® results of **35**

DiscoveRx	Entrez	**35** at 1 μM
Gene Symbol		% inhibition
GAK	GAK	10
MEK5	MAP2K5	11
TGFBR2	TGFBR2	18
RIPK2	RIPK2	29
ACVR2B	ACVR2B	36
ABL1(E255K)-phosphorylated	ABL1	44
ABL1-phosphorylated	ABL1	46
CDKL1	CDKL1	47
MEK2	MAP2K2	50
HIPK1	HIPK1	53
HIPK4	HIPK4	57
RSK1(Kin.Dom.2-C-terminal)	RPS6KA1	57
ABL1(Q252H)-nonphosphorylated	ABL1	58
GCN2(Kin.Dom.2,S808G)	EIF2AK4	59
PRKCI	PRKCI	60
CSNK1A1	CSNK1A1	62
MKNK2	MKNK2	63
PFCDPK1(P.falciparum)	CDPK1	63
ALK(C1156Y)	ALK	64
CHEK2	CHEK2	65
EGFR(L747-S752del, P753S)	EGFR	66
PIK3CA(E545K)	PIK3CA	66
CASK	CASK	68
GRK1	GRK1	68
NDR1	STK38	69
PFTK1	CDK14	69
TNK1	TNK1	69
CDC2L2	CDC2L2	70
RPS6KA5(Kin.Dom.2-C-terminal)	RPS6KA5	70
MEK1	MAP2K1	71
ROCK1	ROCK1	71
CDC2L5	CDK13	73
DMPK2	CDC42BPG	73
LRRK2(G2019S)	LRRK2	73
CDK9	CDK9	75
GRK3	ADRBK2	76
OSR1	OXSR1	76

PIK3CA(I800L)	PIK3CA	76
TBK1	TBK1	76
ABL1(F317I)-nonphosphorylated	ABL1	77
DYRK1A	DYRK1A	77
PKNB(M.tuberculosis)	pknB	77
TSSK3	TSSK3	77
ABL1(F317L)-nonphosphorylated	ABL1	78
CDK4	CDK4	78
EGFR(T790M)	EGFR	78
DDR1	DDR1	79
EGFR(E746-A750del)	EGFR	79
HASPIN	GSG2	79
MKNK1	MKNK1	79
PRP4	PRPF4B	79
BRAF(V600E)	BRAF	80
CDK11	CDK19	80
EGFR(L747-E749del, A750P)	EGFR	81
RSK4(Kin.Dom.2-C-terminal)	RPS6KA6	81
ULK3	ULK3	81
TRKC	NTRK3	82
CDK8	CDK8	83
EGFR(G719C)	EGFR	83
FLT3(R834Q)	FLT3	83
JAK3(JH1domain-catalytic)	JAK3	83
JNK1	MAPK8	83
ABL1(F317L)-phosphorylated	ABL1	85
MERTK	MERTK	85
PAK1	PAK1	85
PAK2	PAK2	85
TRKB	NTRK2	85
ABL1(H396P)-nonphosphorylated	ABL1	86
ALK(L1196M)	ALK	86
CIT	CIT	86
CLK2	CLK2	86
MAP3K1	MAP3K1	86
MEK3	MAP2K3	86
NEK2	NEK2	86
HIPK3	HIPK3	87
PIK3CA(H1047L)	PIK3CA	87
SRMS	SRMS	87
ABL1-nonphosphorylated	ABL1	88
ABL2	ABL2	88

DAPK3	DAPK3	88
HIPK2	HIPK2	88
JAK1(JH2domain-pseudokinase)	JAK1	88
LATS2	LATS2	88
NIM1	MGC42105	88
PRKCQ	PRKCQ	88
EGFR(S752-I759del)	EGFR	89
NDR2	STK38L	89
NEK3	NEK3	89
SYK	SYK	89
AURKB	AURKB	90
FLT3(N841I)	FLT3	90
PRKD2	PRKD2	90
RSK2(Kin.Dom.1-N-terminal)	RPS6KA3	90
BMPR1B	BMPR1B	91
ERN1	ERN1	91
ROCK2	ROCK2	91
S6K1	RPS6KB1	91
TAOK2	TAOK2	91
ULK1	ULK1	91
BRAF	BRAF	92
CAMK1B	PNCK	92
EIF2AK1	EIF2AK1	92
NEK7	NEK7	92
WNK3	WNK3	92
BRK	PTK6	93
BUB1	BUB1	93
CTK	MATK	93
NEK5	NEK5	93
CSNK1D	CSNK1D	94
FLT3(D835H)	FLT3	94
p38-gamma	MAPK12	94
PIK3CB	PIK3CB	94
ROS1	ROS1	94
ULK2	ULK2	94
ABL1(T315I)-phosphorylated	ABL1	95
CSF1R	CSF1R	95
IKK-epsilon	IKBKE	95
PHKG2	PHKG2	95
PIP5K1C	PIP5K1C	95
PIP5K2C	PIP4K2C	95
PRKCE	PRKCE	95

TAOK1	TAOK1	95
TLK1	TLK1	95
TNK2	TNK2	95
BLK	BLK	96
CDK3	CDK3	96
CSNK1A1L	CSNK1A1L	96
FER	FER	96
SGK2	SGK2	96
ACVR1	ACVR1	97
EGFR(G719S)	EGFR	97
ERK4	MAPK4	97
FLT3(ITD)	FLT3	97
FLT3(K663Q)	FLT3	97
GSK3B	GSK3B	97
RET(V804L)	RET	97
SIK2	SIK2	97
SRC	SRC	97
TAOK3	TAOK3	97
TNIK	TNIK	97
WEE2	WEE2	97
EGFR(L858R,T790M)	EGFR	98
JAK2(JH1domain-catalytic)	JAK2	98
MAP3K15	MAP3K15	98
PCTK1	CDK16	98
PIK3CA(E545A)	PIK3CA	98
PKAC-alpha	PRKACA	98
STK16	STK16	98
YSK4	MAP3K19	98
DCAMKL1	DCLK1	99
DCAMKL3	DCLK3	99
FAK	PTK2	99
INSR	INSR	99
PIK3CA(M1043I)	PIK3CA	99
PKN2	PKN2	99
SBK1	SBK1	99
TYRO3	TYRO3	99
AAK1	AAK1	100
ABL1(F317I)-phosphorylated	ABL1	100
ABL1(H396P)-phosphorylated	ABL1	100
ABL1(M351T)-phosphorylated	ABL1	100
ABL1(Q252H)-phosphorylated	ABL1	100
ABL1(T315I)-nonphosphorylated	ABL1	100

ABL1(Y253F)-phosphorylated	ABL1	100
ACVR1B	ACVR1B	100
ACVR2A	ACVR2A	100
ACVRL1	ACVRL1	100
ADCK3	CABC1	100
ADCK4	ADCK4	100
AKT1	AKT1	100
AKT2	AKT2	100
AKT3	AKT3	100
ALK	ALK	100
AMPK-alpha1	PRKAA1	100
AMPK-alpha2	PRKAA2	100
ANKK1	ANKK1	100
ARK5	NUAK1	100
ASK1	MAP3K5	100
ASK2	MAP3K6	100
AURKA	AURKA	100
AURKC	AURKC	100
AXL	AXL	100
BIKE	BMP2K	100
BMPR1A	BMPR1A	100
BMPR2	BMPR2	100
BMX	BMX	100
BRSK1	BRSK1	100
BRSK2	BRSK2	100
BTK	BTK	100
CAMK1	CAMK1	100
CAMK1D	CAMK1D	100
CAMK1G	CAMK1G	100
CAMK2A	CAMK2A	100
CAMK2B	CAMK2B	100
CAMK2D	CAMK2D	100
CAMK2G	CAMK2G	100
CAMK4	CAMK4	100
CAMKK1	CAMKK1	100
CAMKK2	CAMKK2	100
CDC2L1	CDK11B	100
CDK2	CDK2	100
CDK4-cyclinD1	CDK4	100
CDK4-cyclinD3	CDK4	100
CDK5	CDK5	100
CDK7	CDK7	100

CDKL2	CDKL2	100
CDKL3	CDKL3	100
CDKL5	CDKL5	100
CHEK1	CHEK1	100
CLK1	CLK1	100
CLK3	CLK3	100
CLK4	CLK4	100
CSF1R-autoinhibited	CSF1R	100
CSK	CSK	100
CSNK1E	CSNK1E	100
CSNK1G1	CSNK1G1	100
CSNK1G2	CSNK1G2	100
CSNK1G3	CSNK1G3	100
CSNK2A1	CSNK2A1	100
CSNK2A2	CSNK2A2	100
DAPK1	DAPK1	100
DAPK2	DAPK2	100
DCAMKL2	DCLK2	100
DDR2	DDR2	100
DLK	MAP3K12	100
DMPK	DMPK	100
DRAK1	STK17A	100
DRAK2	STK17B	100
DYRK1B	DYRK1B	100
DYRK2	DYRK2	100
EGFR	EGFR	100
EGFR(L747-T751del,Sins)	EGFR	100
EGFR(L858R)	EGFR	100
EGFR(L861Q)	EGFR	100
EPHA1	EPHA1	100
EPHA2	EPHA2	100
EPHA3	EPHA3	100
EPHA4	EPHA4	100
EPHA5	EPHA5	100
EPHA6	EPHA6	100
EPHA7	EPHA7	100
EPHA8	EPHA8	100
EPHB1	EPHB1	100
EPHB2	EPHB2	100
EPHB3	EPHB3	100
EPHB4	EPHB4	100
EPHB6	EPHB6	100

ERBB2	ERBB2	100
ERBB3	ERBB3	100
ERBB4	ERBB4	100
ERK1	MAPK3	100
ERK2	MAPK1	100
ERK3	MAPK6	100
ERK5	MAPK7	100
ERK8	MAPK15	100
FES	FES	100
FGFR1	FGFR1	100
FGFR2	FGFR2	100
FGFR3	FGFR3	100
FGFR3(G697C)	FGFR3	100
FGFR4	FGFR4	100
FGR	FGR	100
FLT1	FLT1	100
FLT3	FLT3	100
FLT3(D835V)	FLT3	100
FLT3(D835Y)	FLT3	100
FLT3(ITD,D835V)	FLT3	100
FLT3(ITD,F691L)	FLT3	100
FLT3-autoinhibited	FLT3	100
FLT4	FLT4	100
FRK	FRK	100
FYN	FYN	100
GRK2	ADRBK1	100
GRK4	GRK4	100
GRK7	GRK7	100
GSK3A	GSK3A	100
HCK	HCK	100
HPK1	MAP4K1	100
HUNK	HUNK	100
ICK	ICK	100
IGF1R	IGF1R	100
IKK-alpha	CHUK	100
IKK-beta	IKBKB	100
INSRR	INSRR	100
IRAK1	IRAK1	100
IRAK3	IRAK3	100
IRAK4	IRAK4	100
ITK	ITK	100
JAK1(JH1domain-catalytic)	JAK1	100

JNK2	MAPK9	100
JNK3	MAPK10	100
KIT	KIT	100
KIT(A829P)	KIT	100
KIT(D816H)	KIT	100
KIT(D816V)	KIT	100
KIT(L576P)	KIT	100
KIT(V559D)	KIT	100
KIT(V559D,T670I)	KIT	100
KIT(V559D,V654A)	KIT	100
KIT-autoinhibited	KIT	100
LATS1	LATS1	100
LCK	LCK	100
LIMK1	LIMK1	100
LIMK2	LIMK2	100
LKB1	STK11	100
LOK	STK10	100
LRRK2	LRRK2	100
LTK	LTK	100
LYN	LYN	100
LZK	MAP3K13	100
MAK	MAK	100
MAP3K2	MAP3K2	100
MAP3K3	MAP3K3	100
MAP3K4	MAP3K4	100
MAP4K2	MAP4K2	100
MAP4K3	MAP4K3	100
MAP4K4	MAP4K4	100
MAP4K5	MAP4K5	100
MAPKAPK2	MAPKAPK2	100
MAPKAPK5	MAPKAPK5	100
MARK1	MARK1	100
MARK2	MARK2	100
MARK3	MARK3	100
MARK4	MARK4	100
MAST1	MAST1	100
MEK4	MAP2K4	100
MEK6	MAP2K6	100
MELK	MELK	100
MET	MET	100
MET(M1250T)	MET	100
MET(Y1235D)	MET	100

MINK	MINK1	100
MKK7	MAP2K7	100
MLCK	MYLK3	100
MLK1	MAP3K9	100
MLK2	MAP3K10	100
MLK3	MAP3K11	100
MRCKA	CDC42BPA	100
MRCKB	CDC42BPB	100
MST1	STK4	100
MST1R	MST1R	100
MST2	STK3	100
MST3	STK24	100
MST4	MST4	100
MTOR	MTOR	100
MUSK	MUSK	100
MYLK	MYLK	100
MYLK2	MYLK2	100
MYLK4	MYLK4	100
MYO3A	MYO3A	100
MYO3B	MYO3B	100
NEK1	NEK1	100
NEK10	NEK10	100
NEK11	NEK11	100
NEK4	NEK4	100
NEK6	NEK6	100
NEK9	NEK9	100
NIK	MAP3K14	100
NLK	NLK	100
p38-alpha	MAPK14	100
p38-beta	MAPK11	100
p38-delta	MAPK13	100
PAK3	PAK3	100
PAK4	PAK4	100
PAK6	PAK6	100
PAK7	PAK7	100
PCTK2	CDK17	100
PCTK3	CDK18	100
PDGFRA	PDGFRA	100
PDGFRB	PDGFRB	100
PDPK1	PDPK1	100
PFPK5(P.falciparum)	MAL13P1.279	100
PFTAIRE2	CDK15	100

PHKG1	PHKG1	100
PIK3C2B	PIK3C2B	100
PIK3C2G	PIK3C2G	100
PIK3CA	PIK3CA	100
PIK3CA(C420R)	PIK3CA	100
PIK3CA(E542K)	PIK3CA	100
PIK3CA(H1047Y)	PIK3CA	100
PIK3CA(Q546K)	PIK3CA	100
PIK3CD	PIK3CD	100
PIK3CG	PIK3CG	100
PIK4CB	PI4KB	100
PIKFYVE	PIKFYVE	100
PIM1	PIM1	100
PIM2	PIM2	100
PIM3	PIM3	100
PIP5K1A	PIP5K1A	100
PIP5K2B	PIP4K2B	100
PKAC-beta	PRKACB	100
PKMYT1	PKMYT1	100
PKN1	PKN1	100
PLK1	PLK1	100
PLK2	PLK2	100
PLK3	PLK3	100
PLK4	PLK4	100
PRKCD	PRKCD	100
PRKCH	PRKCH	100
PRKD1	PRKD1	100
PRKD3	PRKD3	100
PRKG1	PRKG1	100
PRKG2	PRKG2	100
PRKR	EIF2AK2	100
PRKX	PRKX	100
PYK2	PTK2B	100
QSK	KIAA0999	100
RAF1	RAF1	100
RET	RET	100
RET(M918T)	RET	100
RET(V804M)	RET	100
RIOK1	RIOK1	100
RIOK2	RIOK2	100
RIOK3	RIOK3	100
RIPK1	RIPK1	100

RIPK4	RIPK4	100
RIPK5	DSTYK	100
RPS6KA4(Kin.Dom.1-N-terminal)	RPS6KA4	100
RPS6KA4(Kin.Dom.2-C-terminal)	RPS6KA4	100
RPS6KA5(Kin.Dom.1-N-terminal)	RPS6KA5	100
RSK1(Kin.Dom.1-N-terminal)	RPS6KA1	100
RSK2(Kin.Dom.2-C-terminal)	RPS6KA3	100
RSK3(Kin.Dom.1-N-terminal)	RPS6KA2	100
RSK3(Kin.Dom.2-C-terminal)	RPS6KA2	100
RSK4(Kin.Dom.1-N-terminal)	RPS6KA6	100
SGK	SGK1	100
SgK110	SgK110	100
SGK3	SGK3	100
SIK	SIK1	100
SLK	SLK	100
SNARK	NUAK2	100
SNRK	SNRK	100
SRPK1	SRPK1	100
SRPK2	SRPK2	100
SRPK3	SRPK3	100
STK33	STK33	100
STK35	STK35	100
STK36	STK36	100
STK39	STK39	100
TAK1	MAP3K7	100
TEC	TEC	100
TESK1	TESK1	100
TGFBR1	TGFBR1	100
TIE1	TIE1	100
TIE2	TEK	100
TLK2	TLK2	100
TNNI3K	TNNI3K	100
TRKA	NTRK1	100
TRPM6	TRPM6	100
TSSK1B	TSSK1B	100
TTK	TTK	100
TXK	TXK	100
TYK2(JH1domain-catalytic)	TYK2	100
TYK2(JH2domain-pseudokinase)	TYK2	100
VEGFR2	KDR	100
VPS34	PIK3C3	100
VRK2	VRK2	100

WEE1	WEE1	100
WNK1	WNK1	100
WNK2	WNK2	100
WNK4	WNK4	100
YANK1	STK32A	100
YANK2	STK32B	100
YANK3	STK32C	100
YES	YES1	100
YSK1	STK25	100
ZAK	ZAK	100
ZAP70	ZAP70	100
